# Identification and characterization of circular RNAs in *Ganoderma lucidum*

**DOI:** 10.1038/s41598-019-52932-w

**Published:** 2019-11-11

**Authors:** Junjie Shao, Liqiang Wang, Xinyue Liu, Meng Yang, Haimei Chen, Bin Wu, Chang Liu

**Affiliations:** 1 Institute of Medicinal Plant Development, Chinese Academy of Medical Sciences & Peking Union Medical College, No. 151 Malianwa North Road, Haidian District Beijing, 100193 P.R. China; 2 School of Chinese Materia Medica, Beijing University of Chinese Medicine, Beijing, 100029 P.R. China

**Keywords:** Data mining, Data publication and archiving, Long non-coding RNAs

## Abstract

Circular RNAs (circRNAs) play important roles in animals, plants, and fungi. However, no circRNAs have been reported in *Ganoderma lucidum*. Here, we carried out a genome-wide identification of the circRNAs in *G.lucidum* using RNA-Seq data, and analyzed their features. In total, 250 and 2193 circRNAs were identified from strand-specific RNA-seq data generated from the polyA(−) and polyA(−)/RNase R-treated libraries, respectively. Six of 131 (4.58%) predicted circRNAs were experimentally confirmed. Across three developmental stages, 731 exonic circRNAs (back spliced read counts ≥ 5) and their parent genes were further analyzed. CircRNAs were preferred originating from exons with flanking introns, and the lengths of the flanking intron were longer than those of the control introns. A total of 200 circRNAs were differentially expressed across the three developmental stages of *G. lucidum*. The expression profiles of 119 (16.3%) exonic circRNAs and their parent genes showed significant positive correlations (*r* ≥ 0.9, *q* < 0.01), whereas 226 (30.9%) exonic circRNAs and their parent genes exhibited significant negative correlations (*r* ≤ −0.9, *q* < 0.01), in which 53 parent genes are potentially involved in the transcriptional regulation, polysaccharide biosynthesis etc. Our results indicated that circRNAs are present in *G. lucidum*, with potentially important regulatory roles.

## Introduction

Circular RNAs (circRNAs) are a type of endogenous noncoding RNAs. Their 5′- and 3′-ends are jointed together, thereby forming covalently closed loop structures. CircRNAs were first discovered when studying the viroid structure in 1976^1^. Over the following years, only a few circRNAs were found^2–6^. In 2012, with the advancement in high-throughput DNA sequencing technologies and bioinformatic data analysis methods, circRNAs were identified in numerous organisms, such as Archaea^7^, *Caenorhabditis elegan*^8^, yeast^9^, mice^10^, humans^11^, *Arabidopsis*^12^, rice^13^, *Triticum aestivum*^14^, tomato^15^, and soybean^16^. After microRNAs (miRNAs) and long noncoding RNAs (lncRNA), circRNAs become a hot research area in molecular biology recently. However, to our knowledge, no circRNAs have been described in basidiomycetes to date.

The circRNA biogenesis is considerably complex. CircRNAs can originate from lariat-driven circularization or exon skipping^17^, intron-pairing-driven circularization or direct backsplicing^17^, intron circularization by tail trimming^18^, and RBP or trans-factor-driven circularization^19^. On the basis of the biogenesis types, circRNAs can be classified as follows: exonic circRNAs^7,8^, circular intronic RNAs^18^, and retained-intron circRNAs^10^.

CircRNAs are detected in a sequencing library constructed with rRNA depletion but without mRNA enrichment (also called polyA(−) library). In addition, the total RNAs can be treated with RNase, which selectively degrades linear RNA molecules, followed by the standard polyA(−) library construction procedure. This library construction method is called polyA(−)/RNase R. The circRNA expression is tissue- or development-specific^13,20^. CircRNAs play critical roles in transcriptional and post-transcriptional regulation^21^. Although many circRNAs have been discovered, only a few have been studied in detail. For example, circRNAs may down- or up-regulate the expression of their parent genes^13,22^. Additionally, circRNAs act as miRNA sponges, which can absorb the miRNA^23^. Consequently, circRNA can not only regulate the expression of its target coding genes, but also stabilize its cognate miRNA through binding. The loss of a mammalian circRNA locus causes miRNA deregulation and affects the brain function^20^.

*Ganoderma lucidum* is an economically important medicinal fungi and can enhance the immune system, promote anticancer activities, and reduce stress^24^. The polysaccharides of *G. lucidum* extracts can induce weight loss through the regulation of intestinal microbiome^25^. Elucidating the genetic basis for the synthesis of secondary metabolites is an active area of research. Previously, we sequenced the complete genome of *G. lucidum*^26^. According to the genome, we systematically analyzed its coding genes, such as the cytochrome P450 (*CYP450*) genes responsible for the production of secondary metabolites, genes encoding carbohydrate-active enzymes (*CAZy*), lignin lytic oxidoreductases genes involved in wood degradation, lncRNA genes^27^, and natural antisense transcripts (NATs)^28^ that are potentially involved in the expression regulation of genes in the cellular process. Hence, *G. lucidum* has become a model species in studying the biology of basidiomycetes. Taken together, these data provide basis for an in-depth study regarding the biology of *G. lucidum*. Nevertheless, circRNAs have not been investigated in *G. lucidum* to date.

In the present study, we performed a transcriptome-wide identification of circRNAs in *G. lucidum* using strand-specific RNA-seq data. The present results provide an overview of the circRNAs in *G. lucidum* and reveal substantial evidence that circRNAs are possibly involved in the expression regulation of genes participating in a wide range of biological processes.

## Results

### Computational identification of circRNAs in *G. lucidum*

CircRNAs were identified based on the presence of back-spliced reads as described previously^29^. To identify the circRNAs in *G. lucidum* systematically, RNA samples were extracted from the three developmental stages of *G. lucidum*, namely, mycelia, primordia, and fruiting bodies, with two biological replicates from each stage. Each set of samples was subjected to the construction of two types of libraries: polyA(−)/RNase R and polyA(−). Each library was sequenced using the HiSeq. 4000 platform. The numbers of reads obtained from the polyA(−)/RNase R libraries are shown in Table S1A. The total numbers of sequencing reads were in the range of 21.6–37.5 million. These clean reads were used to predict circRNAs by using CIRCexplorer2 as described previously^29^; this strategy combines two computation modules, namely, TopHat^30^ and TopHat-Fusion^31^, to obtain back-spliced junction reads for circRNA prediction. The rate of TopHat mapping (splicing reads) ranged from 54.3% to 63.6%. The rate of TopHat-Fusion (back-spliced reads) ranged from 0.79% to 1.18% over clean reads. The numbers of reads obtained from the polyA(−) libraries are presented in Table S1B. The total numbers of reads were in the range of 31.0–37.6 million. The rate of TopHat mapping (splicing reads) ranged from 40.7% to 65.8%. The rate of TopHat-Fusion (back-spliced reads) ranged from 0.51% to 0.99% over clean reads. Evidently, many back-spliced reads were obtained from the polyA(−)/RNase R libraries.

Steps in analyzing the circRNAs are shown in Fig. S1. In step 1, we predicted circRNAs using the read count cutoff of 1 in each of the six samples across the three stages, with two biological replicates for each stage. In step 2, the predicted circRNAs were divided into exonic and intronic groups based on the location of the junction reads. In step 3, the exonic and intronic circRNAs found in both biological replicates (the intersection set) were selected. In step 4, these shared circRNAs from different stages were compared. In step 5, to identify the circRNAs with relatively high expression levels, we combined the exonic circRNAs predicted from all six samples and selected the circRNAs with read count ≥5 across the six samples. These sets of circRNAs were subjected to detailed characterization (step 6), functional enrichment analyses (step 7), differential gene expression analysis (step 8), and expression correlation analyses (step 9).

### Comparison of data generated from the two types of libraries for circRNA identification

**Figure 1:**
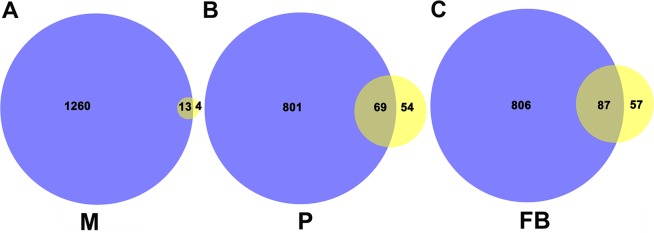
Comparison of circRNAs identified using two different library construction methods: polyA(−)/RNase R and polyA(−). Numbers of circRNAs identified in mycelia (**A**), primordia (**B**) and fruiting bodies (**C**). Purple: polyA(−)/RNase R; yellow: polyA(−).

First, we compared the total numbers of circRNAs identified among the three stages, namely, mycelia, primordia, and fruiting bodies, by using each of the two library construction methods. As shown in Fig. 1, the sequencing results of the polyA(−)/RNase R libraries indicated that 1273, 870, and 893 exonic and intronic circRNAs were identified in both replicate samples of mycelia, primordia, and fruiting bodies, respectively, by using the read cutoff of 1 in at least one sample (Table S2). With regard to the RNA-seq data generated from the polyA(−) libraries, 17, 123, and 144 exonic and intronic circRNAs were identified in both replicate samples from the three stages by using the read cutoff of 1 (Table S3). These results demonstrated that the polyA(−)/RNase R library produced significantly more circRNAs than that of the polyA(−) library. According to the results identified from polyA(−)/RNase R libraries, most circRNAs were identified in mycelia. By contrast, results from polyA(−) libraries indicated the least number of circRNAs was identified in mycelia. The numbers of circRNAs found in primordia and fruiting bodies were similar. The underlying reasons for these discrepancies remain unknown.

Afterward, we compared the identical circRNAs predicted from the two different library construction methods across the three stages. The circRNAs with exactly the same junction positions were considered the same. The numbers of identical circRNAs in the two libraries were 13, 69, and 87 in the samples of mycelia, primordia, and fruiting bodies, respectively (data not shown). These results were consistent with those reported previously that the polyA(−)/RNase R library significantly enriches more circRNAs than that of the polyA(−) library. Hence, in the following text, we focused our analyses on the circRNAs identified from the polyA(−)/RNase R library unless otherwise specified.

### Comparison of circRNAs identified between the two biological replicates

We also investigated the consistency of the circRNAs predicted between the two biological replicates in two approaches. For the first approach, all exonic and intronic circRNAs were considered. In total, 320 out of 1273 (25.1%, Fig. S2A), 200 out of 870 (23.0%, Fig. S2B), and 203 out of 893 (22.7%, Fig. S2C) circRNAs were shared between the two biological replicates in mycelia, primordia, and fruiting bodies, respectively.

With regard to the second approach, we compared the exonic and intronic circRNAs identified between the replicates from each of the three stages. A total of 1273 circRNAs in mycelia included 1102 exonic and 171 intronic circRNAs. A total of 870 circRNAs in primordia included 672 exonic and 198 intronic circRNAs. A total of 893 circRNAs in fruiting bodies included 733 exonic and 160 intronic circRNAs. As shown in Fig. S2, 283 out of 1102 (25.7%, Fig. S2D), 156 out of 672 (23.2%, Fig. S2E), and 170 out of 733 (23.2%, Fig. S2F) exonic circRNAs were found in both replicates in each of the three stages. As shown in Fig. S3, 37 out of 171 (21.6%, Fig. S2G), 44 out of 198 (22.2%, Fig. S2H), and 33 out of 160 (20.6%, Fig. S2I) intronic circRNAs were shared by the two biological replicates. These results showed the presence of significant variations (only 20%–26% consistency) among the circRNAs identified even between the biological replicates.

### Comparison of circRNAs identified among the three developmental stages

**Figure 2:**
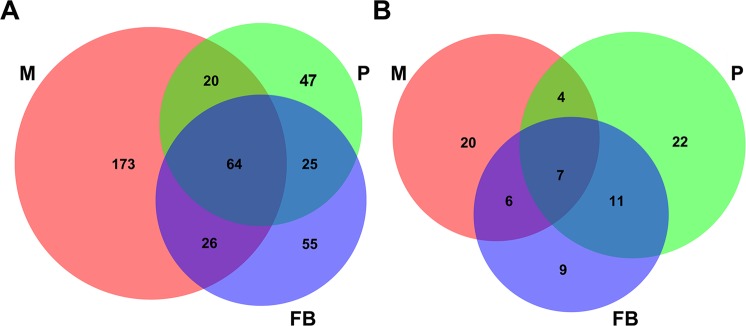
Comparison of circRNAs identified among the three stages. Exonic (**A**) and intronic circRNAs (**B**). M: mycelia; P: primordia; and FB: fruiting bodies.

As described, the circRNAs identified between the biological replicates showed low consistency. Consequently, we used the circRNAs identified in both replicates to determine the circRNA distribution among the three developmental stages. A total of 489 circRNAs, which included 410 exonic and 79 intronic circRNAs, were shared by the two replicates in all three stages. The distributions of the exonic and intronic circRNAs among the three stages are shown in Fig. 2A,B, respectively. A total of 173, 47, and 55 out of 410 (42.2%, 11.5%, and 13.4%) exonic circRNAs were found only in mycelia, primordia, and fruiting bodies, respectively. A total of 20, 22, and 9 out of 79 (25.3%, 27.8%, and 11.4%) intronic circRNAs were found only in mycelia, primordia, and fruiting bodies, respectively. In addition, 64 out of 410 exonic (15.6%) and 7 out of 79 intronic (8.9%) circRNAs were shared across all the three tissues. The genes to which the back-spliced reads were mapped were considered the parent genes of the circRNAs.

### Characterization of circRNAs in *G. lucidum*

Given that some circRNAs can be found in only one of the replicates in more than two stages, only considering the circRNAs found in two replicates as described above will exclude this group of circRNAs, thereby leading to a high false-negative rate. Consequently, we combined data from the three stages and used a cutoff of back-spliced junction reads ≥5 across the six samples as used previously to select the circRNAs for detailed characterization^17^. We only analyzed the exonic circRNAs in the following text because they represent the majority of circRNAs in *G. lucidum*. A total of 731 exonic circRNAs were identified by using this cutoff; 666, 324, and 351 of these circRNAs were expressed in mycelia, primordia, and fruiting bodies, respectively.

**Figure 3:**
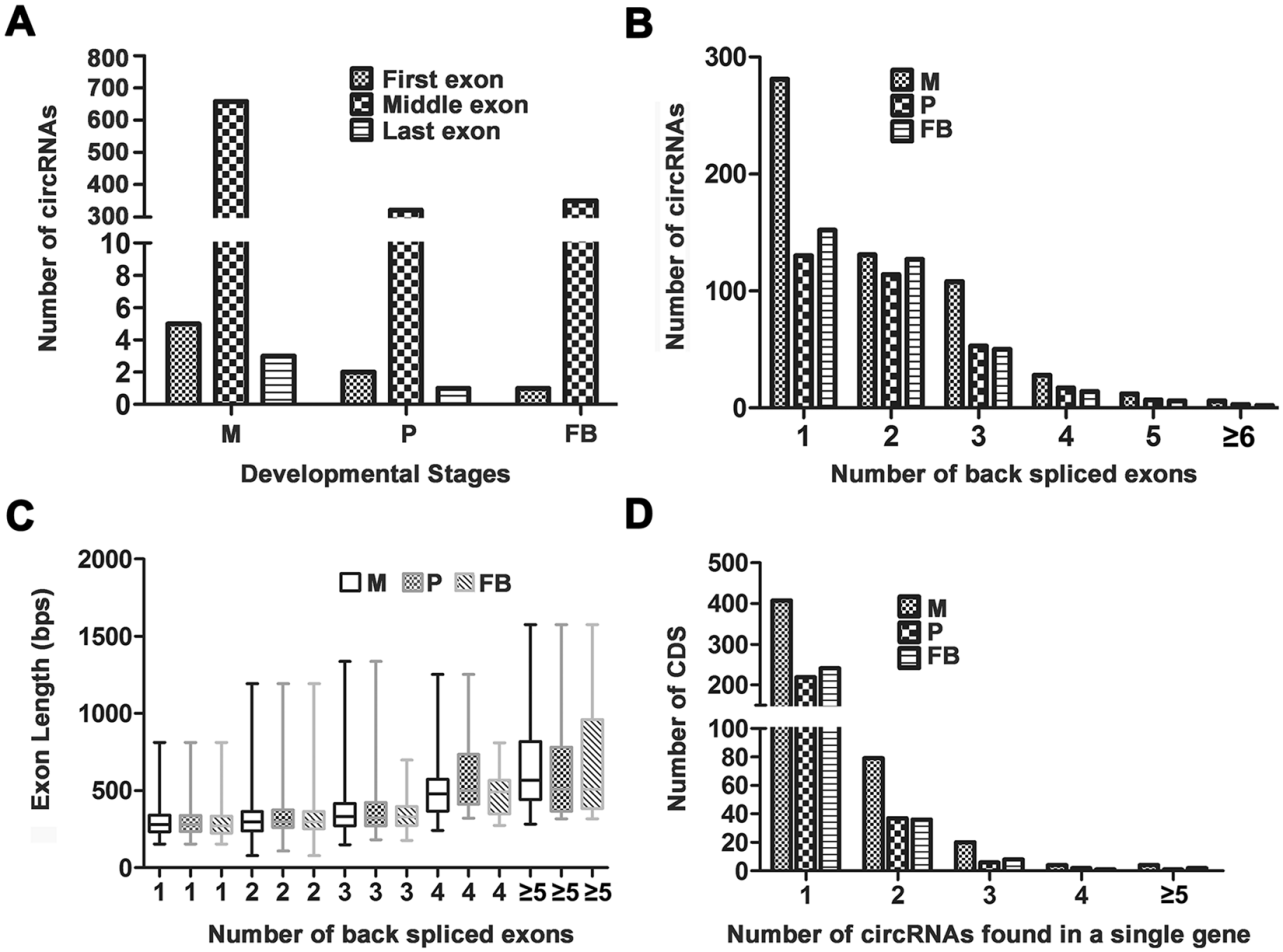
Genomic feature analyses of circRNAs in *G lucidum*. (**A**) Positions of the back-spliced exons of the circRNAs across three developmental stages. (**B**) Length distribution of back-spliced exons. The X-axis indicates the number of back-spliced exons, and the Y-axis indicates the exon length distribution of circRNAs identified across the three developmental stages. (**C**) Number of back-spliced exons distributed in circRNAs across three developmental stages. The X-axis indicates the number of back-spliced exons, and the Y-axis indicates the number of circRNAs. The lengths of the exons for the circRNAs that originated from a single exon were significantly shorter than those in other categories (*p < 2.2e-16, Wilcoxon rank sum test). (**D**) Relationship between the circRNAs and their parent genes. The X-axis indicates the number of back-spliced exons, and the Y-axis indicates the number of parent genes that can produce the corresponding numbers of circRNAs. M: mycelia; P: primordia; and FB: fruiting bodies.

Then, we analyzed the position of the circRNAs containing exons in their parent genes in to determine if the postions of exons have any effects on the generation of circRNAs. As illustrated in Fig 3A, 5, 658, and 3 out of 666 (0.75%, 98.8%, and 0.45%) circRNAs in mycelia originated from the first, middle and last exons, respectively. A total of 2, 321, and 1 out of 324 (0.61%, 99.1%, and 0.3%) circRNAs in primordia originated from the first, middle and last exons, respectively. A total of 1, 350, and 0 out of 351 (0.3%, 99.7%, and 0%) circRNAs in fruiting bodies originated from the first, middle and last exons, respectively. Evidently, most circRNAs (>98.8%) were mapped to the exons in the middle of the genes; only a considerably small portion of circRNAs was obtained from the first and the last exons. This finding strongly suggested that the biogenesis of circRNAs is associated with the splicing processes.

The junction reads can locate in the same back-spliced exon or in two different back-spliced exons (i.e., containing none or multiple exons). To determine whether circRNA biogenesis exhibits any preference for the numbers of exons, we compared the circRNAs in terms of the exons they are mapped to. As shown in Fig. 3B, 281 out of 666 (42.2%), 130 out of 324 (40.1%), and 152 out of 351 (43.3%) circRNAs originated from a single back-spliced exon in mycelia, primordia, and fruiting bodies, respectively, which are the major types of circRNAs. A total of 231 out of 666 (34.7%), 114 out of 324 (35.2%), and 127 out of 351 (35.6%) circRNAs originated from two different back-spliced exons in mycelia, primordia, and fruiting bodies, respectively. Finally, 154 out of 666 (23.1%), 80 out of 324 (24.7%), and 72 out of 351 (20.5%) circRNAs contained more than one exon in the corresponding stages, respectively. In summary, most circRNAs (>80%) originated from one or two back-spliced exons across the three stages.

To determine whether the formation of circRNAs exhibits any preference for the length of exon, we analyzed the effect of exon length on the formation of circRNAs. With regard to the circRNAs that originated from two different back-spliced exons, the lengths of all exonic sequences between the two junction positions were added for the analysis (Fig. 3C). In mycelia, the exon length ranged from 150 bps to 800 bps for the 281 circRNAs that originated from one exon. The lengths ranged from 150 bps to 900 bps for the 231 circRNAs that originated from two exons. In primordia, the exon lengths ranged from 154 bps to 813 bps for the 130 circRNAs that originated from one exon. The length ranged from 108 bps to 1192 bps for the 114 circRNAs that originated from two exons. In fruiting bodies, the exon lengths ranged from 154 bps to 813 bps for the 152 circRNAs that originated from one exon. The length ranged from 157 bps to 1192 bps for the 127 circRNAs that originated from two exons. Details are presented in Table S2. In general, the exon length increased with the increased number of exon from which the circRNAs originated.

We also analyzed the mapping relationship between circRNAs and their parent genes. A total of 561 parent genes were mapped to the 731 circRNAs. As shown in Fig. 3D, 407, 219, and 241 parent genes demonstrated 1:1 relationships with circRNAs in the samples from mycelia, primordia, and fruiting bodies, respectively. A total of 79, 37, and 36 parent genes showed 1:2 relationships with circRNAs across the three stages. Finally, 28, 8, and 1 parent genes demonstrated 1:n relationships with circRNAs across the three stages. Obviously, most parent genes produce only one circRNA.

**Figure 4:**
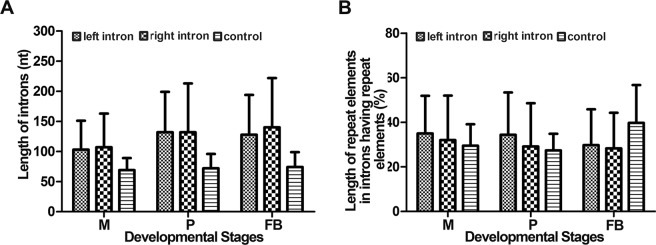
Comparison of the left, right, and control introns. (**A**) Length comparison. (**B**) Percentage of the length of repetitive sequences over that of the intron. M: mycelia; P: primordia; and FB: fruiting bodies.

Subsequently, we analyzed the lengths of flanking introns of circRNAs because they can affect the formation of circRNAs^13^. First, we selected the circRNAs that originated from two different back-spliced exons. The regions bracketing the circRNAs were defined as the linear genes. The introns within the linear genes were defined as the control introns. First, we compared the lengths of the left and right flanking introns and control introns. Results showed that the flanking introns of the circRNAs were much longer than those of the controls (Fig. 4A). These results were consistent with those reported previously in plants and animals^12^. Details for this comparison are shown in Table S4a. For example, the average lengths of the left and right flanking introns and the control introns were 150, 147, and 84 bps, respectively, in the mycelia. The average lengths of the left and right flanking introns and the control introns were 174, 178, and 91 bps long, respectively, in the primordia. The average lengths of the left and right flanking introns and control introns were 169, 176, and 94 bps, respectively, in fruiting bodies. Data showed that the lengths of the flanking introns were significantly longer than those of the control introns. These results were similar to those described previously.

A previous study showed that inverted repeats can lead to RNA circularization^17^. For example, 30–40 nt inverted repeats are sufficient for the circularization of RNA molecules. To determine whether repeats are associated with circRNAs in *G. lucidum*, we identified the repetitive elements in the flanking and control introns. Details are presented in Table S4b. Repeat elements were 149 out of 731 (20.4%) exonic circRNAs. Among them, 72, 67, and 19 out of 149 (48.3%, 45%, and 10.7%) circRNAs contained repeats in their left, right, and control introns, respectively (Fig. S3). More than 90% of all repeats were found either on the left or right introns. Only 9 out of 149 (6.0%) circRNAs contained repeats in two types of introns. None of the circRNAs contained repeats in all three types of associated introns. With regard to the types of repeats, a total of 178 repeats were identified, and 142 of them (79.8%) were simple repeats (Fig. 4B, Table S4C). Particularly, two pairs of inverted repeats were found in GaLu96scf26_559095_559469 and GaLu96scf25_426165_426869, which were 44 and 24 nt long, respectively (Table S5). The presence of inverted repetitive elements is related to the genesis of circRNAs^17^. Hence, whether these inverted repeats are involved in the biogenesis of the associated circRNAs should be determined.

### Validation of the authenticity of the predicted circRNAs

**Figure 5:**
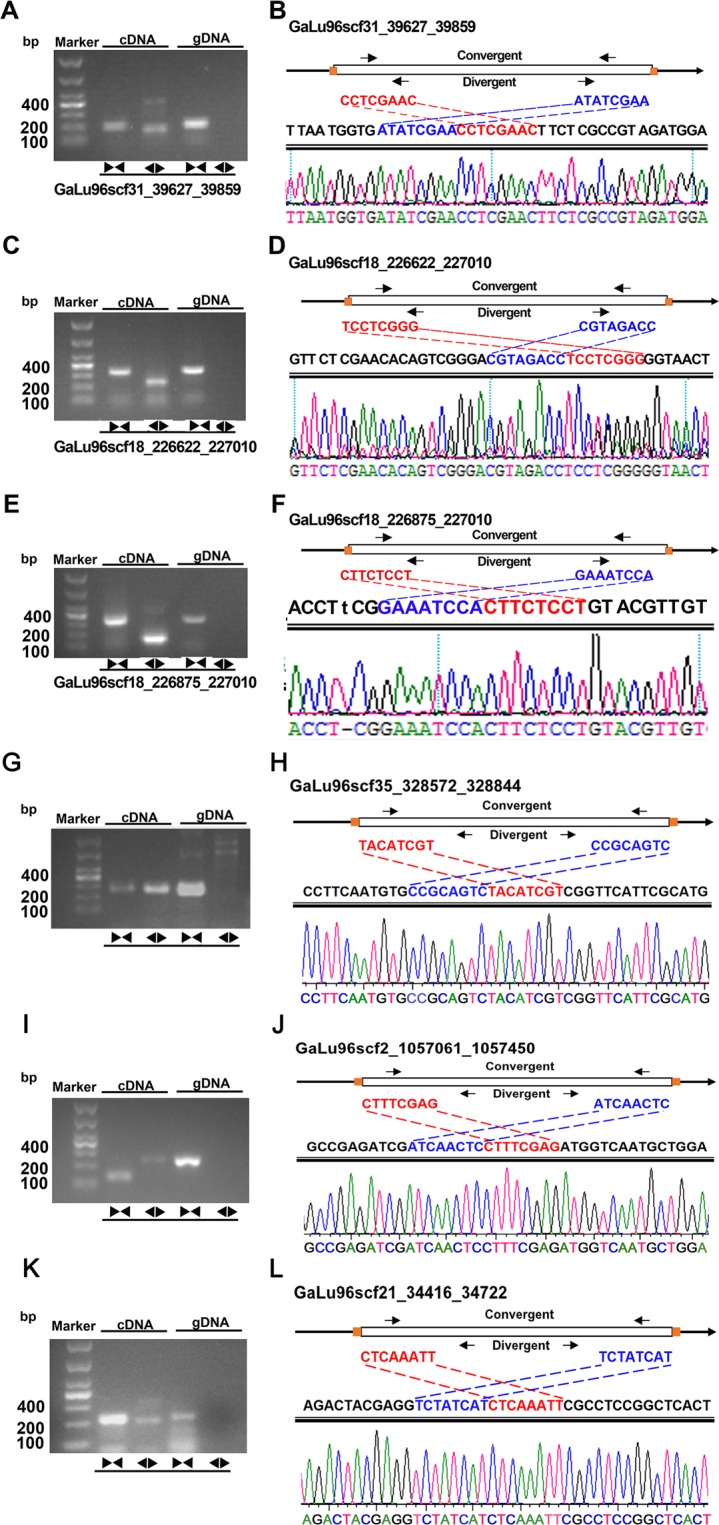
Validation of selected circRNAs. PCR experiments were conducted with convergent (represented by the pair of arrows facing inward) and divergent (represented by the pair of arrow heads facing outward) primers for the amplification of the cDNA and gDNA (the gel electrophoresis panels). The names of the circRNAs were shown below the gels. The PCR products were excised and subjected to Sanger sequencing. The corresponding results and their mapping to the genome are shown in the chromatogram panels. In each panel, the exon and relative locations of the corresponding convergent and divergent primers are shown at the top. The sequences around the junctions are shown in red and blue respectively.

To confirm the authenticity of these circRNAs, 20 circRNAs predicted by CIRCexplorer2^29^ with high expression level were selected randomly for experimental validation by using RT-PCR amplification and Sanger sequencing. The primers used for validation are listed in Table S6. For authentic circRNAs, convergent and divergent primers can generate products from RNase R-treated RNAs. Only the corresponding convergent primers can generate products from the genomic DNAs, whereas the divergent primers cannot generate any product from the genomic DNAs. In addition, the correctness of the back-splicing sites of these amplified products was further verified using Sanger sequence. Only 3 circRNAs were amplified (Fig. 5A,C,E, Table S6). Sanger sequencing revealed that the sequence of the three circRNAs were the same as those expected (Fig. 5B,D,F). Among the validated circRNAs, two circRNAs originated from the same parent gene (Fig. 5C,E). In order to identify the reliability of more circRNAs, the circRNAs were furtherly predicted by other two softwares, circRNA_finder^32^ and CIRI2^33^. The predicted results were shown in Supplementary files 1 and 2. Another 111 circRNAs predicted were validated, which were selected from the intersection of the results predicted by the three softwares. However, only 7 circRNAs (Table S6) were amplified using convergent and divergent primers simultaneously. All of them were sequenced but only three (Fig. 5G,I,K) of them have the sequences consistent with those expected (Fig. 5H,J,L). The gels showing PCR amplification products for validating candidate circRNAs were displayed in Fig. S4.

### Functional enrichment analysis of the parent genes of circRNAs across the three developmental stages

Given that circRNAs possibly function through the regulation of the expression of their parent genes, the biological functions of circRNAs may be identified through the functional analyses of their parent genes^13,22^. To determine the potential role of circRNAs, those with back-spliced read counts ≥5 were selected for further analysis, as described in our previous paper^28^. First, we carried out functional enrichment analysis of parent genes of circRNAs by GO terms and KEGG pathways in different developmental stages (Tables S7 and S8). Among the 561 parent genes, 380 of them were mapped to 335 GO terms. Among these GO terms, the q-values for the enrichment of 37 GO terms were less than 0.01. Among them, 22 GO terms displayed more than 10 parent genes mapped to them (Table 1). A similar enrichment analysis was conducted for the KEGG pathways. Among the 561 parent genes, 192 of them were mapped to 208 KEGG pathways. The q-values for the enrichment of 35 KEGG pathways were less than 0.01. A total of 11 KEGG pathways were found; each pathway contained more than 10 genes mapped to them (Table 1).

**Table 1:** Significantly enriched GO terms and KEGG pathways for the parent genes of exonic circular RNAs (circRNAs) expressed in at least one of the developmental stages.

Category ID	Annotation	No. of genes in the genome	No. of parent genes in the genome	q-value (FDR)
GO:0003824	Catalytic activity	791	58	<0.0001
GO:0055114	Oxidation reduction	618	50	<0.0001
GO:0008152	Metabolic process	683	41	0.0033
GO:0005622	Intracellular	381	36	<0.0001
GO:0016491	Oxidoreductase activity	482	34	0.0011
GO:0016020	Membrane	312	32	<0.0001
GO:0005488	Binding	476	31	0.0041
GO:0055085	Transmembrane transport	340	30	0.0001
GO:0016021	Integral to membrane	251	21	0.0023
GO:0020037	Heme binding	257	21	0.0028
GO:0005506	Iron ion binding	260	21	0.0031
GO:0006412	Translation	149	20	<0.0001
GO:0000166	Nucleotide binding	214	20	0.0011
GO:0009055	Electron carrier activity	250	20	0.0041
GO:0004497	Monooxygenase activity	258	19	0.0095
GO:0006810	Transport	174	17	0.0019
GO:0005525	GTP binding	97	16	<0.0001
GO:0003735	Structural constituent of ribosome	107	16	<0.0001
GO:0005840	Ribosome	105	15	0.0001
GO:0005737	Cytoplasm	138	14	0.0034
GO:0015031	Protein transport	57	10	0.0006
GO:0006350	Transcription	76	10	0.0035
ko01100	Metabolic pathways	675	90	<0.0001
ko01130	Biosynthesis of antibiotics	193	22	<0.0001
ko01120	Microbial metabolism in diverse environments	187	21	<0.0001
ko01200	Carbon metabolism	91	13	<0.0001
ko01212	Fatty acid metabolism	21	12	<0.0001
ko05016	Huntington’s disease	73	12	0.0001
ko03010	Ribosome	69	12	0.0001
ko00190	Oxidative phosphorylation	64	11	0.0002
ko01220	Degradation of aromatic compounds	31	10	0.0000
ko05010	Alzheimer’s disease	54	10	0.0001

To determine if circRNAs can function in a development specific manner, we performed functional enrichment analysis in each of the three developmental stages (Table S9). A total of 40, 13, and 4 GO terms were enriched in mycelia, primordia, and fruiting bodies, respectively, with q-value < 0.01. Additionally, 22, 20, and 23 GO terms were enriched in the three stages with 0.01 < q-value < 0.05. The most significantly enriched GO terms in each of the three stages are shown in Table 2. Analysis results for the KEGG pathways are shown in Table S10. A total of 28, 17, and 6 pathways were significantly enriched with *q*-value < 0.01 in the mycelia, primordia, and fruiting bodies, respectively. Furthermore, 33, 22, and 21 pathways were enriched in the three stages with 0.01 < q-value < 0.05. The most significantly enriched KEGG pathways in each of the three stages are presented in Table 2.

**Table 2:** Significantly enriched GO terms and KEGG pathways for the parent genes of exonic circRNAs expressed in particular developmental stages.

ID	Annotation	Mycelia	Primordia	Fruiting bodies
GO:0005622	Intracellular	q < 0.01	q < 0.01	q < 0.01
GO:0016020	Membrane	q < 0.01	q < 0.01	q < 0.01
GO:0003824	Catalytic activity	q < 0.01	q < 0.01	0.01 < q < 0.05
GO:0055114	Oxidation reduction	q < 0.01	0.01 < q < 0.05	q < 0.01
GO:0016616	Oxidoreductase activity, acting on the CH-OH group of donors, NAD, or NADP as acceptor	q < 0.01	0.01 < q < 0.05	0.01 < q < 0.05
GO:0007165	Signal transduction	q < 0.01	q < 0.01	^a^N
GO:0008483	Transaminase activity	q < 0.01	q < 0.01	N
GO:0005525	GTP binding	q < 0.01	q < 0.01	N
GO:0006457	Protein folding	q < 0.01	N	0.01 < q < 0.05
GO:0009055	Electron carrier activity	q < 0.01	N	0.01 < q < 0.05
GO:0020037	Heme binding	q < 0.01	N	0.01 < q < 0.05
GO:0004497	Monooxygenase activity	q < 0.01	N	0.01 < q < 0.05
GO:0005506	Iron ion binding	q < 0.01	N	0.01 < q < 0.05
GO:0006355	Regulation of transcription, DNA dependent	N	0.01 < q < 0.05	0.01 < q < 0.05
GO:0008152	Metabolic process	q < 0.01	N	N
GO:0016021	Integral to membrane	q < 0.01	N	N
GO:0016491	Oxidoreductase activity	q < 0.01	N	N
GO:0046872	Metal ion binding	q < 0.01	N	N
GO:0005488	Binding	q < 0.01	N	N
ko01100	Metabolic pathways	q < 0.01	q < 0.01	q < 0.01
ko00330	Arginine and proline metabolism	q < 0.01	q < 0.01	q < 0.01
ko01110	Biosynthesis of secondary metabolites	q < 0.01	q < 0.01	0.01 < q < 0.05
ko01120	Microbial metabolism in diverse environments	q < 0.01	q < 0.01	0.01 < q < 0.05
ko01130	Biosynthesis of antibiotics	q < 0.01	q < 0.01	0.01 < q < 0.05
ko04120	Ubiquitin-mediated proteolysis	0.01 < q < 0.05	q < 0.01	0.01 < q < 0.05
ko00620	Pyruvate metabolism	q < 0.01	0.01 < q < 0.05	N
ko01200	Carbon metabolism	q < 0.01	0.01 < q < 0.05	N
ko01230	Biosynthesis of amino acids	q < 0.01	0.01 < q < 0.05	N
ko04144	Endocytosis	0.01 < q < 0.05	0.01 < q < 0.05	N
ko00270	Cysteine and methionine metabolism	0.01 < q < 0.05	N	N
ko01524	Platinum drug resistance	0.01 < q < 0.05	N	N
ko03430	Mismatch repair	0.01 < q < 0.05	N	N

^a^N: not statistically significant

### Potential role of circRNAs in transcriptional regulation

Transcriptional factors (TFs) function in a wide range of biological processes^34^. To determine whether the circRNAs are related to a TF factor, we compared the protein sequences of 561 parent genes of the circRNAs against those in FFTD with default parameters^35^. A total of 157 parent genes (28.0%) were annotated as TFs according to the annotations of the best hits from FFTD (Table S11). The expression profiles of 68 circRNAs and their 56 parent genes were positively correlated (*r* ≥ 0.9, q < 0.01). By contrast, the expression profiles of 38 circRNAs and their parent genes were negatively correlated (*r* ≤ −0.9, *q* < 0.01). The precise mechanisms remain to be elucidated in the future.

### Differential expression of exonic circRNAs across the three developmental stages

Differential expression can indicate the stage-specific roles of circRNAs. We investigated whether the circRNAs were significantly differentially expressed across the three stages by using t-test. In total, 200 circRNAs, including 177 exonic and 23 intronic circRNAs, were significantly differentially expressed (*q* < 0.05) across the three developmental stages (Table S12). A total of 151, 18, and 8 of 177 (85.3%, 10.2% and 4.5%) exonic circRNAs had the highest expression in the mycelia, primordia, and fruiting bodies, respectively. A total of 53 parent genes are potentially involved in the transcriptional regulation, polysaccharide biosynthesis and etc (Table S12).

### Correlation of the expression profiles of circRNAs and their parent genes

Next, we determined the correlation between the expression profiles of circRNAs and those of their parent genes. The expression profiles of 119 of 731 (16.3%) exonic circRNAs and their parent genes were significantly positively correlated (*r* ≥ 0.9, *q* < 0.01), whereas those of 226 of 731 (30.9%) were significantly negatively correlated (*r* ≤ −0.9, *q* < 0.01) (Table S13).

**Figure 6:**
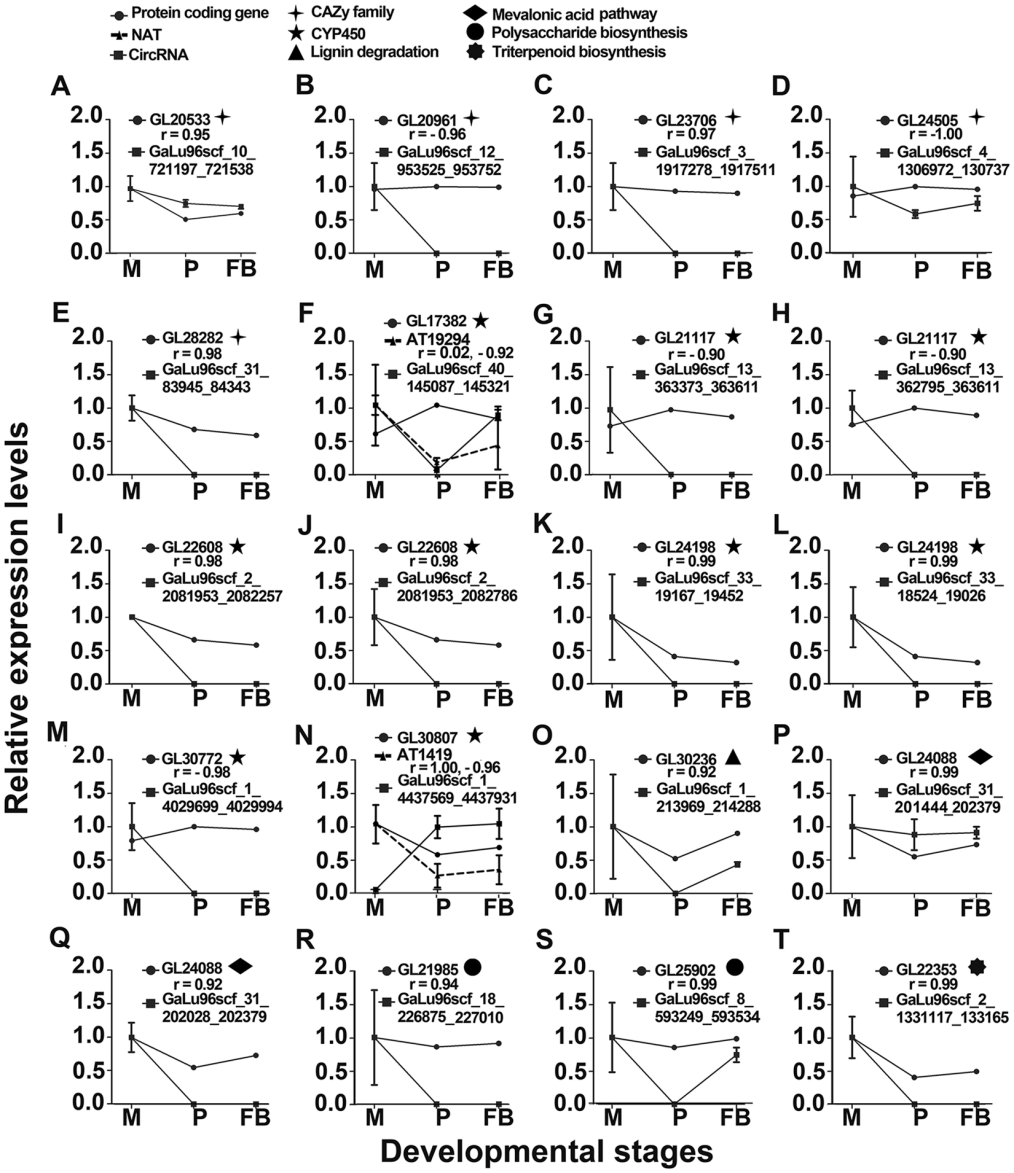
Correlation of the expression profiles of the parent genes and circRNAs or NATs across three developmental stages. The expression profiles among the 20 pairs of parent genes and their circRNAs or natural antisense transcripts (NATs) are shown. The functional categories of the corresponding genes are shown on the top. For each panel, the X-axis shows the developmental stages. The Y-axis shows the relative expression levels of the protein-coding genes (GL), circRNAs (GaLu), and NAT (AT). The error bar represents variations between two biological replicates. M: mycelia; P: primordia; FB: fruiting bodies; ✦: CAZy family; ▲: CYP450; ◆: lignin degradation; ●: mevalonic acid; ✺: polysaccharide biosynthesis; ★: triterpenoid biosynthesis.

To determine whether any expression correlation existed between noncoding RNAs, such as circRNAs, NATs and miRNAs and their parent genes, the expression profiles of 20 circRNAs and their parent genes were analyzed (Fig. 6A–T). Group one included five parent genes belonging to the CAZy family. The expression profiles of three of them positively correlated with those of their circRNAs; the two other genes negatively correlated with those of their circRNAs (Fig. 6A–E).

Group two included nine circRNA-gene pairs, which corresponded to six parent genes that belonged to the *CYP*450 family (Fig. 6F–N); three of them, namely, GL2117 (Fig. 6G,H), GL22608 (Fig. 6I,J), and GL24198 (Fig. 6K,L), produced two circRNAs each. Four of these nine pairs exhibited positive correlation (Fig. 6I–L), and the other five exhibited negative correlation (Fig. 6F–H, M,N). The expression of GL17382 and GL30807 was correlated with their NATs, consistent with those reported previously^16^. Particularly, GL17382 exhibited a slightly positive correlation with its NAT transcript AT19294, whereas GL30807 exhibited a significantly positive correlation with AT1419.

The third group included one positively correlated circRNA-gene pair related to lignin degradation (Fig. 6O). The fourth group included one parent gene related to the MVA pathway. This gene contained two alternative circRNAs, which were both positively correlated with their parent genes (Fig. 6P,Q). The fifth group included two positively correlated circRNA-gene pairs (Fig. 6R,S). Their parent genes were involved in polysaccharide biosynthesis. The final group included one positively correlated circRNA-gene pair (Fig. 6T), which was involved in the triterpenoid acid pathway.

The high-degree correlation and the diverse correlation pattern between the circRNAs and their parent genes indicated that circRNAs may be involved in the expression regulation of their parent genes in a complicated manner. The multiple relationships among the parent genes, their circRNAs, and NAT transcripts suggested that noncoding RNAs play important roles in the biology of *G. lucidum* (Table S14).

### Alternative circularization of exonic circRNAs in *G. lucidum*

One parent gene can produce one or more circRNAs. We identified 226 alternative backsplicing circularization events produced from 92 distinct parent gene loci (Table S2). A total of 70 of the 92 genes each produced two circRNA isoforms, 14 genes each produced three distinct circRNA isoforms, and 8 genes each produced at least four distinct circRNA isoforms.

**Figure 7:**
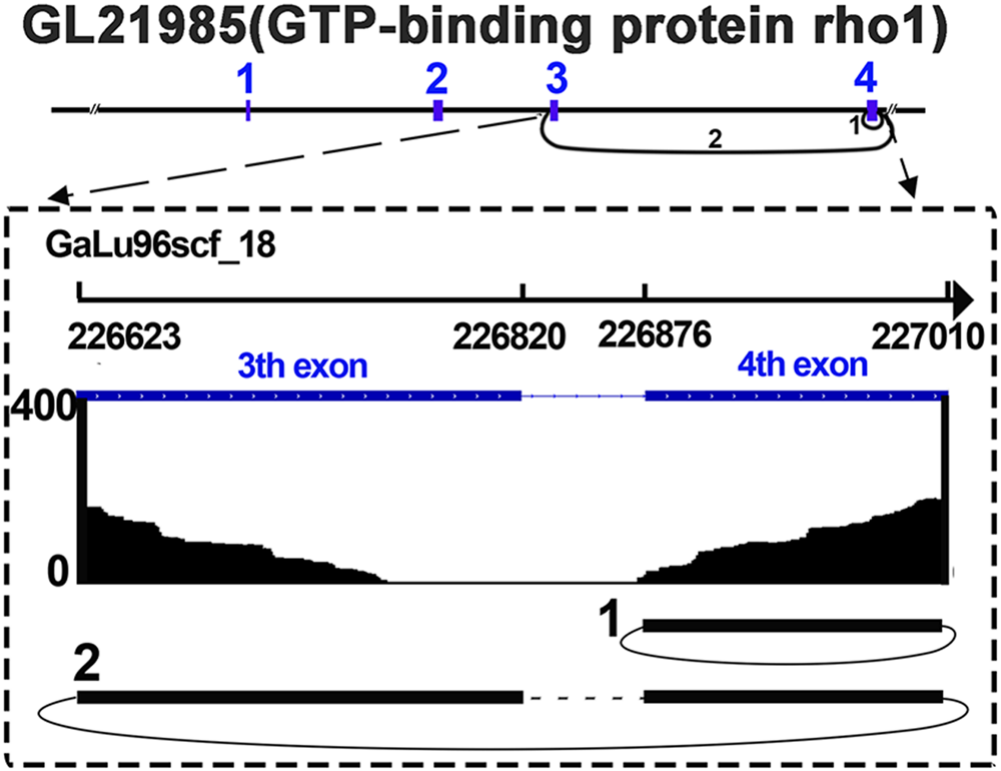
Visualization of alternative circularization events. The X-axis indicates the chromosomal region by using the Scaffold number and coordinates. The Y-axis indicates the numbers of junction reads. The back-spliced exons of circRNAs are shown in blue. The numbers of circRNAs are shown at the bottom of each panel.

One circularization event was analyzed in detail. GaLu96scf_18_226875_227010 and GaLu96scf_18_226622_227010 originated from GL21985 (Fig. 7), which was verified by RT-PCR (Fig. 5C,E). The expression level of GaLu96scf_18_226875_227010 was also significantly positively correlated with that of its parent gene GL21985 (Fig. 6R).

## Discussion

### Discovery of circRNAs in *G. lucidum*

To date, many circRNAs have been identified in animals, plants, and fungi^8–16,36^. However, no circRNAs have been reported in the *G.lucidum* to our knowledge. In the present study, we identified 250 and 2193 circRNAs in *G. lucidum* using next-generation DNA sequencing technologies from polyA(−) and polyA(−)/RNase R-treated RNA-seq libraries, respectively. High-degree variations between replicates were observed in the data generated from these libraries because circRNAs were expressed at considerably low levels, or their expressions were tissue-, temporally, and/or spatially specific.

Further analysis on circRNAs from the RNase R-treated library revealed many specific features of circRNAs from *G. lucidum*. For example, the length of the flanking introns of the circRNAs was much longer than that of the introns of linear genes. The percentage of repetitive elements in the flanking introns of *G. lucidum* is less than those in animals but much more than those in plants^12^. The short inverted repeats in the flanking introns of *G. lucidum* circRNAs are less than those in animals and plants^10,12^. The majority of circRNAs identified in *G. lucidum* originated from exons located in the middle of its parent gene. Therefore, circRNA formation is coupled with RNA splicing, which is similar to that described in animals^29^. The exon lengths of the circRNAs with only one back-spliced exon were much shorter than those in circRNAs with multiple back-spliced exons. This result was inconsistent with those described in animals^29^.

This study also found that multiple circRNAs originated from exons located in the same locus, which is referred to as alternative circularization. All these results indicated that the biogenesis of *G. lucidum* circRNAs is considerably complicated.

### Analyses of high false positive rate of circRNAs in *G. lucidum*

In this study, we first selected the circRNAs predicted by CIRCexplorer2^29^ with high expression level. However, only 3 out of 20 predicted circRNAs were amplified with expected sequences. Next, we selected 111 circRNAs from the intersection of three software predicted results and validated them by using RT-PCR and Sanger sequencing methods. Unfortunately, only another 3 circRNAs were validated with expected sequences. The reasons might be: (1) the softwares used in this study might not be suitable for predicting circRNAs in fungi; (2) the predicted circRNAs might be unreal; (3) the experimental methods were not suitable for validating the circRNAs of fungi. Correspondingly, more robust softwares were needed to predict the circRNAs in fungi. For experimental validation, more advanced methods were employed for validation of circRNAs.

### Potential functions of circRNAs

CircRNAs have been proposed to play important roles in diverse biological processes, such as cancer-related pathways^21^, Pi starvation^12^, dehydration stress^14^, and nitrogen stress^9^. CircRNAs function in three different manners. First, circRNAs may serve as miRNA sponges to inhibit miRNA function^23^. For example, circRNA CDR1 binds to miR-7 in neuronal tissues^8^. Second, circRNAs may regulate alternative splicing or transcription. General splicing factors, such as MBL, may affect the alternative splicing that modulates the balance between circRNA biogenesis and canonical splicing^19^. Jeck *et al*. discovered that many single-exon circRNAs contain a translation start site in human fibroblasts^37^; this result indicated that circRNAs can act as mRNA traps by sequestering the translation start site to regulate protein expression. Finally, circRNAs regulate the expression of their parent genes^13,22^. For example, circEIF3J and circPAIP2 up-regulate the expression of their parent genes in human cells^22^. Os08circ16564 down-regulates the expression of its parent genes in rice^13^.

This study represented the comprehensive analysis on circRNAs in *G. lucidum*as. During the preparation of our paper, luo *et al*.^37^ identified and characterized the the circRNAs of *Cryptococcus neoformans*, which was the first report of circRNAs in basidiomycetous. In this study, results suggested that circRNAs may regulate the expression of their parent genes across the three developmental stages or in developmental-stage-specific manners. The potentially regulated genes have been involved in the cellular growth, development, and secondary metabolism of *G. lucidum*. Several examples are given in the succeeding section. Parent gene GL24088 encodes a glutaryl-CoA synthase. Parent genes GL20553 and GL24505 encode members of the *CAZy* families. These parent genes were mapped to the following GO terms: catalytic activity (GO:0003824), membrane (GO:001620), oxidation reduction (GO:0055114), and oxidoreduction activity acting on the CH–OH group of donors NAD, or NADP as acceptor (GO:0016616); these terms were enriched in all three stages. Parent gene GL24088 encodes a glutaryl-CoA synthase; this gene was mapped to the GO term integral to membrane (GO:0016021), which was enriched only in mycelia. Parent genes GL17382, GL30772, and GL30807 belong to the CYP450 families. They were mapped to electron carrier activity (GO:0009055), heme binding (GO:0020037), and monooxygenase activity (GO:0004497), which were enriched in mycelia and fruiting bodies.

3-Hydro-3-methyl glutaryl coenzyme A (HMG-CoA) reductase is the first and the most important rate-limiting enzyme in the MVA pathway, which produce the precursors involved in triterpene synthesis, the main active components of *G. lucidum*. Glutaryl-CoA synthase is another important enzyme for the synthesis of HMG-CoA. Genes belonging to the CAZy families are potentially involved in polysaccharide synthesis^26^. CYP450 family genes are potentially involved in triterpenoid synthesis through a series of oxidation reactions^26^. The identification of circRNAs targeting these genes suggested the potential involvement of circRNAs in the biosynthesis of active components of *G. lucidum*.

This study also demonstrated that the expression profiles of circRNAs and their parent genes were highly positively (16.3%) or negatively (30.9%) correlated (Table S10). These observations were consistent with those previously reported in plants^13,22^ but different from those reported in animals^10^. Notably, some parent genes can generate multiple circRNA isoforms, which can exhibit expression profiles that are either positively or negatively correlated with those of the parent genes. This finding suggested the additional layers of interactions among circRNA isoforms and their parent genes in terms of gene expression regulation. The phenomenon was similar to that reported in two miRNA precursors identified in a single unigene^38,39^. All these data showed that circRNAs are potentially involved in gene expression regulation in *G. lucidu*m. Significantly less amount of knowledge is available regarding the mechanism of biogenesis and functional roles of circRNAs than those of mRNA, miRNA, and lncRNA.

## Materials and Methods

### Materials and data availability

A dikaryotic strain of *G. lucidum* CGMCC5.0026 was cultured as described previously^26^. The complete genome sequence of *G. lucidum* has been deposited at GenBank with the accession number PRJNA71455. The Illumina RNA-seq reads have been deposited in the short-read archive at GenBank under the project ID of PRJNA559154.

### RNA extraction and RNase R treatment, DNA extraction

Total RNA was extracted from three developmental stages of *G. lucidum*: mycelia, primordia, and fruiting bodies using Trizol reagent following the protocol recommended by the manufacturers. To prepare the RNase R-treated total RNA samples, the purified DNase I-treated total RNA was incubated for 15 min at 37 °C with 3 units/μl of RNase R. Then, the RNA was purified by phenol–chloroform extraction and re-precipitated in three volumes of ethanol. DNA was extracted by plant genome DNA kit using the recommend protocol.

### ssRNA-seq library construction and sequencing

To obtain circRNAs, two ssRNA-seq libraries were constructed: polyA(−) and polyA(−)/RNase R. The ssRNA-seq library was constructed following the manufacturer’s recommendation. High-throughput sequencing was performed using Illumina Hiseq4000. Each experiment was performed with two biological replicates.

### CircRNA identification

We systematically identified the circRNAs of mycelia, primordia, and fruiting bodies using CIRCexplorer2^29^, circRNA_finder^32^ and CIRI2^33^ with the default parameters. Briefly, to obtain back-spliced junction reads for circRNA prediction, a two-step mapping strategy was used. First, clean sequencing reads were mapped to the *G. lucidum* genome using TopHat (v2.0.9)^30^ with the default parameter. Afterward, the unmapped reads were mapped to the reference genome using TopHat-Fusion^31^ with default parameters. The abundance of the circRNAs was estimated using the count per million reads for the back-spliced junction reads.

### Statistical analysis

The significance of Pearson’s correlation coefficient was tested using t test as described previously^28^. Briefly, we calculated the t-value of Pearson’s correlation coefficient using Eq. (1)

1 $${\rm{t}}=\frac{r}{sqrt[(1-{r}^{2})/(N-2)]}$$

where *r* indicates the value of Pearson’s correlation coefficient, and N indicates the sample size.

### Functional enrichment analysis of parent genes

Functional enrichment analysis of *G. lucidum* genes were conducted as described previously^28^. Hyper geometric probability was calculated for each GO term using Eq. (2)

2 $$P({\rm{x}}={\rm{i}})=\frac{(\frac{n}{i})(\frac{m}{N-i})}{(\frac{m+n}{N})}=\frac{m!n!N!(m+n-N)!}{i!(n-i)!(m+i-N)!(N-i)!(m+n)!}\cdot $$

where “P” is the hypergeometric probability. “n” is the number of all genes in *G. lucidum* that are associated with the GO term. “m” is the number of all genes in *G. lucidum* that are not associated with the GO term. “N” is the number of all STs. And “x” is number of STs that are associated with the GO term.

### Validation of the authenticity of circRNAs by using PCR and sanger sequencing

Divergent and convergent primers were designed for circRNA validation. Total RNAs were extracted and treated by RNase R as described previously to construct the corresponding libraries. The cDNA samples were retrotranscribed from the total RNA treated with DNase I and RNase R and the genomic DNAs were used as control. Convergent primers were used as positive controls for linear transcripts, and divergent primers were used to confirm the presence of circular templates. Approximately 20 ng of cDNA or genomic DNA was used with Taq DNA polymerase and 10 × buffer (Takara, Dalian, China) for each PCR amplification, which was performed under the following conditions: 95 °C for 3 min; 35 cycles of 94 °C for 60 s; 55 °C for 30 s; and 72 °C for 30 s. The PCR products were subjected to gel electrophoresis analysis. The bands with size similar to the expected ones were dissected and purified using the AXYGEN Gel Extraction Kit (Qiagen, CA, USA). Sequencing was performed on an ABI3730 sequencer according to the manufacturer’s protocol.

## Supplementary information

Dataset 1

Dataset 4

Dataset 5

Dataset 2

Dataset 3
