# Supplementary material for: Identification and characterization of circular RNAs in Ganoderma lucidum
Source: Sci Rep. 2019 Nov 11;9:16522. doi: 10.1038/s41598-019-52932-w (PMC6848116; doi:10.1038/s41598-019-52932-w)
Supplement: Supplementary file 1 — Dataset 1 [file 41598_2019_52932_MOESM1_ESM.pdf]

Identification and characterization of circular RNAs in *Ganoderma lucidum*

Junjie Shao[#1], Liqiang Wang[#1], Xinyue Liu[2], Meng Yang[1], Haimei Chen[1], Bin Wu[$1], Chang Liu[$1]

[1]Institute of Medicinal Plant Development, Chinese Academy of Medical Sciences & Peking Union Medical College, No. 151 Malianwa North Road, Haidian District, Beijing 100193, P. R. China.
[2]School of Chinese Materia Medica, Beijing University of Chinese Medicine, Beijing 100029, P. R. China

[#]Those authors contributed equally to this work.
[$]Corresponding author: Bin Wu, E-mail: bwu@implad.ac.cn, Phone:+86-10-57833201 and Chang Liu, cliu6688@yahoo.com, Phone:+86-10-57833111

JJS: shaojie415@126.com
LQW: lys832000@163.com
XYL: lxy_medicine@163.com
MY:347988352@qq.com
HMC: hmchen@implad.ac.cn
BW: bwu@implad.ac.cn
CL: cliu6688@yahoo.com

Figure S1 Flow chart for the circRNAs prediction and analysis.

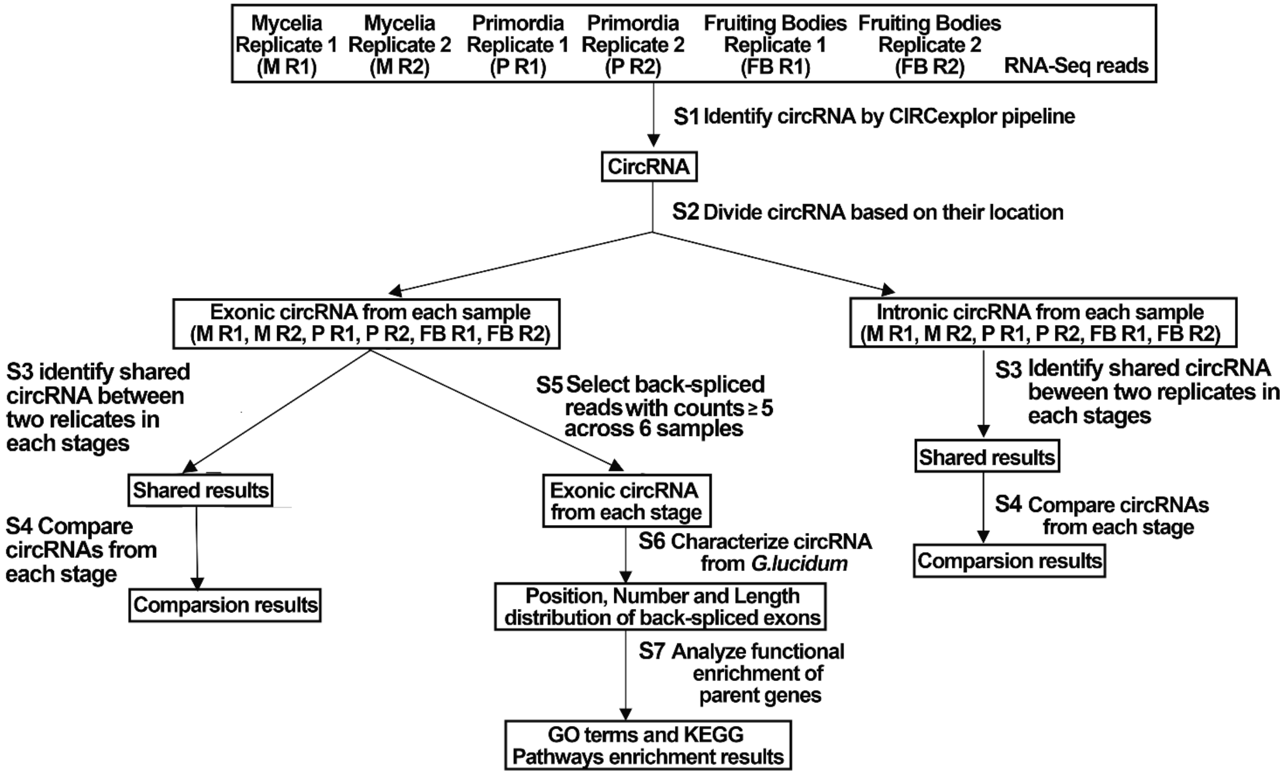

Figure S2 Comparison of circRNAs identified between replicates across the three developmental stages of *G. lucidum*. (A) All exonic and intronic circRNAs; (B) Exonic circRNAs only; (C) Intronic circRNAs only.

(A)

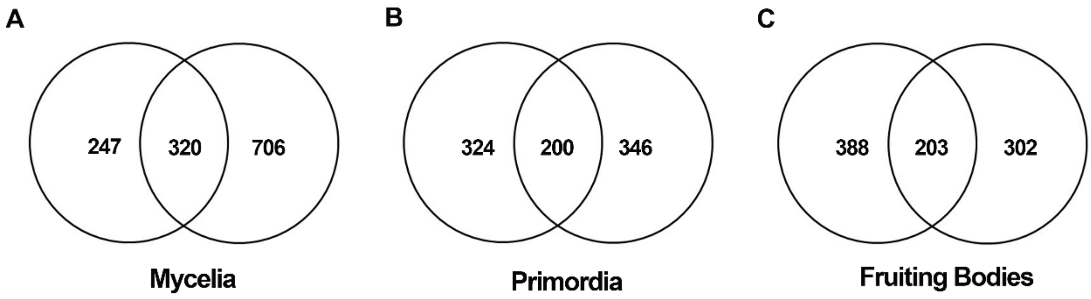

(B)

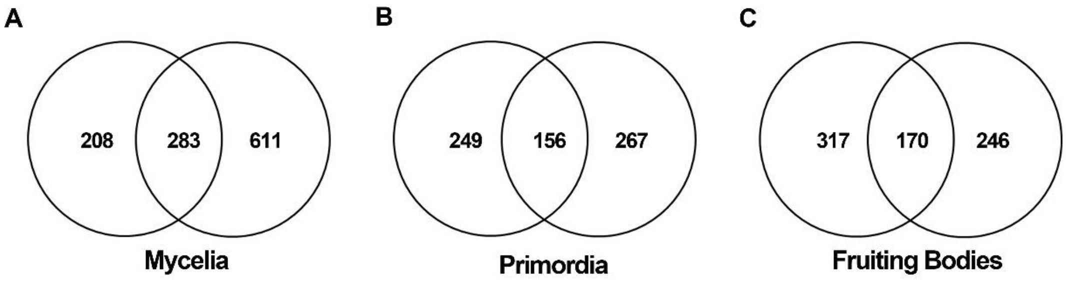

(C)

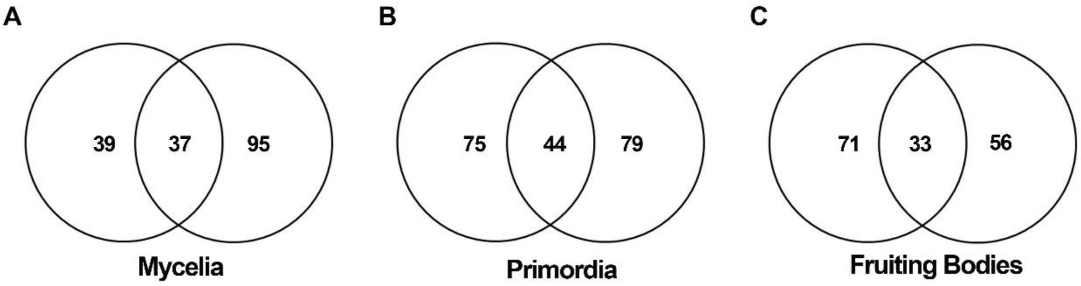

Figure S3 Numbers of exonic circRNAs have repeat elements in their left, right and control introns respectively.

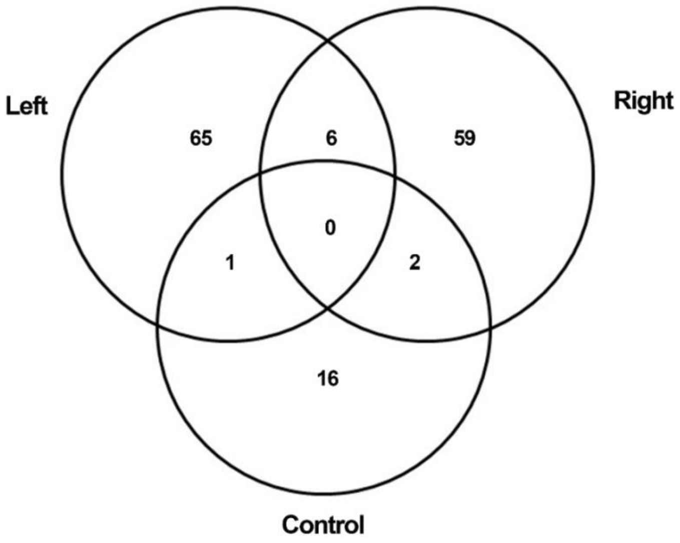

Figure S4 The full length gels and blots of PCR experiments for validating candidate circRNAs.

(A) Lines 1-4, Lines 5-8 and Lines 9-12 are the results of candidate circRNAs GaLu96scf31_39627_39859, GaLu96scf18_226622_227010 and GaLu96scf18_226875_227010, respectively. These results are corresponding to panel A, C and E in Figure 5, respectively. (B) The results of candidate circRNAs GaLu96scf35_328572_328844. They are corresponding to panel G. (C) The results of candidate circRNAs of GaLu96scf2_1057061_1057450. They are corresponding to panel I. (D) The results of candidate circRNAs GaLu96scf21_34416_34722. They are corresponding to panel K.

(A)

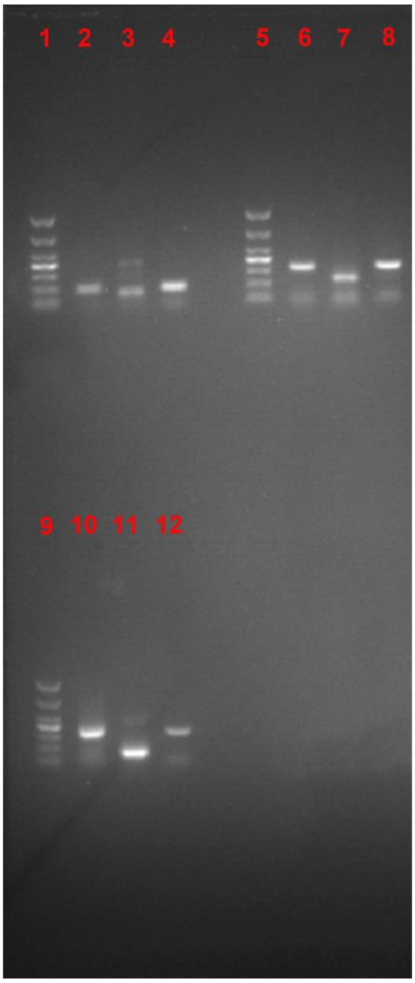

(B)

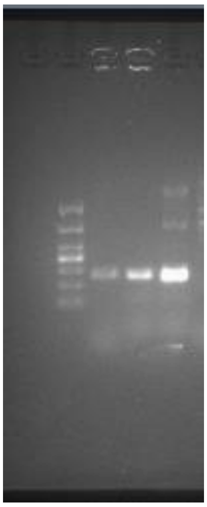

(C)

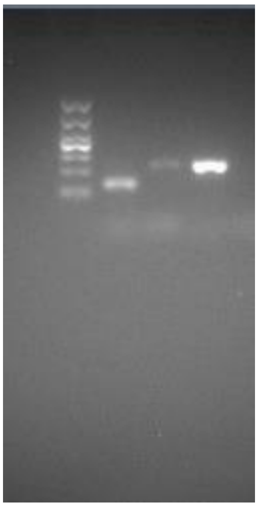

(D)

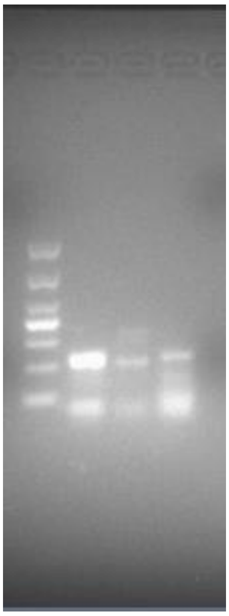

Table S1a Summary of the RNA-seq data generated from polyA(-)/Rnase R treated library. R1: replicate 1; R2: replicate 2.

**Developmental stages**	**Mycelia**		**Primodia**		**Fruiting Bodies**	
	**R1**	**R2**	**R1**	**R2**	**R1**	**R2**
Total reads(raw reads)	37495878	32352166	30525556	2996290	28424268	21619950
Clean reads	31283412	28866828	29245280	28556084	26439266	20988694
Stastic for clean reads:Q20	94.78	94.82	95.75	97.69	95.54	95.38
Stastic for clean reads:Q30	88.12	88.09	89.88	94.06	89.47	89.16
Stastic for clean reads:GC content	54.52	54.25	54.62	55.03	54.51	53.45
Tophat Total mapped reads (pairs)	19899893	16785938	17143430	17435822	16629797	11214578
Tophat Total multiple alignment	30551	65469	69992	88256	158846	1519420
Tophat Unique alignment	19869342	12080890	17073438	17347566	16470951	9774116
Tophat Mapping rate	63.60%	58.10%	58.60%	61.10%	62.90%	54.30%
Tophat fusion Total Input	11383519	12022769	12101850	11120262	9809469	10609802
Tophat fusion Total Mapped	328934	343065	314941	310355	370668	167261
Tophat fusion multiple alignments	100328	79385	132125	109837	142486	51668
Tophat fusion Unique alignment	228606	263680	11969725	200518	228182	115593
Tophat fusion mapping rate	2.89%	2.85%	2.60%	2.79%	3.78%	1.58%

Table S1b Summary of the RNA-seq data generated from polyA(-) libraries. R1: replicate 1; R2: replicate 2.

Developmental stages	Mycelia		Primodia		Fruiting Bodies	
	R1	R2	R1	R2	R1	R2
Total reads(raw reads)	34385168	32941534	37574716	33342852	32624858	31047910
Clean reads	33882672	32492742	30082137	32442788	31281026	30068422
Stastic for clean reads:Q20	97.12	97.14	96.52	96.78	96.56	95.47
Stastic for clean reads:Q30	92.76	92.78	91.34	91.82	91.41	89.34
Stastic for clean reads:GC content	48.45	48.41	54.26	54.95	52.94	53.1
Tophat Total mapped reads (pairs)	13778301	13236956	12070481	21343005	18752330	15140919
Tophat Total multiple alignment	445310	401423	170984	148054	136206	822038
Tophat Unique alignment	13332991	12835533	11899497	21194951	18616124	14318881
Tophat Mapping rate	40.70%	40.70%	63.40%	65.80%	59.90%	49.60%
Tophat fusion Total Input	20104371	19255786	13250352	11099783	12528696	15756135
Tophat fusion Total Mapped	294717	292012	215938	168115	225781	298830
Tophat fusion multiple alignments	53058	60422	36923	26224	50051	51021
Tophat fusion Unique alignment	241659	231590	179015	141891	175730	247809
Tophat fusion mapping rate	1.47%	1.52%	1.63%	1.51%	1.80%	1.90%

Table S2. List of identified circRNAs in *G. lucidum* based on RNA-Seq data generated from polyA(-)/RNase R treated libraries. R1: replicate 1; R2: replicate 2.

		Numbers of back spliced junction reads								
	Exon	Mycelia		Primordia		Fruiting Bodies		Name of		
exonic ID	Count	R1	R2	R1	R2	R1	R2	Parent Gene	Strand	exonic Type
GaLu96scf_35_328571_328844	1	2599	3480	11	17	31	22	GL24337	+	exonic
GaLu96scf_27_128048_128539	3	971	1370	523	538	586	338	GL23383	+	exonic
GaLu96scf_8_863122_863358	1	1509	1180	6	4	37	25	GL25929	-	exonic
GaLu96scf_24_643346_643626	1	531	1056	3	2	8	13	GL23172	-	exonic
GaLu96scf_3_711836_712165	2	434	897	0	6	11	3	GL23962	-	exonic
GaLu96scf_2_663567_664349	2	169	592	8	26	61	43	GL22656	-	exonic
GaLu96scf_2_1057061_1057245	1	245	425	9	10	38	17	GL22329	-	exonic
GaLu96scf_1_3332784_3333104	2	134	144	58	61	165	69	GL30048	+	exonic
GaLu96scf_7_847373_847794	2	137	436	0	1	0	0	GL25620	+	exonic
GaLu96scf_2_778974_780464	2	181	185	26	28	76	42	GL22662	+	exonic
GaLu96scf_31_39627_39859	1	133	281	18	14	31	17	GL24100	+	exonic
GaLu96scf_6_1278057_1278485	2	9	7	114	155	93	72	GL31640	-	exonic
GaLu96scf_45_47325_47584	2	213	228	0	0	0	0	GL28656	-	exonic
GaLu96scf_7_847373_848067	4	65	345	6	10	0	6	GL25620	+	exonic
GaLu96scf_4_1279082_1279396	1	168	218	4	11	8	18	GL24762	-	exonic
GaLu96scf_1_2046624_2046987	1	121	266	0	0	0	0	GL30934	-	exonic

GaLu96scf_14_977560_978372	1	11	50	76	72	85	65	GL27220	+	exonic
GaLu96scf_22_118127_118753	5	63	81	18	38	99	58	GL22968	+	exonic
GaLu96scf_11_567777_568108	2	101	218	0	0	0	0	GL26980	+	exonic
GaLu96scf_13_438876_439167	1	110	138	1	5	40	17	GL21226	+	exonic
GaLu96scf_33_107955_108150	1	81	71	19	11	84	45	GL24214	-	exonic
GaLu96scf_1_2922218_2922676	2	134	132	6	10	12	12	GL30011	+	exonic
GaLu96scf_5_516816_517239	1	94	197	2	0	0	2	GL17615	-	exonic
GaLu96scf_16_607479_608085	1	17	78	49	57	52	34	GL21693	-	exonic
GaLu96scf_2_1057061_1057450	2	76	162	8	10	19	7	GL22329	-	exonic
GaLu96scf_19_250245_250455	1	74	108	7	7	31	15	GL22149	-	exonic
GaLu96scf_12_922030_922271	1	74	85	9	21	33	11	GL20960	+	exonic
GaLu96scf_7_847373_847936	3	62	169	0	1	0	0	GL25620	+	exonic
GaLu96scf_11_1153012_1153342	2	46	111	12	13	30	14	GL20745	-	exonic
GaLu96scf_18_226622_227010	2	79	145	1	0	0	0	GL21985	-	exonic
GaLu96scf_5_36393_36732	2	106	80	3	4	0	5	GL25151	-	exonic
GaLu96scf_17_309529_309938	2	43	119	4	3	18	8	GL15753	-	exonic
GaLu96scf_13_436487_436788	2	77	85	0	8	18	3	GL21226	+	exonic
GaLu96scf_18_621638_622038	3	85	98	5	0	0	0	GL22024	-	exonic
GaLu96scf_9_981635_982113	3	22	81	14	32	14	21	GL26582	-	exonic
GaLu96scf_1_441534_441919	2	30	48	30	18	20	26	GL30097	-	exonic
GaLu96scf_2_1224538_1224876	1	48	48	13	11	24	20	GL22551	+	exonic
GaLu96scf_15_895046_895301	1	134	0	0	0	28	0	GL21512	+	intronic
GaLu96scf_21_181527_182048	3	39	65	15	11	13	13	GL22829	+	exonic
GaLu96scf_35_314043_314229	1	76	65	0	0	3	4	GL24340	-	exonic
GaLu96scf_7_848191_848404	1	81	66	0	0	0	0	GL25620	+	exonic

GaLu96scf_1_4154920_4155164	1	6	12	39	36	26	22	GL30108	+	intronic
GaLu96scf_29_427459_427700	1	56	17	15	17	26	8	GL23621	-	exonic
GaLu96scf_3_1675827_1676107	1	73	54	1	0	0	6	GL23899	+	exonic
GaLu96scf_5_1256765_1256931	1	22	17	10	22	54	8	GL28715	-	intronic
GaLu96scf_1_2004521_2004822	2	23	82	6	2	9	4	GL30538	-	exonic
GaLu96scf_10_633036_633434	2	24	38	12	19	24	9	GL20627	+	exonic
GaLu96scf_47_89640_90331	6	79	37	10	0	0	0	GL25106	+	exonic
GaLu96scf_32_269961_270152	1	79	43	0	0	0	0	GL24170	-	intronic
GaLu96scf_16_604576_605067	2	38	81	0	1	1	0	GL21653	-	exonic
GaLu96scf_43_107810_108039	1	42	53	4	8	9	5	GL28634	+	exonic
GaLu96scf_4_168492_169400	4	0	0	16	41	21	37	GL28477	+	exonic
GaLu96scf_5_1256745_1256931	1	0	0	113	0	0	0	GL28715	-	intronic
GaLu96scf_23_237146_237325	1	64	47	0	0	0	0	GL23044	-	intronic
GaLu96scf_23_615904_616279	2	25	63	1	11	0	6	GL16483	+	exonic
GaLu96scf_6_217665_218029	2	32	70	0	0	0	0	GL31658	+	exonic
GaLu96scf_16_544240_544486	1	30	70	0	0	0	0	GL18694	+	exonic
GaLu96scf_19_649330_649603	1	43	38	2	1	8	5	GL22217	-	exonic
GaLu96scf_5_1256747_1256931	1	19	22	10	8	33	5	GL28715	-	intronic
GaLu96scf_9_866454_867150	5	10	5	9	39	16	18	GL26572	-	exonic
GaLu96scf_32_269704_269946	1	23	67	0	0	0	2	GL24170	-	exonic
GaLu96scf_14_731709_732043	2	38	37	4	8	4	0	GL21362	-	exonic
GaLu96scf_7_1167778_1168011	1	11	8	16	12	27	17	GL25544	+	exonic
GaLu96scf_21_34416_34722	1	13	76	0	0	0	1	GL22778	+	exonic
GaLu96scf_26_136975_137250	1	10	43	6	3	23	4	GL23327	+	exonic
GaLu96scf_2_724504_724771	1	41	42	0	1	0	4	GL22518	-	exonic

GaLu96scf_1_3968929_3969173	1	28	57	0	0	2	0	GL29512	-	exonic
GaLu96scf_33_19167_19452	2	21	61	0	0	0	0	GL24198	+	exonic
GaLu96scf_37_231727_232145	3	24	37	7	0	3	10	GL17179	-	exonic
GaLu96scf_11_627413_627989	2	20	59	0	0	0	0	GL20764	+	exonic
GaLu96scf_12_716899_717289	1	29	40	1	3	4	0	GL21074	-	exonic
GaLu96scf_3_1675629_1676107	2	0	72	0	0	5	0	GL23899	+	exonic
GaLu96scf_1_4323938_4324354	3	26	15	4	17	7	7	GL30121	-	exonic
GaLu96scf_2_176740_177035	2	17	20	4	9	18	8	GL15974	+	exonic
GaLu96scf_5_681470_681784	1	34	36	0	0	6	0	GL17624	-	exonic
GaLu96scf_1_89101_89611	2	14	34	10	11	0	5	GL30875	-	exonic
GaLu96scf_25_479247_479504	1	0	0	22	6	35	11	GL23247	-	exonic
GaLu96scf_15_577800_578307	3	25	25	3	6	9	4	GL21539	+	exonic
GaLu96scf_18_621762_622038	2	48	24	0	0	0	0	GL22024	-	exonic
GaLu96scf_8_89554_89854	1	24	20	6	3	8	10	GL26030	-	exonic
GaLu96scf_12_451474_452244	2	9	36	9	12	2	2	GL18437	-	exonic
GaLu96scf_1_88856_89101	1	21	23	10	6	6	3	GL30875	-	intronic
GaLu96scf_17_725829_726187	1	7	31	11	12	3	5	GL15782	+	intronic
GaLu96scf_10_633173_633434	1	39	29	0	0	0	0	GL20627	+	exonic
GaLu96scf_4_138240_138550	2	17	24	3	0	21	2	GL24734	-	exonic
GaLu96scf_19_175500_176092	2	28	37	0	0	1	0	GL22184	+	exonic
GaLu96scf_2_778673_779108	2	0	5	10	19	20	12	GL22662	+	exonic
GaLu96scf_27_128806_129874	3	0	11	0	27	15	13	GL23383	+	exonic
GaLu96scf_2_889910_890290	2	44	21	0	0	0	0	GL16169	-	exonic
GaLu96scf_34_336501_336980	3	21	20	6	11	6	1	GL24263	-	exonic
GaLu96scf_7_1442180_1442333	1	13	21	3	3	19	6	GL28890	+	intronic

GaLu96scf_21_35045_35886	2	19	42	1	1	1	0	GL22778	+	exonic
GaLu96scf_9_820471_820877	3	20	20	8	4	6	5	GL26562	+	exonic
GaLu96scf_21_642308_642647	1	32	4	12	11	0	3	GL22854	-	intronic
GaLu96scf_25_72244_72482	1	24	11	6	14	0	7	GL23234	-	intronic
GaLu96scf_12_837790_838122	1	20	27	5	5	2	2	GL20950	+	exonic
GaLu96scf_22_171118_171475	1	11	17	3	11	9	10	GL22965	-	exonic
GaLu96scf_11_730171_730635	2	14	38	0	8	0	0	GL20772	-	exonic
GaLu96scf_1_213969_214288	2	22	36	0	0	1	0	GL30236	+	exonic
GaLu96scf_41_156524_157254	2	24	15	13	1	5	1	GL28597	-	exonic
GaLu96scf_1_1822874_1823210	3	0	58	0	0	0	0	GL29687	+	exonic
GaLu96scf_19_664017_664332	1	43	11	3	0	0	0	GL22212	+	exonic
GaLu96scf_3_1818467_1818647	1	12	19	2	5	14	5	GL23913	+	exonic
GaLu96scf_7_847536_847794	1	20	36	1	0	0	0	GL25620	+	exonic
GaLu96scf_15_966920_967395	3	0	38	2	7	3	6	GL21618	-	exonic
GaLu96scf_27_339069_339598	3	20	30	0	0	4	2	GL23420	+	exonic
GaLu96scf_4_619405_619652	1	11	42	1	0	0	2	GL24851	+	exonic
GaLu96scf_8_1159399_1159900	3	13	35	0	3	5	0	GL25837	-	exonic
GaLu96scf_16_675161_675427	2	22	17	2	3	7	4	GL15686	+	exonic
GaLu96scf_5_1100364_1100544	1	24	12	2	0	14	3	GL17543	+	exonic
GaLu96scf_12_449898_450282	1	15	19	2	7	7	4	GL18437	-	exonic
GaLu96scf_2_1463452_1463671	1	24	19	0	3	6	1	GL22567	-	exonic
GaLu96scf_9_1006778_1007111	1	18	23	1	2	6	3	GL20397	+	exonic
GaLu96scf_11_1234719_1235461	3	4	32	6	2	2	6	GL20816	-	exonic
GaLu96scf_7_148441_148680	1	25	11	3	1	10	2	GL25633	-	exonic
GaLu96scf_12_450878_451254	1	17	32	2	0	0	0	GL18437	-	exonic

GaLu96scf_31_264930_265127	1	25	26	0	0	0	0	GL24114	+	exonic
GaLu96scf_31_290588_290742	1	15	7	8	8	13	0	GL24058	+	intronic
GaLu96scf_7_1167778_1168333	2	0	13	14	8	6	10	GL25544	+	exonic
GaLu96scf_1_2047995_2049134	3	0	50	0	0	0	0	GL30239	+	exonic
GaLu96scf_14_295185_295479	1	27	23	0	0	0	0	GL18545	+	exonic
GaLu96scf_16_604798_605067	1	11	34	0	5	0	0	GL21653	-	exonic
GaLu96scf_18_884414_884741	3	19	31	0	0	0	0	GL22034	-	exonic
GaLu96scf_29_326624_326915	1	14	12	11	9	0	4	GL23585	+	intronic
GaLu96scf_6_670719_670947	1	14	23	4	2	5	2	GL31438	+	exonic
GaLu96scf_1_3141761_3142000	1	15	3	9	8	6	8	GL30662	+	intronic
GaLu96scf_7_783494_783783	1	0	10	11	15	6	7	GL28937	+	intronic
GaLu96scf_14_881012_881244	1	19	24	2	0	1	2	GL21446	+	intronic
GaLu96scf_2_1770335_1770882	3	0	3	14	6	18	7	GL22592	-	exonic
GaLu96scf_28_526348_526653	1	14	32	2	0	0	0	GL23538	+	exonic
GaLu96scf_4_1128146_1128467	2	0	8	19	4	13	4	GL24752	-	exonic
GaLu96scf_7_847373_848404	5	15	33	0	0	0	0	GL25620	+	exonic
GaLu96scf_11_612424_612944	3	26	17	0	0	4	0	GL20868	-	exonic
GaLu96scf_2_923644_924029	2	16	16	3	9	3	0	GL22451	+	exonic
GaLu96scf_35_259014_259521	4	17	30	0	0	0	0	GL24331	+	exonic
GaLu96scf_1_1408495_1408813	1	20	26	0	0	0	0	GL30901	+	exonic
GaLu96scf_11_101360_101610	1	11	30	0	0	5	0	GL20660	+	exonic
GaLu96scf_16_855760_856605	4	4	11	3	9	5	14	GL15699	-	exonic
GaLu96scf_18_227010_227256	1	11	10	7	0	15	3	GL21985	-	intronic
GaLu96scf_34_95773_96416	1	3	42	0	1	0	0	GL24286	+	exonic
GaLu96scf_2_979223_980204	2	0	0	10	25	8	2	GL27636	-	exonic

GaLu96scf_22_119250_120074	4	0	38	0	0	3	4	GL22968	+	exonic
GaLu96scf_22_181588_181808	1	22	23	0	0	0	0	GL22913	+	exonic
GaLu96scf_35_328844_329092	1	0	43	0	0	0	2	GL24337	+	intronic
GaLu96scf_25_426165_426869	4	16	22	4	2	0	0	GL31764	+	exonic
GaLu96scf_3_1140214_1140529	1	1	13	4	11	12	3	GL23798	+	exonic
GaLu96scf_5_10565_10871	2	17	23	0	0	4	0	GL25146	+	exonic
GaLu96scf_1_1292918_1293336	3	24	17	1	1	0	0	GL29863	-	exonic
GaLu96scf_1_2046413_2046987	2	21	22	0	0	0	0	GL30934	-	exonic
GaLu96scf_18_226875_227010	1	15	28	0	0	0	0	GL21985	-	exonic
GaLu96scf_2_179449_179714	2	24	19	0	0	0	0	GL27501	+	exonic
GaLu96scf_4_148792_149052	1	12	15	6	2	0	8	GL24464	+	exonic
GaLu96scf_10_1309498_1309814	2	14	2	6	10	8	2	GL20477	+	exonic
GaLu96scf_31_235519_235921	1	2	35	2	1	0	2	GL28294	+	exonic
GaLu96scf_12_387721_388104	2	5	5	7	10	8	6	GL21056	-	exonic
GaLu96scf_2_820827_822137	4	8	33	0	0	0	0	GL22666	+	exonic
GaLu96scf_28_434983_435391	1	24	17	0	0	0	0	GL23491	+	exonic
GaLu96scf_7_647053_647313	1	0	16	4	7	14	0	GL25610	-	exonic
GaLu96scf_1_1891179_1891452	1	26	1	1	6	2	4	GL29690	+	exonic
GaLu96scf_1_4761471_4761677	1	18	21	1	0	0	0	GL29593	-	exonic
GaLu96scf_17_39623_39835	1	0	6	0	13	18	3	GL21894	+	intronic
GaLu96scf_2_1682148_1682409	1	4	27	3	2	4	0	GL22369	-	exonic
GaLu96scf_20_142419_143191	2	11	15	0	5	4	5	GL22680	+	exonic
GaLu96scf_21_181854_182048	1	0	32	2	2	1	3	GL22829	+	exonic
GaLu96scf_25_71903_72224	2	11	29	0	0	0	0	GL23234	-	exonic
GaLu96scf_1_440858_441430	3	1	13	8	13	3	1	GL30097	-	exonic

GaLu96scf_1_823157_823531	2	2	2	12	8	6	9	GL29633	-	exonic
GaLu96scf_15_791486_791712	1	8	14	0	4	11	2	GL21542	+	exonic
GaLu96scf_2_202079_202358	1	15	10	0	1	7	6	GL16088	+	exonic
GaLu96scf_1_3585501_3585800	1	15	12	0	0	4	7	GL30712	+	exonic
GaLu96scf_12_1110164_1110488	1	0	19	9	3	4	3	GL18413	-	exonic
GaLu96scf_4_1040337_1040815	3	0	38	0	0	0	0	GL24479	-	exonic
GaLu96scf_9_864649_865042	3	0	13	2	3	10	10	GL26572	-	exonic
GaLu96scf_13_572959_573298	1	17	20	0	0	0	0	GL21186	-	exonic
GaLu96scf_3_1135050_1135563	1	17	20	0	0	0	0	GL23647	+	exonic
GaLu96scf_12_716899_717846	3	0	35	0	0	0	1	GL21074	-	exonic
GaLu96scf_1_822816_823309	2	2	7	4	5	15	2	GL29633	-	exonic
GaLu96scf_8_586612_586805	1	12	21	0	0	2	0	GL26114	-	exonic
GaLu96scf_8_731051_731388	2	0	5	7	9	7	7	GL25920	-	exonic
GaLu96scf_11_725031_725261	2	22	7	0	0	5	0	GL20718	-	exonic
GaLu96scf_6_579734_580190	2	0	9	12	4	5	4	GL31671	+	exonic
GaLu96scf_11_1283922_1284272	3	0	33	0	0	0	0	GL20814	+	exonic
GaLu96scf_2_313829_314427	3	8	16	2	7	0	0	GL22634	+	exonic
GaLu96scf_20_845487_845780	1	0	0	3	0	16	14	GL22726	-	exonic
GaLu96scf_21_814520_814865	1	11	20	0	0	0	2	GL16370	-	exonic
GaLu96scf_5_683753_684778	5	7	14	4	5	0	3	GL17625	-	exonic
GaLu96scf_19_175500_175732	1	22	10	0	0	0	0	GL22184	+	exonic
GaLu96scf_19_270134_270627	1	0	24	0	4	2	2	GL22252	+	exonic
GaLu96scf_4_988435_988869	3	11	17	0	0	4	0	GL24619	-	exonic
GaLu96scf_15_895046_895302	1	31	0	0	0	0	0	GL21512	+	intronic
GaLu96scf_5_683753_684096	2	10	15	0	0	0	6	GL17625	-	exonic

GaLu96scf_9_497310_497494	1	11	18	2	0	0	0	GL26637	-	exonic
GaLu96scf_9_729466_729718	1	7	3	4	6	5	6	GL26645	+	exonic
GaLu96scf_1_3002925_3003256	1	17	11	1	0	1	0	GL30023	+	exonic
GaLu96scf_17_937387_937830	3	0	0	11	0	11	8	GL18792	+	exonic
GaLu96scf_19_616057_616293	1	10	3	0	2	11	4	GL22214	-	exonic
GaLu96scf_22_124704_125002	1	12	15	0	3	0	0	GL22915	-	exonic
GaLu96scf_27_284010_284295	2	9	21	0	0	0	0	GL23411	+	exonic
GaLu96scf_3_1654376_1654824	2	15	15	0	0	0	0	GL23811	+	exonic
GaLu96scf_1_822816_823531	3	0	4	6	4	9	6	GL29633	-	exonic
GaLu96scf_29_246147_246434	1	8	17	0	0	4	0	GL23564	-	exonic
GaLu96scf_29_326624_326917	1	5	9	0	12	3	0	GL23585	+	intronic
GaLu96scf_29_50957_51214	1	10	8	0	3	4	4	GL23547	-	exonic
GaLu96scf_3_1654565_1654824	1	4	24	0	1	0	0	GL23811	+	exonic
GaLu96scf_4_1283551_1283837	2	18	11	0	0	0	0	GL24640	+	exonic
GaLu96scf_4_532307_532692	3	22	4	1	1	1	0	GL28557	-	exonic
GaLu96scf_7_527164_527536	1	15	10	1	0	1	2	GL20180	-	exonic
GaLu96scf_8_531717_531891	1	5	24	0	0	0	0	GL25996	-	exonic
GaLu96scf_10_1254810_1255309	1	0	13	9	3	1	2	GL20535	-	intronic
GaLu96scf_12_649243_649552	2	18	3	0	0	2	5	GL21066	-	exonic
GaLu96scf_2_718009_718422	2	0	19	0	4	2	3	GL22440	-	exonic
GaLu96scf_4_863734_864566	4	0	24	0	4	0	0	GL17361	-	exonic
GaLu96scf_6_796087_796305	1	6	15	1	0	6	0	GL31330	+	exonic
GaLu96scf_11_1309786_1310357	4	0	0	0	10	10	7	GL20818	+	exonic
GaLu96scf_28_437148_437556	1	14	13	0	0	0	0	GL23491	+	exonic
GaLu96scf_3_1853089_1853479	1	0	14	1	4	4	4	GL23696	+	intronic

GaLu96scf_44_81238_81513	1	1	15	3	0	6	2	GL17453	+	intronic
GaLu96scf_5_1042559_1042864	1	6	14	3	0	1	3	GL25355	-	exonic
GaLu96scf_14_125920_126149	1	20	4	2	0	0	0	GL21387	-	exonic
GaLu96scf_17_309529_310109	3	10	16	0	0	0	0	GL15753	-	exonic
GaLu96scf_4_1127979_1128467	3	0	6	9	0	7	4	GL24752	-	exonic
GaLu96scf_5_288195_288558	2	0	24	2	0	0	0	GL25300	+	exonic
GaLu96scf_1_1832393_1832925	4	0	8	7	1	3	6	GL30917	+	exonic
GaLu96scf_1_2047995_2048512	2	2	20	0	3	0	0	GL30239	+	exonic
GaLu96scf_10_597910_598302	1	0	2	9	5	4	5	GL20626	+	intronic
GaLu96scf_10_941606_941818	1	20	5	0	0	0	0	GL20528	-	exonic
GaLu96scf_11_185670_186176	2	4	18	1	1	1	0	GL20738	+	exonic
GaLu96scf_12_922271_922428	1	0	7	3	9	6	0	GL20960	+	intronic
GaLu96scf_21_562743_563142	1	10	0	2	5	1	7	GL16341	-	exonic
GaLu96scf_3_1121840_1122206	2	10	15	0	0	0	0	GL23646	+	exonic
GaLu96scf_7_455907_456236	1	3	20	0	2	0	0	GL25740	+	exonic
GaLu96scf_7_851851_852235	3	6	17	0	2	0	0	GL25671	-	exonic
GaLu96scf_11_965780_967233	3	0	23	0	1	0	0	GL20883	+	exonic
GaLu96scf_13_1093913_1094212	1	11	10	0	0	1	2	GL15367	-	intronic
GaLu96scf_18_382504_382751	2	11	9	0	4	0	0	GL21997	-	exonic
GaLu96scf_2_2221011_2221383	2	0	23	0	0	0	1	GL22403	-	exonic
GaLu96scf_2_821935_822137	1	12	12	0	0	0	0	GL22666	+	exonic
GaLu96scf_22_744830_745187	1	8	16	0	0	0	0	GL22950	+	exonic
GaLu96scf_3_1102021_1102243	1	0	16	0	0	4	4	GL23794	-	exonic
GaLu96scf_34_337457_337659	2	20	0	0	1	1	2	GL24263	-	exonic
GaLu96scf_4_140106_140434	1	0	17	0	4	2	1	GL17243	-	exonic

GaLu96scf_4_858934_859286	2	17	3	1	0	3	0	GL24604	-	exonic
GaLu96scf_41_157014_157254	1	1	8	4	0	5	6	GL28597	-	exonic
GaLu96scf_45_134109_134540	2	0	0	0	0	11	13	GL25065	+	exonic
GaLu96scf_1_3517326_3517680	2	0	0	6	12	5	0	GL29481	+	exonic
GaLu96scf_10_956005_956405	1	10	13	0	0	0	0	GL20648	+	exonic
GaLu96scf_17_302614_303070	3	14	0	5	4	0	0	GL21921	-	exonic
GaLu96scf_17_791214_792475	5	0	9	1	4	5	4	GL21844	+	exonic
GaLu96scf_19_390915_391439	4	9	14	0	0	0	0	GL22154	+	exonic
GaLu96scf_2_2184361_2184635	2	21	2	0	0	0	0	GL22396	+	exonic
GaLu96scf_2_993116_993709	1	5	3	1	7	7	0	GL22678	-	exonic
GaLu96scf_3_586110_586294	1	0	16	4	0	3	0	GL23736	+	exonic
GaLu96scf_31_234356_234749	1	5	18	0	0	0	0	GL28294	+	exonic
GaLu96scf_32_29553_29915	2	5	11	0	0	0	7	GL28308	-	exonic
GaLu96scf_47_129180_129519	1	0	18	0	0	3	2	GL25114	+	exonic
GaLu96scf_5_621587_621966	2	7	16	0	0	0	0	GL25241	+	exonic
GaLu96scf_50_63748_64028	1	1	15	0	3	1	3	GL28774	+	exonic
GaLu96scf_7_847536_847936	2	15	8	0	0	0	0	GL25620	+	exonic
GaLu96scf_8_1164203_1164592	3	8	15	0	0	0	0	GL20241	-	exonic
GaLu96scf_8_89554_90055	2	0	5	3	7	8	0	GL26030	-	exonic
GaLu96scf_1_407026_407234	1	10	11	0	0	0	1	GL30095	-	exonic
GaLu96scf_1_4533060_4533570	3	7	0	9	5	1	0	GL29573	-	exonic
GaLu96scf_12_810175_810526	2	7	7	0	0	7	1	GL20955	-	exonic
GaLu96scf_16_964984_965284	1	0	18	0	0	4	0	GL15708	-	exonic
GaLu96scf_26_117579_117907	1	11	10	0	0	0	1	GL16639	+	exonic
GaLu96scf_31_450431_450722	1	0	20	1	1	0	0	GL24131	+	exonic

GaLu96scf_8_1080012_1080573	2	19	0	0	3	0	0	GL26045	-	exonic
GaLu96scf_15_894656_895046	2	0	4	4	7	0	6	GL21512	+	exonic
GaLu96scf_16_207724_208080	1	0	20	0	1	0	0	GL21684	-	exonic
GaLu96scf_17_692008_692283	2	3	18	0	0	0	0	GL21953	+	exonic
GaLu96scf_20_869159_869376	1	18	1	0	0	0	2	GL27716	-	exonic
GaLu96scf_23_535599_536009	2	0	17	0	0	4	0	GL27854	-	exonic
GaLu96scf_3_1899354_1899568	1	1	8	0	0	10	2	GL23921	+	exonic
GaLu96scf_47_92737_93159	1	0	21	0	0	0	0	GL25107	+	exonic
GaLu96scf_1_2922471_2922676	1	6	9	1	0	0	4	GL30011	+	exonic
GaLu96scf_1_407026_407504	2	6	7	6	0	1	0	GL30095	-	exonic
GaLu96scf_11_596697_596895	1	0	11	0	0	5	4	GL20763	-	exonic
GaLu96scf_15_527991_528366	2	12	6	0	0	2	0	GL21580	+	exonic
GaLu96scf_17_301396_301681	2	19	1	0	0	0	0	GL21921	-	exonic
GaLu96scf_31_120722_121354	2	11	2	1	0	2	4	GL24079	+	exonic
GaLu96scf_31_201444_202379	2	0	9	4	0	5	2	GL24088	-	exonic
GaLu96scf_34_95413_95660	1	14	2	3	1	0	0	GL24286	+	exonic
GaLu96scf_4_1782074_1782367	1	18	0	0	2	0	0	GL24804	+	exonic
GaLu96scf_43_149820_150177	2	20	0	0	0	0	0	GL24996	-	exonic
GaLu96scf_46_40959_41378	3	0	20	0	0	0	0	GL28673	+	exonic
GaLu96scf_6_284761_285099	1	0	9	1	3	4	3	GL31157	-	exonic
GaLu96scf_12_667804_668296	1	0	18	0	0	0	1	GL21067	-	exonic
GaLu96scf_15_528104_528366	1	15	3	0	0	1	0	GL21580	+	exonic
GaLu96scf_15_577641_578307	4	4	13	0	2	0	0	GL21539	+	exonic
GaLu96scf_24_410504_410987	1	0	19	0	0	0	0	GL23153	-	intronic
GaLu96scf_4_1306972_1307375	1	12	3	1	0	3	0	GL24505	-	exonic

GaLu96scf_5_521739_521956	1	0	7	1	1	7	3	GL25231	+	exonic
GaLu96scf_7_210293_210747	2	13	0	1	0	3	2	GL28919	+	exonic
GaLu96scf_9_150313_150641	2	7	1	1	0	8	2	GL26588	-	exonic
GaLu96scf_11_798425_798658	1	1	17	0	0	0	0	GL20726	-	intronic
GaLu96scf_13_438564_439167	2	9	6	0	0	0	3	GL21226	+	exonic
GaLu96scf_2_1724880_1725615	3	0	18	0	0	0	0	GL22587	+	exonic
GaLu96scf_22_187637_188188	2	0	3	9	2	4	0	GL22966	-	exonic
GaLu96scf_3_1242983_1243427	1	0	15	0	3	0	0	GL23880	+	exonic
GaLu96scf_3_586110_586630	2	16	0	0	2	0	0	GL23736	+	exonic
GaLu96scf_31_39414_39859	2	13	3	0	0	2	0	GL24100	-	exonic
GaLu96scf_31_430223_430702	4	0	15	0	0	3	0	GL24072	+	exonic
GaLu96scf_5_683753_684258	3	5	7	2	2	1	1	GL17625	-	exonic
GaLu96scf_5_882253_882426	1	5	0	5	2	5	1	GL17637	-	intronic
GaLu96scf_8_815914_816098	1	0	8	0	8	0	2	GL17965	+	intronic
GaLu96scf_1_1430262_1430474	1	0	14	0	1	2	0	GL30469	-	exonic
GaLu96scf_10_1363675_1363940	1	15	2	0	0	0	0	GL20592	-	exonic
GaLu96scf_12_358451_359047	2	5	11	0	0	0	1	GL27036	+	exonic
GaLu96scf_12_450300_450878	1	0	8	3	6	0	0	GL18437	-	intronic
GaLu96scf_13_1002066_1002338	2	0	16	1	0	0	0	GL21206	+	exonic
GaLu96scf_13_1104139_1104490	1	7	3	1	1	3	2	GL21214	+	exonic
GaLu96scf_13_363373_363611	2	17	0	0	0	0	0	GL21117	+	exonic
GaLu96scf_13_562681_563010	2	0	10	0	0	7	0	GL18500	-	exonic
GaLu96scf_19_649636_649805	1	9	7	0	0	0	1	GL22217	-	intronic
GaLu96scf_2_1238285_1238581	2	0	13	0	0	4	0	GL22552	+	exonic
GaLu96scf_2_1262267_1262709	4	0	0	0	0	12	5	GL22343	+	exonic

GaLu96scf_26_549215_549566	1	0	0	10	7	0	0	GL23326	-	intronic
GaLu96scf_3_1674340_1675076	4	0	17	0	0	0	0	GL23899	+	exonic
GaLu96scf_3_1898905_1899335	1	0	0	0	0	17	0	GL23921	+	intronic
GaLu96scf_4_6819_7159	1	7	4	2	2	1	1	GL24715	+	exonic
GaLu96scf_42_145252_145529	1	0	12	0	2	1	2	GL17424	-	exonic
GaLu96scf_45_69519_69787	1	17	0	0	0	0	0	GL25070	+	exonic
GaLu96scf_50_52088_52940	1	14	0	0	3	0	0	GL25484	+	intronic
GaLu96scf_7_499827_500109	1	6	11	0	0	0	0	GL28931	+	exonic
GaLu96scf_1_1143289_1143591	2	15	0	1	0	0	0	GL29852	+	exonic
GaLu96scf_1_4020862_4021597	5	0	0	0	9	7	0	GL30770	+	exonic
GaLu96scf_1_4775922_4776176	1	11	0	0	0	2	3	GL31086	-	exonic
GaLu96scf_10_991858_992304	2	2	13	0	0	0	1	GL20560	+	exonic
GaLu96scf_11_612651_612944	2	9	5	2	0	0	0	GL20868	-	exonic
GaLu96scf_12_358606_358870	1	0	16	0	0	0	0	GL27036	+	intronic
GaLu96scf_15_577955_578307	2	5	7	2	0	1	1	GL21539	+	exonic
GaLu96scf_17_474886_475109	1	9	7	0	0	0	0	GL21931	-	exonic
GaLu96scf_17_922528_922707	1	0	15	1	0	0	0	GL21891	-	intronic
GaLu96scf_2_1544548_1545992	4	0	15	1	0	0	0	GL22569	+	exonic
GaLu96scf_20_109747_109966	1	13	0	2	0	0	1	GL22704	-	intronic
GaLu96scf_24_18964_19388	2	0	16	0	0	0	0	GL23161	-	exonic
GaLu96scf_31_202028_202379	1	6	10	0	0	0	0	GL24088	-	exonic
GaLu96scf_5_1256741_1256931	1	0	0	0	0	16	0	GL28715	-	intronic
GaLu96scf_5_1382118_1382794	2	6	0	0	3	3	4	GL25382	-	exonic
GaLu96scf_9_1049221_1049550	2	4	12	0	0	0	0	GL26471	+	exonic
GaLu96scf_1_1296928_1297282	1	0	0	3	3	4	5	GL30461	-	intronic

GaLu96scf_1_3294448_3294739	1	9	5	0	0	0	1	GL31004	+	exonic
GaLu96scf_1_4023593_4023916	1	0	9	0	0	0	6	GL30771	+	exonic
GaLu96scf_12_982987_983486	3	3	10	0	0	1	1	GL15342	-	exonic
GaLu96scf_15_58153_58592	3	0	15	0	0	0	0	GL18595	-	exonic
GaLu96scf_19_99310_99744	1	2	11	2	0	0	0	GL22136	-	exonic
GaLu96scf_2_1034675_1034909	1	0	9	0	1	0	5	GL22327	-	exonic
GaLu96scf_2_820827_821844	3	0	15	0	0	0	0	GL22666	+	exonic
GaLu96scf_21_34722_35031	1	0	15	0	0	0	0	GL22778	+	intronic
GaLu96scf_28_525829_526191	1	8	4	0	0	3	0	GL23538	+	exonic
GaLu96scf_3_1113081_1113421	1	8	0	0	4	0	3	GL23795	-	exonic
GaLu96scf_3_1121840_1122081	1	0	15	0	0	0	0	GL23646	+	exonic
GaLu96scf_3_1899568_1899775	1	0	1	0	6	8	0	GL23921	+	intronic
GaLu96scf_3_704958_706056	3	0	15	0	0	0	0	GL23755	+	exonic
GaLu96scf_31_376798_377183	3	11	0	0	2	2	0	GL24119	+	exonic
GaLu96scf_38_176312_176825	3	10	5	0	0	0	0	GL24391	+	exonic
GaLu96scf_4_169756_169975	1	14	0	1	0	0	0	GL28477	+	exonic
GaLu96scf_4_740674_740930	1	0	15	0	0	0	0	GL24591	-	exonic
GaLu96scf_5_138024_138175	1	0	0	2	6	3	4	GL25276	+	intronic
GaLu96scf_5_417693_418112	3	6	0	2	4	0	3	GL25223	+	exonic
GaLu96scf_5_811662_812027	1	11	4	0	0	0	0	GL25253	+	exonic
GaLu96scf_6_356445_356910	1	0	15	0	0	0	0	GL31415	+	exonic
GaLu96scf_7_527164_527672	2	0	7	4	3	0	1	GL20180	-	exonic
GaLu96scf_8_731122_731302	1	0	0	8	7	0	0	GL25920	-	intronic
GaLu96scf_1_2346905_2347327	2	0	7	2	0	3	2	GL30566	+	exonic
GaLu96scf_14_989202_990323	6	0	14	0	0	0	0	GL15529	-	exonic

GaLu96scf_15_561186_562040	2	0	14	0	0	0	0	GL18632	+	exonic
GaLu96scf_15_888470_888722	1	0	13	0	0	1	0	GL21511	+	intronic
GaLu96scf_17_72501_72680	1	9	5	0	0	0	0	GL21904	-	intronic
GaLu96scf_18_474051_474301	1	3	7	0	2	0	2	GL22006	+	intronic
GaLu96scf_2_1497801_1497965	1	11	3	0	0	0	0	GL22362	+	exonic
GaLu96scf_24_131181_131394	1	0	0	7	2	4	1	GL23137	-	intronic
GaLu96scf_27_428519_428861	1	9	5	0	0	0	0	GL16711	+	exonic
GaLu96scf_3_495478_495826	2	0	1	0	3	7	3	GL23731	-	exonic
GaLu96scf_3_586294_586517	1	8	0	2	2	2	0	GL23736	+	intronic
GaLu96scf_33_91707_92921	3	0	10	1	3	0	0	GL24196	-	exonic
GaLu96scf_34_45294_45620	2	5	1	3	4	0	1	GL24242	-	exonic
GaLu96scf_35_54893_55259	3	0	14	0	0	0	0	GL17140	+	exonic
GaLu96scf_45_107155_107404	1	14	0	0	0	0	0	GL25061	+	exonic
GaLu96scf_5_640216_640543	2	0	14	0	0	0	0	GL25243	+	exonic
GaLu96scf_6_1268432_1268833	3	13	0	0	1	0	0	GL31192	+	exonic
GaLu96scf_7_1416763_1417203	1	7	0	1	1	5	0	GL25706	+	exonic
GaLu96scf_9_1425968_1426155	1	12	2	0	0	0	0	GL29184	-	exonic
GaLu96scf_1_1560803_1561066	2	0	9	0	1	2	1	GL29355	+	exonic
GaLu96scf_1_2047995_2048234	1	0	13	0	0	0	0	GL30239	+	exonic
GaLu96scf_1_2659518_2659735	1	0	0	5	3	4	1	GL29411	-	exonic
GaLu96scf_1_96079_96323	1	0	0	4	6	1	2	GL30174	+	intronic
GaLu96scf_11_292610_293000	1	0	0	4	9	0	0	GL18323	-	intronic
GaLu96scf_13_985328_985970	1	0	13	0	0	0	0	GL15443	+	exonic
GaLu96scf_14_202279_202474	1	3	5	0	0	3	2	GL21342	-	intronic
GaLu96scf_16_623035_623891	4	0	10	0	1	2	0	GL21694	-	exonic

GaLu96scf_19_506883_507372	1	2	8	0	0	1	2	GL15931	-	exonic
GaLu96scf_28_510504_510669	1	0	0	7	1	4	1	GL23508	+	intronic
GaLu96scf_4_1040438_1040815	2	5	7	0	0	0	1	GL24479	-	exonic
GaLu96scf_4_941240_941524	1	6	0	0	3	3	1	GL17366	+	exonic
GaLu96scf_5_1045707_1046124	3	0	6	2	5	0	0	GL25280	-	exonic
GaLu96scf_6_1422199_1422635	3	0	12	0	0	1	0	GL31393	+	exonic
GaLu96scf_8_593249_593534	2	0	10	0	0	3	0	GL25902	+	exonic
GaLu96scf_8_731120_731302	1	0	8	0	0	2	3	GL25920	-	intronic
GaLu96scf_9_1035105_1035555	3	0	8	1	3	0	1	GL26477	-	exonic
GaLu96scf_1_2691071_2691374	1	0	12	0	0	0	0	GL30273	+	exonic
GaLu96scf_1_3450815_3451106	1	4	8	0	0	0	0	GL30704	-	exonic
GaLu96scf_1_3923133_3923420	1	1	2	3	2	2	2	GL30361	+	exonic
GaLu96scf_1_4548752_4549297	1	7	5	0	0	0	0	GL30386	-	exonic
GaLu96scf_1_613526_613932	1	0	12	0	0	0	0	GL29617	+	exonic
GaLu96scf_1_835280_835527	1	8	1	2	0	1	0	GL30851	+	exonic
GaLu96scf_11_131486_132103	1	12	0	0	0	0	0	GL20790	-	intronic
GaLu96scf_11_611428_611980	3	0	0	0	12	0	0	GL20868	-	exonic
GaLu96scf_11_714664_715040	2	4	6	0	0	0	2	GL20771	-	exonic
GaLu96scf_12_1230662_1230959	1	0	2	5	2	2	1	GL15290	+	intronic
GaLu96scf_13_62206_62569	2	12	0	0	0	0	0	GL27080	+	exonic
GaLu96scf_15_817686_817952	2	12	0	0	0	0	0	GL21508	+	exonic
GaLu96scf_15_888470_888724	1	12	0	0	0	0	0	GL21511	+	intronic
GaLu96scf_2_1297860_1298666	3	0	1	3	1	2	5	GL16020	+	exonic
GaLu96scf_2_2104852_2105011	1	7	0	2	3	0	0	GL22393	+	intronic
GaLu96scf_22_119250_119804	2	3	9	0	0	0	0	GL22968	+	exonic

GaLu96scf_22_187637_187860	1	9	0	1	0	1	1	GL22966	-	exonic
GaLu96scf_29_261029_261362	1	0	5	0	0	7	0	GL23582	+	exonic
GaLu96scf_29_351483_351829	1	0	0	6	0	2	4	GL16798	-	intronic
GaLu96scf_3_1674497_1674803	2	9	0	0	3	0	0	GL23899	+	exonic
GaLu96scf_31_210887_211133	1	12	0	0	0	0	0	GL24081	-	intronic
GaLu96scf_31_83945_84343	2	8	4	0	0	0	0	GL28282	-	exonic
GaLu96scf_4_168492_169034	3	0	3	0	0	6	3	GL28477	+	exonic
GaLu96scf_4_169756_170276	2	0	1	2	4	4	1	GL28477	+	exonic
GaLu96scf_4_988325_988869	4	0	12	0	0	0	0	GL24619	-	exonic
GaLu96scf_43_107810_108315	2	0	8	2	0	2	0	GL28634	+	exonic
GaLu96scf_5_595052_595416	2	0	8	0	0	4	0	GL25234	+	exonic
GaLu96scf_5_686345_686732	2	6	6	0	0	0	0	GL25319	+	exonic
GaLu96scf_8_1022209_1023370	2	0	0	5	7	0	0	GL25822	-	exonic
GaLu96scf_8_549342_549577	2	8	2	0	0	2	0	GL25899	+	exonic
GaLu96scf_9_583208_583481	1	10	2	0	0	0	0	GL18154	-	exonic
GaLu96scf_1_1046885_1047489	2	11	0	0	0	0	0	GL30887	-	exonic
GaLu96scf_1_224262_224453	1	4	5	0	0	2	0	GL29930	+	exonic
GaLu96scf_1_3550729_3551062	2	11	0	0	0	0	0	GL30069	-	exonic
GaLu96scf_1_3622004_3622288	2	0	5	0	1	5	0	GL30076	-	exonic
GaLu96scf_1_4115967_4116481	2	1	10	0	0	0	0	GL29535	+	exonic
GaLu96scf_1_441144_441430	2	7	0	1	1	1	1	GL30097	-	exonic
GaLu96scf_10_547494_547761	1	5	0	4	2	0	0	GL15096	-	exonic
GaLu96scf_11_100041_100404	2	0	0	4	4	3	0	GL20660	+	exonic
GaLu96scf_12_280250_280639	2	0	11	0	0	0	0	GL15296	+	exonic
GaLu96scf_13_1074725_1075149	1	3	7	0	1	0	0	GL21210	+	exonic

GaLu96scf_14_997917_998788	4	0	3	7	1	0	0	GL21283	-	exonic
GaLu96scf_17_470660_470904	1	11	0	0	0	0	0	GL21829	-	exonic
GaLu96scf_18_567176_568351	4	0	0	4	4	0	3	GL27401	-	exonic
GaLu96scf_2_1541899_1545769	6	0	3	3	0	5	0	GL22569	+	exonic
GaLu96scf_22_682319_682655	1	0	7	0	0	2	2	GL19186	+	exonic
GaLu96scf_23_136332_136641	1	3	1	2	0	1	4	GL23075	-	intronic
GaLu96scf_26_547883_548228	2	0	0	3	0	4	4	GL23326	-	exonic
GaLu96scf_3_1293950_1294103	1	8	3	0	0	0	0	GL23885	-	intronic
GaLu96scf_3_1674340_1674803	3	3	8	0	0	0	0	GL23899	+	exonic
GaLu96scf_3_575832_576089	2	3	0	1	0	5	2	GL23742	-	exonic
GaLu96scf_32_269962_270152	1	8	2	0	0	1	0	GL24170	-	intronic
GaLu96scf_33_18524_19026	1	10	1	0	0	0	0	GL24198	+	exonic
GaLu96scf_4_1111596_1111926	1	3	3	2	0	3	0	GL19760	-	intronic
GaLu96scf_5_517364_518001	1	4	7	0	0	0	0	GL17615	-	exonic
GaLu96scf_6_963910_964283	2	0	9	1	0	1	0	GL31622	+	exonic
GaLu96scf_9_613750_614179	1	4	7	0	0	0	0	GL18163	+	intronic
GaLu96scf_9_981294_981635	1	3	5	0	1	0	2	GL26582	-	intronic
GaLu96scf_1_1137382_1137856	3	0	0	0	6	4	0	GL30182	-	exonic
GaLu96scf_1_1293399_1293832	1	0	8	0	0	0	2	GL29863	-	intronic
GaLu96scf_1_1296926_1297282	1	2	8	0	0	0	0	GL30461	-	intronic
GaLu96scf_1_1533440_1533730	1	2	3	0	3	2	0	GL29359	-	exonic
GaLu96scf_1_1744520_1744982	3	10	0	0	0	0	0	GL30217	+	exonic
GaLu96scf_1_2158293_2158658	2	8	2	0	0	0	0	GL30942	+	exonic
GaLu96scf_1_2457103_2457612	2	10	0	0	0	0	0	GL30586	-	exonic
GaLu96scf_1_2575581_2575944	2	0	6	0	4	0	0	GL30598	-	exonic

GaLu96scf_1_3183139_3183352	1	0	10	0	0	0	0	GL30324	-	exonic
GaLu96scf_10_1396074_1396289	2	0	8	2	0	0	0	GL20588	+	exonic
GaLu96scf_11_965353_965635	1	0	3	0	2	2	3	GL20883	+	exonic
GaLu96scf_12_837790_838612	2	10	0	0	0	0	0	GL20950	+	exonic
GaLu96scf_13_66924_67136	1	10	0	0	0	0	0	GL21093	+	exonic
GaLu96scf_14_125920_126881	2	0	6	2	0	2	0	GL21387	-	exonic
GaLu96scf_14_824459_824759	1	0	5	1	0	0	4	GL15526	-	intronic
GaLu96scf_14_989976_990323	2	0	4	0	4	1	1	GL15529	-	exonic
GaLu96scf_15_50637_50979	1	0	0	4	0	0	6	GL21556	-	exonic
GaLu96scf_15_573883_574306	3	10	0	0	0	0	0	GL21586	-	exonic
GaLu96scf_15_825007_825259	1	0	10	0	0	0	0	GL21605	-	intronic
GaLu96scf_15_838752_839281	1	0	0	3	4	2	1	GL21608	-	exonic
GaLu96scf_17_71814_72193	2	0	8	0	0	0	2	GL21904	-	exonic
GaLu96scf_18_617342_617818	2	0	0	2	5	3	0	GL27410	+	exonic
GaLu96scf_18_621638_621839	2	0	0	3	3	4	0	GL22024	-	exonic
GaLu96scf_2_1056816_1057245	2	2	4	2	0	2	0	GL22329	-	exonic
GaLu96scf_2_1579790_1580086	1	10	0	0	0	0	0	GL16044	+	exonic
GaLu96scf_2_2124181_2124502	1	0	0	2	6	0	2	GL22615	+	intronic
GaLu96scf_20_474304_475276	3	10	0	0	0	0	0	GL22753	+	exonic
GaLu96scf_24_374067_374477	2	4	6	0	0	0	0	GL23146	+	exonic
GaLu96scf_27_128508_128850	2	7	0	0	0	0	3	GL23383	+	exonic
GaLu96scf_3_1178883_1179123	1	0	9	0	0	1	0	GL23655	-	exonic
GaLu96scf_3_1675305_1676107	3	3	2	0	0	5	0	GL23899	+	exonic
GaLu96scf_3_663522_663837	1	0	3	4	2	0	1	GL23754	-	intronic
GaLu96scf_30_120197_120467	2	10	0	0	0	0	0	GL24012	-	exonic

GaLu96scf_30_183359_183598	1	9	0	0	1	0	0	GL28255	-	exonic
GaLu96scf_31_98237_98568	1	10	0	0	0	0	0	GL24047	-	exonic
GaLu96scf_4_331341_331861	3	0	10	0	0	0	0	GL17331	-	exonic
GaLu96scf_45_66710_67086	2	0	9	0	0	1	0	GL17472	-	exonic
GaLu96scf_5_1256746_1256931	1	0	6	0	0	0	4	GL28715	-	intronic
GaLu96scf_7_1121284_1121658	1	10	0	0	0	0	0	GL28863	+	exonic
GaLu96scf_8_1086287_1086584	1	0	10	0	0	0	0	GL25820	+	exonic
GaLu96scf_8_413101_413324	1	0	10	0	0	0	0	GL26101	+	exonic
GaLu96scf_9_1014924_1015283	2	3	4	0	0	3	0	GL26475	-	exonic
GaLu96scf_9_1106239_1106552	2	8	2	0	0	0	0	GL26480	+	exonic
GaLu96scf_9_1409566_1409817	3	0	10	0	0	0	0	GL26506	+	exonic
GaLu96scf_9_1418381_1418731	2	10	0	0	0	0	0	GL26510	-	exonic
GaLu96scf_9_1521817_1522123	2	4	6	0	0	0	0	GL26620	-	exonic
GaLu96scf_1_2046624_2047207	2	0	9	0	0	0	0	GL30934	-	exonic
GaLu96scf_1_223979_224453	2	0	0	2	0	3	4	GL29930	+	exonic
GaLu96scf_1_3517326_3518004	3	0	0	5	4	0	0	GL29481	+	exonic
GaLu96scf_1_4154920_4155161	1	5	0	3	0	0	1	GL30108	+	intronic
GaLu96scf_1_4323600_4323938	1	0	0	3	5	0	1	GL30121	-	intronic
GaLu96scf_1_4437569_4437931	2	0	0	3	0	6	0	GL30807	-	exonic
GaLu96scf_11_713891_714215	1	0	5	0	0	4	0	GL20771	-	exonic
GaLu96scf_13_1074454_1074622	1	0	3	0	0	0	6	GL21210	+	intronic
GaLu96scf_14_474829_475175	1	5	0	0	3	1	0	GL21351	+	exonic
GaLu96scf_15_577641_578017	3	0	9	0	0	0	0	GL21539	+	exonic
GaLu96scf_16_219107_219575	2	0	5	4	0	0	0	GL15663	+	exonic
GaLu96scf_16_358893_359309	1	0	8	0	1	0	0	GL27298	-	exonic

GaLu96scf_16_607116_607479	1	0	2	4	0	3	0	GL21693	-	intronic
GaLu96scf_17_425284_425663	1	9	0	0	0	0	0	GL18762	-	exonic
GaLu96scf_17_538649_538861	1	4	2	1	0	2	0	GL15763	-	exonic
GaLu96scf_18_509337_509757	2	7	0	0	0	2	0	GL15847	+	exonic
GaLu96scf_19_48561_48872	1	6	0	2	0	1	0	GL22232	+	exonic
GaLu96scf_2_728269_728479	2	9	0	0	0	0	0	GL27629	-	exonic
GaLu96scf_22_118538_118753	2	0	3	0	0	6	0	GL22968	+	exonic
GaLu96scf_22_715146_715511	1	9	0	0	0	0	0	GL27813	-	intronic
GaLu96scf_23_555457_555654	1	0	0	2	0	6	1	GL23085	-	intronic
GaLu96scf_26_449079_449496	2	0	9	0	0	0	0	GL23341	+	exonic
GaLu96scf_27_432715_433523	1	0	7	0	0	0	2	GL28055	+	exonic
GaLu96scf_27_464123_464435	1	0	9	0	0	0	0	GL23453	+	exonic
GaLu96scf_28_502114_502375	1	9	0	0	0	0	0	GL23507	+	exonic
GaLu96scf_3_1332003_1332585	4	0	6	3	0	0	0	GL16846	+	exonic
GaLu96scf_3_1674984_1675525	2	0	5	0	0	4	0	GL23899	+	exonic
GaLu96scf_3_1784058_1784342	1	7	0	0	2	0	0	GL16873	-	intronic
GaLu96scf_31_140867_141632	2	6	2	0	0	0	1	GL17001	-	exonic
GaLu96scf_33_12161_12892	2	0	9	0	0	0	0	GL24222	-	exonic
GaLu96scf_45_24180_24572	2	4	3	0	0	0	2	GL28663	+	exonic
GaLu96scf_5_516816_518001	2	0	9	0	0	0	0	GL17615	-	exonic
GaLu96scf_7_647515_647836	1	0	9	0	0	0	0	GL25610	-	exonic
GaLu96scf_9_1280373_1280724	2	0	8	1	0	0	0	GL26718	-	exonic
GaLu96scf_1_2168459_2168738	1	0	3	2	3	0	0	GL30250	-	exonic
GaLu96scf_1_3217665_3218130	2	5	0	0	0	0	3	GL30326	+	exonic
GaLu96scf_1_336402_336711	1	2	6	0	0	0	0	GL30301	-	exonic

GaLu96scf_1_3551960_3552335	3	0	5	0	3	0	0	GL30069	-	exonic
GaLu96scf_1_564557_564850	2	0	0	2	0	4	2	GL30398	-	exonic
GaLu96scf_1_613208_613932	2	0	4	0	0	2	2	GL29617	+	exonic
GaLu96scf_1_83631_83927	1	8	0	0	0	0	0	GL30422	-	exonic
GaLu96scf_10_738185_738462	2	8	0	0	0	0	0	GL20637	-	exonic
GaLu96scf_10_989288_989517	1	0	8	0	0	0	0	GL20653	-	exonic
GaLu96scf_11_132103_132409	1	1	7	0	0	0	0	GL20790	-	exonic
GaLu96scf_12_162281_162525	1	0	8	0	0	0	0	GL21029	-	exonic
GaLu96scf_13_661306_661700	3	0	0	8	0	0	0	GL21135	-	exonic
GaLu96scf_13_946765_947266	1	0	7	0	1	0	0	GL21157	-	exonic
GaLu96scf_15_578105_578307	1	0	8	0	0	0	0	GL21539	+	exonic
GaLu96scf_15_821086_822351	2	0	2	1	2	0	3	GL21515	-	exonic
GaLu96scf_15_889124_889844	2	0	7	0	1	0	0	GL21511	+	exonic
GaLu96scf_16_257810_258051	1	7	0	0	1	0	0	GL21632	-	exonic
GaLu96scf_17_924788_925156	1	8	0	0	0	0	0	GL21972	-	exonic
GaLu96scf_19_248473_248676	1	0	0	0	0	5	3	GL18854	+	exonic
GaLu96scf_19_259702_260237	3	0	8	0	0	0	0	GL15912	-	exonic
GaLu96scf_19_436708_437006	1	5	3	0	0	0	0	GL22199	+	exonic
GaLu96scf_2_2081953_2082257	2	0	8	0	0	0	0	GL22608	+	exonic
GaLu96scf_2_2081953_2082786	6	0	8	0	0	0	0	GL22608	+	exonic
GaLu96scf_20_272630_272957	1	3	1	0	0	4	0	GL22708	+	exonic
GaLu96scf_20_336853_337127	1	0	7	1	0	0	0	GL19024	-	exonic
GaLu96scf_21_26613_26895	1	0	8	0	0	0	0	GL22826	-	exonic
GaLu96scf_21_298566_298876	1	7	0	1	0	0	0	GL19097	-	exonic
GaLu96scf_24_18576_19388	3	0	7	0	0	0	1	GL23161	-	exonic

GaLu96scf_25_314155_314449	3	0	8	0	0	0	0	GL16604	-	exonic
GaLu96scf_26_449240_449496	1	0	8	0	0	0	0	GL23341	+	exonic
GaLu96scf_29_326308_326624	2	8	0	0	0	0	0	GL23585	+	exonic
GaLu96scf_3_51339_51710	2	0	8	0	0	0	0	GL16814	+	exonic
GaLu96scf_30_254564_256000	4	4	0	3	1	0	0	GL24029	-	exonic
GaLu96scf_31_36002_36247	1	8	0	0	0	0	0	GL24099	-	exonic
GaLu96scf_32_167826_168203	1	8	0	0	0	0	0	GL24162	+	exonic
GaLu96scf_32_29345_29713	2	2	5	0	1	0	0	GL28308	-	exonic
GaLu96scf_44_81238_81515	1	0	6	0	0	0	2	GL17453	+	intronic
GaLu96scf_46_78604_78861	1	1	0	7	0	0	0	GL25091	+	exonic
GaLu96scf_47_82969_83169	1	0	1	0	4	3	0	GL25104	+	exonic
GaLu96scf_5_1256744_1256931	1	0	0	8	0	0	0	GL28715	-	intronic
GaLu96scf_5_356471_356897	2	8	0	0	0	0	0	GL25417	-	exonic
GaLu96scf_5_985045_985501	2	8	0	0	0	0	0	GL25266	+	exonic
GaLu96scf_6_118733_118934	1	7	0	0	1	0	0	GL31183	-	intronic
GaLu96scf_7_1465011_1465205	1	0	0	0	0	8	0	GL28887	+	exonic
GaLu96scf_7_851952_852235	2	4	4	0	0	0	0	GL25671	-	exonic
GaLu96scf_8_1207123_1207684	1	0	0	8	0	0	0	GL20245	+	exonic
GaLu96scf_8_866919_867522	3	5	1	0	0	2	0	GL25925	+	exonic
GaLu96scf_8_867522_867712	1	0	6	0	0	2	0	GL25925	+	intronic
GaLu96scf_8_997116_997284	1	0	8	0	0	0	0	GL25950	+	intronic
GaLu96scf_9_1106364_1106552	1	6	0	0	0	2	0	GL26480	+	exonic
GaLu96scf_9_1524047_1524338	1	0	3	0	1	4	0	GL26740	-	exonic
GaLu96scf_9_729107_729718	2	0	0	2	3	0	3	GL26645	+	exonic
GaLu96scf_9_819996_820255	1	2	3	1	0	2	0	GL26562	+	exonic

GaLu96scf_1_1832393_1833145	5	0	7	0	0	0	0	GL30917	+	exonic
GaLu96scf_1_1853379_1853571	1	7	0	0	0	0	0	GL30222	+	exonic
GaLu96scf_1_2168323_2168738	2	0	7	0	0	0	0	GL30250	-	exonic
GaLu96scf_1_3294448_3295348	3	7	0	0	0	0	0	GL31004	+	exonic
GaLu96scf_1_336123_336402	1	0	7	0	0	0	0	GL30301	-	intronic
GaLu96scf_1_4029699_4029994	2	7	0	0	0	0	0	GL30772	+	exonic
GaLu96scf_1_4434778_4435327	4	0	0	0	0	7	0	GL29561	+	exonic
GaLu96scf_1_559682_559969	2	7	0	0	0	0	0	GL30394	+	exonic
GaLu96scf_1_84704_84952	1	0	7	0	0	0	0	GL30422	-	exonic
GaLu96scf_10_405028_405715	1	0	3	1	0	0	3	GL20491	+	intronic
GaLu96scf_10_548448_548906	1	0	7	0	0	0	0	GL15096	-	intronic
GaLu96scf_10_633434_633756	1	7	0	0	0	0	0	GL20627	+	intronic
GaLu96scf_10_989135_989288	1	0	0	3	3	0	1	GL20653	-	intronic
GaLu96scf_11_1160952_1161458	3	7	0	0	0	0	0	GL20742	+	exonic
GaLu96scf_11_433670_433872	1	0	7	0	0	0	0	GL20856	-	exonic
GaLu96scf_11_518310_518605	2	0	7	0	0	0	0	GL20761	-	exonic
GaLu96scf_11_798658_799212	3	0	0	0	0	7	0	GL20726	-	exonic
GaLu96scf_12_953525_953752	1	7	0	0	0	0	0	GL20961	+	exonic
GaLu96scf_13_1078212_1078536	1	0	1	5	0	1	0	GL21210	+	intronic
GaLu96scf_14_990763_990994	1	0	4	0	0	3	0	GL15529	-	intronic
GaLu96scf_15_824691_824989	3	0	7	0	0	0	0	GL21605	-	exonic
GaLu96scf_16_192216_192441	1	0	5	0	0	1	1	GL21626	+	intronic
GaLu96scf_16_369107_369503	2	5	0	0	0	1	1	GL21747	-	exonic
GaLu96scf_16_838615_839104	1	0	4	0	0	3	0	GL21786	+	intronic
GaLu96scf_16_856246_856605	2	0	0	5	0	2	0	GL15699	-	exonic

GaLu96scf_16_961743_962077	2	7	0	0	0	0	0	GL21717	-	exonic
GaLu96scf_17_28292_28622	1	4	0	0	1	1	1	GL21859	-	intronic
GaLu96scf_17_538649_539229	3	0	3	0	0	2	2	GL15763	-	exonic
GaLu96scf_17_630205_630493	1	0	4	0	0	0	3	GL21838	-	exonic
GaLu96scf_19_61276_61455	1	0	0	0	6	0	1	GL15899	-	intronic
GaLu96scf_19_870608_870940	2	0	5	0	0	2	0	GL22225	+	exonic
GaLu96scf_2_646494_646912	1	0	0	7	0	0	0	GL22426	-	intronic
GaLu96scf_20_142419_143504	3	0	7	0	0	0	0	GL22680	+	exonic
GaLu96scf_20_338537_338818	1	7	0	0	0	0	0	GL16218	+	intronic
GaLu96scf_20_411775_412087	1	0	7	0	0	0	0	GL22716	-	intronic
GaLu96scf_21_724122_724413	1	0	3	0	4	0	0	GL22820	-	intronic
GaLu96scf_22_188417_188634	1	4	3	0	0	0	0	GL22966	-	exonic
GaLu96scf_23_114407_115124	2	0	1	5	0	0	1	GL23036	-	exonic
GaLu96scf_23_535599_536301	3	0	6	0	0	0	1	GL27854	-	exonic
GaLu96scf_23_84546_84768	1	0	7	0	0	0	0	GL23073	+	exonic
GaLu96scf_26_531078_531338	1	0	0	0	0	5	2	GL23342	-	exonic
GaLu96scf_27_318775_318952	1	0	6	0	0	0	1	GL23418	-	exonic
GaLu96scf_27_366191_366606	4	0	7	0	0	0	0	GL23395	-	exonic
GaLu96scf_3_1179570_1180275	2	0	5	2	0	0	0	GL23655	-	exonic
GaLu96scf_3_1329424_1329647	1	0	6	0	0	1	0	GL19461	+	exonic
GaLu96scf_3_1898905_1899334	1	0	0	3	2	0	2	GL23921	+	intronic
GaLu96scf_3_1917278_1917511	2	7	0	0	0	0	0	GL23706	-	exonic
GaLu96scf_3_507235_507764	3	0	6	0	0	1	0	GL23732	+	exonic
GaLu96scf_30_191172_191529	1	0	4	0	2	1	0	GL24011	+	intronic
GaLu96scf_31_140867_141452	1	0	6	0	0	1	0	GL17001	-	exonic

GaLu96scf_31_259187_259684	2	0	2	2	2	1	0	GL28295	-	exonic
GaLu96scf_31_320750_320941	1	5	2	0	0	0	0	GL17009	+	exonic
GaLu96scf_32_314680_314960	1	0	2	0	0	5	0	GL24158	-	exonic
GaLu96scf_36_127166_127531	2	0	7	0	0	0	0	GL29251	-	exonic
GaLu96scf_37_224634_225370	2	0	0	2	1	0	4	GL24384	-	exonic
GaLu96scf_4_1459868_1460308	2	0	1	0	4	2	0	GL17270	+	exonic
GaLu96scf_4_168642_169400	3	5	0	0	0	0	2	GL28477	+	exonic
GaLu96scf_4_638508_638817	1	3	4	0	0	0	0	GL24575	+	exonic
GaLu96scf_4_692800_693247	2	7	0	0	0	0	0	GL24584	-	exonic
GaLu96scf_4_942652_942888	1	0	2	0	0	1	4	GL17366	+	exonic
GaLu96scf_4_948009_948194	1	3	3	1	0	0	0	GL24707	+	intronic
GaLu96scf_40_78231_78716	3	0	6	0	0	1	0	GL24887	-	exonic
GaLu96scf_42_145252_145819	3	0	4	1	2	0	0	GL17424	-	exonic
GaLu96scf_42_187462_188053	1	0	5	0	0	2	0	GL28618	+	exonic
GaLu96scf_46_79582_80089	1	6	0	0	0	1	0	GL25091	+	exonic
GaLu96scf_5_419746_420267	3	0	0	0	4	0	3	GL25223	+	exonic
GaLu96scf_5_420067_420267	1	0	0	0	0	6	1	GL25223	+	exonic
GaLu96scf_51_90105_90342	1	0	0	1	0	6	0	GL25489	+	exonic
GaLu96scf_6_1277664_1278485	3	0	0	2	5	0	0	GL31640	-	exonic
GaLu96scf_6_33894_34253	3	0	3	1	0	3	0	GL31133	+	exonic
GaLu96scf_7_1425181_1425884	2	0	7	0	0	0	0	GL25711	-	exonic
GaLu96scf_7_1425350_1425884	1	0	6	0	0	1	0	GL25711	-	exonic
GaLu96scf_8_460253_460763	3	3	3	1	0	0	0	GL26103	+	exonic
GaLu96scf_9_208429_208736	3	7	0	0	0	0	0	GL18143	+	exonic
GaLu96scf_9_517326_517556	1	7	0	0	0	0	0	GL26763	-	exonic

GaLu96scf_9_917450_917719	1	0	7	0	0	0	0	GL26657	+	exonic
GaLu96scf_9_958374_958775	3	0	7	0	0	0	0	GL18179	+	exonic
GaLu96scf_1_1186490_1186746	1	0	6	0	0	0	0	GL29333	+	exonic
GaLu96scf_1_1832017_1833145	7	0	1	1	0	2	2	GL30917	+	exonic
GaLu96scf_1_2047789_2047968	1	6	0	0	0	0	0	GL30239	+	intronic
GaLu96scf_1_2065870_2066251	1	0	6	0	0	0	0	GL30536	+	exonic
GaLu96scf_1_2460056_2460446	1	0	6	0	0	0	0	GL29985	-	exonic
GaLu96scf_1_2485437_2485732	1	0	6	0	0	0	0	GL29981	+	exonic
GaLu96scf_1_2496686_2497062	3	0	3	2	0	1	0	GL30578	+	exonic
GaLu96scf_1_2965726_2965968	1	0	0	0	1	4	1	GL30637	-	exonic
GaLu96scf_1_3142092_3142483	2	0	4	0	2	0	0	GL30662	+	exonic
GaLu96scf_1_441754_442359	2	0	0	0	5	1	0	GL30097	-	exonic
GaLu96scf_1_85022_85476	1	0	6	0	0	0	0	GL30422	-	exonic
GaLu96scf_1_89438_89611	1	0	0	1	0	5	0	GL30875	-	exonic
GaLu96scf_10_239610_240198	3	0	0	0	3	0	3	GL20604	+	exonic
GaLu96scf_10_57748_58352	4	2	4	0	0	0	0	GL15004	-	exonic
GaLu96scf_10_721197_721538	3	0	4	1	0	0	1	GL20553	-	exonic
GaLu96scf_11_1253258_1253721	1	0	6	0	0	0	0	GL20675	+	intronic
GaLu96scf_11_379444_380247	4	0	0	2	3	0	1	GL20847	-	exonic
GaLu96scf_11_550698_551236	2	0	6	0	0	0	0	GL20860	+	exonic
GaLu96scf_11_798428_798658	1	0	2	1	2	0	1	GL20726	-	intronic
GaLu96scf_12_716899_717730	2	6	0	0	0	0	0	GL21074	-	exonic
GaLu96scf_12_750458_750903	3	0	6	0	0	0	0	GL15327	-	exonic
GaLu96scf_13_1106880_1107339	1	0	6	0	0	0	0	GL21215	+	exonic
GaLu96scf_13_62664_63412	1	0	6	0	0	0	0	GL27080	+	exonic

GaLu96scf_13_70956_71305	1	0	6	0	0	0	0	GL21093	+	exonic
GaLu96scf_13_716319_716608	2	0	4	0	2	0	0	GL21141	-	exonic
GaLu96scf_13_786042_786430	3	6	0	0	0	0	0	GL21149	-	exonic
GaLu96scf_13_937874_938103	1	6	0	0	0	0	0	GL21156	-	exonic
GaLu96scf_14_299794_300343	1	0	2	0	4	0	0	GL21290	+	intronic
GaLu96scf_15_577955_578706	3	0	6	0	0	0	0	GL21539	+	exonic
GaLu96scf_15_583443_583687	1	0	4	0	0	1	1	GL31774	-	exonic
GaLu96scf_18_739989_740151	1	5	1	0	0	0	0	GL22032	-	intronic
GaLu96scf_2_1331117_1331659	3	0	6	0	0	0	0	GL22353	+	exonic
GaLu96scf_2_1541899_1542100	1	4	0	1	0	1	0	GL22569	+	exonic
GaLu96scf_2_775722_776014	1	0	6	0	0	0	0	GL18988	-	exonic
GaLu96scf_2_777117_778787	2	0	6	0	0	0	0	GL22662	+	exonic
GaLu96scf_20_411774_412087	1	0	0	6	0	0	0	GL22716	-	intronic
GaLu96scf_22_119730_120074	3	0	6	0	0	0	0	GL22968	+	exonic
GaLu96scf_23_309149_309310	1	6	0	0	0	0	0	GL23108	+	intronic
GaLu96scf_23_432457_432750	2	0	6	0	0	0	0	GL23057	+	exonic
GaLu96scf_24_34110_34346	1	5	0	0	0	1	0	GL23132	+	exonic
GaLu96scf_24_410506_410987	1	6	0	0	0	0	0	GL23153	-	intronic
GaLu96scf_25_479612_479927	2	0	0	0	0	5	1	GL23247	-	exonic
GaLu96scf_26_186682_187294	1	0	6	0	0	0	0	GL19316	+	exonic
GaLu96scf_26_559095_559469	1	0	3	1	0	0	2	GL23344	-	exonic
GaLu96scf_27_566829_567492	2	6	0	0	0	0	0	GL16715	+	exonic
GaLu96scf_28_108207_108560	2	0	6	0	0	0	0	GL23496	+	exonic
GaLu96scf_29_14026_14218	1	0	0	0	2	3	1	GL23544	+	intronic
GaLu96scf_3_1902628_1903523	4	0	4	0	0	1	1	GL16882	+	exonic

GaLu96scf_31_234899_235338	1	0	3	3	0	0	0	GL28294	+	exonic
GaLu96scf_31_249500_249822	3	6	0	0	0	0	0	GL24117	-	exonic
GaLu96scf_31_371585_371932	2	0	6	0	0	0	0	GL24092	-	exonic
GaLu96scf_32_131663_132039	1	0	0	6	0	0	0	GL24166	+	intronic
GaLu96scf_33_107500_108150	2	3	0	3	0	0	0	GL24214	-	exonic
GaLu96scf_33_113771_114519	1	0	6	0	0	0	0	GL24201	-	intronic
GaLu96scf_37_75073_75655	3	0	6	0	0	0	0	GL24358	+	exonic
GaLu96scf_4_119002_119433	3	6	0	0	0	0	0	GL24469	-	exonic
GaLu96scf_4_139725_139975	1	0	4	0	0	2	0	GL17243	-	exonic
GaLu96scf_4_1411238_1411498	1	0	6	0	0	0	0	GL24783	-	exonic
GaLu96scf_4_1470480_1471133	5	1	0	3	2	0	0	GL24779	+	exonic
GaLu96scf_4_1470917_1471133	1	4	2	0	0	0	0	GL24779	+	exonic
GaLu96scf_4_620240_620393	1	0	3	0	1	1	1	GL24851	+	exonic
GaLu96scf_4_692800_693127	1	3	3	0	0	0	0	GL24584	-	exonic
GaLu96scf_4_762139_762431	1	6	0	0	0	0	0	GL24863	-	intronic
GaLu96scf_4_942888_943040	1	3	1	0	2	0	0	GL17366	+	intronic
GaLu96scf_5_288195_288837	3	1	3	0	2	0	0	GL25300	+	exonic
GaLu96scf_5_801582_801954	1	0	0	0	6	0	0	GL25252	+	intronic
GaLu96scf_5_811092_811588	2	0	6	0	0	0	0	GL25253	+	exonic
GaLu96scf_50_82315_82708	2	0	6	0	0	0	0	GL25480	-	exonic
GaLu96scf_52_7460_8063	4	0	6	0	0	0	0	GL25501	-	exonic
GaLu96scf_6_1278284_1278485	1	0	3	1	0	2	0	GL31640	-	exonic
GaLu96scf_6_1347523_1348145	1	0	0	2	3	1	0	GL31148	-	exonic
GaLu96scf_6_217787_218029	1	0	6	0	0	0	0	GL31658	+	exonic
GaLu96scf_6_813635_813907	1	6	0	0	0	0	0	GL31615	-	exonic

GaLu96scf_6_871714_871951	2	6	0	0	0	0	0	GL31341	+	exonic
GaLu96scf_6_871714_872157	3	4	0	0	0	2	0	GL31341	+	exonic
GaLu96scf_7_206066_206470	2	0	3	0	3	0	0	GL28915	-	exonic
GaLu96scf_8_1061304_1061639	2	0	0	0	0	4	2	GL25824	-	exonic
GaLu96scf_8_1164705_1165005	1	0	6	0	0	0	0	GL20241	-	exonic
GaLu96scf_8_359260_361090	8	0	6	0	0	0	0	GL25880	+	exonic
GaLu96scf_8_463132_463471	1	6	0	0	0	0	0	GL25987	+	exonic
GaLu96scf_8_498907_499140	1	0	0	6	0	0	0	GL25895	+	exonic
GaLu96scf_8_997325_997534	1	0	5	0	0	1	0	GL25950	+	exonic
GaLu96scf_9_1185508_1185778	1	0	6	0	0	0	0	GL26704	+	exonic
GaLu96scf_9_468608_469135	2	0	6	0	0	0	0	GL26635	-	exonic
GaLu96scf_9_564822_565176	2	0	2	1	0	0	3	GL26766	-	exonic
GaLu96scf_9_672509_673108	3	0	6	0	0	0	0	GL20443	-	exonic
GaLu96scf_9_782358_782812	3	0	6	0	0	0	0	GL26651	-	exonic
GaLu96scf_9_864649_865380	5	4	0	0	0	0	2	GL26572	-	exonic
GaLu96scf_1_1350356_1350654	1	0	5	0	0	0	0	GL29865	+	exonic
GaLu96scf_1_1525668_1525994	1	1	1	0	3	0	0	GL30903	+	exonic
GaLu96scf_1_2114443_2114867	3	5	0	0	0	0	0	GL29950	-	exonic
GaLu96scf_1_2259921_2260257	2	0	4	1	0	0	0	GL30562	-	exonic
GaLu96scf_1_2406810_2407749	4	0	0	0	5	0	0	GL29974	+	exonic
GaLu96scf_1_3045559_3045913	3	0	5	0	0	0	0	GL30656	-	exonic
GaLu96scf_1_336121_336402	1	0	5	0	0	0	0	GL30301	-	intronic
GaLu96scf_1_343489_344528	5	5	0	0	0	0	0	GL30986	-	exonic
GaLu96scf_1_344308_344528	1	4	0	0	0	1	0	GL30986	-	exonic
GaLu96scf_1_3483992_3484226	1	0	5	0	0	0	0	GL30057	+	exonic

GaLu96scf_1_4117290_4117452	1	3	2	0	0	0	0	GL29535	+	intronic
GaLu96scf_1_4204512_4204829	1	0	5	0	0	0	0	GL30112	+	exonic
GaLu96scf_1_441144_441919	4	0	3	0	1	1	0	GL30097	-	exonic
GaLu96scf_1_4460049_4460531	4	0	5	0	0	0	0	GL30130	-	exonic
GaLu96scf_1_4599953_4601526	5	0	5	0	0	0	0	GL29585	+	exonic
GaLu96scf_1_88854_89101	1	0	0	5	0	0	0	GL30875	-	intronic
GaLu96scf_1_96079_96312	1	0	0	0	0	0	5	GL30174	+	intronic
GaLu96scf_10_1027170_1027578	1	0	5	0	0	0	0	GL15031	+	exonic
GaLu96scf_10_1240738_1241190	1	0	5	0	0	0	0	GL20473	-	intronic
GaLu96scf_11_1110999_1111405	3	5	0	0	0	0	0	GL20673	-	exonic
GaLu96scf_11_1234416_1234903	2	0	0	0	5	0	0	GL20816	-	exonic
GaLu96scf_11_1234719_1234903	1	5	0	0	0	0	0	GL20816	-	exonic
GaLu96scf_11_699350_699504	1	2	0	0	0	0	3	GL18364	+	exonic
GaLu96scf_11_86033_87347	3	0	5	0	0	0	0	GL20786	+	exonic
GaLu96scf_12_934652_935015	1	2	3	0	0	0	0	GL20966	-	intronic
GaLu96scf_13_1002778_1003234	3	0	5	0	0	0	0	GL21206	+	exonic
GaLu96scf_13_276282_276873	4	0	5	0	0	0	0	GL15391	+	exonic
GaLu96scf_13_302188_302428	1	0	0	0	5	0	0	GL21175	+	intronic
GaLu96scf_13_362795_363611	5	0	5	0	0	0	0	GL21117	+	exonic
GaLu96scf_15_312810_313365	2	0	5	0	0	0	0	GL21534	-	exonic
GaLu96scf_15_561186_561534	1	0	5	0	0	0	0	GL18632	+	exonic
GaLu96scf_15_821086_821358	1	0	5	0	0	0	0	GL21515	-	exonic
GaLu96scf_16_216972_217600	3	0	5	0	0	0	0	GL21685	-	exonic
GaLu96scf_16_26866_27197	1	4	0	0	1	0	0	GL27278	+	exonic
GaLu96scf_16_825491_825940	2	5	0	0	0	0	0	GL18716	+	exonic

GaLu96scf_16_916727_917143	3	5	0	0	0	0	0	GL21669	+	exonic
GaLu96scf_17_265349_265533	1	0	5	0	0	0	0	GL18743	-	exonic
GaLu96scf_18_532142_532405	1	0	0	0	0	5	0	GL22017	-	exonic
GaLu96scf_18_567176_567508	2	0	3	1	1	0	0	GL27401	-	exonic
GaLu96scf_19_259914_260237	2	0	5	0	0	0	0	GL15912	-	exonic
GaLu96scf_19_528439_528683	1	0	0	0	0	5	0	GL22203	-	exonic
GaLu96scf_19_585745_586100	2	0	5	0	0	0	0	GL18873	+	exonic
GaLu96scf_19_819546_819912	2	0	2	0	3	0	0	GL22176	+	exonic
GaLu96scf_2_1056816_1057450	3	0	5	0	0	0	0	GL22329	-	exonic
GaLu96scf_2_1578873_1579051	1	0	5	0	0	0	0	GL22574	-	intronic
GaLu96scf_2_1629480_1629720	1	0	5	0	0	0	0	GL22368	-	exonic
GaLu96scf_2_807888_808986	2	0	5	0	0	0	0	GL22664	+	exonic
GaLu96scf_21_737969_738131	1	0	4	0	0	0	1	GL19109	+	exonic
GaLu96scf_22_759446_759774	2	0	5	0	0	0	0	GL22951	+	exonic
GaLu96scf_23_302320_302518	1	0	0	0	3	2	0	GL23051	+	intronic
GaLu96scf_23_532213_532829	2	0	3	0	0	2	0	GL27852	-	exonic
GaLu96scf_24_642832_643626	2	3	2	0	0	0	0	GL23172	-	exonic
GaLu96scf_24_643047_643346	1	0	5	0	0	0	0	GL23172	-	intronic
GaLu96scf_25_389997_390561	1	0	0	0	5	0	0	GL23279	+	exonic
GaLu96scf_26_126835_127042	2	5	0	0	0	0	0	GL23299	+	exonic
GaLu96scf_27_499582_499912	1	0	5	0	0	0	0	GL23465	-	exonic
GaLu96scf_27_564880_565267	2	0	0	5	0	0	0	GL23463	+	exonic
GaLu96scf_28_510504_510671	1	0	5	0	0	0	0	GL23508	+	intronic
GaLu96scf_28_525456_526191	4	0	5	0	0	0	0	GL23538	+	exonic
GaLu96scf_29_105018_105403	1	0	3	0	2	0	0	GL23599	+	intronic

GaLu96scf_3_1029170_1029354	1	0	0	0	0	5	0	GL23865	+	intronic
GaLu96scf_3_1674100_1674319	1	0	2	3	0	0	0	GL23899	+	intronic
GaLu96scf_3_416325_416483	1	4	0	0	1	0	0	GL23939	-	intronic
GaLu96scf_3_507235_507497	2	0	5	0	0	0	0	GL23732	+	exonic
GaLu96scf_3_637440_638171	3	0	0	5	0	0	0	GL23952	-	exonic
GaLu96scf_32_174746_175093	2	0	5	0	0	0	0	GL24139	+	exonic
GaLu96scf_32_267595_268008	1	0	0	5	0	0	0	GL24187	-	intronic
GaLu96scf_36_88635_89008	2	0	5	0	0	0	0	GL29224	-	exonic
GaLu96scf_36_89918_90195	2	0	5	0	0	0	0	GL29224	-	exonic
GaLu96scf_36_97718_98460	4	0	5	0	0	0	0	GL29258	+	exonic
GaLu96scf_4_169285_169975	2	0	0	0	3	2	0	GL28477	+	exonic
GaLu96scf_4_581267_581650	3	0	5	0	0	0	0	GL24567	+	exonic
GaLu96scf_4_619131_620137	4	0	5	0	0	0	0	GL24851	+	exonic
GaLu96scf_4_82007_82257	1	5	0	0	0	0	0	GL17240	-	exonic
GaLu96scf_4_869298_869525	1	0	5	0	0	0	0	GL24605	-	exonic
GaLu96scf_40_120652_120959	1	0	0	2	2	0	1	GL24914	+	exonic
GaLu96scf_40_145087_145321	1	3	0	0	0	2	0	GL17382	-	exonic
GaLu96scf_43_25907_26321	2	3	2	0	0	0	0	GL25003	+	exonic
GaLu96scf_45_47325_47496	1	0	5	0	0	0	0	GL28656	-	exonic
GaLu96scf_47_37444_38068	2	0	4	0	0	0	1	GL28679	-	exonic
GaLu96scf_5_1092795_1093112	3	5	0	0	0	0	0	GL25357	-	exonic
GaLu96scf_5_11814_12136	2	0	5	0	0	0	0	GL25146	+	exonic
GaLu96scf_5_13170_13484	2	0	5	0	0	0	0	GL25146	+	exonic
GaLu96scf_5_596965_597421	2	0	5	0	0	0	0	GL17626	+	exonic
GaLu96scf_5_683996_684442	3	0	5	0	0	0	0	GL17625	-	exonic

GaLu96scf_50_72806_73658	5	0	4	1	0	0	0	GL28773	+	exonic
GaLu96scf_6_124526_124939	1	0	5	0	0	0	0	GL31371	-	exonic
GaLu96scf_6_1450153_1450393	2	0	5	0	0	0	0	GL31536	+	exonic
GaLu96scf_6_306628_307187	2	0	0	1	0	4	0	GL31411	+	exonic
GaLu96scf_6_55244_55589	3	0	0	0	2	0	3	GL31471	-	exonic
GaLu96scf_6_569531_569725	1	0	4	1	0	0	0	GL31314	-	exonic
GaLu96scf_7_207334_207925	2	0	0	0	5	0	0	GL25646	+	exonic
GaLu96scf_7_509985_510258	1	0	0	0	0	5	0	GL25588	+	intronic
GaLu96scf_7_644305_645029	3	0	0	0	5	0	0	GL25664	-	exonic
GaLu96scf_7_813730_814074	1	0	0	0	0	5	0	GL25772	-	intronic
GaLu96scf_7_932083_932292	2	4	0	0	0	0	1	GL25780	+	exonic
GaLu96scf_8_1061304_1061968	3	0	0	0	0	5	0	GL25824	-	exonic
GaLu96scf_8_1085842_1086215	2	0	5	0	0	0	0	GL25820	+	exonic
GaLu96scf_8_1095720_1096046	1	5	0	0	0	0	0	GL25821	+	intronic
GaLu96scf_8_1226373_1226573	1	0	5	0	0	0	0	GL25958	-	exonic
GaLu96scf_8_1303781_1304246	1	0	5	0	0	0	0	GL25853	-	exonic
GaLu96scf_8_1384261_1384494	1	5	0	0	0	0	0	GL25850	+	exonic
GaLu96scf_8_1393722_1393919	1	0	5	0	0	0	0	GL25852	+	exonic
GaLu96scf_8_541961_542452	2	5	0	0	0	0	0	GL17944	-	exonic
GaLu96scf_8_58909_59252	1	0	0	0	0	5	0	GL25940	-	exonic
GaLu96scf_8_68809_69109	2	0	5	0	0	0	0	GL26027	-	exonic
GaLu96scf_8_779487_779954	2	4	0	1	0	0	0	GL26008	+	exonic
GaLu96scf_9_1031117_1031333	2	0	0	0	0	5	0	GL26688	+	exonic
GaLu96scf_9_1059347_1059821	1	0	1	0	0	0	4	GL18105	+	exonic
GaLu96scf_9_1475386_1476375	2	0	5	0	0	0	0	GL26735	-	exonic

GaLu96scf_9_636082_636429	2	0	4	0	0	0	1	GL26775	-	exonic
GaLu96scf_9_820471_820700	2	5	0	0	0	0	0	GL26562	+	exonic
GaLu96scf_9_841950_842346	3	0	0	0	5	0	0	GL26791	+	exonic
GaLu96scf_1_1060364_1060682	1	0	4	0	0	0	0	GL30434	-	intronic
GaLu96scf_1_1349897_1350654	4	0	3	0	0	1	0	GL29865	+	exonic
GaLu96scf_1_1351823_1352120	1	4	0	0	0	0	0	GL29346	+	exonic
GaLu96scf_1_176092_176863	1	0	4	0	0	0	0	GL29315	+	exonic
GaLu96scf_1_2175889_2176296	2	4	0	0	0	0	0	GL29394	-	exonic
GaLu96scf_1_2297866_2298350	4	0	3	0	0	1	0	GL29968	-	exonic
GaLu96scf_1_2658853_2659140	1	0	0	0	4	0	0	GL29411	-	intronic
GaLu96scf_1_2837166_2837435	1	0	4	0	0	0	0	GL29731	+	exonic
GaLu96scf_1_3063259_3063541	2	4	0	0	0	0	0	GL30650	+	exonic
GaLu96scf_1_3942029_3943106	4	0	4	0	0	0	0	GL30362	+	exonic
GaLu96scf_1_4021255_4021597	2	3	0	0	0	1	0	GL30770	+	exonic
GaLu96scf_1_407026_407897	5	0	0	0	4	0	0	GL30095	-	exonic
GaLu96scf_1_4341800_4342887	3	0	0	0	0	0	4	GL30122	-	exonic
GaLu96scf_1_4533060_4533858	4	0	0	4	0	0	0	GL29573	-	exonic
GaLu96scf_1_4601594_4601991	3	4	0	0	0	0	0	GL29585	+	exonic
GaLu96scf_1_4641233_4642054	3	0	4	0	0	0	0	GL29575	+	exonic
GaLu96scf_1_613208_613436	1	0	4	0	0	0	0	GL29617	+	exonic
GaLu96scf_10_1255309_1255563	1	0	0	4	0	0	0	GL20535	-	exonic
GaLu96scf_10_1301597_1301961	2	0	0	1	0	0	3	GL15046	-	exonic
GaLu96scf_10_56926_57614	4	4	0	0	0	0	0	GL15004	-	exonic
GaLu96scf_10_988973_989517	2	4	0	0	0	0	0	GL20653	-	exonic
GaLu96scf_11_611687_612565	4	0	0	0	4	0	0	GL20868	-	exonic

GaLu96scf_11_730171_730390	1	3	1	0	0	0	0	GL20772	-	exonic
GaLu96scf_11_730656_730809	1	0	0	3	0	1	0	GL20772	-	intronic
GaLu96scf_12_358451_358606	1	4	0	0	0	0	0	GL27036	+	exonic
GaLu96scf_12_358451_360530	5	0	4	0	0	0	0	GL27036	+	exonic
GaLu96scf_12_449898_451254	2	0	3	1	0	0	0	GL18437	-	exonic
GaLu96scf_12_671396_671891	2	0	0	4	0	0	0	GL21068	-	exonic
GaLu96scf_12_793839_794091	2	0	4	0	0	0	0	GL20947	-	exonic
GaLu96scf_12_953752_953928	1	0	0	3	0	0	1	GL20961	+	intronic
GaLu96scf_13_1074725_1075413	2	0	1	0	0	0	3	GL21210	+	exonic
GaLu96scf_13_400014_400571	3	0	0	0	0	0	4	GL15415	-	exonic
GaLu96scf_13_63531_64301	2	4	0	0	0	0	0	GL27080	+	exonic
GaLu96scf_13_66003_67136	3	0	4	0	0	0	0	GL21093	+	exonic
GaLu96scf_13_711667_712174	3	0	0	2	0	2	0	GL15437	+	exonic
GaLu96scf_14_989687_990323	4	0	4	0	0	0	0	GL15529	-	exonic
GaLu96scf_15_528366_528683	1	0	4	0	0	0	0	GL21580	+	intronic
GaLu96scf_15_572974_573315	1	0	4	0	0	0	0	GL21586	-	exonic
GaLu96scf_15_791486_791962	2	0	1	1	2	0	0	GL21542	+	exonic
GaLu96scf_15_825259_825463	1	0	0	0	0	4	0	GL21605	-	exonic
GaLu96scf_16_691593_692107	2	0	4	0	0	0	0	GL21652	+	exonic
GaLu96scf_16_915422_916641	4	0	4	0	0	0	0	GL21669	+	exonic
GaLu96scf_16_975346_975747	2	0	0	0	3	0	1	GL21673	+	exonic
GaLu96scf_17_336566_336730	1	0	4	0	0	0	0	GL21918	+	intronic
GaLu96scf_17_783119_783494	2	0	0	4	0	0	0	GL27365	-	exonic
GaLu96scf_18_187339_187580	1	0	4	0	0	0	0	GL21984	+	exonic
GaLu96scf_18_218203_218560	2	0	0	0	0	0	4	GL22047	-	exonic

GaLu96scf_18_227010_227249	1	0	4	0	0	0	0	GL21985	-	intronic
GaLu96scf_18_447013_448372	3	4	0	0	0	0	0	GL22059	-	exonic
GaLu96scf_18_554297_554670	1	0	0	3	1	0	0	GL22019	-	exonic
GaLu96scf_18_567602_568351	2	0	2	0	0	0	2	GL27401	-	exonic
GaLu96scf_18_621423_621638	1	0	4	0	0	0	0	GL22024	-	intronic
GaLu96scf_19_735126_735759	4	0	4	0	0	0	0	GL22224	+	exonic
GaLu96scf_2_1186191_1186600	2	0	0	1	3	0	0	GL15997	-	exonic
GaLu96scf_2_1262267_1262558	3	0	2	0	0	2	0	GL22343	+	exonic
GaLu96scf_2_1543633_1544313	2	0	4	0	0	0	0	GL22569	+	exonic
GaLu96scf_2_1569479_1569709	1	0	0	0	3	1	0	GL22365	-	intronic
GaLu96scf_2_1700186_1700778	3	0	4	0	0	0	0	GL22372	+	exonic
GaLu96scf_2_1700392_1700778	2	4	0	0	0	0	0	GL22372	+	exonic
GaLu96scf_2_176558_177035	3	0	0	2	0	0	2	GL15974	+	exonic
GaLu96scf_2_2068219_2068519	2	0	2	2	0	0	0	GL16098	+	exonic
GaLu96scf_2_2191183_2191516	1	0	4	0	0	0	0	GL22621	-	intronic
GaLu96scf_2_286720_287175	3	0	0	0	1	3	0	GL22603	-	exonic
GaLu96scf_2_314019_314427	2	0	4	0	0	0	0	GL22634	+	exonic
GaLu96scf_2_519187_519731	2	0	0	0	4	0	0	GL22417	+	exonic
GaLu96scf_2_575900_576673	1	0	0	0	0	4	0	GL22647	+	intronic
GaLu96scf_2_576687_577621	2	0	0	0	0	0	4	GL22647	+	exonic
GaLu96scf_2_820970_821844	2	0	4	0	0	0	0	GL22666	+	exonic
GaLu96scf_2_893378_893769	1	0	0	0	0	2	2	GL22528	-	intronic
GaLu96scf_20_607683_608420	2	4	0	0	0	0	0	GL16249	-	exonic
GaLu96scf_21_557606_557784	1	3	1	0	0	0	0	GL22848	-	intronic
GaLu96scf_22_188208_188417	1	4	0	0	0	0	0	GL22966	-	intronic

GaLu96scf_22_93007_93254	1	0	0	0	0	4	0	GL22909	+	exonic
GaLu96scf_23_307567_307911	2	0	0	0	1	3	0	GL23110	-	exonic
GaLu96scf_23_532672_532829	1	0	4	0	0	0	0	GL27852	-	exonic
GaLu96scf_23_56480_56851	2	0	4	0	0	0	0	GL19197	+	exonic
GaLu96scf_24_43838_44290	2	0	0	0	4	0	0	GL23173	-	exonic
GaLu96scf_25_135198_135487	1	2	0	0	2	0	0	GL23260	+	exonic
GaLu96scf_25_304321_304541	1	1	3	0	0	0	0	GL23242	+	exonic
GaLu96scf_25_328978_329467	5	0	0	0	0	0	4	GL16593	+	exonic
GaLu96scf_25_397276_397957	1	0	4	0	0	0	0	GL16611	-	exonic
GaLu96scf_25_456468_456894	1	4	0	0	0	0	0	GL27956	+	exonic
GaLu96scf_25_72482_72755	2	0	2	0	0	2	0	GL23234	-	exonic
GaLu96scf_26_140017_140541	2	0	0	1	2	1	0	GL23348	+	exonic
GaLu96scf_26_261993_262152	1	0	0	2	2	0	0	GL23358	+	intronic
GaLu96scf_27_193935_194803	1	0	4	0	0	0	0	GL16691	-	exonic
GaLu96scf_27_225254_225551	1	4	0	0	0	0	0	GL23386	+	exonic
GaLu96scf_28_28229_28835	5	0	4	0	0	0	0	GL28065	+	exonic
GaLu96scf_28_430755_431088	2	4	0	0	0	0	0	GL23488	-	exonic
GaLu96scf_29_261362_261634	1	0	0	0	2	0	2	GL23582	+	intronic
GaLu96scf_29_265965_266260	1	0	0	0	3	0	1	GL23561	+	intronic
GaLu96scf_29_76196_76542	1	0	4	0	0	0	0	GL23578	-	exonic
GaLu96scf_29_94299_94790	2	0	0	0	4	0	0	GL16778	+	exonic
GaLu96scf_3_1028636_1029170	1	0	4	0	0	0	0	GL23865	+	exonic
GaLu96scf_3_1035453_1035907	2	3	1	0	0	0	0	GL23866	+	exonic
GaLu96scf_3_1227678_1227993	2	0	4	0	0	0	0	GL23661	-	exonic
GaLu96scf_3_1236789_1237003	1	0	0	0	0	4	0	GL16835	-	exonic

GaLu96scf_3_1260528_1260857	1	0	0	0	4	0	0	GL23882	-	intronic
GaLu96scf_3_1527434_1527691	1	4	0	0	0	0	0	GL23674	+	exonic
GaLu96scf_3_495263_495826	4	0	0	0	0	4	0	GL23731	-	exonic
GaLu96scf_3_716846_717535	3	4	0	0	0	0	0	GL28217	+	exonic
GaLu96scf_3_824291_824681	3	0	2	0	2	0	0	GL23770	-	exonic
GaLu96scf_30_123774_124031	1	0	4	0	0	0	0	GL19568	+	intronic
GaLu96scf_30_58310_58623	2	0	3	0	0	1	0	GL24022	-	exonic
GaLu96scf_31_171102_171522	1	0	0	2	1	0	1	GL24111	-	intronic
GaLu96scf_31_424050_424833	3	4	0	0	0	0	0	GL28302	-	exonic
GaLu96scf_31_70509_70945	3	4	0	0	0	0	0	GL24040	-	exonic
GaLu96scf_32_30470_30827	1	2	2	0	0	0	0	GL28308	-	exonic
GaLu96scf_33_180613_181069	3	2	2	0	0	0	0	GL24232	-	exonic
GaLu96scf_35_345181_345350	1	4	0	0	0	0	0	GL24321	+	intronic
GaLu96scf_36_11891_12080	2	4	0	0	0	0	0	GL29265	-	exonic
GaLu96scf_37_231727_232296	5	0	4	0	0	0	0	GL17179	-	exonic
GaLu96scf_4_1167577_1167835	1	0	4	0	0	0	0	GL24638	+	exonic
GaLu96scf_4_1181547_1181873	2	0	0	0	0	4	0	GL19765	+	exonic
GaLu96scf_4_121367_121788	1	0	4	0	0	0	0	GL24469	-	exonic
GaLu96scf_4_168137_168467	1	0	0	0	4	0	0	GL28477	+	intronic
GaLu96scf_4_1819482_1819795	2	4	0	0	0	0	0	GL24532	-	exonic
GaLu96scf_4_1884292_1884883	3	0	0	1	3	0	0	GL24531	+	exonic
GaLu96scf_4_546220_546606	1	0	4	0	0	0	0	GL24681	-	exonic
GaLu96scf_4_619799_620137	1	0	4	0	0	0	0	GL24851	+	exonic
GaLu96scf_40_201337_201609	1	0	4	0	0	0	0	GL24905	-	exonic
GaLu96scf_41_18642_18834	1	3	0	0	0	0	1	GL24922	+	intronic

GaLu96scf_41_74161_74429	1	4	0	0	0	0	0	GL24933	-	exonic
GaLu96scf_41_87866_88462	1	0	4	0	0	0	0	GL19845	+	exonic
GaLu96scf_42_144909_145252	1	4	0	0	0	0	0	GL17424	-	intronic
GaLu96scf_47_133994_134511	3	0	4	0	0	0	0	GL19904	+	exonic
GaLu96scf_49_51185_51461	2	0	4	0	0	0	0	GL25138	-	exonic
GaLu96scf_5_1064257_1064555	2	0	0	4	0	0	0	GL25169	-	exonic
GaLu96scf_5_11612_12136	3	1	3	0	0	0	0	GL25146	+	exonic
GaLu96scf_5_303925_304789	2	0	4	0	0	0	0	GL17594	-	exonic
GaLu96scf_5_41260_41857	3	0	4	0	0	0	0	GL25149	+	exonic
GaLu96scf_5_681237_681784	2	0	0	0	2	2	0	GL17624	-	exonic
GaLu96scf_5_76587_76933	2	0	0	0	0	0	4	GL17531	-	exonic
GaLu96scf_5_825181_825654	3	0	4	0	0	0	0	GL25254	+	exonic
GaLu96scf_6_392502_393291	3	0	0	0	4	0	0	GL31574	-	exonic
GaLu96scf_6_411931_412372	3	0	4	0	0	0	0	GL31296	+	exonic
GaLu96scf_6_565494_565785	2	0	4	0	0	0	0	GL31431	+	exonic
GaLu96scf_6_796087_796530	2	4	0	0	0	0	0	GL31330	+	exonic
GaLu96scf_7_1167567_1168333	3	0	0	4	0	0	0	GL25544	+	exonic
GaLu96scf_7_1311481_1311744	1	0	0	1	0	3	0	GL17766	+	intronic
GaLu96scf_7_1425350_1426168	3	3	1	0	0	0	0	GL25711	-	exonic
GaLu96scf_7_443074_443459	3	1	0	0	2	0	1	GL25738	+	exonic
GaLu96scf_7_454962_455834	3	0	4	0	0	0	0	GL25740	+	exonic
GaLu96scf_7_805388_806083	3	0	4	0	0	0	0	GL28943	-	exonic
GaLu96scf_7_847536_848067	3	0	4	0	0	0	0	GL25620	+	exonic
GaLu96scf_7_926139_926753	4	0	0	1	1	1	1	GL25783	-	exonic
GaLu96scf_8_730438_730594	1	0	0	0	4	0	0	GL25920	-	intronic

GaLu96scf_8_780301_780960	3	0	4	0	0	0	0	GL26008	+	exonic
GaLu96scf_8_867730_868021	1	0	0	4	0	0	0	GL25925	+	exonic
GaLu96scf_9_1021257_1021539	1	0	2	2	0	0	0	GL26687	+	exonic
GaLu96scf_9_1048703_1049550	5	0	0	0	3	1	0	GL26471	+	exonic
GaLu96scf_9_1062767_1063289	2	4	0	0	0	0	0	GL18105	+	exonic
GaLu96scf_9_1500539_1500724	1	0	4	0	0	0	0	GL26736	+	exonic
GaLu96scf_9_1508935_1509106	1	0	4	0	0	0	0	GL26739	-	exonic
GaLu96scf_9_472840_473204	1	0	0	0	0	0	4	GL26537	-	intronic
GaLu96scf_1_1050652_1050867	1	0	3	0	0	0	0	GL29848	-	exonic
GaLu96scf_1_1104880_1106528	4	0	3	0	0	0	0	GL29649	-	exonic
GaLu96scf_1_1138269_1138809	2	0	0	0	3	0	0	GL30182	-	exonic
GaLu96scf_1_1288668_1289038	2	0	0	0	3	0	0	GL29858	+	exonic
GaLu96scf_1_1779304_1779570	1	0	0	0	2	1	0	GL29682	+	exonic
GaLu96scf_1_1969800_1970472	3	0	3	0	0	0	0	GL30518	-	exonic
GaLu96scf_1_2047789_2047965	1	0	3	0	0	0	0	GL30239	+	intronic
GaLu96scf_1_2106788_2107758	1	0	1	2	0	0	0	GL29700	+	exonic
GaLu96scf_1_2162378_2163025	3	0	3	0	0	0	0	GL29391	-	exonic
GaLu96scf_1_2190249_2190615	1	0	0	0	0	3	0	GL30545	+	exonic
GaLu96scf_1_223809_224453	3	2	1	0	0	0	0	GL29930	+	exonic
GaLu96scf_1_2297344_2297727	2	0	0	0	3	0	0	GL29968	-	exonic
GaLu96scf_1_2412502_2412869	3	0	0	0	0	3	0	GL30579	-	exonic
GaLu96scf_1_2789441_2790043	2	3	0	0	0	0	0	GL30614	-	exonic
GaLu96scf_1_3211470_3211751	1	0	0	3	0	0	0	GL30041	+	intronic
GaLu96scf_1_3246287_3246766	3	0	0	0	0	3	0	GL30046	-	exonic
GaLu96scf_1_3383244_3383417	1	3	0	0	0	0	0	GL29474	+	exonic

GaLu96scf_1_344052_344528	3	0	0	0	0	3	0	GL30986	-	exonic
GaLu96scf_1_3475609_3475899	2	0	0	0	0	3	0	GL30055	+	exonic
GaLu96scf_1_3605457_3605978	3	0	0	0	0	0	3	GL29485	+	exonic
GaLu96scf_1_3643462_3643907	3	0	3	0	0	0	0	GL30079	-	exonic
GaLu96scf_1_3884241_3884749	1	0	2	1	0	0	0	GL29497	-	exonic
GaLu96scf_1_407026_407697	3	3	0	0	0	0	0	GL30095	-	exonic
GaLu96scf_1_4323598_4323938	1	0	0	0	0	3	0	GL30121	-	intronic
GaLu96scf_1_4341800_4342106	1	0	3	0	0	0	0	GL30122	-	exonic
GaLu96scf_1_4348141_4348941	6	0	0	0	0	3	0	GL30119	+	exonic
GaLu96scf_1_441754_442564	3	0	3	0	0	0	0	GL30097	-	exonic
GaLu96scf_1_4434778_4435080	3	0	0	0	0	0	3	GL29561	+	exonic
GaLu96scf_1_4533308_4533858	3	0	0	0	3	0	0	GL29573	-	exonic
GaLu96scf_1_823963_824340	1	0	0	0	0	0	3	GL29633	-	exonic
GaLu96scf_1_823963_824808	3	0	0	0	0	0	3	GL29633	-	exonic
GaLu96scf_1_879405_879566	1	0	0	1	1	0	1	GL30855	+	intronic
GaLu96scf_1_94170_94407	1	0	0	2	0	0	1	GL29306	+	exonic
GaLu96scf_10_1402095_1402499	2	0	3	0	0	0	0	GL20481	+	exonic
GaLu96scf_10_552706_553346	2	0	2	1	0	0	0	GL18259	+	exonic
GaLu96scf_10_61699_61980	1	0	3	0	0	0	0	GL15005	-	exonic
GaLu96scf_10_761382_761678	2	0	3	0	0	0	0	GL20638	-	exonic
GaLu96scf_10_955144_955411	2	2	0	1	0	0	0	GL20648	+	exonic
GaLu96scf_10_984456_984875	2	0	0	3	0	0	0	GL20650	+	exonic
GaLu96scf_11_100041_101610	6	0	3	0	0	0	0	GL20660	+	exonic
GaLu96scf_11_1152744_1153012	1	0	0	0	3	0	0	GL20745	-	intronic
GaLu96scf_11_1152745_1153012	1	0	0	0	3	0	0	GL20745	-	intronic

GaLu96scf_11_1208461_1208707	1	0	0	0	0	3	0	GL20810	+	exonic
GaLu96scf_11_1234173_1234903	3	0	3	0	0	0	0	GL20816	-	exonic
GaLu96scf_11_1307794_1308186	1	0	3	0	0	0	0	GL20825	-	intronic
GaLu96scf_11_1334669_1335872	2	0	3	0	0	0	0	GL20680	+	exonic
GaLu96scf_11_184938_185819	3	0	3	0	0	0	0	GL20738	+	exonic
GaLu96scf_11_377445_377622	1	0	3	0	0	0	0	GL20754	+	exonic
GaLu96scf_11_483461_483693	1	0	3	0	0	0	0	GL20854	+	exonic
GaLu96scf_11_518070_518413	2	0	0	0	3	0	0	GL20761	-	exonic
GaLu96scf_11_708674_708916	1	0	0	0	2	1	0	GL20717	-	exonic
GaLu96scf_11_714664_715339	3	0	1	0	2	0	0	GL20771	-	exonic
GaLu96scf_11_798426_798658	1	0	3	0	0	0	0	GL20726	-	intronic
GaLu96scf_11_799012_799212	2	0	3	0	0	0	0	GL20726	-	exonic
GaLu96scf_12_149041_150539	2	0	3	0	0	0	0	GL21027	-	exonic
GaLu96scf_12_263165_263451	1	0	0	0	0	3	0	GL20989	+	exonic
GaLu96scf_12_703142_703558	1	0	0	0	0	0	3	GL21069	+	exonic
GaLu96scf_12_922271_922427	1	3	0	0	0	0	0	GL20960	+	intronic
GaLu96scf_13_1074454_1074621	1	0	0	2	1	0	0	GL21210	+	intronic
GaLu96scf_13_1154821_1155201	1	0	0	0	0	3	0	GL21217	-	intronic
GaLu96scf_13_404666_405078	2	0	0	3	0	0	0	GL21225	+	exonic
GaLu96scf_13_436487_437312	3	0	3	0	0	0	0	GL21226	+	exonic
GaLu96scf_13_452689_453120	1	0	0	0	0	0	3	GL21238	-	exonic
GaLu96scf_13_961762_961992	1	0	0	0	0	0	3	GL21269	-	exonic
GaLu96scf_14_1069559_1069812	1	0	0	3	0	0	0	GL21282	+	exonic
GaLu96scf_14_380775_381156	2	0	0	0	1	2	0	GL21406	+	exonic
GaLu96scf_14_406887_407305	3	0	1	0	0	2	0	GL21413	+	exonic

GaLu96scf_14_585671_586031	2	0	0	0	0	0	3	GL21354	-	exonic
GaLu96scf_14_639562_639865	1	0	0	1	0	2	0	GL21358	+	intronic
GaLu96scf_14_737539_737858	2	0	0	0	0	3	0	GL21361	+	exonic
GaLu96scf_14_819657_819979	1	0	3	0	0	0	0	GL21448	-	exonic
GaLu96scf_15_1001650_1002246	3	0	3	0	0	0	0	GL21469	-	exonic
GaLu96scf_15_32320_32643	1	0	2	0	1	0	0	GL21460	+	exonic
GaLu96scf_15_577800_578706	4	0	3	0	0	0	0	GL21539	+	exonic
GaLu96scf_15_714949_715461	1	0	2	0	0	0	1	GL21598	-	intronic
GaLu96scf_15_845785_846042	2	0	0	0	3	0	0	GL21516	-	exonic
GaLu96scf_16_207191_207724	1	0	3	0	0	0	0	GL21684	-	intronic
GaLu96scf_16_219107_219318	1	0	2	1	0	0	0	GL15663	+	exonic
GaLu96scf_16_25083_25432	1	0	3	0	0	0	0	GL18668	-	exonic
GaLu96scf_16_275739_276095	3	0	0	0	3	0	0	GL21628	+	exonic
GaLu96scf_16_577479_577728	1	0	0	0	0	3	0	GL15678	-	intronic
GaLu96scf_16_662612_663103	3	3	0	0	0	0	0	GL21773	-	exonic
GaLu96scf_16_681976_682687	2	0	3	0	0	0	0	GL21775	-	exonic
GaLu96scf_16_691376_691569	1	3	0	0	0	0	0	GL21652	+	intronic
GaLu96scf_16_821330_822094	3	0	3	0	0	0	0	GL21791	-	exonic
GaLu96scf_16_863928_864218	2	0	0	0	0	3	0	GL21664	+	exonic
GaLu96scf_17_24055_24231	1	0	1	1	0	1	0	GL27324	-	exonic
GaLu96scf_17_302614_303226	4	0	0	3	0	0	0	GL21921	-	exonic
GaLu96scf_17_309808_310315	3	0	3	0	0	0	0	GL15753	-	exonic
GaLu96scf_17_50569_50828	1	0	3	0	0	0	0	GL21902	-	exonic
GaLu96scf_17_590558_590885	2	0	1	0	0	2	0	GL15767	-	exonic
GaLu96scf_17_737815_738454	1	0	3	0	0	0	0	GL21959	-	exonic

GaLu96scf_17_740258_740477	1	0	0	3	0	0	0	GL18772	-	intronic
GaLu96scf_17_791933_792475	1	0	3	0	0	0	0	GL21844	+	exonic
GaLu96scf_17_912833_913118	1	0	0	0	3	0	0	GL21849	+	intronic
GaLu96scf_18_150626_150983	1	0	3	0	0	0	0	GL22045	-	intronic
GaLu96scf_18_364708_365062	2	0	3	0	0	0	0	GL22049	+	exonic
GaLu96scf_18_550773_551645	2	0	3	0	0	0	0	GL22111	-	exonic
GaLu96scf_18_621102_621401	2	0	3	0	0	0	0	GL22024	-	exonic
GaLu96scf_19_165936_166161	1	0	3	0	0	0	0	GL27448	+	intronic
GaLu96scf_19_165936_166254	1	0	0	3	0	0	0	GL27448	+	intronic
GaLu96scf_19_405425_405616	2	0	0	0	0	3	0	GL22200	-	exonic
GaLu96scf_19_494852_495167	2	0	0	0	0	0	3	GL22279	-	exonic
GaLu96scf_19_649165_649603	2	0	0	0	2	0	1	GL22217	-	exonic
GaLu96scf_19_666353_666776	3	0	3	0	0	0	0	GL22162	+	exonic
GaLu96scf_19_696641_697138	2	0	0	2	1	0	0	GL18879	-	exonic
GaLu96scf_19_700802_701028	1	0	3	0	0	0	0	GL22295	+	exonic
GaLu96scf_2_1035829_1036005	1	0	0	0	0	3	0	GL22327	-	exonic
GaLu96scf_2_1096807_1097193	2	0	0	0	3	0	0	GL27511	-	exonic
GaLu96scf_2_1252394_1252875	1	0	0	0	3	0	0	GL22555	+	intronic
GaLu96scf_2_1541899_1544313	3	0	1	0	0	2	0	GL22569	+	exonic
GaLu96scf_2_1990385_1990615	1	0	2	0	0	0	1	GL22599	-	exonic
GaLu96scf_2_2230730_2231131	1	0	3	0	0	0	0	GL27593	+	exonic
GaLu96scf_2_2254411_2254711	3	0	3	0	0	0	0	GL16117	-	exonic
GaLu96scf_2_2292444_2292702	1	0	3	0	0	0	0	GL22508	+	exonic
GaLu96scf_2_663405_663567	1	0	3	0	0	0	0	GL22656	-	intronic
GaLu96scf_2_687184_687623	3	3	0	0	0	0	0	GL22657	-	exonic

GaLu96scf_2_763740_764305	4	3	0	0	0	0	0	GL16163	-	exonic
GaLu96scf_2_778673_780464	3	0	0	0	0	0	3	GL22662	+	exonic
GaLu96scf_2_951265_952060	4	0	3	0	0	0	0	GL27634	-	exonic
GaLu96scf_21_12421_12838	1	3	0	0	0	0	0	GL22824	+	exonic
GaLu96scf_21_139739_139895	1	0	0	2	1	0	0	GL22868	-	intronic
GaLu96scf_21_411133_411598	1	0	2	0	0	0	1	GL27743	+	exonic
GaLu96scf_21_536672_537018	2	0	0	2	1	0	0	GL22843	+	exonic
GaLu96scf_21_641342_642294	4	0	1	0	2	0	0	GL22854	-	exonic
GaLu96scf_21_641871_642294	2	0	0	0	0	0	3	GL22854	-	exonic
GaLu96scf_21_7286_7501	1	0	0	0	0	3	0	GL27718	-	exonic
GaLu96scf_22_118127_119121	6	0	0	0	0	0	3	GL22968	+	exonic
GaLu96scf_22_119250_120316	5	0	3	0	0	0	0	GL22968	+	exonic
GaLu96scf_22_374699_374956	1	0	0	3	0	0	0	GL23000	-	intronic
GaLu96scf_22_780137_780538	3	0	1	1	0	1	0	GL16427	+	exonic
GaLu96scf_22_793393_793771	1	0	0	3	0	0	0	GL23028	-	intronic
GaLu96scf_23_485364_486548	1	0	2	0	1	0	0	GL16469	+	intronic
GaLu96scf_23_531474_532340	3	0	0	0	0	3	0	GL27852	-	exonic
GaLu96scf_23_576088_576351	1	0	0	0	1	1	1	GL27844	-	intronic
GaLu96scf_24_373047_373409	2	3	0	0	0	0	0	GL23146	+	exonic
GaLu96scf_24_432217_432708	3	3	0	0	0	0	0	GL27899	-	exonic
GaLu96scf_24_89145_89484	1	0	2	0	0	0	1	GL23174	-	exonic
GaLu96scf_25_188834_189102	1	0	3	0	0	0	0	GL23270	-	exonic
GaLu96scf_25_427065_427245	1	3	0	0	0	0	0	GL31764	+	exonic
GaLu96scf_25_479612_480088	3	0	0	0	0	3	0	GL23247	-	exonic
GaLu96scf_25_552395_553019	3	0	3	0	0	0	0	GL23228	+	exonic

GaLu96scf_25_72246_72482	1	0	0	0	0	0	3	GL23234	-	intronic
GaLu96scf_26_148280_148789	3	2	0	0	1	0	0	GL23300	+	exonic
GaLu96scf_26_154573_154797	1	0	3	0	0	0	0	GL23350	+	exonic
GaLu96scf_26_157091_157524	2	0	0	0	0	0	3	GL23356	-	exonic
GaLu96scf_26_190303_190716	1	0	0	0	3	0	0	GL16638	-	exonic
GaLu96scf_27_264824_265316	3	0	0	0	3	0	0	GL23438	-	exonic
GaLu96scf_27_504642_504969	2	3	0	0	0	0	0	GL16712	-	exonic
GaLu96scf_28_246565_247057	3	0	3	0	0	0	0	GL23501	-	exonic
GaLu96scf_28_501725_502010	1	3	0	0	0	0	0	GL23507	+	exonic
GaLu96scf_29_265965_266259	1	0	3	0	0	0	0	GL23561	+	intronic
GaLu96scf_29_50588_51371	3	3	0	0	0	0	0	GL23547	-	exonic
GaLu96scf_3_1014919_1015212	1	0	0	0	2	1	0	GL23791	-	exonic
GaLu96scf_3_1237267_1237545	1	3	0	0	0	0	0	GL16835	-	exonic
GaLu96scf_3_1293952_1294103	1	0	3	0	0	0	0	GL23885	-	intronic
GaLu96scf_3_1296420_1296820	1	0	3	0	0	0	0	GL23665	-	exonic
GaLu96scf_3_169589_170273	1	0	3	0	0	0	0	GL16822	+	exonic
GaLu96scf_3_1708280_1708723	3	0	3	0	0	0	0	GL16870	+	exonic
GaLu96scf_3_1868925_1869368	1	0	0	0	0	3	0	GL23915	+	exonic
GaLu96scf_3_330893_331222	1	0	0	3	0	0	0	GL23827	+	exonic
GaLu96scf_3_487592_488357	3	0	0	0	0	0	3	GL23730	-	exonic
GaLu96scf_3_681294_681737	2	0	1	0	0	2	0	GL23954	-	exonic
GaLu96scf_3_702395_703282	1	0	3	0	0	0	0	GL23958	+	exonic
GaLu96scf_31_182848_183132	2	3	0	0	0	0	0	GL24078	+	exonic
GaLu96scf_31_201279_201444	1	3	0	0	0	0	0	GL24088	-	intronic
GaLu96scf_31_259187_259382	1	0	0	0	0	3	0	GL28295	-	exonic

GaLu96scf_31_259442_259684	1	1	0	0	2	0	0	GL28295	-	exonic
GaLu96scf_31_438410_439709	2	0	0	0	0	0	3	GL24130	+	exonic
GaLu96scf_32_167460_167705	1	0	3	0	0	0	0	GL24162	+	exonic
GaLu96scf_32_169832_170260	3	0	3	0	0	0	0	GL24144	-	exonic
GaLu96scf_32_199917_200290	1	3	0	0	0	0	0	GL24168	+	exonic
GaLu96scf_34_36492_36748	1	0	2	0	0	1	0	GL28358	-	exonic
GaLu96scf_34_81486_82461	2	0	3	0	0	0	0	GL24264	+	exonic
GaLu96scf_35_54893_55439	4	0	3	0	0	0	0	GL17140	+	exonic
GaLu96scf_36_11891_12276	3	0	3	0	0	0	0	GL29265	-	exonic
GaLu96scf_37_118613_119521	5	0	0	0	3	0	0	GL24359	-	exonic
GaLu96scf_37_57598_57784	1	0	0	1	2	0	0	GL24357	+	intronic
GaLu96scf_37_79294_79630	1	1	2	0	0	0	0	GL17170	+	intronic
GaLu96scf_38_28561_28821	1	0	3	0	0	0	0	GL28437	+	exonic
GaLu96scf_39_138773_139007	2	0	0	0	0	3	0	GL28461	+	exonic
GaLu96scf_4_1159977_1160256	1	0	0	3	0	0	0	GL24490	+	exonic
GaLu96scf_4_1332782_1333094	1	0	3	0	0	0	0	GL24774	-	exonic
GaLu96scf_4_1587419_1588086	2	0	0	0	0	3	0	GL17283	-	exonic
GaLu96scf_4_168137_168468	1	0	0	0	0	3	0	GL28477	+	intronic
GaLu96scf_4_169285_170276	3	3	0	0	0	0	0	GL28477	+	exonic
GaLu96scf_4_1883505_1885135	10	0	0	0	0	3	0	GL24531	+	exonic
GaLu96scf_4_210025_210368	2	0	3	0	0	0	0	GL24544	-	exonic
GaLu96scf_4_283196_283529	3	0	0	3	0	0	0	GL24548	-	exonic
GaLu96scf_4_387739_388725	5	0	0	0	0	0	3	GL17336	+	exonic
GaLu96scf_4_388390_388725	1	0	3	0	0	0	0	GL17336	+	exonic
GaLu96scf_4_672212_672533	2	0	0	0	0	3	0	GL24581	-	exonic

GaLu96scf_4_706195_706457	2	0	0	0	0	3	0	GL17355	-	exonic
GaLu96scf_4_947139_948009	3	0	3	0	0	0	0	GL24707	+	exonic
GaLu96scf_40_129008_129524	3	0	2	0	0	0	1	GL24889	+	exonic
GaLu96scf_40_3122_3636	3	0	0	3	0	0	0	GL24883	+	exonic
GaLu96scf_41_124935_125140	1	0	2	0	1	0	0	GL24934	-	exonic
GaLu96scf_44_96119_96919	2	0	3	0	0	0	0	GL25033	-	exonic
GaLu96scf_46_57339_57765	2	0	1	0	1	0	1	GL25090	+	exonic
GaLu96scf_47_64056_64701	2	0	0	0	1	2	0	GL25120	-	exonic
GaLu96scf_47_71790_72319	2	0	3	0	0	0	0	GL25110	-	exonic
GaLu96scf_47_89640_89895	2	0	1	0	2	0	0	GL25106	+	exonic
GaLu96scf_48_75884_76579	2	0	0	1	0	0	2	GL19912	-	exonic
GaLu96scf_49_51185_51575	3	3	0	0	0	0	0	GL25138	-	exonic
GaLu96scf_5_1254833_1255305	1	0	0	3	0	0	0	GL25376	-	exonic
GaLu96scf_5_1257569_1258000	1	0	0	0	0	0	3	GL28715	-	exonic
GaLu96scf_5_174453_175007	2	0	0	0	3	0	0	GL25348	+	exonic
GaLu96scf_5_275854_276060	1	0	0	0	0	3	0	GL25408	+	exonic
GaLu96scf_5_417846_418112	2	0	3	0	0	0	0	GL25223	+	exonic
GaLu96scf_5_683753_684442	4	0	3	0	0	0	0	GL17625	-	exonic
GaLu96scf_6_1003430_1004109	2	0	3	0	0	0	0	GL31373	+	exonic
GaLu96scf_6_129518_129950	2	3	0	0	0	0	0	GL31480	-	exonic
GaLu96scf_6_1366658_1366983	1	2	0	1	0	0	0	GL31646	+	exonic
GaLu96scf_6_267038_267281	1	0	0	0	3	0	0	GL31561	-	exonic
GaLu96scf_6_597929_599233	2	0	3	0	0	0	0	GL31599	-	exonic
GaLu96scf_6_864126_864880	4	0	0	3	0	0	0	GL31453	-	exonic
GaLu96scf_6_940758_941146	2	0	3	0	0	0	0	GL31455	+	exonic

GaLu96scf_7_1167567_1168011	2	0	0	3	0	0	0	GL25544	+	exonic
GaLu96scf_7_1311167_1311481	1	0	0	1	0	1	1	GL17766	+	exonic
GaLu96scf_7_1367812_1368966	3	0	0	2	0	0	1	GL25639	-	exonic
GaLu96scf_7_238736_240226	1	0	0	0	2	0	1	GL20165	+	exonic
GaLu96scf_7_395510_396253	4	0	3	0	0	0	0	GL25583	-	exonic
GaLu96scf_7_641555_641838	3	0	3	0	0	0	0	GL25603	+	exonic
GaLu96scf_7_763431_763969	4	0	0	0	0	1	2	GL25764	+	exonic
GaLu96scf_7_926139_927101	6	0	3	0	0	0	0	GL25783	-	exonic
GaLu96scf_8_1095637_1096306	3	0	3	0	0	0	0	GL25821	+	exonic
GaLu96scf_8_1226682_1228494	8	0	0	0	1	1	1	GL25958	-	exonic
GaLu96scf_8_1294273_1294858	1	0	0	0	3	0	0	GL25845	+	exonic
GaLu96scf_8_131690_132152	4	0	0	3	0	0	0	GL25810	+	exonic
GaLu96scf_8_1530460_1530686	1	0	0	3	0	0	0	GL26080	+	intronic
GaLu96scf_8_179541_179841	1	0	0	0	0	3	0	GL20230	+	exonic
GaLu96scf_8_369875_370161	2	0	0	0	0	0	3	GL25881	+	exonic
GaLu96scf_8_460253_460584	2	0	3	0	0	0	0	GL26103	+	exonic
GaLu96scf_8_460763_461010	1	0	3	0	0	0	0	GL26103	+	intronic
GaLu96scf_8_800829_801235	2	0	3	0	0	0	0	GL26014	+	exonic
GaLu96scf_8_885685_886027	2	3	0	0	0	0	0	GL26140	-	exonic
GaLu96scf_9_1336812_1337034	1	0	3	0	0	0	0	GL26723	+	exonic
GaLu96scf_9_1384751_1385131	3	0	3	0	0	0	0	GL26730	-	exonic
GaLu96scf_9_1491693_1492185	3	0	3	0	0	0	0	GL18134	+	exonic
GaLu96scf_9_1516501_1517364	2	0	0	0	0	0	3	GL26737	+	exonic
GaLu96scf_9_220011_220903	3	0	0	3	0	0	0	GL18145	-	exonic
GaLu96scf_9_672509_673606	5	0	1	2	0	0	0	GL20443	-	exonic

GaLu96scf_9_801409_801927	2	0	3	0	0	0	0	GL26561	+	exonic
GaLu96scf_9_819996_820877	4	0	3	0	0	0	0	GL26562	+	exonic
GaLu96scf_9_865492_865878	2	0	0	0	0	3	0	GL26572	-	exonic
GaLu96scf_1_1097860_1098442	4	0	0	2	0	0	0	GL29849	+	exonic
GaLu96scf_1_111570_111746	1	2	0	0	0	0	0	GL30176	-	exonic
GaLu96scf_1_1136981_1137856	5	0	2	0	0	0	0	GL30182	-	exonic
GaLu96scf_1_1137924_1138269	1	0	0	2	0	0	0	GL30182	-	intronic
GaLu96scf_1_1148535_1148700	1	0	0	0	0	0	2	GL30442	-	exonic
GaLu96scf_1_1349897_1350878	5	0	0	2	0	0	0	GL29865	+	exonic
GaLu96scf_1_1468308_1468583	1	0	0	0	0	0	2	GL29882	-	exonic
GaLu96scf_1_1608824_1609226	2	0	2	0	0	0	0	GL30910	-	exonic
GaLu96scf_1_2046413_2047207	3	0	2	0	0	0	0	GL30934	-	exonic
GaLu96scf_1_2061880_2062166	2	0	0	0	0	1	1	GL29943	-	exonic
GaLu96scf_1_2168770_2168960	1	0	0	0	0	2	0	GL30250	-	intronic
GaLu96scf_1_2188296_2188591	2	0	0	0	0	2	0	GL30947	+	exonic
GaLu96scf_1_2214466_2214970	4	0	0	1	1	0	0	GL29400	-	exonic
GaLu96scf_1_2347108_2347579	2	0	0	0	0	0	2	GL30566	+	exonic
GaLu96scf_1_2395173_2395579	2	0	0	2	0	0	0	GL30574	-	exonic
GaLu96scf_1_2607455_2607804	1	0	2	0	0	0	0	GL30965	+	exonic
GaLu96scf_1_2716972_2717313	1	0	2	0	0	0	0	GL29424	-	exonic
GaLu96scf_1_2837166_2837700	2	0	2	0	0	0	0	GL29731	+	exonic
GaLu96scf_1_2937155_2938045	2	0	0	2	0	0	0	GL30292	+	exonic
GaLu96scf_1_2942213_2942637	2	0	0	0	2	0	0	GL30630	+	exonic
GaLu96scf_1_3003256_3003452	1	0	2	0	0	0	0	GL30023	+	intronic
GaLu96scf_1_3332229_3333104	3	0	0	0	0	2	0	GL30048	+	exonic

GaLu96scf_1_343818_344528	4	0	0	0	0	1	1	GL30986	-	exonic
GaLu96scf_1_3477024_3477420	1	0	2	0	0	0	0	GL30340	+	exonic
GaLu96scf_1_3587554_3587760	1	0	0	0	2	0	0	GL30713	+	intronic
GaLu96scf_1_3736883_3737106	1	0	2	0	0	0	0	GL30357	-	exonic
GaLu96scf_1_3766876_3767525	4	0	0	2	0	0	0	GL30084	-	exonic
GaLu96scf_1_3906930_3907600	4	0	2	0	0	0	0	GL31772	+	exonic
GaLu96scf_1_3922878_3923420	3	0	0	0	2	0	0	GL30361	+	exonic
GaLu96scf_1_3929297_3929778	2	0	2	0	0	0	0	GL29505	-	exonic
GaLu96scf_1_4116640_4117290	1	2	0	0	0	0	0	GL29535	+	exonic
GaLu96scf_1_4154920_4155135	1	0	0	2	0	0	0	GL30108	+	intronic
GaLu96scf_1_4154920_4155149	1	0	0	2	0	0	0	GL30108	+	intronic
GaLu96scf_1_4225035_4225218	1	0	0	2	0	0	0	GL30790	+	intronic
GaLu96scf_1_4342184_4342887	2	0	0	0	2	0	0	GL30122	-	exonic
GaLu96scf_1_441144_442809	7	0	0	1	1	0	0	GL30097	-	exonic
GaLu96scf_1_441534_442359	3	0	0	0	0	2	0	GL30097	-	exonic
GaLu96scf_1_441534_442809	5	0	0	0	1	0	1	GL30097	-	exonic
GaLu96scf_1_447870_448211	1	0	2	0	0	0	0	GL30761	+	exonic
GaLu96scf_1_4712603_4712936	1	0	0	0	0	2	0	GL29595	-	exonic
GaLu96scf_1_4765780_4766160	2	0	2	0	0	0	0	GL31084	+	exonic
GaLu96scf_1_481248_481622	1	2	0	0	0	0	0	GL30766	+	exonic
GaLu96scf_1_4812876_4813249	1	0	2	0	0	0	0	GL30826	-	exonic
GaLu96scf_1_749484_749685	1	2	0	0	0	0	0	GL30159	-	exonic
GaLu96scf_1_815100_815299	1	0	0	0	0	0	2	GL30164	+	exonic
GaLu96scf_1_823157_823309	1	0	0	0	2	0	0	GL29633	-	exonic
GaLu96scf_10_1240737_1241190	1	0	0	0	0	2	0	GL20473	-	intronic

GaLu96scf_10_1301597_1301774	1	0	2	0	0	0	0	GL15046	-	exonic
GaLu96scf_10_231140_231396	1	0	2	0	0	0	0	GL20598	-	exonic
GaLu96scf_10_325914_326491	1	0	0	1	1	0	0	GL15079	+	exonic
GaLu96scf_10_339814_340017	1	0	0	2	0	0	0	GL20542	+	exonic
GaLu96scf_10_57748_58573	5	0	0	0	0	2	0	GL15004	-	exonic
GaLu96scf_10_632631_633434	3	2	0	0	0	0	0	GL20627	+	exonic
GaLu96scf_10_633434_633755	1	0	2	0	0	0	0	GL20627	+	intronic
GaLu96scf_10_64515_64701	1	0	0	1	0	0	1	GL20529	+	intronic
GaLu96scf_10_743406_744527	2	0	0	0	0	2	0	GL20510	+	exonic
GaLu96scf_10_743556_744003	1	2	0	0	0	0	0	GL20510	+	intronic
GaLu96scf_10_946341_947176	3	0	2	0	0	0	0	GL20523	+	exonic
GaLu96scf_11_1246408_1246608	1	0	0	0	0	2	0	GL20812	+	intronic
GaLu96scf_11_1356921_1357132	1	0	2	0	0	0	0	GL15172	-	intronic
GaLu96scf_11_289556_290566	7	0	0	2	0	0	0	GL20687	-	exonic
GaLu96scf_11_612117_612944	4	0	2	0	0	0	0	GL20868	-	exonic
GaLu96scf_11_714241_714664	1	0	0	0	2	0	0	GL20771	-	intronic
GaLu96scf_11_714341_714664	1	0	0	0	0	2	0	GL20771	-	intronic
GaLu96scf_11_748237_748931	3	0	0	2	0	0	0	GL20712	+	exonic
GaLu96scf_11_836856_837292	2	0	2	0	0	0	0	GL20877	+	exonic
GaLu96scf_12_387158_387622	2	0	2	0	0	0	0	GL21056	-	exonic
GaLu96scf_12_450878_452244	3	0	0	1	0	1	0	GL18437	-	exonic
GaLu96scf_12_711922_712334	3	0	0	0	1	0	1	GL15330	-	exonic
GaLu96scf_12_717872_718096	1	0	2	0	0	0	0	GL21074	-	intronic
GaLu96scf_12_722155_722381	1	0	0	2	0	0	0	GL20939	+	exonic
GaLu96scf_12_842952_843260	1	0	0	0	0	1	1	GL20952	+	exonic

GaLu96scf_12_883406_884593	2	0	0	2	0	0	0	GL15334	-	exonic
GaLu96scf_12_925185_926090	4	0	0	2	0	0	0	GL21085	+	exonic
GaLu96scf_12_934832_935015	1	0	2	0	0	0	0	GL20966	-	intronic
GaLu96scf_13_1191551_1191848	1	0	0	0	2	0	0	GL21218	-	intronic
GaLu96scf_13_299485_300034	3	0	0	1	0	0	1	GL21224	-	exonic
GaLu96scf_13_301980_302188	1	0	0	0	0	2	0	GL21175	+	exonic
GaLu96scf_13_548130_548451	1	0	2	0	0	0	0	GL27133	-	exonic
GaLu96scf_13_657956_658414	3	0	2	0	0	0	0	GL18503	+	exonic
GaLu96scf_13_742845_743112	2	0	2	0	0	0	0	GL21139	+	exonic
GaLu96scf_13_760855_761049	1	0	0	0	2	0	0	GL21147	-	exonic
GaLu96scf_13_965523_965806	2	0	2	0	0	0	0	GL21264	+	exonic
GaLu96scf_14_268918_269255	2	0	0	0	0	0	2	GL27169	+	exonic
GaLu96scf_14_341177_341518	1	0	0	0	0	0	2	GL15489	+	exonic
GaLu96scf_14_521364_521765	2	0	0	2	0	0	0	GL21420	-	exonic
GaLu96scf_14_579214_579573	1	0	0	0	2	0	0	GL21307	+	exonic
GaLu96scf_14_722703_722905	1	0	0	0	0	2	0	GL21438	-	exonic
GaLu96scf_14_736748_736996	1	0	0	1	0	0	1	GL21361	+	exonic
GaLu96scf_14_8634_9042	2	0	0	2	0	0	0	GL21375	+	exonic
GaLu96scf_14_881012_881245	1	0	0	0	1	1	0	GL21446	+	intronic
GaLu96scf_15_511452_512189	1	0	2	0	0	0	0	GL21487	+	exonic
GaLu96scf_15_512189_512496	1	0	2	0	0	0	0	GL21487	+	intronic
GaLu96scf_15_512512_513690	1	0	0	0	2	0	0	GL21487	+	exonic
GaLu96scf_15_528366_528675	1	0	2	0	0	0	0	GL21580	+	intronic
GaLu96scf_15_578462_578927	2	2	0	0	0	0	0	GL21539	+	exonic
GaLu96scf_15_610777_611483	2	0	0	0	0	0	2	GL15617	+	exonic

GaLu96scf_15_763952_764785	5	0	0	0	2	0	0	GL21597	+	exonic
GaLu96scf_15_805013_805191	1	0	0	1	1	0	0	GL21513	-	intronic
GaLu96scf_15_888746_889844	3	0	0	2	0	0	0	GL21511	+	exonic
GaLu96scf_16_622307_623891	6	0	0	0	1	1	0	GL21694	-	exonic
GaLu96scf_16_744184_744677	3	2	0	0	0	0	0	GL21660	-	exonic
GaLu96scf_16_803908_804240	2	0	0	0	0	0	2	GL15698	+	exonic
GaLu96scf_16_851595_852384	4	0	0	0	2	0	0	GL21712	-	exonic
GaLu96scf_16_855760_856976	5	0	0	0	2	0	0	GL15699	-	exonic
GaLu96scf_17_427671_428169	1	0	0	2	0	0	0	GL21870	+	exonic
GaLu96scf_17_538958_539229	2	0	0	0	0	2	0	GL15763	-	exonic
GaLu96scf_17_565232_566488	8	0	2	0	0	0	0	GL21832	+	exonic
GaLu96scf_17_790693_791200	1	0	0	0	2	0	0	GL21844	+	intronic
GaLu96scf_17_917642_917802	1	0	0	2	0	0	0	GL21970	+	intronic
GaLu96scf_18_167689_168072	1	0	0	0	0	0	2	GL18809	+	exonic
GaLu96scf_18_250631_251183	2	0	0	0	2	0	0	GL21992	-	exonic
GaLu96scf_18_295732_296259	2	2	0	0	0	0	0	GL22088	+	exonic
GaLu96scf_18_447013_447709	2	0	0	2	0	0	0	GL22059	-	exonic
GaLu96scf_18_567176_567953	3	0	0	0	0	0	2	GL27401	-	exonic
GaLu96scf_18_57827_58034	1	0	0	0	2	0	0	GL18793	+	intronic
GaLu96scf_18_710668_711112	2	0	2	0	0	0	0	GL22029	-	exonic
GaLu96scf_19_130489_130885	1	0	0	0	0	0	2	GL22138	-	exonic
GaLu96scf_19_140306_140674	1	0	0	0	0	2	0	GL22248	-	exonic
GaLu96scf_19_564649_565108	2	0	0	0	0	0	2	GL22285	-	exonic
GaLu96scf_19_737976_738397	2	0	0	2	0	0	0	GL22301	-	exonic
GaLu96scf_19_798779_799537	2	0	0	0	0	0	2	GL22226	-	exonic

GaLu96scf_19_800399_801639	4	0	0	0	2	0	0	GL22226	-	exonic
GaLu96scf_19_819732_819912	1	0	0	1	0	1	0	GL22176	+	exonic
GaLu96scf_2_1017108_1017376	1	0	2	0	0	0	0	GL22326	-	exonic
GaLu96scf_2_1155577_1155908	2	0	0	0	0	2	0	GL22335	+	exonic
GaLu96scf_2_1251831_1252254	1	0	0	0	2	0	0	GL22555	+	intronic
GaLu96scf_2_1262267_1262457	2	0	0	0	0	0	2	GL22343	+	exonic
GaLu96scf_2_1315017_1315436	2	0	0	0	0	0	2	GL22351	+	exonic
GaLu96scf_2_145953_146524	1	0	2	0	0	0	0	GL22317	+	exonic
GaLu96scf_2_1547585_1547797	1	0	0	0	0	2	0	GL22570	+	exonic
GaLu96scf_2_1664767_1665032	1	0	0	2	0	0	0	GL22585	-	exonic
GaLu96scf_2_1724880_1725264	1	0	2	0	0	0	0	GL22587	+	exonic
GaLu96scf_2_1792380_1792908	3	0	0	0	2	0	0	GL16056	+	exonic
GaLu96scf_2_2071323_2071648	2	0	2	0	0	0	0	GL27583	-	exonic
GaLu96scf_2_2244381_2245316	6	0	0	2	0	0	0	GL22624	+	exonic
GaLu96scf_2_235229_235496	2	0	2	0	0	0	0	GL22491	+	exonic
GaLu96scf_2_575900_576671	1	0	0	0	2	0	0	GL22647	+	intronic
GaLu96scf_2_663567_663734	1	0	2	0	0	0	0	GL22656	-	exonic
GaLu96scf_2_733930_734257	2	0	0	0	1	1	0	GL18990	+	exonic
GaLu96scf_2_787531_787884	2	2	0	0	0	0	0	GL22443	-	exonic
GaLu96scf_2_813532_814126	1	0	0	0	2	0	0	GL22665	+	exonic
GaLu96scf_2_873081_873396	1	2	0	0	0	0	0	GL22673	-	exonic
GaLu96scf_2_888992_889828	6	0	2	0	0	0	0	GL16169	-	exonic
GaLu96scf_2_954528_954932	2	0	0	0	0	2	0	GL22445	+	exonic
GaLu96scf_2_980219_980498	1	0	0	1	1	0	0	GL27636	-	intronic
GaLu96scf_20_277215_277725	1	0	0	0	0	0	2	GL22709	+	exonic

GaLu96scf_20_567409_567694	2	0	0	2	0	0	0	GL22720	-	exonic
GaLu96scf_20_71483_71788	1	2	0	0	0	0	0	GL22679	+	exonic
GaLu96scf_20_824149_825288	5	0	0	0	2	0	0	GL22699	+	exonic
GaLu96scf_20_846169_846664	3	0	0	0	2	0	0	GL22726	-	exonic
GaLu96scf_20_865069_865450	1	0	0	0	2	0	0	GL16274	+	exonic
GaLu96scf_20_869159_869574	2	0	2	0	0	0	0	GL27716	-	exonic
GaLu96scf_21_135392_135697	1	0	0	0	0	0	2	GL22863	+	exonic
GaLu96scf_21_181854_182269	2	0	2	0	0	0	0	GL22829	+	exonic
GaLu96scf_21_370616_370925	1	0	0	0	0	2	0	GL22878	+	intronic
GaLu96scf_21_370616_370933	1	0	0	0	2	0	0	GL22878	+	intronic
GaLu96scf_21_759169_759412	1	0	0	0	0	0	2	GL22902	+	intronic
GaLu96scf_22_100403_100647	1	0	0	0	2	0	0	GL31760	-	exonic
GaLu96scf_22_184039_184954	2	0	0	0	2	0	0	GL22918	-	exonic
GaLu96scf_22_598214_598958	3	0	0	0	0	2	0	GL27809	+	exonic
GaLu96scf_22_715149_715511	1	0	0	2	0	0	0	GL27813	-	intronic
GaLu96scf_22_718382_719099	3	0	2	0	0	0	0	GL22946	+	exonic
GaLu96scf_22_757720_757914	1	0	0	2	0	0	0	GL22951	+	intronic
GaLu96scf_22_779372_780064	4	0	0	2	0	0	0	GL16427	+	exonic
GaLu96scf_23_264842_265195	1	0	0	2	0	0	0	GL23102	+	intronic
GaLu96scf_23_286187_286610	1	0	2	0	0	0	0	GL23040	+	intronic
GaLu96scf_23_531474_532829	4	0	0	0	2	0	0	GL27852	-	exonic
GaLu96scf_23_615904_616496	3	0	0	0	2	0	0	GL16483	+	exonic
GaLu96scf_23_84103_84530	1	0	0	2	0	0	0	GL23073	+	intronic
GaLu96scf_23_85116_85717	2	0	2	0	0	0	0	GL23073	+	exonic
GaLu96scf_24_113250_113461	1	0	0	0	2	0	0	GL23185	-	exonic

GaLu96scf_24_187624_188064	3	0	2	0	0	0	0	GL23184	+	exonic
GaLu96scf_24_19069_19388	1	0	2	0	0	0	0	GL23161	-	exonic
GaLu96scf_24_191115_191629	1	0	0	1	0	0	1	GL19230	+	intronic
GaLu96scf_24_224193_224464	1	0	2	0	0	0	0	GL27891	-	exonic
GaLu96scf_24_237549_237961	2	0	2	0	0	0	0	GL16506	-	exonic
GaLu96scf_24_246613_246869	1	0	0	0	0	0	2	GL23189	-	exonic
GaLu96scf_24_274858_275334	2	0	0	1	0	0	1	GL23141	+	exonic
GaLu96scf_24_410157_410485	1	0	2	0	0	0	0	GL23153	-	exonic
GaLu96scf_24_45061_45349	1	0	0	1	0	0	1	GL23173	-	intronic
GaLu96scf_24_642832_643032	1	0	2	0	0	0	0	GL23172	-	exonic
GaLu96scf_25_109707_109981	2	0	0	0	0	2	0	GL19267	-	exonic
GaLu96scf_25_479837_480088	2	0	0	1	1	0	0	GL23247	-	exonic
GaLu96scf_26_125622_126013	2	0	2	0	0	0	0	GL23301	-	exonic
GaLu96scf_26_147365_148201	4	0	2	0	0	0	0	GL23300	+	exonic
GaLu96scf_26_278041_278380	1	2	0	0	0	0	0	GL16646	+	exonic
GaLu96scf_26_548301_548661	1	0	0	0	0	2	0	GL23326	-	exonic
GaLu96scf_27_502381_502971	2	0	2	0	0	0	0	GL23466	-	exonic
GaLu96scf_27_502790_502971	1	0	2	0	0	0	0	GL23466	-	exonic
GaLu96scf_27_564612_564797	2	0	0	0	2	0	0	GL23463	+	exonic
GaLu96scf_28_363019_363817	3	0	0	0	2	0	0	GL23482	+	exonic
GaLu96scf_28_39753_39969	1	0	2	0	0	0	0	GL19382	-	intronic
GaLu96scf_28_39969_40185	2	0	2	0	0	0	0	GL19382	-	exonic
GaLu96scf_29_307235_307405	1	2	0	0	0	0	0	GL19429	-	intronic
GaLu96scf_29_326624_326916	1	0	1	0	0	1	0	GL23585	+	intronic
GaLu96scf_29_36074_36450	2	0	0	0	2	0	0	GL23546	-	exonic

GaLu96scf_29_434371_435062	2	0	0	0	0	0	2	GL23575	+	exonic
GaLu96scf_3_1113081_1113834	3	2	0	0	0	0	0	GL23795	-	exonic
GaLu96scf_3_1117772_1118098	1	0	0	1	0	1	0	GL23645	+	exonic
GaLu96scf_3_1134626_1134932	1	0	0	2	0	0	0	GL23647	+	exonic
GaLu96scf_3_1176468_1176724	1	2	0	0	0	0	0	GL23799	+	exonic
GaLu96scf_3_1247057_1247583	2	0	2	0	0	0	0	GL16837	-	exonic
GaLu96scf_3_1279545_1279732	1	0	0	0	0	2	0	GL28147	-	exonic
GaLu96scf_3_1340129_1340492	1	0	0	2	0	0	0	GL23808	-	exonic
GaLu96scf_3_1341812_1342237	2	0	0	0	2	0	0	GL23669	+	exonic
GaLu96scf_3_1674340_1676107	7	0	2	0	0	0	0	GL23899	+	exonic
GaLu96scf_3_179057_179236	1	0	0	0	0	1	1	GL23630	+	exonic
GaLu96scf_3_1816326_1816794	2	0	2	0	0	0	0	GL23918	-	exonic
GaLu96scf_3_1899568_1899774	1	0	0	0	0	0	2	GL23921	+	intronic
GaLu96scf_3_1912474_1913997	6	0	2	0	0	0	0	GL23706	-	exonic
GaLu96scf_3_217542_217997	2	0	0	2	0	0	0	GL31719	+	exonic
GaLu96scf_3_261404_261712	2	0	0	0	0	0	2	GL16890	-	exonic
GaLu96scf_3_539732_539962	2	0	0	0	0	2	0	GL19525	-	exonic
GaLu96scf_3_628032_628395	2	0	1	0	0	1	0	GL23752	-	exonic
GaLu96scf_3_711836_712356	3	0	2	0	0	0	0	GL23962	-	exonic
GaLu96scf_3_952022_952910	2	0	2	0	0	0	0	GL23774	-	exonic
GaLu96scf_3_997583_997926	2	0	1	0	0	1	0	GL16827	+	exonic
GaLu96scf_30_123774_124029	1	0	0	2	0	0	0	GL19568	+	intronic
GaLu96scf_30_184163_184358	1	0	2	0	0	0	0	GL28255	-	intronic
GaLu96scf_30_188966_189297	1	0	2	0	0	0	0	GL24014	-	exonic
GaLu96scf_30_367670_368401	2	0	2	0	0	0	0	GL19589	-	exonic

GaLu96scf_30_58013_58623	4	2	0	0	0	0	0	GL24022	-	exonic
GaLu96scf_31_21062_21764	4	0	2	0	0	0	0	GL24095	+	exonic
GaLu96scf_31_287068_287601	3	0	2	0	0	0	0	GL17002	-	exonic
GaLu96scf_31_361450_361696	1	0	0	0	2	0	0	GL17007	-	intronic
GaLu96scf_31_361451_361696	1	0	0	0	0	2	0	GL17007	-	intronic
GaLu96scf_31_375583_375831	1	0	0	0	0	2	0	GL19615	-	exonic
GaLu96scf_31_435294_435723	1	0	2	0	0	0	0	GL24130	+	intronic
GaLu96scf_31_47445_47654	1	0	2	0	0	0	0	GL24075	-	intronic
GaLu96scf_32_169978_170260	2	2	0	0	0	0	0	GL24144	-	exonic
GaLu96scf_33_192894_193677	3	0	0	2	0	0	0	GL24233	-	exonic
GaLu96scf_34_340054_340405	2	0	0	0	1	1	0	GL24260	+	exonic
GaLu96scf_34_36769_37109	1	2	0	0	0	0	0	GL28358	-	intronic
GaLu96scf_35_180898_181075	1	0	0	0	0	2	0	GL24307	-	exonic
GaLu96scf_35_279057_279437	2	0	0	0	2	0	0	GL28408	-	exonic
GaLu96scf_35_350223_350993	4	0	0	2	0	0	0	GL24312	+	exonic
GaLu96scf_36_11719_12276	4	2	0	0	0	0	0	GL29265	-	exonic
GaLu96scf_36_208549_209343	4	0	0	0	0	2	0	GL29277	-	exonic
GaLu96scf_36_242948_243391	2	0	0	0	0	2	0	GL29262	-	exonic
GaLu96scf_36_243459_243973	4	0	0	0	0	0	2	GL29262	-	exonic
GaLu96scf_37_224737_225370	1	0	2	0	0	0	0	GL24384	-	exonic
GaLu96scf_37_72558_72946	2	0	0	0	0	2	0	GL24371	-	exonic
GaLu96scf_39_134915_135305	2	0	0	0	0	2	0	GL24433	+	exonic
GaLu96scf_39_186149_186703	2	0	2	0	0	0	0	GL24454	-	exonic
GaLu96scf_4_1040193_1040815	4	0	0	0	0	0	2	GL24479	-	exonic
GaLu96scf_4_1120939_1121351	1	0	1	0	1	0	0	GL28483	+	exonic

GaLu96scf_4_1124355_1124856	3	0	0	0	2	0	0	GL17256	-	exonic
GaLu96scf_4_1158491_1158957	3	2	0	0	0	0	0	GL24490	+	exonic
GaLu96scf_4_1218208_1218787	1	0	0	0	0	2	0	GL24496	-	exonic
GaLu96scf_4_1372260_1372863	2	0	2	0	0	0	0	GL19770	-	exonic
GaLu96scf_4_1388291_1388647	2	0	2	0	0	0	0	GL24775	-	exonic
GaLu96scf_4_1397236_1397474	1	0	1	1	0	0	0	GL17274	-	exonic
GaLu96scf_4_1560573_1560988	2	0	0	0	2	0	0	GL24518	-	exonic
GaLu96scf_4_168642_170276	5	0	2	0	0	0	0	GL28477	+	exonic
GaLu96scf_4_175342_176622	7	0	0	0	0	2	0	GL28475	-	exonic
GaLu96scf_4_1902315_1902566	2	0	0	0	0	2	0	GL24533	-	exonic
GaLu96scf_4_381502_382169	3	0	0	2	0	0	0	GL17335	+	exonic
GaLu96scf_4_385639_386010	2	0	0	0	0	0	2	GL28544	-	exonic
GaLu96scf_4_56967_57903	5	0	0	0	2	0	0	GL24457	+	exonic
GaLu96scf_4_619799_620393	2	1	0	0	0	0	1	GL24851	+	exonic
GaLu96scf_4_692211_693127	4	0	0	0	2	0	0	GL24584	-	exonic
GaLu96scf_4_772594_773018	1	0	0	0	0	0	2	GL24689	+	exonic
GaLu96scf_4_942105_942888	3	0	0	0	1	1	0	GL17366	+	exonic
GaLu96scf_4_973260_973767	3	0	2	0	0	0	0	GL24618	-	exonic
GaLu96scf_41_110255_110854	3	0	0	1	0	0	1	GL24929	+	exonic
GaLu96scf_41_110911_111187	1	0	0	0	0	2	0	GL24929	+	exonic
GaLu96scf_42_126736_127063	2	0	0	0	0	0	2	GL24958	+	exonic
GaLu96scf_42_65944_66602	3	0	0	2	0	0	0	GL19863	+	exonic
GaLu96scf_44_32765_33242	1	0	0	0	0	2	0	GL25038	-	exonic
GaLu96scf_46_125126_125501	2	2	0	0	0	0	0	GL17498	-	exonic
GaLu96scf_46_78604_80089	3	0	0	0	0	2	0	GL25091	+	exonic

GaLu96scf_47_127623_127778	1	0	1	1	0	0	0	GL25125	-	exonic
GaLu96scf_5_1002250_1002412	1	0	0	0	1	1	0	GL19945	+	exonic
GaLu96scf_5_1055983_1056246	2	0	0	0	0	2	0	GL25356	-	exonic
GaLu96scf_5_138024_138176	1	0	2	0	0	0	0	GL25276	+	intronic
GaLu96scf_5_1381747_1382048	1	0	2	0	0	0	0	GL25382	-	exonic
GaLu96scf_5_1583766_1584771	5	0	0	0	2	0	0	GL25397	-	exonic
GaLu96scf_5_1645188_1645538	3	0	0	0	0	0	2	GL17581	-	exonic
GaLu96scf_5_288366_288837	2	0	2	0	0	0	0	GL25300	+	exonic
GaLu96scf_5_327008_327330	2	2	0	0	0	0	0	GL25216	+	exonic
GaLu96scf_5_440858_441231	2	0	2	0	0	0	0	GL25312	+	exonic
GaLu96scf_5_626118_626279	1	2	0	0	0	0	0	GL25242	+	exonic
GaLu96scf_5_76225_76661	2	0	0	0	0	0	2	GL17531	-	exonic
GaLu96scf_5_788336_788501	1	0	0	2	0	0	0	GL25251	-	intronic
GaLu96scf_5_789033_789509	2	0	0	0	0	1	1	GL25322	+	exonic
GaLu96scf_5_800729_802271	2	0	0	0	0	2	0	GL25252	+	exonic
GaLu96scf_5_811092_812027	3	0	2	0	0	0	0	GL25253	+	exonic
GaLu96scf_5_881446_881949	3	0	2	0	0	0	0	GL17637	-	exonic
GaLu96scf_52_7460_8266	5	0	0	0	0	2	0	GL25501	-	exonic
GaLu96scf_6_1040523_1040859	2	0	0	0	0	2	0	GL31487	+	exonic
GaLu96scf_6_1059544_1059718	1	0	0	1	1	0	0	GL31380	-	intronic
GaLu96scf_6_1245249_1245602	1	0	0	1	1	0	0	GL31387	+	exonic
GaLu96scf_6_1645833_1646175	1	0	0	0	2	0	0	GL31551	+	intronic
GaLu96scf_6_196215_196660	3	2	0	0	0	0	0	GL31370	+	exonic
GaLu96scf_6_354196_354411	2	0	0	0	0	2	0	GL31414	+	exonic
GaLu96scf_6_354335_354658	2	0	0	0	2	0	0	GL31414	+	exonic

GaLu96scf_6_370594_370941	1	0	0	0	2	0	0	GL31572	-	exonic
GaLu96scf_6_38197_38349	1	0	0	0	0	2	0	GL31134	-	intronic
GaLu96scf_6_490500_491793	6	0	0	0	0	2	0	GL31303	-	exonic
GaLu96scf_6_597741_599233	3	0	0	1	0	1	0	GL31599	-	exonic
GaLu96scf_6_782597_782901	1	0	2	0	0	0	0	GL31170	-	exonic
GaLu96scf_6_822135_822391	2	0	2	0	0	0	0	GL31611	+	exonic
GaLu96scf_6_864126_864411	1	0	2	0	0	0	0	GL31453	-	exonic
GaLu96scf_7_1159515_1160280	3	0	2	0	0	0	0	GL17744	-	exonic
GaLu96scf_7_1177218_1177701	3	0	2	0	0	0	0	GL17746	-	exonic
GaLu96scf_7_1208790_1209091	2	0	0	0	0	2	0	GL17757	-	exonic
GaLu96scf_7_1294553_1295084	4	0	0	0	0	2	0	GL20127	-	exonic
GaLu96scf_7_1350796_1351206	3	0	2	0	0	0	0	GL17761	-	exonic
GaLu96scf_7_1362732_1363143	2	0	2	0	0	0	0	GL28881	+	exonic
GaLu96scf_7_1367812_1368149	1	0	0	0	2	0	0	GL25639	-	exonic
GaLu96scf_7_1441990_1442180	1	0	0	0	0	2	0	GL28890	+	exonic
GaLu96scf_7_1463041_1463287	1	0	2	0	0	0	0	GL28894	-	exonic
GaLu96scf_7_205785_206066	1	0	1	0	0	1	0	GL28915	-	intronic
GaLu96scf_7_207334_207675	1	0	0	0	1	0	1	GL25646	+	exonic
GaLu96scf_7_207334_208830	5	0	0	0	0	0	2	GL25646	+	exonic
GaLu96scf_7_497512_497795	2	2	0	0	0	0	0	GL25586	+	exonic
GaLu96scf_7_522958_523628	2	0	0	0	0	2	0	GL25594	-	exonic
GaLu96scf_7_596888_597263	3	0	0	0	2	0	0	GL25757	-	exonic
GaLu96scf_7_727396_727764	2	2	0	0	0	0	0	GL25667	+	exonic
GaLu96scf_7_776327_777342	5	0	0	2	0	0	0	GL25669	+	exonic
GaLu96scf_7_901352_902575	7	0	0	0	0	0	2	GL25782	-	exonic

GaLu96scf_8_1023680_1023844	1	0	2	0	0	0	0	GL25822	-	exonic
GaLu96scf_8_1077710_1078142	2	0	2	0	0	0	0	GL25948	-	exonic
GaLu96scf_8_1125335_1125888	3	0	2	0	0	0	0	GL25832	+	exonic
GaLu96scf_8_1130885_1131278	4	0	2	0	0	0	0	GL25834	-	exonic
GaLu96scf_8_1155208_1155609	2	2	0	0	0	0	0	GL25954	+	exonic
GaLu96scf_8_1266331_1266546	2	0	0	0	0	0	2	GL26055	+	exonic
GaLu96scf_8_1423364_1423713	3	0	2	0	0	0	0	GL25861	-	exonic
GaLu96scf_8_1482013_1482437	2	0	2	0	0	0	0	GL25968	+	exonic
GaLu96scf_8_1522338_1522623	1	0	2	0	0	0	0	GL29012	-	intronic
GaLu96scf_8_390580_390915	1	0	1	0	0	0	1	GL25882	+	exonic
GaLu96scf_8_459981_460763	4	0	0	0	0	2	0	GL26103	+	exonic
GaLu96scf_8_488801_489086	2	0	0	1	0	1	0	GL29029	-	exonic
GaLu96scf_8_703476_703647	1	0	0	0	0	2	0	GL29044	-	exonic
GaLu96scf_8_866779_867068	2	0	0	0	0	2	0	GL25925	+	exonic
GaLu96scf_8_990820_991014	1	0	0	0	0	2	0	GL25932	+	intronic
GaLu96scf_9_1034945_1035105	1	0	0	0	0	2	0	GL26477	-	intronic
GaLu96scf_9_1036960_1037214	2	2	0	0	0	0	0	GL26477	-	exonic
GaLu96scf_9_1059347_1060594	3	0	0	0	0	2	0	GL18105	+	exonic
GaLu96scf_9_1151810_1153112	3	0	2	0	0	0	0	GL26485	-	exonic
GaLu96scf_9_1237193_1237844	2	0	0	2	0	0	0	GL26494	-	exonic
GaLu96scf_9_1287215_1287962	1	0	0	0	0	0	2	GL26713	+	exonic
GaLu96scf_9_1435059_1435635	3	0	2	0	0	0	0	GL26613	+	exonic
GaLu96scf_9_1484896_1485150	2	0	0	0	0	2	0	GL26514	-	exonic
GaLu96scf_9_177970_178263	1	0	0	0	2	0	0	GL26589	-	intronic
GaLu96scf_9_305743_306731	5	0	0	0	0	0	2	GL26628	-	exonic

GaLu96scf_9_676203_676387	1	0	0	2	0	0	0	GL26643	-	intronic
GaLu96scf_9_910863_911166	2	0	2	0	0	0	0	GL29219	+	exonic
GaLu96scf_9_957206_957503	3	0	0	0	0	0	2	GL18179	+	exonic
GaLu96scf_9_981165_982113	4	0	2	0	0	0	0	GL26582	-	exonic
GaLu96scf_1_1022620_1023054	2	0	0	0	0	0	1	GL30888	+	exonic
GaLu96scf_1_1051568_1051866	3	0	0	0	0	0	1	GL29848	-	exonic
GaLu96scf_1_1098082_1098442	3	0	0	0	0	1	0	GL29849	+	exonic
GaLu96scf_1_1168183_1168736	1	0	0	1	0	0	0	GL30444	-	intronic
GaLu96scf_1_1201072_1202016	3	0	1	0	0	0	0	GL30186	+	exonic
GaLu96scf_1_1319541_1319959	1	0	0	0	0	0	1	GL30195	+	exonic
GaLu96scf_1_1324115_1324364	1	0	0	0	1	0	0	GL30463	-	intronic
GaLu96scf_1_1349226_1349861	1	0	0	1	0	0	0	GL29865	+	intronic
GaLu96scf_1_1427859_1428495	1	0	1	0	0	0	0	GL29877	-	exonic
GaLu96scf_1_14418_14801	3	0	0	0	1	0	0	GL29833	-	exonic
GaLu96scf_1_1461056_1461301	2	0	0	0	0	1	0	GL29874	+	exonic
GaLu96scf_1_1478494_1479724	8	0	1	0	0	0	0	GL29885	-	exonic
GaLu96scf_1_1587853_1588196	1	0	1	0	0	0	0	GL30475	-	exonic
GaLu96scf_1_1739242_1739494	1	0	0	1	0	0	0	GL29907	+	exonic
GaLu96scf_1_1745033_1745513	3	0	0	1	0	0	0	GL30217	+	exonic
GaLu96scf_1_1767527_1767906	2	0	0	0	0	1	0	GL30912	+	exonic
GaLu96scf_1_1832017_1832555	3	0	0	0	0	0	1	GL30917	+	exonic
GaLu96scf_1_2046202_2046987	3	0	1	0	0	0	0	GL30934	-	exonic
GaLu96scf_1_2062031_2062265	2	0	1	0	0	0	0	GL29943	-	exonic
GaLu96scf_1_2105119_2105545	2	0	0	1	0	0	0	GL30248	-	exonic
GaLu96scf_1_223624_224453	4	1	0	0	0	0	0	GL29930	+	exonic

GaLu96scf_1_2416564_2416956	2	0	0	0	1	0	0	GL29975	+	exonic
GaLu96scf_1_2457825_2458227	3	0	0	0	1	0	0	GL30586	-	exonic
GaLu96scf_1_2583158_2583352	2	0	0	0	0	0	1	GL30599	-	exonic
GaLu96scf_1_2753942_2754859	4	0	0	1	0	0	0	GL29999	+	exonic
GaLu96scf_1_3063699_3063973	1	0	1	0	0	0	0	GL30650	+	exonic
GaLu96scf_1_3083115_3083416	3	0	0	0	0	0	1	GL30027	+	exonic
GaLu96scf_1_3122931_3123121	1	0	0	0	1	0	0	GL30319	-	exonic
GaLu96scf_1_3123138_3123296	1	1	0	0	0	0	0	GL30319	-	intronic
GaLu96scf_1_3125046_3125500	3	0	0	1	0	0	0	GL30033	+	exonic
GaLu96scf_1_3139273_3139569	1	0	0	0	1	0	0	GL30038	-	exonic
GaLu96scf_1_3141761_3141999	1	0	0	0	0	1	0	GL30662	+	intronic
GaLu96scf_1_3210453_3211470	2	0	0	0	0	1	0	GL30041	+	exonic
GaLu96scf_1_3383244_3383958	2	0	0	0	1	0	0	GL29474	+	exonic
GaLu96scf_1_3407007_3407419	2	0	0	0	0	0	1	GL30701	-	exonic
GaLu96scf_1_3461522_3461886	2	0	1	0	0	0	0	GL30705	-	exonic
GaLu96scf_1_3517885_3518491	3	0	0	0	0	0	1	GL29481	+	exonic
GaLu96scf_1_3517885_3519158	5	0	0	0	0	0	1	GL29481	+	exonic
GaLu96scf_1_3550020_3550550	3	0	0	0	0	1	0	GL30069	-	exonic
GaLu96scf_1_3734072_3734489	1	0	0	0	0	1	0	GL30736	-	exonic
GaLu96scf_1_3767318_3767642	1	0	0	0	0	0	1	GL30084	-	exonic
GaLu96scf_1_3850733_3851106	2	0	0	0	1	0	0	GL30744	+	exonic
GaLu96scf_1_3851106_3851397	1	0	0	0	1	0	0	GL30744	+	intronic
GaLu96scf_1_3903242_3903592	2	0	0	0	0	1	0	GL31771	+	exonic
GaLu96scf_1_4037375_4037662	1	0	0	0	0	0	1	GL30777	-	exonic
GaLu96scf_1_4109049_4110316	5	0	0	0	1	0	0	GL30111	-	exonic

GaLu96scf_1_4154920_4155163	1	0	0	0	1	0	0	GL30108	+	intronic
GaLu96scf_1_4215237_4215825	2	0	1	0	0	0	0	GL30114	-	exonic
GaLu96scf_1_4219277_4219642	2	0	0	1	0	0	0	GL30115	-	exonic
GaLu96scf_1_441144_441668	3	0	0	1	0	0	0	GL30097	-	exonic
GaLu96scf_1_441534_442564	4	0	1	0	0	0	0	GL30097	-	exonic
GaLu96scf_1_4532729_4533226	2	0	0	0	0	0	1	GL29573	-	exonic
GaLu96scf_1_4532729_4533408	3	0	1	0	0	0	0	GL29573	-	exonic
GaLu96scf_1_4574564_4575062	3	0	0	0	0	1	0	GL29567	-	exonic
GaLu96scf_1_4641233_4642347	4	1	0	0	0	0	0	GL29575	+	exonic
GaLu96scf_1_4651912_4652220	3	0	0	0	0	1	0	GL30139	+	exonic
GaLu96scf_1_4761227_4761471	1	0	0	0	1	0	0	GL29593	-	intronic
GaLu96scf_1_480330_480613	2	0	0	0	0	0	1	GL29518	-	exonic
GaLu96scf_1_4806917_4807338	2	0	0	0	0	1	0	GL31092	-	exonic
GaLu96scf_1_526341_526824	1	0	0	0	1	0	0	GL31102	+	intronic
GaLu96scf_1_55000_55251	1	0	1	0	0	0	0	GL29831	+	exonic
GaLu96scf_1_577199_577434	1	0	1	0	0	0	0	GL30831	-	exonic
GaLu96scf_1_687514_688523	2	0	0	1	0	0	0	GL29620	-	exonic
GaLu96scf_1_744317_745161	4	0	0	1	0	0	0	GL30403	+	exonic
GaLu96scf_1_807086_807416	1	0	0	0	0	1	0	GL30163	+	exonic
GaLu96scf_1_822816_824808	7	0	0	0	0	0	1	GL29633	-	exonic
GaLu96scf_1_834218_834518	1	1	0	0	0	0	0	GL30851	+	intronic
GaLu96scf_1_872394_872739	1	0	1	0	0	0	0	GL30408	+	exonic
GaLu96scf_1_879405_879568	1	0	0	0	0	0	1	GL30855	+	intronic
GaLu96scf_1_96079_96314	1	0	0	1	0	0	0	GL30174	+	intronic
GaLu96scf_1_96079_96319	1	0	0	0	0	1	0	GL30174	+	intronic

GaLu96scf_1_988574_989088	2	0	0	0	1	0	0	GL30867	+	exonic
GaLu96scf_10_1001471_1002017	4	0	0	0	0	0	1	GL15024	+	exonic
GaLu96scf_10_1051143_1051484	1	0	0	0	0	0	1	GL20460	+	exonic
GaLu96scf_10_1240724_1241190	1	0	0	0	0	1	0	GL20473	-	intronic
GaLu96scf_10_1335974_1336291	2	0	0	0	0	0	1	GL20590	-	exonic
GaLu96scf_10_231455_231857	2	0	1	0	0	0	0	GL20598	-	exonic
GaLu96scf_10_339670_340017	2	0	0	0	0	1	0	GL20542	+	exonic
GaLu96scf_10_540957_541282	2	0	0	0	0	0	1	GL20621	+	exonic
GaLu96scf_10_57748_58097	3	0	0	0	1	0	0	GL15004	-	exonic
GaLu96scf_10_64515_64702	1	0	0	0	0	1	0	GL20529	+	intronic
GaLu96scf_10_656443_656704	2	0	0	0	1	0	0	GL20506	-	exonic
GaLu96scf_10_864091_864282	1	0	0	0	0	1	0	GL20641	+	exonic
GaLu96scf_10_935596_935959	1	1	0	0	0	0	0	GL20521	+	exonic
GaLu96scf_10_991858_992050	1	0	0	0	0	0	1	GL20560	+	exonic
GaLu96scf_11_100320_100634	2	0	0	0	1	0	0	GL20660	+	exonic
GaLu96scf_11_1099916_1100405	1	0	1	0	0	0	0	GL20805	-	exonic
GaLu96scf_11_1104547_1105354	3	0	0	0	0	1	0	GL20668	+	exonic
GaLu96scf_11_1145978_1146259	2	0	0	0	0	1	0	GL20744	-	exonic
GaLu96scf_11_125939_126436	1	0	1	0	0	0	0	GL20662	+	intronic
GaLu96scf_11_183914_186176	6	0	0	0	1	0	0	GL20738	+	exonic
GaLu96scf_11_343167_343648	2	0	0	0	1	0	0	GL20693	-	exonic
GaLu96scf_11_432871_433153	1	0	0	0	0	1	0	GL20856	-	exonic
GaLu96scf_11_550080_550507	1	0	0	0	1	0	0	GL20860	+	intronic
GaLu96scf_11_610406_610576	1	0	0	1	0	0	0	GL20868	-	intronic
GaLu96scf_11_699350_700144	2	0	0	0	1	0	0	GL18364	+	exonic

GaLu96scf_11_714343_714664	1	0	0	0	0	1	0	GL20771	-	intronic
GaLu96scf_11_714917_715339	2	0	0	1	0	0	0	GL20771	-	exonic
GaLu96scf_11_715123_715339	1	0	0	0	0	0	1	GL20771	-	exonic
GaLu96scf_11_798423_798658	1	0	0	0	0	1	0	GL20726	-	intronic
GaLu96scf_11_88460_88892	2	0	0	0	0	0	1	GL18287	-	exonic
GaLu96scf_11_908140_908955	2	0	0	0	0	1	0	GL18382	-	exonic
GaLu96scf_12_1077467_1077895	1	0	1	0	0	0	0	GL20980	+	exonic
GaLu96scf_12_1124455_1125337	3	0	1	0	0	0	0	GL21038	+	exonic
GaLu96scf_12_1124455_1125519	4	0	0	0	0	1	0	GL21038	+	exonic
GaLu96scf_12_184028_184418	2	0	0	0	0	0	1	GL21030	-	exonic
GaLu96scf_12_201364_201773	2	0	0	0	0	0	1	GL20911	-	exonic
GaLu96scf_12_358886_359047	1	1	0	0	0	0	0	GL27036	+	exonic
GaLu96scf_12_406586_406748	1	0	0	0	0	0	1	GL21003	+	exonic
GaLu96scf_12_456938_457309	2	0	0	0	0	1	0	GL18438	+	exonic
GaLu96scf_12_672275_672658	2	0	0	1	0	0	0	GL21068	-	exonic
GaLu96scf_12_757741_758109	1	0	0	0	0	0	1	GL21071	+	exonic
GaLu96scf_12_800728_800908	1	0	0	0	0	1	0	GL15336	-	intronic
GaLu96scf_12_809983_810343	2	0	0	1	0	0	0	GL20955	-	exonic
GaLu96scf_12_920457_920630	1	0	0	0	1	0	0	GL21013	-	intronic
GaLu96scf_12_936751_937355	1	0	1	0	0	0	0	GL21087	-	exonic
GaLu96scf_12_938199_938675	1	0	0	1	0	0	0	GL21087	-	intronic
GaLu96scf_12_956568_957270	1	0	0	1	0	0	0	GL21086	+	exonic
GaLu96scf_12_996661_997041	2	0	0	0	0	1	0	GL15273	-	exonic
GaLu96scf_13_1002778_1003046	2	0	0	0	0	1	0	GL21206	+	exonic
GaLu96scf_13_1035832_1036700	2	0	0	0	1	0	0	GL21164	-	exonic

GaLu96scf_13_1077937_1078212	1	1	0	0	0	0	0	GL21210	+	exonic
GaLu96scf_13_1094212_1094578	2	0	1	0	0	0	0	GL15367	-	exonic
GaLu96scf_13_1103920_1104490	2	0	0	0	0	1	0	GL21214	+	exonic
GaLu96scf_13_1186000_1186308	2	0	0	0	0	1	0	GL21111	-	exonic
GaLu96scf_13_1191550_1191848	1	0	0	1	0	0	0	GL21218	-	intronic
GaLu96scf_13_299638_300034	2	0	0	0	1	0	0	GL21224	-	exonic
GaLu96scf_13_299794_300034	1	0	1	0	0	0	0	GL21224	-	exonic
GaLu96scf_13_360765_361149	2	0	0	0	0	0	1	GL18490	+	exonic
GaLu96scf_13_502018_502275	1	0	0	0	1	0	0	GL15426	-	intronic
GaLu96scf_13_598428_598963	3	0	0	0	0	0	1	GL21190	-	exonic
GaLu96scf_13_67467_67663	1	0	1	0	0	0	0	GL21093	+	exonic
GaLu96scf_13_711903_712174	2	0	0	0	0	0	1	GL15437	+	exonic
GaLu96scf_13_719131_719439	3	0	0	0	1	0	0	GL15438	+	exonic
GaLu96scf_13_91326_91598	1	0	0	1	0	0	0	GL15348	-	exonic
GaLu96scf_13_976810_977053	1	0	0	0	0	1	0	GL21270	-	exonic
GaLu96scf_14_1000040_1001025	3	0	0	1	0	0	0	GL21283	-	exonic
GaLu96scf_14_1069126_1069454	1	0	0	1	0	0	0	GL21282	+	exonic
GaLu96scf_14_1070963_1071347	1	0	0	0	0	1	0	GL21333	+	intronic
GaLu96scf_14_120234_120581	1	0	0	0	0	1	0	GL21384	+	exonic
GaLu96scf_14_126501_126728	1	0	0	0	0	1	0	GL21387	-	intronic
GaLu96scf_14_269088_269255	1	0	0	1	0	0	0	GL27169	+	exonic
GaLu96scf_14_294888_295479	2	0	0	1	0	0	0	GL18545	+	exonic
GaLu96scf_14_340073_340860	2	0	0	0	1	0	0	GL15489	+	exonic
GaLu96scf_14_340584_340860	1	0	0	0	0	1	0	GL15489	+	exonic
GaLu96scf_14_340860_341136	1	0	0	0	0	1	0	GL15489	+	intronic

GaLu96scf_14_353431_353957	2	0	0	1	0	0	0	GL21346	+	exonic
GaLu96scf_14_46375_46574	1	0	0	0	0	0	1	GL21272	-	intronic
GaLu96scf_14_470047_470611	3	0	1	0	0	0	0	GL21417	-	exonic
GaLu96scf_14_552517_552747	2	0	0	0	0	0	1	GL31755	-	exonic
GaLu96scf_14_626976_627294	1	0	0	0	1	0	0	GL21430	-	exonic
GaLu96scf_14_722703_723639	4	0	1	0	0	0	0	GL21438	-	exonic
GaLu96scf_14_76181_76366	1	0	1	0	0	0	0	GL15449	+	intronic
GaLu96scf_14_829963_830304	2	0	1	0	0	0	0	GL21364	+	exonic
GaLu96scf_14_836124_836543	1	0	0	0	1	0	0	GL21444	+	exonic
GaLu96scf_14_836602_837518	3	0	0	0	1	0	0	GL21444	+	exonic
GaLu96scf_14_868258_869140	4	0	0	1	0	0	0	GL21368	-	exonic
GaLu96scf_14_87845_88236	1	0	0	0	1	0	0	GL18533	-	exonic
GaLu96scf_14_886519_886708	1	0	0	0	0	1	0	GL21366	+	exonic
GaLu96scf_14_895159_895983	2	0	0	0	1	0	0	GL21322	-	exonic
GaLu96scf_14_912475_912865	2	0	0	0	1	0	0	GL21457	-	exonic
GaLu96scf_14_978372_978709	1	0	0	0	1	0	0	GL27220	+	intronic
GaLu96scf_15_1002028_1002633	3	0	0	0	0	0	1	GL21469	-	exonic
GaLu96scf_15_164699_165283	4	1	0	0	0	0	0	GL27226	-	exonic
GaLu96scf_15_180233_180491	1	0	0	0	0	1	0	GL15546	-	exonic
GaLu96scf_15_433885_434304	1	0	0	0	0	1	0	GL21536	+	intronic
GaLu96scf_15_495640_496094	2	0	0	0	0	0	1	GL21538	-	exonic
GaLu96scf_15_633635_634004	1	0	0	0	0	1	0	GL27256	-	exonic
GaLu96scf_15_719431_720049	3	0	0	1	0	0	0	GL21545	-	exonic
GaLu96scf_15_82990_83598	2	0	1	0	0	0	0	GL21525	-	exonic
GaLu96scf_15_894471_894632	1	0	0	0	0	0	1	GL21512	+	intronic

GaLu96scf_15_988437_989128	1	0	0	0	0	1	0	GL21619	-	intronic
GaLu96scf_16_104022_104377	3	0	0	0	0	1	0	GL21682	+	exonic
GaLu96scf_16_193371_193692	2	0	1	0	0	0	0	GL21626	+	exonic
GaLu96scf_16_251463_252081	2	1	0	0	0	0	0	GL15656	-	exonic
GaLu96scf_16_312675_313512	3	0	1	0	0	0	0	GL21744	-	exonic
GaLu96scf_16_312928_313887	4	0	0	0	0	0	1	GL21744	-	exonic
GaLu96scf_16_313804_314549	4	0	0	1	0	0	0	GL21744	-	exonic
GaLu96scf_16_314176_314549	2	0	0	0	0	0	1	GL21744	-	exonic
GaLu96scf_16_322297_322719	3	0	0	0	0	0	1	GL21739	+	exonic
GaLu96scf_16_36126_36437	1	0	0	0	0	1	0	GL21679	+	intronic
GaLu96scf_16_375080_375362	1	0	0	1	0	0	0	GL15668	+	exonic
GaLu96scf_16_375461_375677	1	0	0	0	1	0	0	GL15668	+	exonic
GaLu96scf_16_375461_376544	3	0	0	0	1	0	0	GL15668	+	exonic
GaLu96scf_16_463359_463766	1	0	0	0	0	1	0	GL21690	-	intronic
GaLu96scf_16_479394_479577	1	0	0	0	1	0	0	GL21755	-	intronic
GaLu96scf_16_544486_545121	1	1	0	0	0	0	0	GL18694	+	intronic
GaLu96scf_16_549145_549300	1	0	0	0	1	0	0	GL21766	-	exonic
GaLu96scf_16_577505_577728	1	0	0	0	0	0	1	GL15678	-	intronic
GaLu96scf_16_647368_647529	1	0	0	0	0	0	1	GL21771	-	exonic
GaLu96scf_16_688683_689186	1	0	0	1	0	0	0	GL18702	+	intronic
GaLu96scf_16_744501_744869	2	0	0	0	1	0	0	GL21660	-	exonic
GaLu96scf_16_833844_834113	1	0	0	1	0	0	0	GL21710	-	intronic
GaLu96scf_16_856076_856605	3	0	0	1	0	0	0	GL15699	-	exonic
GaLu96scf_16_962229_962893	3	0	0	0	1	0	0	GL21717	-	exonic
GaLu96scf_16_972866_973152	2	0	0	1	0	0	0	GL21673	+	exonic

GaLu96scf_17_257434_257824	1	1	0	0	0	0	0	GL21862	-	exonic
GaLu96scf_17_265084_265533	2	0	0	0	0	1	0	GL18743	-	exonic
GaLu96scf_17_310040_310315	2	0	0	0	1	0	0	GL15753	-	exonic
GaLu96scf_17_335371_335566	1	0	0	0	0	1	0	GL21924	-	exonic
GaLu96scf_17_354409_354918	2	0	0	0	0	0	1	GL21821	-	exonic
GaLu96scf_17_482106_482368	1	0	1	0	0	0	0	GL15755	+	exonic
GaLu96scf_17_548716_549290	2	0	0	1	0	0	0	GL21944	-	exonic
GaLu96scf_17_630022_630493	2	0	0	0	0	0	1	GL21838	-	exonic
GaLu96scf_17_664828_665119	2	0	1	0	0	0	0	GL21882	-	exonic
GaLu96scf_17_664828_665303	3	0	1	0	0	0	0	GL21882	-	exonic
GaLu96scf_17_674441_674600	1	0	1	0	0	0	0	GL21954	-	intronic
GaLu96scf_17_715388_715580	1	0	1	0	0	0	0	GL21956	-	intronic
GaLu96scf_17_792475_792808	1	0	0	1	0	0	0	GL21844	+	intronic
GaLu96scf_17_861050_861664	1	0	0	0	1	0	0	GL21966	-	intronic
GaLu96scf_18_150624_150983	1	1	0	0	0	0	0	GL22045	-	intronic
GaLu96scf_18_188754_188962	1	0	0	0	0	0	1	GL21984	+	exonic
GaLu96scf_18_251927_252232	2	0	0	0	0	0	1	GL21992	-	exonic
GaLu96scf_18_263111_263670	4	0	0	0	0	1	0	GL18813	-	exonic
GaLu96scf_18_381958_382504	1	0	0	0	0	0	1	GL21997	-	intronic
GaLu96scf_18_411488_411923	3	0	0	0	0	0	1	GL22001	+	exonic
GaLu96scf_18_432723_433059	1	0	0	0	0	0	1	GL22003	+	exonic
GaLu96scf_18_621102_621839	4	0	0	0	1	0	0	GL22024	-	exonic
GaLu96scf_18_621320_621839	3	0	1	0	0	0	0	GL22024	-	exonic
GaLu96scf_19_106421_106770	1	1	0	0	0	0	0	GL22137	-	intronic
GaLu96scf_19_113299_113660	1	0	0	1	0	0	0	GL22245	+	intronic

GaLu96scf_19_256680_257199	3	0	0	1	0	0	0	GL22189	-	exonic
GaLu96scf_19_389593_390075	3	0	1	0	0	0	0	GL22268	-	exonic
GaLu96scf_19_560789_561026	1	0	0	0	0	0	1	GL22208	+	intronic
GaLu96scf_19_560789_561051	1	0	0	0	0	0	1	GL22208	+	intronic
GaLu96scf_19_682545_683129	1	0	0	1	0	0	0	GL15946	-	exonic
GaLu96scf_19_687191_687541	2	0	0	0	0	1	0	GL18881	-	exonic
GaLu96scf_19_730538_730692	1	0	0	0	0	1	0	GL22223	+	intronic
GaLu96scf_19_75161_75416	1	0	0	1	0	0	0	GL22235	+	intronic
GaLu96scf_19_81561_81814	1	0	0	1	0	0	0	GL22132	-	intronic
GaLu96scf_19_868555_868969	2	0	1	0	0	0	0	GL22180	-	exonic
GaLu96scf_2_1035390_1035778	2	0	0	0	0	0	1	GL22327	-	exonic
GaLu96scf_2_1177722_1177901	1	1	0	0	0	0	0	GL22549	+	exonic
GaLu96scf_2_1238164_1238581	3	0	0	1	0	0	0	GL22552	+	exonic
GaLu96scf_2_1251586_1251752	1	0	0	0	1	0	0	GL22555	+	intronic
GaLu96scf_2_1297860_1299215	6	1	0	0	0	0	0	GL16020	+	exonic
GaLu96scf_2_1337037_1337825	5	0	0	0	1	0	0	GL22470	+	exonic
GaLu96scf_2_1501802_1502294	1	0	1	0	0	0	0	GL22568	+	exonic
GaLu96scf_2_1544313_1544511	1	0	0	1	0	0	0	GL22569	+	intronic
GaLu96scf_2_1597429_1597916	2	1	0	0	0	0	0	GL16049	+	exonic
GaLu96scf_2_1770102_1770882	4	0	0	0	1	0	0	GL22592	-	exonic
GaLu96scf_2_2104852_2105020	1	0	0	0	0	1	0	GL22393	+	intronic
GaLu96scf_2_2124181_2124489	1	0	0	0	1	0	0	GL22615	+	intronic
GaLu96scf_2_2184361_2184856	4	0	0	0	0	1	0	GL22396	+	exonic
GaLu96scf_2_2213163_2213490	1	0	1	0	0	0	0	GL22627	-	intronic
GaLu96scf_2_663179_663567	1	0	0	1	0	0	0	GL22656	-	intronic

GaLu96scf_2_755936_756119	1	0	0	0	0	0	1	GL22433	+	exonic
GaLu96scf_2_79752_80226	1	1	0	0	0	0	0	GL27487	+	exonic
GaLu96scf_2_802905_803128	1	0	0	0	0	1	0	GL22670	-	exonic
GaLu96scf_2_857613_857952	1	0	1	0	0	0	0	GL22672	-	intronic
GaLu96scf_2_977213_977848	3	0	0	0	0	0	1	GL16173	+	exonic
GaLu96scf_20_142969_143191	1	0	0	0	0	0	1	GL22680	+	exonic
GaLu96scf_20_277827_278019	1	0	0	0	0	0	1	GL22709	+	exonic
GaLu96scf_20_279184_279457	2	0	0	0	0	0	1	GL22736	-	exonic
GaLu96scf_20_345879_346625	2	0	0	0	0	1	0	GL27668	-	exonic
GaLu96scf_20_507012_507274	1	0	0	0	0	1	0	GL16234	+	exonic
GaLu96scf_20_579568_579831	1	0	0	0	1	0	0	GL22718	+	intronic
GaLu96scf_20_73587_73946	2	0	1	0	0	0	0	GL22679	+	exonic
GaLu96scf_21_294285_294966	3	0	0	0	1	0	0	GL22792	-	exonic
GaLu96scf_21_370616_370950	1	0	0	1	0	0	0	GL22878	+	intronic
GaLu96scf_21_411672_412071	3	0	0	0	1	0	0	GL27743	+	exonic
GaLu96scf_21_442699_443029	3	0	0	0	0	1	0	GL22885	+	exonic
GaLu96scf_21_457469_458175	4	0	0	0	0	1	0	GL31715	-	exonic
GaLu96scf_21_513955_514272	1	0	0	0	1	0	0	GL22842	+	intronic
GaLu96scf_21_562582_562743	1	0	0	0	1	0	0	GL16341	-	intronic
GaLu96scf_21_678667_678866	2	0	0	0	0	1	0	GL22818	-	exonic
GaLu96scf_21_706994_707285	1	0	0	0	1	0	0	GL22903	-	intronic
GaLu96scf_21_724120_724413	1	0	0	1	0	0	0	GL22820	-	intronic
GaLu96scf_21_759169_759413	1	0	0	0	0	1	0	GL22902	+	intronic
GaLu96scf_22_124307_124704	1	0	0	1	0	0	0	GL22915	-	intronic
GaLu96scf_22_188017_188188	1	0	1	0	0	0	0	GL22966	-	exonic

GaLu96scf_22_188017_188634	2	0	0	1	0	0	0	GL22966	-	exonic
GaLu96scf_22_274908_275127	1	0	0	0	0	0	1	GL16392	+	intronic
GaLu96scf_22_318923_319217	2	0	0	0	1	0	0	GL19134	-	exonic
GaLu96scf_22_403759_404534	4	0	1	0	0	0	0	GL22932	-	exonic
GaLu96scf_22_587845_588196	2	0	1	0	0	0	0	GL22936	+	exonic
GaLu96scf_22_686708_687026	1	0	0	1	0	0	0	GL19179	+	exonic
GaLu96scf_22_775066_775273	1	0	0	0	1	0	0	GL23027	-	exonic
GaLu96scf_22_788338_788870	3	0	0	1	0	0	0	GL16429	+	exonic
GaLu96scf_23_150973_151124	1	0	0	0	0	1	0	GL23076	-	intronic
GaLu96scf_23_197130_198088	2	0	0	0	1	0	0	GL23080	-	exonic
GaLu96scf_23_280609_280862	2	0	0	1	0	0	0	GL23107	-	exonic
GaLu96scf_23_555442_555654	1	0	0	0	1	0	0	GL23085	-	intronic
GaLu96scf_23_56480_57117	3	0	0	0	0	1	0	GL19197	+	exonic
GaLu96scf_23_576351_577209	4	0	0	0	0	1	0	GL27844	-	exonic
GaLu96scf_23_634186_634396	1	0	0	1	0	0	0	GL23068	-	intronic
GaLu96scf_23_73825_74248	2	0	0	1	0	0	0	GL16436	+	exonic
GaLu96scf_24_123224_123543	1	0	0	0	1	0	0	GL23179	+	exonic
GaLu96scf_24_275334_275631	1	0	0	0	0	1	0	GL23141	+	intronic
GaLu96scf_24_33643_34346	2	0	0	0	0	0	1	GL23132	+	exonic
GaLu96scf_24_347084_347487	3	0	0	0	0	0	1	GL16520	-	exonic
GaLu96scf_24_87252_88358	6	0	0	0	1	0	0	GL23174	-	exonic
GaLu96scf_25_426165_427245	5	0	0	0	0	1	0	GL31764	+	exonic
GaLu96scf_26_132391_132670	2	0	0	0	1	0	0	GL23355	-	exonic
GaLu96scf_26_149025_149311	2	1	0	0	0	0	0	GL23300	+	exonic
GaLu96scf_26_154904_155440	1	0	0	0	1	0	0	GL23350	+	exonic

GaLu96scf_26_446952_447744	1	0	0	0	0	0	1	GL23341	+	intronic
GaLu96scf_26_483750_484356	3	0	0	1	0	0	0	GL23374	-	exonic
GaLu96scf_26_536937_537166	2	0	0	0	0	1	0	GL16673	+	exonic
GaLu96scf_27_127851_128033	1	0	1	0	0	0	0	GL23383	+	intronic
GaLu96scf_27_318548_318775	1	0	0	0	1	0	0	GL23418	-	intronic
GaLu96scf_27_366457_366866	3	0	0	0	1	0	0	GL23395	-	exonic
GaLu96scf_27_502128_502381	1	0	1	0	0	0	0	GL23466	-	intronic
GaLu96scf_27_527876_528053	1	0	0	0	1	0	0	GL23405	+	exonic
GaLu96scf_28_279749_280049	2	0	0	1	0	0	0	GL23477	+	exonic
GaLu96scf_28_28458_28835	3	0	0	0	0	0	1	GL28065	+	exonic
GaLu96scf_28_39612_40185	3	0	0	1	0	0	0	GL19382	-	exonic
GaLu96scf_28_430888_431088	1	0	1	0	0	0	0	GL23488	-	exonic
GaLu96scf_28_501725_502375	2	0	0	0	1	0	0	GL23507	+	exonic
GaLu96scf_29_121333_121780	3	0	0	1	0	0	0	GL23554	+	exonic
GaLu96scf_29_175846_176123	1	0	0	0	0	0	1	GL23580	+	exonic
GaLu96scf_29_249130_249358	1	0	0	0	0	1	0	GL23608	-	exonic
GaLu96scf_29_307238_307405	1	0	0	0	0	1	0	GL19429	-	intronic
GaLu96scf_29_307671_307865	1	0	0	1	0	0	0	GL19429	-	exonic
GaLu96scf_29_346438_346649	1	0	0	0	0	1	0	GL23586	+	exonic
GaLu96scf_29_441073_441605	1	0	0	1	0	0	0	GL19436	-	exonic
GaLu96scf_3_1099343_1100168	2	0	0	0	0	0	1	GL23651	-	exonic
GaLu96scf_3_1099558_1099748	1	0	0	1	0	0	0	GL23651	-	intronic
GaLu96scf_3_1117008_1117243	1	0	0	1	0	0	0	GL23645	+	exonic
GaLu96scf_3_1180362_1181582	2	0	1	0	0	0	0	GL23655	-	exonic
GaLu96scf_3_1218035_1218333	2	0	0	0	1	0	0	GL23656	+	exonic

GaLu96scf_3_126852_127125	1	0	0	0	0	1	0	GL23856	+	exonic
GaLu96scf_3_1279545_1280311	3	0	0	0	0	0	1	GL28147	-	exonic
GaLu96scf_3_1279877_1280046	1	0	0	0	0	0	1	GL28147	-	exonic
GaLu96scf_3_1314243_1315185	6	0	0	1	0	0	0	GL16852	-	exonic
GaLu96scf_3_1338202_1338773	1	0	1	0	0	0	0	GL23886	+	exonic
GaLu96scf_3_1857838_1858067	1	0	0	0	0	1	0	GL23703	-	exonic
GaLu96scf_3_1898905_1899231	1	0	0	0	0	1	0	GL23921	+	intronic
GaLu96scf_3_1899568_1899773	1	0	0	0	0	0	1	GL23921	+	intronic
GaLu96scf_3_1926382_1926600	1	0	0	0	0	0	1	GL23823	+	exonic
GaLu96scf_3_220458_220912	2	0	0	0	1	0	0	GL31720	+	exonic
GaLu96scf_3_221040_221312	1	0	0	0	0	0	1	GL31720	+	exonic
GaLu96scf_3_223378_223889	2	0	0	0	1	0	0	GL31721	+	exonic
GaLu96scf_3_495172_495826	5	0	0	0	1	0	0	GL23731	-	exonic
GaLu96scf_3_495382_495826	3	0	0	0	1	0	0	GL23731	-	exonic
GaLu96scf_3_558841_559401	3	0	1	0	0	0	0	GL23740	-	exonic
GaLu96scf_3_574673_574924	2	0	0	0	0	0	1	GL23742	-	exonic
GaLu96scf_3_586294_586516	1	0	1	0	0	0	0	GL23736	+	intronic
GaLu96scf_3_605030_605284	2	0	0	0	0	1	0	GL23743	+	exonic
GaLu96scf_3_646715_647119	3	0	0	0	0	1	0	GL28211	-	exonic
GaLu96scf_3_832775_833150	2	0	1	0	0	0	0	GL23769	+	exonic
GaLu96scf_3_835590_835889	2	0	0	0	0	1	0	GL16923	-	exonic
GaLu96scf_3_962695_963289	2	0	0	0	0	0	1	GL23776	-	exonic
GaLu96scf_3_997583_998062	3	0	0	0	0	0	1	GL16827	+	exonic
GaLu96scf_30_147614_147791	1	0	0	0	1	0	0	GL28261	-	intronic
GaLu96scf_30_207368_207928	3	0	0	0	0	1	0	GL23995	-	exonic

GaLu96scf_30_396932_397418	2	0	0	0	0	0	1	GL24002	+	exonic
GaLu96scf_30_57146_58623	7	0	1	0	0	0	0	GL24022	-	exonic
GaLu96scf_30_57146_58820	8	0	1	0	0	0	0	GL24022	-	exonic
GaLu96scf_30_58487_58820	2	0	0	0	0	1	0	GL24022	-	exonic
GaLu96scf_31_178594_178809	1	0	0	1	0	0	0	GL24053	-	exonic
GaLu96scf_31_253189_253504	1	0	0	0	1	0	0	GL24085	+	intronic
GaLu96scf_31_27644_27912	1	0	0	0	0	0	1	GL24035	+	exonic
GaLu96scf_31_287068_287448	2	0	0	0	0	0	1	GL17002	-	exonic
GaLu96scf_31_290588_290754	1	0	0	0	1	0	0	GL24058	+	intronic
GaLu96scf_31_309685_310046	1	0	0	1	0	0	0	GL24062	+	exonic
GaLu96scf_31_361318_361941	2	0	0	0	0	0	1	GL17007	-	exonic
GaLu96scf_31_361449_361696	1	0	0	0	1	0	0	GL17007	-	intronic
GaLu96scf_31_361458_361696	1	0	0	0	1	0	0	GL17007	-	intronic
GaLu96scf_31_430223_431842	8	0	0	0	0	0	1	GL24072	+	exonic
GaLu96scf_31_435742_439709	10	0	0	0	1	0	0	GL24130	+	exonic
GaLu96scf_31_53347_53557	1	0	0	0	0	1	0	GL24097	+	intronic
GaLu96scf_31_72669_73684	4	0	0	0	0	1	0	GL24037	+	exonic
GaLu96scf_31_88837_89129	2	0	0	1	0	0	0	GL24038	+	exonic
GaLu96scf_32_131663_132041	1	0	1	0	0	0	0	GL24166	+	intronic
GaLu96scf_32_167826_168525	2	0	0	1	0	0	0	GL24162	+	exonic
GaLu96scf_32_182781_183194	1	0	1	0	0	0	0	GL24163	+	exonic
GaLu96scf_32_312513_312758	1	0	0	1	0	0	0	GL24157	-	intronic
GaLu96scf_32_331602_332981	5	0	0	1	0	0	0	GL24152	+	exonic
GaLu96scf_32_337194_338607	1	0	0	1	0	0	0	GL24194	-	exonic
GaLu96scf_33_112982_113199	1	0	0	0	0	0	1	GL24201	-	exonic

GaLu96scf_33_205403_205934	4	0	1	0	0	0	0	GL24216	+	exonic
GaLu96scf_34_127114_127406	1	0	1	0	0	0	0	GL24243	+	exonic
GaLu96scf_34_188909_189328	2	0	0	0	1	0	0	GL24273	-	exonic
GaLu96scf_34_224443_224864	2	0	0	0	1	0	0	GL24253	+	exonic
GaLu96scf_34_259203_259693	1	0	0	0	1	0	0	GL24297	-	intronic
GaLu96scf_34_30502_30896	1	1	0	0	0	0	0	GL28361	-	exonic
GaLu96scf_34_336024_337659	7	0	1	0	0	0	0	GL24263	-	exonic
GaLu96scf_34_45294_45907	3	0	0	1	0	0	0	GL24242	-	exonic
GaLu96scf_34_45294_46255	4	0	0	1	0	0	0	GL24242	-	exonic
GaLu96scf_34_58949_59214	1	0	0	0	0	0	1	GL24283	+	exonic
GaLu96scf_35_310937_311352	2	0	0	0	0	1	0	GL24311	+	exonic
GaLu96scf_35_311434_311815	2	0	0	1	0	0	0	GL24311	+	exonic
GaLu96scf_35_350223_350515	2	0	1	0	0	0	0	GL24312	+	exonic
GaLu96scf_35_67311_67925	6	0	0	1	0	0	0	GL17141	-	exonic
GaLu96scf_35_76588_77089	3	0	0	0	0	1	0	GL28383	-	exonic
GaLu96scf_36_74533_75102	1	0	0	0	0	0	1	GL29257	-	exonic
GaLu96scf_37_94304_94612	1	0	0	0	1	0	0	GL24367	+	exonic
GaLu96scf_39_134081_134900	1	0	0	0	0	1	0	GL24433	+	intronic
GaLu96scf_39_134081_134902	1	0	0	0	1	0	0	GL24433	+	intronic
GaLu96scf_39_56368_56625	1	0	0	1	0	0	0	GL17207	+	exonic
GaLu96scf_4_1068703_1069116	3	0	1	0	0	0	0	GL17247	+	exonic
GaLu96scf_4_1146831_1147097	1	0	0	0	0	0	1	GL24489	+	exonic
GaLu96scf_4_1266072_1266428	1	0	1	0	0	0	0	GL24642	-	exonic
GaLu96scf_4_1317866_1318323	4	1	0	0	0	0	0	GL24503	+	exonic
GaLu96scf_4_1339336_1339717	2	0	0	0	0	1	0	GL24645	-	exonic

GaLu96scf_4_1459669_1460308	3	0	0	0	0	0	1	GL17270	+	exonic
GaLu96scf_4_1565519_1565823	1	0	0	0	1	0	0	GL28504	+	exonic
GaLu96scf_4_1567685_1568247	3	0	0	0	0	0	1	GL24515	+	exonic
GaLu96scf_4_1632667_1632989	2	0	1	0	0	0	0	GL17290	-	exonic
GaLu96scf_4_168642_169034	2	0	0	0	0	1	0	GL28477	+	exonic
GaLu96scf_4_168873_169400	2	0	0	1	0	0	0	GL28477	+	exonic
GaLu96scf_4_1740690_1741143	2	0	0	0	0	0	1	GL17299	+	exonic
GaLu96scf_4_1879635_1880507	3	0	0	0	1	0	0	GL28524	+	exonic
GaLu96scf_4_226425_226589	2	0	0	0	1	0	0	GL24546	-	exonic
GaLu96scf_4_228229_229023	4	0	0	1	0	0	0	GL24817	+	exonic
GaLu96scf_4_349000_349315	2	0	1	0	0	0	0	GL24826	+	exonic
GaLu96scf_4_376373_376587	1	0	0	0	1	0	0	GL24553	-	exonic
GaLu96scf_4_408318_409324	1	0	0	0	0	1	0	GL24672	-	exonic
GaLu96scf_4_422602_422972	3	0	0	1	0	0	0	GL17345	+	exonic
GaLu96scf_4_435074_435741	2	0	0	0	1	0	0	GL19811	+	exonic
GaLu96scf_4_546220_546831	2	0	1	0	0	0	0	GL24681	-	exonic
GaLu96scf_4_553538_554648	2	0	0	0	0	1	0	GL24683	-	exonic
GaLu96scf_4_554664_554910	1	0	0	0	0	0	1	GL24683	-	intronic
GaLu96scf_4_57355_57903	3	0	0	0	0	0	1	GL24457	+	exonic
GaLu96scf_4_619223_619652	2	0	1	0	0	0	0	GL24851	+	exonic
GaLu96scf_4_6272_6665	1	0	0	0	1	0	0	GL24715	+	exonic
GaLu96scf_4_63989_64320	2	0	0	0	0	1	0	GL24621	+	exonic
GaLu96scf_4_667456_667663	1	0	0	0	0	1	0	GL24855	-	intronic
GaLu96scf_4_716722_717017	1	0	0	1	0	0	0	GL24688	+	exonic
GaLu96scf_4_738085_738381	1	0	1	0	0	0	0	GL24586	+	intronic

GaLu96scf_4_753244_753802	2	0	0	0	0	0	1	GL24693	-	exonic
GaLu96scf_4_830825_831108	2	0	1	0	0	0	0	GL24602	-	exonic
GaLu96scf_4_832796_833137	2	0	0	0	1	0	0	GL24702	-	exonic
GaLu96scf_4_941524_942085	1	0	0	0	0	0	1	GL17366	+	intronic
GaLu96scf_4_984554_984792	1	0	0	0	1	0	0	GL24708	+	exonic
GaLu96scf_4_988632_988869	2	0	0	0	0	0	1	GL24619	-	exonic
GaLu96scf_40_33822_34531	3	0	0	0	0	0	1	GL24898	-	exonic
GaLu96scf_41_18285_18642	1	0	0	0	0	0	1	GL24922	+	exonic
GaLu96scf_43_116516_116876	1	0	0	0	1	0	0	GL28638	+	intronic
GaLu96scf_43_58185_58576	1	0	0	1	0	0	0	GL28626	-	exonic
GaLu96scf_44_81238_81514	1	0	0	0	1	0	0	GL17453	+	intronic
GaLu96scf_44_97373_97546	1	0	0	0	0	0	1	GL25033	-	intronic
GaLu96scf_45_134109_134855	3	0	0	0	0	1	0	GL25065	+	exonic
GaLu96scf_45_134269_134540	1	0	1	0	0	0	0	GL25065	+	exonic
GaLu96scf_45_156736_157243	4	0	0	0	0	1	0	GL25078	+	exonic
GaLu96scf_45_158125_158782	3	0	0	1	0	0	0	GL25078	+	exonic
GaLu96scf_45_26854_27149	1	0	0	0	1	0	0	GL25048	+	exonic
GaLu96scf_46_125330_125501	1	0	0	0	0	0	1	GL17498	-	exonic
GaLu96scf_46_55071_55230	1	0	0	0	1	0	0	GL25094	-	exonic
GaLu96scf_46_58490_59354	3	0	0	1	0	0	0	GL25090	+	exonic
GaLu96scf_47_37276_37444	1	0	0	1	0	0	0	GL28679	-	intronic
GaLu96scf_47_37444_37728	1	0	0	0	0	0	1	GL28679	-	exonic
GaLu96scf_47_71790_72960	6	0	0	0	0	1	0	GL25110	-	exonic
GaLu96scf_47_90451_90941	3	0	1	0	0	0	0	GL25106	+	exonic
GaLu96scf_48_60545_60792	2	0	1	0	0	0	0	GL25128	+	exonic

GaLu96scf_48_90717_91060	2	1	0	0	0	0	0	GL25127	-	exonic
GaLu96scf_49_50406_50649	1	0	0	0	1	0	0	GL25138	-	exonic
GaLu96scf_49_55728_56245	3	0	1	0	0	0	0	GL28692	+	exonic
GaLu96scf_5_1092624_1092795	1	0	0	0	0	1	0	GL25357	-	intronic
GaLu96scf_5_1092625_1092795	1	0	0	0	1	0	0	GL25357	-	intronic
GaLu96scf_5_1100364_1100968	2	0	0	0	0	0	1	GL17543	+	exonic
GaLu96scf_5_1117503_1117783	1	0	0	0	0	0	1	GL25171	+	exonic
GaLu96scf_5_1142068_1142814	1	0	0	0	0	1	0	GL25363	-	exonic
GaLu96scf_5_1144051_1144540	2	0	0	1	0	0	0	GL17541	+	exonic
GaLu96scf_5_1254705_1255305	2	0	1	0	0	0	0	GL25376	-	exonic
GaLu96scf_5_1256724_1256931	1	0	0	0	0	1	0	GL28715	-	intronic
GaLu96scf_5_1397507_1397914	1	0	1	0	0	0	0	GL17563	-	exonic
GaLu96scf_5_1397507_1398132	2	0	0	0	0	0	1	GL17563	-	exonic
GaLu96scf_5_1402011_1402230	1	0	0	0	0	1	0	GL17558	+	intronic
GaLu96scf_5_1478381_1478658	1	0	1	0	0	0	0	GL25186	+	exonic
GaLu96scf_5_1626995_1627367	4	0	0	0	1	0	0	GL25203	-	exonic
GaLu96scf_5_276592_276854	1	0	0	0	0	1	0	GL25408	+	exonic
GaLu96scf_5_428692_429138	3	0	1	0	0	0	0	GL25425	-	exonic
GaLu96scf_5_458925_459211	2	0	0	0	0	1	0	GL25428	-	exonic
GaLu96scf_5_471914_472442	2	0	0	1	0	0	0	GL25226	+	exonic
GaLu96scf_5_521739_522697	2	0	0	1	0	0	0	GL25231	+	exonic
GaLu96scf_5_522440_522697	1	0	0	0	1	0	0	GL25231	+	exonic
GaLu96scf_5_540190_540464	1	0	1	0	0	0	0	GL17613	+	exonic
GaLu96scf_5_581823_582260	2	0	0	1	0	0	0	GL25239	-	exonic
GaLu96scf_5_634361_635990	3	0	0	1	0	0	0	GL25443	-	exonic

GaLu96scf_5_655738_656284	4	0	0	0	0	0	1	GL25244	+	exonic
GaLu96scf_5_656284_656476	1	0	0	0	0	0	1	GL25244	+	intronic
GaLu96scf_5_686474_686732	1	0	1	0	0	0	0	GL25319	+	exonic
GaLu96scf_5_781356_782024	1	0	0	0	0	1	0	GL25250	-	exonic
GaLu96scf_5_882246_882426	1	0	0	1	0	0	0	GL17637	-	intronic
GaLu96scf_5_882247_882426	1	0	0	0	1	0	0	GL17637	-	intronic
GaLu96scf_5_892014_892486	3	0	0	0	1	0	0	GL25468	-	exonic
GaLu96scf_5_973327_974690	7	0	1	0	0	0	0	GL25265	+	exonic
GaLu96scf_5_986554_986842	2	0	0	0	0	0	1	GL25266	+	exonic
GaLu96scf_50_64028_64293	1	0	0	0	0	0	1	GL28774	+	intronic
GaLu96scf_50_69505_69982	3	0	0	0	1	0	0	GL20020	-	exonic
GaLu96scf_50_82315_83037	3	0	0	0	0	0	1	GL25480	-	exonic
GaLu96scf_51_11723_11888	1	0	0	0	0	1	0	GL17661	-	exonic
GaLu96scf_51_54110_54692	4	0	0	0	1	0	0	GL17655	+	exonic
GaLu96scf_6_1018372_1018598	1	0	0	1	0	0	0	GL31379	-	exonic
GaLu96scf_6_1077950_1078198	2	0	0	1	0	0	0	GL31499	-	exonic
GaLu96scf_6_118751_118934	1	0	0	1	0	0	0	GL31183	-	intronic
GaLu96scf_6_1268638_1268833	2	0	1	0	0	0	0	GL31192	+	exonic
GaLu96scf_6_1573349_1573850	2	0	0	0	0	1	0	GL31277	-	exonic
GaLu96scf_6_1645833_1646174	1	0	0	0	0	1	0	GL31551	+	intronic
GaLu96scf_6_271266_271532	1	0	0	1	0	0	0	GL31556	+	exonic
GaLu96scf_6_306498_307187	3	0	0	1	0	0	0	GL31411	+	exonic
GaLu96scf_6_321394_321652	1	0	0	0	0	1	0	GL31416	-	exonic
GaLu96scf_6_539666_540005	2	0	1	0	0	0	0	GL31592	-	exonic
GaLu96scf_6_578947_579630	3	0	0	0	0	1	0	GL31671	+	exonic

GaLu96scf_6_598901_599233	1	0	0	0	0	0	1	GL31599	-	exonic
GaLu96scf_6_621826_622047	1	0	0	0	0	1	0	GL31596	+	exonic
GaLu96scf_6_671717_672058	2	0	0	0	1	0	0	GL31439	+	exonic
GaLu96scf_6_671717_672723	3	0	0	0	1	0	0	GL31439	+	exonic
GaLu96scf_6_687443_687846	2	0	0	0	1	0	0	GL31602	-	exonic
GaLu96scf_6_714661_715162	3	0	0	0	1	0	0	GL31333	-	exonic
GaLu96scf_6_784412_784620	1	0	0	0	1	0	0	GL31442	+	exonic
GaLu96scf_6_796087_796690	3	0	0	0	1	0	0	GL31330	+	exonic
GaLu96scf_7_1165988_1168011	9	0	0	1	0	0	0	GL25544	+	exonic
GaLu96scf_7_1303523_1303923	3	0	1	0	0	0	0	GL20137	+	exonic
GaLu96scf_7_1367812_1368797	2	0	0	0	0	1	0	GL25639	-	exonic
GaLu96scf_7_1442180_1442334	1	0	0	0	1	0	0	GL28890	+	intronic
GaLu96scf_7_280080_280435	1	0	0	0	0	0	1	GL25727	+	intronic
GaLu96scf_7_395510_396371	5	0	0	0	1	0	0	GL25583	-	exonic
GaLu96scf_7_429260_429640	2	0	0	0	0	1	0	GL25737	+	exonic
GaLu96scf_7_724417_724685	2	0	0	1	0	0	0	GL25666	+	exonic
GaLu96scf_7_783494_783780	1	0	0	1	0	0	0	GL28937	+	intronic
GaLu96scf_7_848001_848404	2	0	0	0	1	0	0	GL25620	+	exonic
GaLu96scf_7_855404_856900	6	0	1	0	0	0	0	GL20202	-	exonic
GaLu96scf_7_938598_939368	2	0	0	0	0	1	0	GL20209	-	exonic
GaLu96scf_8_1061304_1061464	1	0	1	0	0	0	0	GL25824	-	exonic
GaLu96scf_8_1113615_1114091	4	0	0	0	0	1	0	GL25833	-	exonic
GaLu96scf_8_1147849_1148058	1	0	0	0	1	0	0	GL17882	-	intronic
GaLu96scf_8_1155274_1155430	1	1	0	0	0	0	0	GL25954	+	intronic
GaLu96scf_8_115942_116261	1	0	0	1	0	0	0	GL26035	-	intronic

GaLu96scf_8_1164203_1165005	4	0	1	0	0	0	0	GL20241	-	exonic
GaLu96scf_8_1269426_1269702	2	0	0	0	1	0	0	GL26059	-	exonic
GaLu96scf_8_131438_132010	4	0	0	0	1	0	0	GL25810	+	exonic
GaLu96scf_8_1373042_1373349	2	0	0	0	0	0	1	GL26064	+	exonic
GaLu96scf_8_1396588_1396869	2	0	0	0	0	1	0	GL25857	-	exonic
GaLu96scf_8_1436392_1437740	8	0	1	0	0	0	0	GL25858	+	exonic
GaLu96scf_8_1445249_1445578	2	0	0	0	0	0	1	GL25859	+	exonic
GaLu96scf_8_1487131_1487300	1	0	0	0	1	0	0	GL17911	-	intronic
GaLu96scf_8_1530703_1532779	3	0	0	1	0	0	0	GL26080	+	exonic
GaLu96scf_8_1543565_1544537	4	0	1	0	0	0	0	GL25975	-	exonic
GaLu96scf_8_1546120_1546461	1	0	1	0	0	0	0	GL26081	+	exonic
GaLu96scf_8_1563704_1564128	1	0	0	0	1	0	0	GL26086	-	exonic
GaLu96scf_8_224458_224652	1	0	0	0	0	1	0	GL17923	+	exonic
GaLu96scf_8_459981_460392	2	0	0	0	0	0	1	GL26103	+	exonic
GaLu96scf_8_460468_460763	2	0	0	0	0	1	0	GL26103	+	exonic
GaLu96scf_8_500709_501111	1	0	1	0	0	0	0	GL25903	-	exonic
GaLu96scf_8_70388_70865	1	0	0	0	0	0	1	GL25937	+	intronic
GaLu96scf_8_780301_780833	2	0	1	0	0	0	0	GL26008	+	exonic
GaLu96scf_8_796477_796681	1	0	0	1	0	0	0	GL26012	-	intronic
GaLu96scf_8_815914_816094	1	0	0	1	0	0	0	GL17965	+	intronic
GaLu96scf_8_824225_824594	2	0	0	0	1	0	0	GL26017	-	exonic
GaLu96scf_8_861058_861503	2	1	0	0	0	0	0	GL25928	-	exonic
GaLu96scf_8_885685_886367	4	1	0	0	0	0	0	GL26140	-	exonic
GaLu96scf_8_991639_992137	2	0	0	0	1	0	0	GL25932	+	exonic
GaLu96scf_9_1048703_1049122	3	0	1	0	0	0	0	GL26471	+	exonic

GaLu96scf_9_1049550_1050143	1	0	1	0	0	0	0	GL26471	+	intronic
GaLu96scf_9_1062767_1063090	1	0	0	0	0	0	1	GL18105	+	exonic
GaLu96scf_9_1080098_1080337	2	0	0	1	0	0	0	GL26691	-	exonic
GaLu96scf_9_1142258_1142870	2	0	0	0	0	1	0	GL26597	+	exonic
GaLu96scf_9_1269023_1269328	2	0	1	0	0	0	0	GL26606	+	exonic
GaLu96scf_9_1293365_1293918	2	0	0	1	0	0	0	GL26498	-	exonic
GaLu96scf_9_1338565_1339219	2	0	0	0	0	1	0	GL26724	+	exonic
GaLu96scf_9_1411797_1412144	2	0	0	0	1	0	0	GL26733	+	exonic
GaLu96scf_9_1431677_1432015	2	0	1	0	0	0	0	GL18130	+	exonic
GaLu96scf_9_1454807_1455167	2	0	0	0	1	0	0	GL18132	-	exonic
GaLu96scf_9_1500539_1500934	2	0	0	0	1	0	0	GL26736	+	exonic
GaLu96scf_9_1521817_1522411	3	0	0	0	0	0	1	GL26620	-	exonic
GaLu96scf_9_158269_158700	1	0	0	0	0	0	1	GL26678	-	intronic
GaLu96scf_9_249538_249788	1	0	0	1	0	0	0	GL26621	+	exonic
GaLu96scf_9_316434_316999	5	0	0	0	0	0	1	GL26745	+	exonic
GaLu96scf_9_478315_478519	1	0	0	0	0	1	0	GL26633	+	intronic
GaLu96scf_9_513792_514820	3	0	0	0	0	0	1	GL26638	-	exonic
GaLu96scf_9_60299_60551	1	1	0	0	0	0	0	GL20391	+	exonic
GaLu96scf_9_614617_614812	1	0	0	0	0	0	1	GL18163	+	exonic
GaLu96scf_9_636235_636599	2	0	1	0	0	0	0	GL26775	-	exonic
GaLu96scf_9_729107_730748	7	0	0	0	1	0	0	GL26645	+	exonic
GaLu96scf_9_737529_738069	1	0	0	0	0	1	0	GL26648	-	exonic
GaLu96scf_9_751112_751492	2	0	0	0	0	1	0	GL26781	+	exonic
GaLu96scf_9_765949_766240	2	0	0	1	0	0	0	GL26788	-	exonic
GaLu96scf_9_766111_766410	2	0	0	1	0	0	0	GL26788	-	exonic

GaLu96scf_9_864177_864365	2	0	0	0	0	0	1	GL26572	-	exonic
GaLu96scf_9_864649_867150	13	0	0	1	0	0	0	GL26572	-	exonic

Table S3 List of identified of circRNAs in *G. lucidum* based on poly(A) RNA-seq data.

Circ_ID	Exon Count	Back spliced junction reads in poly(A)-RNA-seq						Name of Parent Gene	Strand	CircType
		Mycelia		Primordia		Fruiting Bodies				
		R1	R2	R1	R2	R1	R2			
GaLu96scf_1_1021643_1022328	3	0	1	0	0	0	1	GL30888	+	exonic
GaLu96scf_1_1825532_1825739	1	0	0	0	0	1	0	GL29917	+	exonic
GaLu96scf_1_1832017_1833145	7	0	0	0	1	0	0	GL30917	+	exonic
GaLu96scf_1_187034_187549	4	0	0	1	0	0	0	GL29837	+	exonic
GaLu96scf_1_2004521_2004822	2	0	0	0	2	0	0	GL30538	-	exonic
GaLu96scf_1_2047995_2049134	3	1	0	0	0	0	0	GL30239	+	exonic
GaLu96scf_1_2190249_2190615	1	0	0	0	0	0	2	GL30545	+	exonic
GaLu96scf_1_224262_224453	1	0	0	1	0	0	0	GL29930	+	exonic
GaLu96scf_1_2415795_2416010	1	0	0	0	0	0	1	GL29983	-	exonic
GaLu96scf_1_2659140_2659735	2	0	0	0	1	0	0	GL29411	-	exonic
GaLu96scf_1_2659518_2659735	1	0	0	1	0	0	0	GL29411	-	exonic
GaLu96scf_1_2965726_2965968	1	0	0	2	0	0	0	GL30637	-	exonic
GaLu96scf_1_3332784_3333104	2	0	0	3	8	5	4	GL30048	+	exonic
GaLu96scf_1_344052_344528	3	0	0	0	0	1	0	GL30986	-	exonic
GaLu96scf_1_3451208_3451619	1	1	0	0	0	0	0	GL30704	-	exonic
GaLu96scf_1_3583432_3583976	2	0	0	0	0	1	0	GL30718	-	exonic

GaLu96scf_1_3585501_3585800	1	0	0	0	0	1	0	GL30712	+	exonic
GaLu96scf_1_4020862_4021597	5	0	0	1	0	0	0	GL30770	+	exonic
GaLu96scf_1_4248863_4250422	4	0	0	1	0	0	0	GL30791	+	exonic
GaLu96scf_1_4323938_4324354	3	0	0	1	0	0	0	GL30121	-	exonic
GaLu96scf_1_440858_441430	3	0	0	0	1	0	0	GL30097	-	exonic
GaLu96scf_1_441534_441919	2	0	0	0	3	4	2	GL30097	-	exonic
GaLu96scf_1_613208_613932	2	0	0	0	0	1	0	GL29617	+	exonic
GaLu96scf_1_687514_688523	2	0	0	2	1	0	0	GL29620	-	exonic
GaLu96scf_1_822816_823309	2	0	0	3	0	0	0	GL29633	-	exonic
GaLu96scf_1_822816_823531	3	0	0	1	0	0	0	GL29633	-	exonic
GaLu96scf_1_89101_89611	2	0	0	0	1	0	0	GL30875	-	exonic
GaLu96scf_1_89438_89611	1	0	0	1	0	0	0	GL30875	-	exonic
GaLu96scf_10_1363675_1363940	1	0	0	0	0	0	2	GL20592	-	exonic
GaLu96scf_10_339670_340364	4	0	0	0	2	0	0	GL20542	+	exonic
GaLu96scf_10_595489_595752	1	0	0	0	0	2	0	GL20502	-	intronic
GaLu96scf_10_597910_598302	1	0	0	0	0	1	0	GL20626	+	intronic
GaLu96scf_10_633036_633434	2	0	0	0	0	0	2	GL20627	+	exonic
GaLu96scf_10_926801_927222	2	0	0	0	0	1	0	GL20561	-	exonic
GaLu96scf_11_100041_100404	2	0	0	0	0	0	1	GL20660	+	exonic
GaLu96scf_11_1114592_1115418	2	0	0	0	1	0	0	GL20669	+	exonic
GaLu96scf_11_1153012_1153342	2	0	0	3	3	5	0	GL20745	-	exonic
GaLu96scf_11_1234719_1235461	3	0	0	0	3	0	0	GL20816	-	exonic
GaLu96scf_11_1286548_1286779	1	0	0	0	0	1	0	GL15159	-	exonic
GaLu96scf_11_156087_157001	2	0	0	0	1	0	0	GL15133	+	exonic
GaLu96scf_11_185670_186176	2	0	0	2	0	0	0	GL20738	+	exonic

GaLu96scf_11_338104_338407	2	0	0	0	0	1	0	GL20839	+	exonic
GaLu96scf_11_550080_550507	1	0	0	0	1	0	0	GL20860	+	intronic
GaLu96scf_11_612651_612944	2	0	0	1	0	0	0	GL20868	-	exonic
GaLu96scf_11_699350_699504	1	0	0	0	0	1	0	GL18364	+	exonic
GaLu96scf_11_725031_725261	2	0	0	0	0	2	0	GL20718	-	exonic
GaLu96scf_11_965780_967233	3	0	0	0	0	0	1	GL20883	+	exonic
GaLu96scf_12_1110164_1110488	1	0	0	1	0	0	0	GL18413	-	exonic
GaLu96scf_12_1124455_1125058	2	0	0	3	0	0	0	GL21038	+	exonic
GaLu96scf_12_387721_388104	2	0	0	1	1	0	0	GL21056	-	exonic
GaLu96scf_12_449898_450282	1	0	0	1	0	0	0	GL18437	-	exonic
GaLu96scf_12_450878_452244	3	0	0	0	1	0	0	GL18437	-	exonic
GaLu96scf_12_451474_452244	2	2	0	0	1	0	0	GL18437	-	exonic
GaLu96scf_12_716899_717289	1	0	0	0	0	3	0	GL21074	-	exonic
GaLu96scf_12_922030_922271	1	0	0	0	0	0	3	GL20960	+	exonic
GaLu96scf_13_1104139_1104490	1	0	0	1	0	0	0	GL21214	+	exonic
GaLu96scf_13_214486_215981	7	0	0	0	3	0	0	GL21114	-	exonic
GaLu96scf_13_436487_436788	2	0	0	0	0	0	1	GL21226	+	exonic
GaLu96scf_13_438876_439167	1	0	0	0	2	1	0	GL21226	+	exonic
GaLu96scf_13_946765_947266	1	0	1	0	0	0	1	GL21157	-	exonic
GaLu96scf_14_1023194_1023668	2	0	0	0	1	0	0	GL21392	+	exonic
GaLu96scf_14_294548_295479	3	0	0	0	0	0	1	GL18545	+	exonic
GaLu96scf_14_399551_400019	2	0	0	0	1	0	0	GL15508	-	exonic
GaLu96scf_14_406887_407305	3	0	0	0	1	0	0	GL21413	+	exonic
GaLu96scf_14_585671_586031	2	0	0	1	0	0	0	GL21354	-	exonic
GaLu96scf_14_737539_737858	2	0	0	0	0	0	1	GL21361	+	exonic

GaLu96scf_14_8634_9042	2	0	0	0	1	0	0	GL21375	+	exonic
GaLu96scf_14_977560_978372	1	0	0	6	0	11	0	GL27220	+	exonic
GaLu96scf_14_997917_998788	4	0	0	0	1	0	0	GL21283	-	exonic
GaLu96scf_15_164699_165283	4	0	0	0	0	1	0	GL27226	-	exonic
GaLu96scf_15_263810_264000	2	0	0	1	1	0	0	GL21471	+	exonic
GaLu96scf_15_50637_50979	1	0	0	1	0	0	0	GL21556	-	exonic
GaLu96scf_15_577800_578307	3	0	0	0	0	0	2	GL21539	+	exonic
GaLu96scf_15_577800_578706	4	0	0	1	0	0	0	GL21539	+	exonic
GaLu96scf_15_782118_782375	1	0	0	1	0	0	0	GL18640	-	exonic
GaLu96scf_15_791486_791712	1	0	0	0	0	1	3	GL21542	+	exonic
GaLu96scf_15_791486_791962	2	0	0	0	0	0	1	GL21542	+	exonic
GaLu96scf_15_821086_822351	2	0	0	2	0	0	0	GL21515	-	exonic
GaLu96scf_15_822370_823019	1	0	0	0	0	1	0	GL21515	-	intronic
GaLu96scf_15_894656_895046	2	0	0	0	1	0	0	GL21512	+	exonic
GaLu96scf_16_371965_372200	2	0	0	0	0	0	1	GL21742	+	exonic
GaLu96scf_16_375461_376544	3	0	0	0	0	1	0	GL15668	+	exonic
GaLu96scf_16_544240_544486	1	0	0	0	0	0	1	GL18694	+	exonic
GaLu96scf_16_544486_544886	1	0	0	0	0	1	0	GL18694	+	intronic
GaLu96scf_16_597130_597465	1	0	0	0	1	0	0	GL27313	+	exonic
GaLu96scf_16_604576_605067	2	0	0	0	0	3	0	GL21653	-	exonic
GaLu96scf_16_607479_608085	1	0	0	5	3	5	5	GL21693	-	exonic
GaLu96scf_16_856246_856605	2	1	0	0	0	0	0	GL15699	-	exonic
GaLu96scf_17_302808_303070	2	0	0	0	1	0	0	GL21921	-	exonic
GaLu96scf_17_309529_309938	2	0	0	0	0	0	1	GL15753	-	exonic
GaLu96scf_17_627501_627787	2	0	0	0	0	1	0	GL21838	-	exonic

GaLu96scf_17_937830_938049	1	0	0	1	0	0	0	GL18792	+	intronic
GaLu96scf_18_382504_382751	2	0	0	0	0	2	0	GL21997	-	exonic
GaLu96scf_18_520070_520441	2	0	0	0	0	1	0	GL22109	+	exonic
GaLu96scf_18_567176_567953	3	0	0	2	0	0	0	GL27401	-	exonic
GaLu96scf_18_621638_621839	2	0	0	0	0	1	0	GL22024	-	exonic
GaLu96scf_19_250245_250455	1	0	0	0	0	3	0	GL22149	-	exonic
GaLu96scf_19_280067_280486	2	0	0	0	1	0	0	GL22188	+	exonic
GaLu96scf_19_800578_802324	5	0	0	1	0	0	0	GL22226	-	exonic
GaLu96scf_2_1056816_1057245	2	0	0	2	0	0	0	GL22329	-	exonic
GaLu96scf_2_1057061_1057245	1	1	0	0	0	3	1	GL22329	-	exonic
GaLu96scf_2_1057061_1057450	2	0	0	0	0	1	0	GL22329	-	exonic
GaLu96scf_2_1194086_1194486	2	0	0	0	0	0	1	GL15998	+	exonic
GaLu96scf_2_1224538_1224876	1	0	0	2	0	5	0	GL22551	+	exonic
GaLu96scf_2_1430127_1430302	1	0	0	1	0	0	0	GL22566	-	intronic
GaLu96scf_2_1541899_1545769	6	0	0	0	0	1	0	GL22569	+	exonic
GaLu96scf_2_1682148_1682409	1	0	0	0	0	1	0	GL22369	-	exonic
GaLu96scf_2_1700587_1700778	1	0	0	0	1	0	0	GL22372	+	exonic
GaLu96scf_2_176740_177035	2	0	0	0	0	0	3	GL15974	+	exonic
GaLu96scf_2_1770335_1770882	3	0	0	0	1	1	1	GL22592	-	exonic
GaLu96scf_2_476492_476787	1	0	0	0	3	0	0	GL22642	-	exonic
GaLu96scf_2_663567_664349	2	0	0	0	0	5	0	GL22656	-	exonic
GaLu96scf_2_699135_699514	1	0	0	0	0	1	0	GL22434	-	exonic
GaLu96scf_2_724113_724771	2	0	0	1	0	0	0	GL22518	-	exonic
GaLu96scf_2_778673_779108	2	0	0	0	0	1	0	GL22662	+	exonic
GaLu96scf_2_778974_780464	2	0	0	0	1	4	2	GL22662	+	exonic

GaLu96scf_2_892552_893361	3	0	0	1	0	0	0	GL22528	-	exonic
GaLu96scf_2_923644_924029	2	0	0	0	0	1	0	GL22451	+	exonic
GaLu96scf_2_977213_977848	3	0	0	0	0	1	0	GL16173	+	exonic
GaLu96scf_2_979223_980204	2	0	0	0	1	0	0	GL27636	-	exonic
GaLu96scf_20_109752_109966	1	0	0	0	0	1	0	GL22704	-	intronic
GaLu96scf_20_142419_143191	2	0	0	0	0	4	0	GL22680	+	exonic
GaLu96scf_20_286802_287132	1	0	0	0	1	0	0	GL27658	+	exonic
GaLu96scf_21_181854_182048	1	0	0	1	0	2	0	GL22829	+	exonic
GaLu96scf_21_34416_34722	1	0	0	0	0	1	0	GL22778	+	exonic
GaLu96scf_21_370958_372274	1	0	0	1	0	0	0	GL22878	+	exonic
GaLu96scf_21_411133_411951	3	0	0	1	0	0	0	GL27743	+	exonic
GaLu96scf_22_104707_105018	2	0	0	0	1	0	0	GL31761	-	exonic
GaLu96scf_22_118127_118753	5	0	0	0	0	19	0	GL22968	+	exonic
GaLu96scf_22_122357_124287	5	0	0	0	1	0	0	GL22915	-	exonic
GaLu96scf_22_171118_171475	1	0	0	0	0	0	1	GL22965	-	exonic
GaLu96scf_22_184039_185441	5	0	0	0	0	1	0	GL22918	-	exonic
GaLu96scf_22_187637_188188	2	0	0	1	1	0	0	GL22966	-	exonic
GaLu96scf_22_682319_682655	1	0	0	0	1	0	0	GL19186	+	exonic
GaLu96scf_22_793771_793960	2	0	0	0	0	1	0	GL23028	-	exonic
GaLu96scf_23_114407_115124	2	0	0	0	1	0	0	GL23036	-	exonic
GaLu96scf_23_615904_616279	2	0	0	2	1	0	0	GL16483	+	exonic
GaLu96scf_24_131181_131394	1	0	0	3	0	0	0	GL23137	-	intronic
GaLu96scf_24_643346_643626	1	0	0	0	0	0	1	GL23172	-	exonic
GaLu96scf_25_479247_479504	1	0	0	0	1	0	1	GL23247	-	exonic
GaLu96scf_26_117579_117907	1	1	0	0	0	0	0	GL16639	+	exonic

GaLu96scf_26_136975_137250	1	0	0	0	0	5	0	GL23327	+	exonic
GaLu96scf_26_547883_548661	3	0	0	1	0	0	0	GL23326	-	exonic
GaLu96scf_27_128048_128539	3	0	0	0	0	17	0	GL23383	+	exonic
GaLu96scf_27_412103_415992	13	0	0	0	1	0	0	GL23450	+	exonic
GaLu96scf_28_279935_281242	7	1	0	0	0	0	0	GL23477	+	exonic
GaLu96scf_28_526348_526653	1	0	1	0	0	0	1	GL23538	+	exonic
GaLu96scf_29_35184_36198	3	0	0	1	0	0	0	GL23546	-	exonic
GaLu96scf_29_427459_427700	1	0	0	3	2	2	0	GL23621	-	exonic
GaLu96scf_29_50957_51214	1	0	0	1	0	1	0	GL23547	-	exonic
GaLu96scf_3_1033607_1034216	3	0	0	0	0	2	0	GL23866	+	exonic
GaLu96scf_3_1135050_1135563	1	0	0	0	0	0	1	GL23647	+	exonic
GaLu96scf_3_1140214_1140529	1	0	0	3	0	0	0	GL23798	+	exonic
GaLu96scf_3_1279545_1279732	1	0	0	0	0	2	0	GL28147	-	exonic
GaLu96scf_3_1296420_1296820	1	0	0	1	0	0	0	GL23665	-	exonic
GaLu96scf_3_1332003_1332585	4	0	0	0	3	0	0	GL16846	+	exonic
GaLu96scf_3_1674984_1675525	2	1	0	0	0	0	0	GL23899	+	exonic
GaLu96scf_3_1784058_1784342	1	0	0	0	1	0	0	GL16873	-	intronic
GaLu96scf_3_1818467_1818647	1	0	0	0	0	1	1	GL23913	+	exonic
GaLu96scf_3_1853089_1853479	1	0	0	0	0	1	0	GL23696	+	intronic
GaLu96scf_3_1857435_1857755	2	0	0	0	0	2	0	GL23703	-	exonic
GaLu96scf_3_1899354_1899568	1	0	0	0	0	0	2	GL23921	+	exonic
GaLu96scf_3_495478_495826	2	0	0	0	0	1	0	GL23731	-	exonic
GaLu96scf_3_575832_576089	2	0	0	0	0	1	0	GL23742	-	exonic
GaLu96scf_3_711836_712165	2	1	0	0	2	2	0	GL23962	-	exonic
GaLu96scf_3_804337_805521	3	0	0	0	0	1	0	GL23968	-	exonic

GaLu96scf_3_961241_961584	3	0	0	0	0	1	0	GL23775	-	exonic
GaLu96scf_30_254564_256000	4	0	0	0	0	1	0	GL24029	-	exonic
GaLu96scf_31_234356_235338	2	0	0	2	0	0	0	GL28294	+	exonic
GaLu96scf_31_235519_235921	1	2	0	0	0	0	0	GL28294	+	exonic
GaLu96scf_31_375583_375831	1	0	0	0	1	0	0	GL19615	-	exonic
GaLu96scf_31_39627_39859	1	0	1	0	2	0	1	GL24100	+	exonic
GaLu96scf_31_426823_427539	3	0	0	0	0	1	0	GL24071	+	exonic
GaLu96scf_31_450431_450722	1	0	0	0	0	1	0	GL24131	+	exonic
GaLu96scf_31_70509_70945	3	0	0	0	0	1	0	GL24040	-	exonic
GaLu96scf_33_107955_108150	1	0	0	2	2	7	4	GL24214	-	exonic
GaLu96scf_34_95773_96416	1	2	0	0	0	0	0	GL24286	+	exonic
GaLu96scf_35_279057_279437	2	0	0	0	0	1	0	GL28408	-	exonic
GaLu96scf_35_311434_311687	1	0	0	0	1	0	0	GL24311	+	exonic
GaLu96scf_35_328571_328844	1	0	0	0	0	1	1	GL24337	+	exonic
GaLu96scf_35_328844_329092	1	0	0	1	0	0	0	GL24337	+	intronic
GaLu96scf_38_71801_72077	1	0	0	0	0	1	0	GL24409	-	intronic
GaLu96scf_4_111472_111932	3	0	0	0	1	0	0	GL24629	+	exonic
GaLu96scf_4_1128146_1128467	2	0	0	0	0	3	2	GL24752	-	exonic
GaLu96scf_4_1128319_1128806	3	0	0	1	0	0	0	GL24752	-	exonic
GaLu96scf_4_1339336_1339717	2	0	0	0	0	1	0	GL24645	-	exonic
GaLu96scf_4_140106_140434	1	0	0	0	0	2	0	GL17243	-	exonic
GaLu96scf_4_1459669_1460308	3	0	0	0	0	0	1	GL17270	+	exonic
GaLu96scf_4_1575129_1575693	1	0	0	0	0	1	0	GL24652	-	exonic
GaLu96scf_4_168492_169034	3	0	0	0	0	0	2	GL28477	+	exonic
GaLu96scf_4_168492_169400	4	0	0	0	2	3	0	GL28477	+	exonic

GaLu96scf_4_169756_170276	2	0	0	0	0	2	0	GL28477	+	exonic
GaLu96scf_4_1884292_1885135	4	0	0	0	0	1	0	GL24531	+	exonic
GaLu96scf_4_532307_532692	3	0	0	0	0	2	0	GL28557	-	exonic
GaLu96scf_4_721918_722172	2	0	0	0	0	1	0	GL24589	-	exonic
GaLu96scf_4_753012_754104	4	0	0	0	1	0	0	GL24693	-	exonic
GaLu96scf_4_942105_942888	3	0	0	0	0	0	1	GL17366	+	exonic
GaLu96scf_42_145252_145529	1	0	0	0	0	2	0	GL17424	-	exonic
GaLu96scf_42_187462_188053	1	0	0	0	1	0	0	GL28618	+	exonic
GaLu96scf_43_107810_108039	1	0	0	0	0	3	0	GL28634	+	exonic
GaLu96scf_43_107810_108315	2	0	0	0	0	2	0	GL28634	+	exonic
GaLu96scf_44_81238_81513	1	0	0	0	2	0	0	GL17453	+	intronic
GaLu96scf_44_95901_96640	2	0	0	0	1	0	0	GL25033	-	exonic
GaLu96scf_46_122370_125256	6	0	0	0	1	0	0	GL17498	-	exonic
GaLu96scf_47_89640_90331	6	0	0	0	0	1	0	GL25106	+	exonic
GaLu96scf_5_1045707_1046124	3	0	0	1	0	0	0	GL25280	-	exonic
GaLu96scf_5_10565_10871	2	0	0	0	1	0	0	GL25146	+	exonic
GaLu96scf_5_1100364_1100544	1	0	0	0	0	0	4	GL17543	+	exonic
GaLu96scf_5_1256765_1256931	1	0	0	0	0	0	1	GL28715	-	intronic
GaLu96scf_5_246689_247092	2	0	0	0	0	1	0	GL25209	+	exonic
GaLu96scf_5_288195_288558	2	0	0	3	0	0	2	GL25300	+	exonic
GaLu96scf_5_321020_321938	6	0	0	0	0	1	0	GL25215	+	exonic
GaLu96scf_5_36393_36732	2	0	0	2	0	0	0	GL25151	-	exonic
GaLu96scf_5_417693_418112	3	0	0	2	0	0	0	GL25223	+	exonic
GaLu96scf_5_419746_420267	3	0	0	0	0	1	0	GL25223	+	exonic
GaLu96scf_5_420067_420267	1	0	0	0	0	1	0	GL25223	+	exonic

GaLu96scf_5_428692_429138	3	0	0	3	0	0	0	GL25425	-	exonic
GaLu96scf_5_516816_517239	1	0	0	0	0	0	1	GL17615	-	exonic
GaLu96scf_5_789033_789509	2	0	0	0	0	1	0	GL25322	+	exonic
GaLu96scf_5_825181_825654	3	0	0	0	0	2	0	GL25254	+	exonic
GaLu96scf_51_69268_69618	1	0	0	0	1	0	0	GL28783	+	exonic
GaLu96scf_6_118733_118934	1	0	0	0	0	1	0	GL31183	-	intronic
GaLu96scf_6_1233934_1234381	3	0	0	0	0	1	0	GL31514	+	exonic
GaLu96scf_6_1278057_1278485	2	0	0	2	7	4	2	GL31640	-	exonic
GaLu96scf_6_284761_285099	1	0	0	0	0	0	2	GL31157	-	exonic
GaLu96scf_6_370594_370941	1	0	0	0	0	1	0	GL31572	-	exonic
GaLu96scf_6_579734_580190	2	0	0	0	0	1	0	GL31671	+	exonic
GaLu96scf_6_597741_599233	3	0	0	1	0	0	0	GL31599	-	exonic
GaLu96scf_6_872307_872566	2	0	0	0	1	0	0	GL31341	+	exonic
GaLu96scf_7_1167778_1168011	1	0	0	0	1	1	0	GL25544	+	exonic
GaLu96scf_7_1167778_1168333	2	0	0	2	1	0	4	GL25544	+	exonic
GaLu96scf_7_1367812_1368966	3	0	0	0	1	0	0	GL25639	-	exonic
GaLu96scf_7_1621353_1622307	4	0	0	0	1	0	0	GL25721	-	exonic
GaLu96scf_7_210568_210747	1	0	0	0	0	1	0	GL28919	+	exonic
GaLu96scf_7_454962_456236	4	0	0	0	0	2	0	GL25740	+	exonic
GaLu96scf_7_647053_647313	1	0	0	0	0	1	0	GL25610	-	exonic
GaLu96scf_7_847373_847794	2	5	1	0	0	0	1	GL25620	+	exonic
GaLu96scf_7_926139_926753	4	0	0	0	1	0	0	GL25783	-	exonic
GaLu96scf_7_926461_926753	2	0	0	0	0	3	0	GL25783	-	exonic
GaLu96scf_8_1022209_1023370	2	0	0	0	0	0	1	GL25822	-	exonic
GaLu96scf_8_498907_499140	1	0	0	2	0	0	0	GL25895	+	exonic

GaLu96scf_8_586612_586805	1	0	0	0	0	0	1	GL26114	-	exonic
GaLu96scf_8_863122_863358	1	0	1	3	0	1	1	GL25929	-	exonic
GaLu96scf_8_89554_89854	1	0	0	0	3	2	0	GL26030	-	exonic
GaLu96scf_9_1049221_1049550	2	0	0	0	0	1	0	GL26471	+	exonic
GaLu96scf_9_1059347_1059821	1	0	0	1	0	0	0	GL18105	+	exonic
GaLu96scf_9_517326_517556	1	0	0	1	0	0	0	GL26763	-	exonic
GaLu96scf_9_729107_729718	2	0	0	0	1	1	0	GL26645	+	exonic
GaLu96scf_9_981635_981978	2	0	0	0	0	1	0	GL26582	-	exonic
GaLu96scf_9_981635_982113	3	0	0	0	1	0	2	GL26582	-	exonic

Table S4a Length of introns of exonic circRNAs identified acrosss the three developmental stages of *G.lucidum*

circRNA ID	Left introns		Right Intron		Control Intron	
	Length (bps)	Stages found	Length (bps)	Stages found	Length (bps)	Stages found
GaLu96scf_12_922030_922271	127	M/P/FB	180	M/P/FB	54	FB
GaLu96scf_13_438876_439167	240	M/P/FB	92	M/P/FB	52	FB
GaLu96scf_14_977560_978372	310	M/P/FB	382	M/P/FB	50	FB
GaLu96scf_16_607479_608085	407	M/P/FB	446	M/P/FB	25	FB
GaLu96scf_19_250245_250455	154	M/P/FB	161	M/P/FB	52	FB
GaLu96scf_24_643346_643626	313	M/P/FB	82	M/P/FB	50	FB
GaLu96scf_25_479247_479504	94	P/FB	107	P/FB	52	FB
GaLu96scf_26_136975_137250	99	M/P/FB	316	M/P/FB	55	FB
GaLu96scf_28_526348_526653	156	M/P	67	M/P	98	FB
GaLu96scf_29_427459_427700	108	M/P/FB	225	M/P/FB	74	FB
GaLu96scf_31_39627_39859	77	M/P/FB	143	M/P/FB	52	FB
GaLu96scf_32_269704_269946	75	M/FB	205	M/FB	132	FB
GaLu96scf_35_328571_328844	104	M/P/FB	267	M/P/FB	67	FB
GaLu96scf_4_1279082_1279396	277	M/P/FB	90	M/P/FB	28	FB
GaLu96scf_42_145252_145529	403	M/P/FB	70	M/P/FB	59	FB
GaLu96scf_5_516816_517239	81	M/P/FB	124	M/P/FB	13	FB
GaLu96scf_50_63748_64028	82	M/P/FB	279	M/P/FB	85	FB
GaLu96scf_8_863122_863358	140	M/P/FB	157	M/P/FB	50	FB
GaLu96scf_1_1832393_1832925	106	M/P/FB	98	FB	85	P
GaLu96scf_1_1832393_1832925	106	M/P/FB	98	FB	60	P
GaLu96scf_1_1832393_1832925	106	M/P/FB	98	FB	67	P
GaLu96scf_1_1832393_1833145	106	M/P/FB	100	FB	85	P
GaLu96scf_1_1832393_1833145	106	M/P/FB	100	FB	60	P
GaLu96scf_1_1832393_1833145	106	M/P/FB	100	FB	67	P
GaLu96scf_1_1832393_1833145	106	M/P/FB	100	FB	98	P
GaLu96scf_1_2047995_2048512	205	M/P	73	M	28	P
GaLu96scf_1_2047995_2049134	205	M/P	63	M	28	P
GaLu96scf_1_2047995_2049134	205	M/P	63	M	73	P
GaLu96scf_1_3383244_3383958	58	M/P	60	M	56	P
GaLu96scf_1_407026_407504	275	M/P/FB	61	M/FB	160	P
GaLu96scf_1_407026_407697	275	M/P/FB	67	M/FB	160	P
GaLu96scf_1_407026_407697	275	M/P/FB	67	M/FB	61	P
GaLu96scf_1_407026_407897	275	M/P/FB	653	M/FB	160	P
GaLu96scf_1_407026_407897	275	M/P/FB	653	M/FB	61	P
GaLu96scf_1_407026_407897	275	M/P/FB	653	M/FB	67	P

GaLu96scf_1_407026_407897	275	M/P/FB	653	M/FB	64	P
GaLu96scf_1_4533060_4533570	138	M/P/FB	121	FB	81	P
GaLu96scf_1_4533060_4533570	138	M/P/FB	121	FB	60	P
GaLu96scf_1_4533060_4533858	138	M/P/FB	132	FB	81	P
GaLu96scf_1_4533060_4533858	138	M/P/FB	132	FB	60	P
GaLu96scf_1_4533060_4533858	138	M/P/FB	132	FB	121	P
GaLu96scf_1_4533308_4533858	81	P	132	M	60	P
GaLu96scf_1_4533308_4533858	81	P	132	M	121	P
GaLu96scf_10_1301597_1301961	81	M/P/FB	49	M	62	P
GaLu96scf_11_185670_186176	106	M/P/FB	120	M	242	P
GaLu96scf_11_699350_700144	113	M/P/FB	68	M/FB	488	P
GaLu96scf_11_714917_715339	66	P	91	M/FB	82	P
GaLu96scf_11_730171_730635	114	M/P	173	M	116	P
GaLu96scf_15_577955_578307	81	M/P/FB	154	M	87	P
GaLu96scf_15_577955_578706	81	M/P/FB	91	M	87	P
GaLu96scf_15_577955_578706	81	M/P/FB	91	M	154	P
GaLu96scf_15_821086_822351	131	M/P/FB	667	M	74	P
GaLu96scf_16_313804_314549	102	P	38	FB	96	P
GaLu96scf_16_313804_314549	102	P	38	FB	79	P
GaLu96scf_16_313804_314549	102	P	38	FB	69	P
GaLu96scf_16_744501_744869	47	P	210	M	54	P
GaLu96scf_17_310040_310315	101	P	96	M	70	P
GaLu96scf_2_663567_664349	412	M/P/FB	966	M	68	P
GaLu96scf_2_778673_779108	1226	M/P/FB	1195	M	186	P
GaLu96scf_2_778673_780464	1226	M/P/FB	253	M	186	P
GaLu96scf_2_778673_780464	1226	M/P/FB	253	M	195	P
GaLu96scf_25_479837_480088	63	P	140	FB	60	P
GaLu96scf_27_128806_129874	266	M/P/FB	950	M/FB	485	P
GaLu96scf_27_128806_129874	266	M/P/FB	950	M/FB	372	P
GaLu96scf_27_366457_366866	61	P	50	M	52	P
GaLu96scf_27_366457_366866	61	P	50	M	53	P
GaLu96scf_28_501725_502375	69	M/P	81	M	103	P
GaLu96scf_31_259187_259684	52	M/P/FB	54	FB	59	P
GaLu96scf_32_167826_168525	120	M/P	70	M	81	P
GaLu96scf_4_168873_169400	148	P	355	M/FB	250	P
GaLu96scf_6_354335_354658	49	P	50	FB	21	P
GaLu96scf_6_864126_864880	119	M/P	58	M	74	P
GaLu96scf_6_864126_864880	119	M/P	58	M	132	P
GaLu96scf_6_864126_864880	119	M/P	58	M	20	P
GaLu96scf_8_460253_460584	128	M/P	78	FB	75	P
GaLu96scf_8_460253_460763	128	M/P	371	FB	75	P
GaLu96scf_8_460253_460763	128	M/P	371	FB	78	P
GaLu96scf_9_1500539_1500934	67	M/P	77	M	120	P
GaLu96scf_1_1430262_1430474	151	M/P/FB	118	M/P/FB		

GaLu96scf_1_1533440_1533730	64	M/P/FB	47	M/P/FB		
GaLu96scf_1_1891179_1891452	77	M/P/FB	153	M/P/FB		
GaLu96scf_1_2922471_2922676	181	M/P/FB	192	M/P/FB		
GaLu96scf_1_3002925_3003256	119	M/P/FB	232	M/P/FB		
GaLu96scf_1_3923133_3923420	69	M/P/FB	103	M/P/FB		
GaLu96scf_1_441534_441668	103	M/P/FB	85	P		
GaLu96scf_1_441754_441919	85	M/P/FB	233	M/P/FB		
GaLu96scf_1_823157_823309	197	M/P/FB	77	M/P/FB		
GaLu96scf_1_835280_835527	16	M/P/FB	18	M/P/FB		
GaLu96scf_11_1234719_1234903	193	M/P/FB	99	M/P		
GaLu96scf_11_965353_965635	232	M/P/FB	144	M/P/FB		
GaLu96scf_12_1110164_1110488	136	M/P/FB	50	M/P/FB		
GaLu96scf_12_449898_450282	90	M/P/FB	595	M/P/FB		
GaLu96scf_12_450878_451254	595	M/P/FB	219	M/P		
GaLu96scf_12_716899_717289	278	M/P/FB	346	M/P/FB		
GaLu96scf_12_837790_838122	100	M/P/FB	45	M/P/FB		
GaLu96scf_13_1074725_1075149	270	M/P/FB	147	M/P		
GaLu96scf_13_1104139_1104490	177	M/P/FB	250	M/P/FB		
GaLu96scf_14_125920_126149	448	M/P/FB	578	M/P		
GaLu96scf_14_474829_475175	92	M/P/FB	56	M/P/FB		
GaLu96scf_15_791486_791712	134	M/P/FB	82	M/P/FB		
GaLu96scf_17_24055_24231	162	M/P/FB	133	M/P/FB		
GaLu96scf_17_538649_538861	104	M/P/FB	96	M/P/FB		
GaLu96scf_19_270134_270627	67	M/P/FB	336	M/P/FB		
GaLu96scf_19_48561_48872	70	M/P/FB	50	M/P/FB		
GaLu96scf_19_616057_616293	162	M/P/FB	59	M/P/FB		
GaLu96scf_19_649330_649603	83	M/P/FB	201	M/P/FB		
GaLu96scf_2_1034675_1034909	76	M/P/FB	95	M/P/FB		
GaLu96scf_2_1057061_1057245	117	M/P/FB	108	M/P/FB		
GaLu96scf_2_1224538_1224876	150	M/P/FB	118	M/P/FB		
GaLu96scf_2_1463452_1463671	166	M/P/FB	733	M/P/FB		
GaLu96scf_2_1541899_1542100	1813	M/P/FB	1532	M/P/FB		
GaLu96scf_2_1682148_1682409	146	M/P/FB	55	M/P/FB		
GaLu96scf_2_202079_202358	99	M/P/FB	222	M/P/FB		
GaLu96scf_2_724504_724771	322	M/P/FB	80	M/P/FB		
GaLu96scf_2_778974_779108	186	M/P/FB	1195	M/P/FB		
GaLu96scf_2_993116_993709	324	M/P/FB	236	M/P/FB		
GaLu96scf_21_181854_182048	105	M/P/FB	139	M/P/FB		
GaLu96scf_21_562743_563142	211	M/P/FB	77	M/P/FB		
GaLu96scf_22_171118_171475	363	M/P/FB	102	M/P/FB		
GaLu96scf_22_187637_187860	165	M/P/FB	156	M/P/FB		
GaLu96scf_26_559095_559469	540	M/P/FB	944	M/P/FB		
GaLu96scf_3_1113081_1113421	73	M/P/FB	95	M/P/FB		
GaLu96scf_3_1140214_1140529	96	M/P/FB	225	M/P/FB		

GaLu96scf_3_1675827_1676107	147	M/P/FB	336	M/P/FB		
GaLu96scf_3_1818467_1818647	96	M/P/FB	111	M/P/FB		
GaLu96scf_3_586110_586294	191	M/P/FB	237	M/P/FB		
GaLu96scf_31_235519_235921	180	M/P/FB	85	M/P/FB		
GaLu96scf_33_107955_108150	415	M/P/FB	47	M/P/FB		
GaLu96scf_4_1306972_1307375	98	M/P/FB	96	M/P/FB		
GaLu96scf_4_140106_140434	130	M/P/FB	54	M/P/FB		
GaLu96scf_4_148792_149052	72	M/P/FB	73	M/P/FB		
GaLu96scf_4_169285_169400	250	M/P/FB	355	M/P/FB		
GaLu96scf_4_169756_169975	355	M/P/FB	176	M/P/FB		
GaLu96scf_4_619405_619652	63	M/P/FB	146	M/P/FB		
GaLu96scf_4_620240_620393	102	M/P/FB	119	M/P/FB		
GaLu96scf_4_6819_7159	153	M/P/FB	133	M/P/FB		
GaLu96scf_4_941240_941524	57	M/P/FB	580	M/P/FB		
GaLu96scf_41_157014_157254	383	M/P/FB	114	M/P/FB		
GaLu96scf_43_107810_108039	130	M/P/FB	130	M/P/FB		
GaLu96scf_46_78604_78861	491	M/P/FB	59	M/P		
GaLu96scf_47_82969_83169	208	M/P/FB	72	M/P/FB		
GaLu96scf_5_1042559_1042864	69	M/P/FB	75	M/P/FB		
GaLu96scf_5_1100364_1100544	100	M/P/FB	342	M/P/FB		
GaLu96scf_5_521739_521956	104	M/P/FB	483	M/P/FB		
GaLu96scf_6_1278284_1278485	96	M/P/FB	100	M/P/FB		
GaLu96scf_6_284761_285099	206	M/P/FB	187	M/P/FB		
GaLu96scf_6_670719_670947	87	M/P/FB	77	M/P/FB		
GaLu96scf_6_796087_796305	94	M/P/FB	90	M/P/FB		
GaLu96scf_7_1167778_1168011	121	M/P/FB	128	M/P/FB		
GaLu96scf_7_148441_148680	142	M/P/FB	97	M/P/FB		
GaLu96scf_7_527164_527536	133	M/P/FB	82	M/P/FB		
GaLu96scf_7_647053_647313	138	M/P/FB	201	M/P/FB		
GaLu96scf_8_89554_89854	398	M/P/FB	116	M/P/FB		
GaLu96scf_9_1006778_1007111	148	M/P/FB	228	M/P/FB		
GaLu96scf_9_1524047_1524338	73	M/P/FB	108	M/FB		
GaLu96scf_9_729466_729718	89	M/P/FB	133	M/P/FB		
GaLu96scf_9_819996_820255	94	M/P/FB	215	M/P/FB		
GaLu96scf_1_1525668_1525994	89	M/P	107	M/P		
GaLu96scf_1_2106788_2107758	52	M/P	53	M/P		
GaLu96scf_1_2168459_2168738	30	M/P	221	M/P		
GaLu96scf_1_3884241_3884749	80	M/P	244	M/P		
GaLu96scf_1_4761471_4761677	278	M/P	122	M/P		
GaLu96scf_10_547494_547761	100	M/P	114	M/P		
GaLu96scf_13_946765_947266	138	M/P	100	M/P		
GaLu96scf_15_32320_32643	63	M/P	58	M/P		
GaLu96scf_16_207724_208080	550	M/P	63	M/P		
GaLu96scf_16_219107_219318	139	M/P	160	M/P		

GaLu96scf_16_257810_258051	73	M/P	76	M/P		
GaLu96scf_16_26866_27197	72	M/P	573	M/P		
GaLu96scf_16_358893_359309	112	M/P	71	M/P		
GaLu96scf_16_604798_605067	113	M/P	94	M/P/FB		
GaLu96scf_19_664017_664332	110	M/P	59	M/P		
GaLu96scf_20_336853_337127	88	M/P	78	M/P		
GaLu96scf_21_298566_298876	65	M/P	88	M/P		
GaLu96scf_22_124704_125002	416	M/P	66	M/P		
GaLu96scf_22_188017_188188	156	M/P	228	M/P/FB		
GaLu96scf_25_135198_135487	63	M/P	60	M/P		
GaLu96scf_3_1242983_1243427	150	M/P	61	M/P		
GaLu96scf_3_1654565_1654824	90	M/P	62	M/P		
GaLu96scf_30_183359_183598	80	M/P	111	M/P		
GaLu96scf_31_234899_235338	149	M/P	180	M/P		
GaLu96scf_31_259442_259684	59	M/P	54	M/P/FB		
GaLu96scf_31_450431_450722	148	M/P	666	M/P		
GaLu96scf_34_95413_95660	337	M/P	112	M/P		
GaLu96scf_34_95773_96416	112	M/P	78	M/P		
GaLu96scf_4_1120939_1121351	167	M/P	62	M/P		
GaLu96scf_4_1397236_1397474	171	M/P	251	M/P		
GaLu96scf_4_1782074_1782367	62	M/P	54	M/P		
GaLu96scf_41_124935_125140	60	M/P	276	M/P		
GaLu96scf_47_127623_127778	111	M/P	133	M/P		
GaLu96scf_6_1366658_1366983	180	M/P	183	M/P		
GaLu96scf_6_569531_569725	72	M/P	137	M/P		
GaLu96scf_7_455907_456236	72	M/P	354	M/P		
GaLu96scf_7_847536_847794	62	M/P	58	M/P		
GaLu96scf_9_1021257_1021539	103	M/P	49	M/P		
GaLu96scf_9_497310_497494	114	M/P	244	M/P		
GaLu96scf_1_224262_224453	143	M/FB	308	M/P/FB		
GaLu96scf_1_3294448_3294739	92	M/FB	80	M/FB		
GaLu96scf_1_344308_344528	64	M/FB	104	M/FB		
GaLu96scf_1_3585501_3585800	67	M/FB	118	M/FB		
GaLu96scf_1_3968929_3969173	89	M/FB	79	M/FB		
GaLu96scf_1_4023593_4023916	73	M/FB	157	M/FB		
GaLu96scf_1_4341800_4342106	82	M/FB	77	M		
GaLu96scf_1_4775922_4776176	172	M/FB	86	M/FB		
GaLu96scf_1_613208_613436	91	M/FB	89	M		
GaLu96scf_10_991858_992050	74	M/FB	67	FB		
GaLu96scf_11_101360_101610	131	M/FB	284	M/FB		
GaLu96scf_11_596697_596895	93	M/FB	185	M/FB		
GaLu96scf_11_612424_612565	189	M/FB	85	P		
GaLu96scf_11_713891_714215	124	M/FB	448	M/FB		
GaLu96scf_12_358451_358606	241	M/FB	279	M		

GaLu96scf_12_667804_668296	215	M/FB	74	M/FB		
GaLu96scf_12_810175_810343	56	M/FB	72	P		
GaLu96scf_15_528104_528366	63	M/FB	327	M/FB		
GaLu96scf_15_583443_583687	123	M/FB	63	M/FB		
GaLu96scf_16_964984_965284	112	M/FB	122	M/FB		
GaLu96scf_17_630205_630493	80	M/FB	75	M/FB		
GaLu96scf_18_567602_567953	93	M/FB	165	FB		
GaLu96scf_19_175500_175732	359	M/FB	223	M		
GaLu96scf_19_506883_507372	267	M/FB	105	M/FB		
GaLu96scf_2_1990385_1990615	74	M/FB	76	M/FB		
GaLu96scf_20_272630_272957	60	M/FB	331	M/FB		
GaLu96scf_20_869159_869376	67	M/FB	123	M/FB		
GaLu96scf_21_34416_34722	48	M/FB	322	M/FB		
GaLu96scf_21_411133_411598	64	M/FB	73	M/FB		
GaLu96scf_21_737969_738131	84	M/FB	97	M/FB		
GaLu96scf_21_814520_814865	943	M/FB	79	M/FB		
GaLu96scf_22_682319_682655	88	M/FB	67	M/FB		
GaLu96scf_23_532213_532340	247	M/FB	331	FB		
GaLu96scf_24_34110_34346	259	M/FB	213	M/FB		
GaLu96scf_24_89145_89484	66	M/FB	160	M/FB		
GaLu96scf_26_117579_117907	81	M/FB	197	M/FB		
GaLu96scf_27_318775_318952	292	M/FB	54	M/FB		
GaLu96scf_27_432715_433523	97	M/FB	91	M/FB		
GaLu96scf_28_525829_526191	70	M/FB	156	M/FB		
GaLu96scf_29_246147_246434	104	M/FB	157	M/FB		
GaLu96scf_29_261029_261362	178	M/FB	324	M/FB		
GaLu96scf_3_1102021_1102243	139	M/FB	170	M/FB		
GaLu96scf_3_1178883_1179123	211	M/FB	149	M/FB		
GaLu96scf_3_1329424_1329647	78	M/FB	58	M/FB		
GaLu96scf_3_1674984_1675076	180	M/FB	228	M		
GaLu96scf_3_1675305_1675525	228	M/FB	103	M/FB		
GaLu96scf_3_1899354_1899568	448	M/FB	225	M/FB		
GaLu96scf_31_140867_141452	79	M/FB	77	M/FB		
GaLu96scf_32_29553_29713	89	M/FB	132	M/P		
GaLu96scf_32_314680_314960	207	M/FB	70	M/FB		
GaLu96scf_34_36492_36748	157	M/FB	360	M/FB		
GaLu96scf_35_314043_314229	94	M/FB	18	M/FB		
GaLu96scf_4_139725_139975	57	M/FB	130	M/FB		
GaLu96scf_4_619799_620137	146	M/FB	102	M		
GaLu96scf_4_942652_942888	80	M/FB	316	M/P/FB		
GaLu96scf_40_145087_145321	91	M/FB	136	M/FB		
GaLu96scf_42_187462_188053	58	M/FB	245	M/FB		
GaLu96scf_46_79582_80089	63	M/FB	229	M/FB		
GaLu96scf_47_129180_129519	63	M/FB	166	M/FB		

GaLu96scf_47_37444_37728	188	M/FB	261	FB		
GaLu96scf_5_1397507_1397914	81	M/FB	67	M		
GaLu96scf_5_681470_681784	168	M/FB	78	M/P/FB		
GaLu96scf_7_1425350_1425884	103	M/FB	68	M/FB		
GaLu96scf_8_1061304_1061464	72	M/FB	71	M		
GaLu96scf_8_390580_390915	110	M/FB	66	M/FB		
GaLu96scf_8_586612_586805	127	M/FB	82	M/FB		
GaLu96scf_8_866919_867068	69	M/FB	158	FB		
GaLu96scf_8_997325_997534	208	M/FB	81	M/FB		
GaLu96scf_9_1059347_1059821	121	M/FB	100	M/FB		
GaLu96scf_9_1062767_1063090	111	M/FB	66	FB		
GaLu96scf_9_1106364_1106552	71	M/FB	80	M/FB		
GaLu96scf_1_1779304_1779570	55	P/FB	96	P/FB		
GaLu96scf_1_2659518_2659735	153	P/FB	63	P/FB		
GaLu96scf_1_2965726_2965968	59	P/FB	109	P/FB		
GaLu96scf_1_89438_89611	182	P/FB	70	M/P/FB		
GaLu96scf_1_94170_94407	366	P/FB	133	P/FB		
GaLu96scf_11_708674_708916	72	P/FB	75	P/FB		
GaLu96scf_14_736748_736996	94	P/FB	79	P/FB		
GaLu96scf_15_50637_50979	45	P/FB	104	P/FB		
GaLu96scf_15_838752_839281	227	P/FB	465	P/FB		
GaLu96scf_19_819732_819912	100	P/FB	947	M/P/FB		
GaLu96scf_20_845487_845780	188	P/FB	67	P/FB		
GaLu96scf_3_1014919_1015212	50	P/FB	116	P/FB		
GaLu96scf_3_1117772_1118098	528	P/FB	197	P/FB		
GaLu96scf_40_120652_120959	72	P/FB	84	P/FB		
GaLu96scf_5_1002250_1002412	136	P/FB	110	P/FB		
GaLu96scf_51_90105_90342	102	P/FB	72	P/FB		
GaLu96scf_6_1347523_1348145	113	P/FB	90	P/FB		
GaLu96scf_7_1311167_1311481	104	P/FB	280	P/FB		
GaLu96scf_7_1367812_1368149	87	P/FB	72	P		
GaLu96scf_7_207334_207675	123	P/FB	59	P/FB		
GaLu96scf_7_238736_240226	113	P/FB	176	P/FB		
GaLu96scf_1_1050652_1050867	46	M	53	M		
GaLu96scf_1_111570_111746	76	M	84	M		
GaLu96scf_1_1186490_1186746	73	M	64	M		
GaLu96scf_1_1350356_1350654	78	M	151	M/FB		
GaLu96scf_1_1351823_1352120	64	M	105	M		
GaLu96scf_1_1408495_1408813	74	M	40	M		
GaLu96scf_1_1427859_1428495	76	M	76	M		
GaLu96scf_1_1587853_1588196	70	M	14	M		
GaLu96scf_1_176092_176863	59	M	47	M		
GaLu96scf_1_1853379_1853571	50	M	59	M		
GaLu96scf_1_2046624_2046987	60	M	77	M		

GaLu96scf_1_2062031_2062166	55	M	51	FB		
GaLu96scf_1_2065870_2066251	49	M	56	M		
GaLu96scf_1_2460056_2460446	66	M	69	M		
GaLu96scf_1_2485437_2485732	56	M	136	M		
GaLu96scf_1_2607455_2607804	238	M	55	M		
GaLu96scf_1_2691071_2691374	61	M	68	M		
GaLu96scf_1_2716972_2717313	63	M	51	M		
GaLu96scf_1_2837166_2837435	56	M	60	M		
GaLu96scf_1_3063699_3063973	157	M	105	M		
GaLu96scf_1_3183139_3183352	58	M	67	M		
GaLu96scf_1_336402_336711	294	M	53	M		
GaLu96scf_1_3450815_3451106	26	M	101	M		
GaLu96scf_1_3477024_3477420	51	M	73	M		
GaLu96scf_1_3483992_3484226	73	M	63	M		
GaLu96scf_1_3736883_3737106	116	M	121	M		
GaLu96scf_1_4116640_4117290	158	M	204	M		
GaLu96scf_1_4204512_4204829	69	M	52	M		
GaLu96scf_1_447870_448211	75	M	82	M		
GaLu96scf_1_4548752_4549297	65	M	121	M		
GaLu96scf_1_481248_481622	90	M	61	M		
GaLu96scf_1_4812876_4813249	103	M	40	M		
GaLu96scf_1_55000_55251	97	M	74	M		
GaLu96scf_1_577199_577434	54	M	64	M		
GaLu96scf_1_613526_613932	89	M	229	M/FB		
GaLu96scf_1_749484_749685	54	M	53	M		
GaLu96scf_1_83631_83927	77	M	60	M		
GaLu96scf_1_84704_84952	73	M	69	M		
GaLu96scf_1_85022_85476	69	M	71	M		
GaLu96scf_1_872394_872739	77	M	120	M		
GaLu96scf_10_1027170_1027578	60	M	50	M		
GaLu96scf_10_231140_231396	62	M	58	M		
GaLu96scf_10_61699_61980	71	M	102	M		
GaLu96scf_10_633173_633434	70	M	341	M/P/FB		
GaLu96scf_10_935596_935959	107	M	86	M		
GaLu96scf_10_941606_941818	78	M	96	M		
GaLu96scf_10_956005_956405	593	M	153	M		
GaLu96scf_10_989288_989517	183	M	44	M		
GaLu96scf_11_1099916_1100405	51	M	72	M		
GaLu96scf_11_132103_132409	631	M	99	M		
GaLu96scf_11_377445_377622	167	M	229	M		
GaLu96scf_11_433670_433872	516	M	71	M		
GaLu96scf_11_483461_483693	62	M	64	M		
GaLu96scf_11_518310_518413	70	M	143	P		
GaLu96scf_12_1077467_1077895	859	M	52	M		

GaLu96scf_12_162281_162525	97	M	122	M		
GaLu96scf_12_358886_359047	279	M	98	M/FB		
GaLu96scf_12_936751_937355	60	M	61	M		
GaLu96scf_12_953525_953752	75	M	198	M		
GaLu96scf_13_1077937_1078212	167	M	349	M		
GaLu96scf_13_1106880_1107339	8	M	54	M		
GaLu96scf_13_299794_300034	64	M	142	M/P/FB		
GaLu96scf_13_548130_548451	123	M	52	M		
GaLu96scf_13_572959_573298	79	M	102	M		
GaLu96scf_13_62664_63412	94	M	118	M		
GaLu96scf_13_66924_67136	95	M	104	M		
GaLu96scf_13_67467_67663	75	M	47	M		
GaLu96scf_13_70956_71305	70	M	144	M		
GaLu96scf_13_937874_938103	52	M	56	M		
GaLu96scf_13_985328_985970	82	M	61	M		
GaLu96scf_14_295185_295479	79	M	91	M/P		
GaLu96scf_14_819657_819979	585	M	52	M		
GaLu96scf_15_511452_512189	59	M	322	M		
GaLu96scf_15_561186_561534	141	M	62	M		
GaLu96scf_15_572974_573315	69	M	69	M		
GaLu96scf_15_578105_578307	87	M	154	M/P/FB		
GaLu96scf_15_578462_578706	154	M	91	M		
GaLu96scf_16_25083_25432	94	M	65	M		
GaLu96scf_16_544240_544486	63	M	653	M		
GaLu96scf_17_257434_257824	49	M	54	M		
GaLu96scf_17_265349_265533	69	M	64	M/FB		
GaLu96scf_17_309808_309938	88	M	101	M/P/FB		
GaLu96scf_17_425284_425663	106	M	57	M		
GaLu96scf_17_470660_470904	61	M	54	M		
GaLu96scf_17_474886_475109	130	M	79	M		
GaLu96scf_17_482106_482368	66	M	104	M		
GaLu96scf_17_50569_50828	25	M	69	M		
GaLu96scf_17_737815_738454	48	M	51	M		
GaLu96scf_17_791933_792475	51	M	348	M/P/FB		
GaLu96scf_17_924788_925156	103	M	78	M		
GaLu96scf_18_621320_621401	83	M	236	M		
GaLu96scf_18_621762_621839	94	M	125	M/P/FB		
GaLu96scf_19_436708_437006	61	M	137	M		
GaLu96scf_19_700802_701028	71	M	112	M		
GaLu96scf_2_1017108_1017376	78	M	57	M		
GaLu96scf_2_1177722_1177901	386	M	216	M		
GaLu96scf_2_145953_146524	55	M	63	M		
GaLu96scf_2_1497801_1497965	98	M	88	M		
GaLu96scf_2_1501802_1502294	76	M	97	M		

GaLu96scf_2_1579790_1580086	61	M	61	M		
GaLu96scf_2_1629480_1629720	56	M	69	M		
GaLu96scf_2_1724880_1725264	48	M	60	M		
GaLu96scf_2_2230730_2231131	54	M	50	M		
GaLu96scf_2_2292444_2292702	67	M	152	M		
GaLu96scf_2_775722_776014	116	M	68	M		
GaLu96scf_2_79752_80226	98	M	105	M		
GaLu96scf_2_821935_822137	90	M	4	M		
GaLu96scf_2_873081_873396	4	M	64	M		
GaLu96scf_20_71483_71788	55	M	68	M		
GaLu96scf_21_12421_12838	62	M	127	M		
GaLu96scf_21_26613_26895	55	M	260	M		
GaLu96scf_22_119730_119804	112	M	81	M		
GaLu96scf_22_188417_188634	228	M	79	M/P		
GaLu96scf_22_744830_745187	97	M	82	M		
GaLu96scf_23_532672_532829	331	M	209	M/P/FB		
GaLu96scf_23_84546_84768	442	M	59	M		
GaLu96scf_24_19069_19388	94	M	74	M/FB		
GaLu96scf_24_224193_224464	84	M	112	M		
GaLu96scf_24_410157_410485	53	M	501	M		
GaLu96scf_24_642832_643032	78	M	313	M		
GaLu96scf_25_188834_189102	123	M	75	M		
GaLu96scf_25_304321_304541	86	M	91	M		
GaLu96scf_25_397276_397957	52	M	52	M		
GaLu96scf_25_427065_427245	195	M	88	M/FB		
GaLu96scf_25_456468_456894	167	M	61	M		
GaLu96scf_26_154573_154797	167	M	106	M		
GaLu96scf_26_186682_187294	50	M	53	M		
GaLu96scf_26_278041_278380	83	M	74	M		
GaLu96scf_26_449240_449496	79	M	103	M		
GaLu96scf_27_193935_194803	73	M	84	M		
GaLu96scf_27_428519_428861	67	M	42	M		
GaLu96scf_27_499582_499912	56	M	56	M		
GaLu96scf_27_502790_502971	114	M	139	M		
GaLu96scf_28_430888_431088	67	M	106	M		
GaLu96scf_28_434983_435391	465	M	65	M		
GaLu96scf_28_437148_437556	474	M	66	M		
GaLu96scf_28_502114_502375	103	M	81	M/P		
GaLu96scf_29_76196_76542	67	M	131	M		
GaLu96scf_3_1028636_1029170	85	M	206	M		
GaLu96scf_3_1121840_1122081	104	M	54	M		
GaLu96scf_3_1135050_1135563	117	M	43	M		
GaLu96scf_3_1176468_1176724	334	M	84	M		
GaLu96scf_3_1237267_1237545	61	M	68	M		

GaLu96scf_3_1296420_1296820	51	M	142	M		
GaLu96scf_3_1338202_1338773	120	M	37	M		
GaLu96scf_3_1527434_1527691	53	M	55	M		
GaLu96scf_3_169589_170273	73	M	341	M		
GaLu96scf_3_702395_703282	101	M	107	M		
GaLu96scf_30_188966_189297	93	M	200	M		
GaLu96scf_31_202028_202379	52	M	165	M/P/FB		
GaLu96scf_31_234356_234749	244	M	149	M		
GaLu96scf_31_320750_320941	99	M	113	M		
GaLu96scf_31_36002_36247	54	M	55	M		
GaLu96scf_31_98237_98568	45	M	61	M		
GaLu96scf_32_167460_167705	66	M	120	M		
GaLu96scf_32_182781_183194	75	M	76	M		
GaLu96scf_32_199917_200290	65	M	65	M		
GaLu96scf_32_30470_30827	280	M	148	M		
GaLu96scf_33_18524_19026	62	M	140	M		
GaLu96scf_34_127114_127406	77	M	66	M		
GaLu96scf_34_30502_30896	80	M	95	M		
GaLu96scf_37_224737_225370	47	M	40	M/P/FB		
GaLu96scf_38_28561_28821	54	M	59	M		
GaLu96scf_4_1167577_1167835	50	M	69	M		
GaLu96scf_4_121367_121788	370	M	46	M		
GaLu96scf_4_1266072_1266428	166	M	54	M		
GaLu96scf_4_1332782_1333094	83	M	76	M		
GaLu96scf_4_1411238_1411498	94	M	88	M		
GaLu96scf_4_1470917_1471133	94	M	67	M/P		
GaLu96scf_4_388390_388725	57	M	288	M/FB		
GaLu96scf_4_546220_546606	59	M	98	M		
GaLu96scf_4_638508_638817	89	M	110	M		
GaLu96scf_4_692800_693127	61	M	64	M/P		
GaLu96scf_4_82007_82257	51	M	86	M		
GaLu96scf_4_869298_869525	65	M	54	M		
GaLu96scf_40_201337_201609	65	M	62	M		
GaLu96scf_41_74161_74429	68	M	202	M		
GaLu96scf_41_87866_88462	33	M	315	M		
GaLu96scf_45_107155_107404	72	M	153	M		
GaLu96scf_45_134269_134540	66	M	39	M/FB		
GaLu96scf_45_47325_47496	101	M	28	M		
GaLu96scf_45_69519_69787	106	M	84	M		
GaLu96scf_47_92737_93159	49	M	53	M		
GaLu96scf_5_1381747_1382048	62	M	69	M		
GaLu96scf_5_1478381_1478658	71	M	64	M		
GaLu96scf_5_288366_288558	105	M	122	M/P		
GaLu96scf_5_517364_518001	124	M	135	M		

GaLu96scf_5_540190_540464	58	M	51	M		
GaLu96scf_5_626118_626279	128	M	179	M		
GaLu96scf_5_683996_684096	80	M	82	M/FB		
GaLu96scf_5_686474_686732	52	M	126	M		
GaLu96scf_5_811662_812027	73	M	55	M		
GaLu96scf_6_124526_124939	63	M	65	M		
GaLu96scf_6_217787_218029	64	M	101	M		
GaLu96scf_6_782597_782901	87	M	249	M		
GaLu96scf_6_813635_813907	93	M	79	M		
GaLu96scf_7_1121284_1121658	71	M	66	M		
GaLu96scf_7_1463041_1463287	134	M	54	M		
GaLu96scf_7_499827_500109	74	M	116	M		
GaLu96scf_7_647515_647836	201	M	120	M		
GaLu96scf_7_848191_848404	123	M	66	M/P		
GaLu96scf_8_1023680_1023844	309	M	78	M		
GaLu96scf_8_1086287_1086584	71	M	71	M		
GaLu96scf_8_1164705_1165005	112	M	93	M		
GaLu96scf_8_1226373_1226573	77	M	108	M		
GaLu96scf_8_1303781_1304246	64	M	109	M		
GaLu96scf_8_1384261_1384494	64	M	70	M		
GaLu96scf_8_1393722_1393919	64	M	60	M		
GaLu96scf_8_1546120_1546461	60	M	64	M		
GaLu96scf_8_413101_413324	94	M	224	M		
GaLu96scf_8_463132_463471	161	M	56	M		
GaLu96scf_8_500709_501111	84	M	56	M		
GaLu96scf_8_531717_531891	180	M	170	M		
GaLu96scf_9_1185508_1185778	65	M	299	M		
GaLu96scf_9_1336812_1337034	67	M	61	M		
GaLu96scf_9_1425968_1426155	108	M	101	M		
GaLu96scf_9_1508935_1509106	93	M	124	M		
GaLu96scf_9_583208_583481	138	M	126	M		
GaLu96scf_9_60299_60551	89	M	65	M		
GaLu96scf_9_636235_636429	59	M	70	M/FB		
GaLu96scf_9_917450_917719	68	M	53	M		
GaLu96scf_1_1739242_1739494	51	P	52	P		
GaLu96scf_1_3122931_3123121	108	P	174	P		
GaLu96scf_1_3139273_3139569	49	P	57	P		
GaLu96scf_10_1255309_1255563	515	P	45	P		
GaLu96scf_10_325914_326491	523	P	82	P		
GaLu96scf_10_339814_340017	68	P	60	P/FB		
GaLu96scf_11_100320_100404	112	P	132	P/FB		
GaLu96scf_12_722155_722381	60	P	57	P		
GaLu96scf_12_956568_957270	135	P	62	P		
GaLu96scf_13_760855_761049	76	P	59	P		

GaLu96scf_13_91326_91598	53	P	81	P		
GaLu96scf_14_1069126_1069454	204	P	104	P		
GaLu96scf_14_1069559_1069812	104	P	61	P		
GaLu96scf_14_269088_269255	122	P	437	P/FB		
GaLu96scf_14_579214_579573	64	P	53	P		
GaLu96scf_14_626976_627294	57	P	107	P		
GaLu96scf_14_836124_836543	190	P	58	P		
GaLu96scf_14_87845_88236	216	P	84	P		
GaLu96scf_15_512512_513690	322	P	54	P		
GaLu96scf_16_375080_375362	78	P	98	P		
GaLu96scf_16_375461_375677	98	P	57	P		
GaLu96scf_16_549145_549300	96	P	59	P		
GaLu96scf_17_427671_428169	57	P	50	P		
GaLu96scf_18_554297_554670	118	P	82	P		
GaLu96scf_19_682545_683129	105	P	183	P		
GaLu96scf_2_1664767_1665032	52	P	80	P		
GaLu96scf_2_813532_814126	141	P	95	P		
GaLu96scf_20_865069_865450	50	P	74	P		
GaLu96scf_22_100403_100647	55	P	69	P		
GaLu96scf_22_686708_687026	90	P	60	P		
GaLu96scf_22_775066_775273	371	P	1771	P		
GaLu96scf_24_113250_113461	148	P	91	P		
GaLu96scf_24_123224_123543	211	P	68	P		
GaLu96scf_26_154904_155440	106	P	172	P		
GaLu96scf_26_190303_190716	72	P	98	P		
GaLu96scf_29_307671_307865	103	P	73	P		
GaLu96scf_29_441073_441605	49	P	614	P		
GaLu96scf_3_1117008_1117243	157	P	528	P		
GaLu96scf_3_1134626_1134932	83	P	117	P		
GaLu96scf_3_1340129_1340492	130	P	64	P		
GaLu96scf_3_330893_331222	96	P	82	P		
GaLu96scf_31_178594_178809	243	P	68	P		
GaLu96scf_31_309685_310046	51	P	58	P		
GaLu96scf_37_94304_94612	108	P	58	P		
GaLu96scf_39_56368_56625	57	P	85	P		
GaLu96scf_4_1159977_1160256	231	P	98	P		
GaLu96scf_4_1565519_1565823	91	P	99	P		
GaLu96scf_4_376373_376587	77	P	66	P		
GaLu96scf_4_6272_6665	91	P	153	P		
GaLu96scf_4_716722_717017	63	P	175	P		
GaLu96scf_4_984554_984792	99	P	91	P		
GaLu96scf_43_58185_58576	48	P	52	P		
GaLu96scf_45_26854_27149	95	P	114	P		
GaLu96scf_46_55071_55230	229	P	55	P		

GaLu96scf_49_50406_50649	57	P	68	P		
GaLu96scf_5_1254833_1255305	55	P	69	M/P		
GaLu96scf_5_522440_522697	483	P	125	P		
GaLu96scf_6_1018372_1018598	60	P	91	P		
GaLu96scf_6_1245249_1245602	122	P	53	P		
GaLu96scf_6_267038_267281	70	P	64	P		
GaLu96scf_6_271266_271532	50	P	51	P		
GaLu96scf_6_370594_370941	69	P	118	P		
GaLu96scf_6_784412_784620	81	P	57	P		
GaLu96scf_7_848001_848067	64	P	123	M/P/FB		
GaLu96scf_8_1207123_1207684	101	P	73	P		
GaLu96scf_8_1294273_1294858	538	P	50	P		
GaLu96scf_8_1563704_1564128	62	P	107	P		
GaLu96scf_8_498907_499140	80	P	95	P		
GaLu96scf_8_867730_868021	207	P	58	P		
GaLu96scf_9_249538_249788	60	P	63	P		
GaLu96scf_9_766111_766240	75	P	91	P		
GaLu96scf_1_1148535_1148700	165	FB	54	FB		
GaLu96scf_1_1319541_1319959	78	FB	71	FB		
GaLu96scf_1_1468308_1468583	106	FB	82	FB		
GaLu96scf_1_2190249_2190615	76	FB	144	FB		
GaLu96scf_1_2347108_2347327	91	FB	154	M/P/FB		
GaLu96scf_1_3517885_3518004	204	FB	57	P		
GaLu96scf_1_3734072_3734489	161	FB	77	FB		
GaLu96scf_1_4037375_4037662	86	FB	49	FB		
GaLu96scf_1_4712603_4712936	93	FB	76	FB		
GaLu96scf_1_807086_807416	143	FB	72	FB		
GaLu96scf_1_815100_815299	53	FB	57	FB		
GaLu96scf_1_823963_824340	41	FB	142	FB		
GaLu96scf_10_1051143_1051484	72	FB	91	FB		
GaLu96scf_10_864091_864282	61	FB	35	FB		
GaLu96scf_11_1208461_1208707	80	FB	408	FB		
GaLu96scf_11_432871_433153	225	FB	516	FB		
GaLu96scf_11_715123_715339	82	FB	91	M/P/FB		
GaLu96scf_12_263165_263451	53	FB	55	FB		
GaLu96scf_12_406586_406748	66	FB	59	FB		
GaLu96scf_12_703142_703558	48	FB	81	FB		
GaLu96scf_12_757741_758109	60	FB	67	FB		
GaLu96scf_12_842952_843260	96	FB	47	FB		
GaLu96scf_13_301980_302188	52	FB	239	FB		
GaLu96scf_13_452689_453120	78	FB	83	FB		
GaLu96scf_13_976810_977053	4	FB	186	FB		
GaLu96scf_14_340584_340860	55	FB	316	P/FB		
GaLu96scf_14_341177_341518	316	FB	52	FB		

GaLu96scf_14_886519_886708	74	FB	100	FB		
GaLu96scf_15_1002028_1002246	68	FB	75	M		
GaLu96scf_15_180233_180491	62	FB	93	FB		
GaLu96scf_15_633635_634004	73	FB	70	FB		
GaLu96scf_15_825259_825463	269	FB	85	FB		
GaLu96scf_16_647368_647529	128	FB	96	FB		
GaLu96scf_17_335371_335566	51	FB	127	FB		
GaLu96scf_18_167689_168072	97	FB	70	FB		
GaLu96scf_18_188754_188962	82	FB	66	FB		
GaLu96scf_18_432723_433059	55	FB	60	FB		
GaLu96scf_18_532142_532405	66	FB	75	FB		
GaLu96scf_19_130489_130885	64	FB	55	FB		
GaLu96scf_19_140306_140674	179	FB	64	FB		
GaLu96scf_19_248473_248676	207	FB	130	FB		
GaLu96scf_19_528439_528683	67	FB	117	FB		
GaLu96scf_2_1035829_1036005	50	FB	129	FB		
GaLu96scf_2_1547585_1547797	225	FB	177	FB		
GaLu96scf_2_755936_756119	58	FB	58	FB		
GaLu96scf_2_802905_803128	218	FB	61	FB		
GaLu96scf_20_142969_143191	350	FB	156	M/P/FB		
GaLu96scf_20_277215_277725	115	FB	101	FB		
GaLu96scf_20_277827_278019	101	FB	60	FB		
GaLu96scf_20_507012_507274	107	FB	51	FB		
GaLu96scf_21_135392_135697	113	FB	133	FB		
GaLu96scf_21_7286_7501	170	FB	92	FB		
GaLu96scf_22_93007_93254	101	FB	103	FB		
GaLu96scf_24_246613_246869	63	FB	67	FB		
GaLu96scf_26_531078_531338	77	FB	78	FB		
GaLu96scf_26_548301_548661	72	FB	366	FB		
GaLu96scf_29_175846_176123	134	FB	67	FB		
GaLu96scf_29_249130_249358	30	FB	159	FB		
GaLu96scf_29_346438_346649	70	FB	86	FB		
GaLu96scf_3_1236789_1237003	416	FB	102	FB		
GaLu96scf_3_126852_127125	212	FB	112	FB		
GaLu96scf_3_1279545_1279732	87	FB	144	FB		
GaLu96scf_3_1279877_1280046	144	FB	105	FB		
GaLu96scf_3_179057_179236	77	FB	70	FB		
GaLu96scf_3_1857838_1858067	82	FB	69	FB		
GaLu96scf_3_1868925_1869368	94	FB	78	FB		
GaLu96scf_3_1926382_1926600	58	FB	50	FB		
GaLu96scf_3_221040_221312	54	FB	66	FB		
GaLu96scf_30_58487_58623	78	FB	98	M/FB		
GaLu96scf_31_27644_27912	59	FB	162	FB		
GaLu96scf_31_375583_375831	114	FB	85	FB		

GaLu96scf_33_112982_113199	55	FB	58	FB		
GaLu96scf_34_58949_59214	108	FB	208	FB		
GaLu96scf_35_180898_181075	179	FB	81	FB		
GaLu96scf_36_74533_75102	68	FB	78	FB		
GaLu96scf_4_1146831_1147097	102	FB	48	FB		
GaLu96scf_4_1218208_1218787	53	FB	61	FB		
GaLu96scf_4_408318_409324	85	FB	61	FB		
GaLu96scf_4_772594_773018	203	FB	52	FB		
GaLu96scf_41_110911_111187	56	FB	51	FB		
GaLu96scf_41_18285_18642	64	FB	215	FB		
GaLu96scf_44_32765_33242	100	FB	181	FB		
GaLu96scf_46_125330_125501	73	FB	98	M/FB		
GaLu96scf_5_1117503_1117783	71	FB	90	FB		
GaLu96scf_5_1142068_1142814	48	FB	52	FB		
GaLu96scf_5_1257569_1258000	56	FB	110	FB		
GaLu96scf_5_275854_276060	315	FB	531	FB		
GaLu96scf_5_276592_276854	531	FB	196	FB		
GaLu96scf_5_420067_420267	145	FB	151	P/FB		
GaLu96scf_5_76587_76661	222	FB	73	FB		
GaLu96scf_5_781356_782024	133	FB	84	FB		
GaLu96scf_51_11723_11888	86	FB	56	FB		
GaLu96scf_6_598901_599233	4	FB	34	M/P/FB		
GaLu96scf_7_1441990_1442180	55	FB	186	FB		
GaLu96scf_7_1465011_1465205	79	FB	148	FB		
GaLu96scf_8_179541_179841	102	FB	70	FB		
GaLu96scf_8_224458_224652	58	FB	104	FB		
GaLu96scf_8_460468_460584	75	FB	78	M		
GaLu96scf_8_58909_59252	120	FB	54	FB		
GaLu96scf_8_703476_703647	3	FB	97	FB		
GaLu96scf_9_1287215_1287962	135	FB	53	FB		
GaLu96scf_9_614617_614812	62	FB	50	FB		
GaLu96scf_9_737529_738069	57	FB	78	FB		
GaLu96scf_18_187339_187580	75	M			785	M
GaLu96scf_19_99310_99744	151	M/P			195	M
GaLu96scf_1_1097860_1097994	71	P			87	P
GaLu96scf_1_1137382_1137492	53	P/FB			53	P
GaLu96scf_1_1138269_1138680	412	P			60	P
GaLu96scf_1_1143289_1143365	51	M/P			63	P
GaLu96scf_1_1288668_1288774	69	P			54	P
GaLu96scf_1_1292918_1293088	58	M/P			60	P
GaLu96scf_1_1349897_1350078	670	M/P/FB			52	P
GaLu96scf_1_14418_14544	92	P			78	P
GaLu96scf_1_1560803_1560858	93	M/P/FB			64	P
GaLu96scf_1_1745033_1745151	50	P			53	P

GaLu96scf_1_1832017_1832084	105	M/P/FB			146	P
GaLu96scf_1_2004521_2004717	85	M/P/FB			22	P
GaLu96scf_1_2105119_2105328	54	P			58	P
GaLu96scf_1_2214466_2214600	60	P			57	P
GaLu96scf_1_223979_224118	62	P/FB			143	P
GaLu96scf_1_2259921_2260132	48	M/P			52	P
GaLu96scf_1_2297344_2297554	83	P			57	P
GaLu96scf_1_2346905_2347016	102	M/P/FB			91	P
GaLu96scf_1_2395173_2395228	68	P			62	P
GaLu96scf_1_2406810_2407023	51	P			55	P
GaLu96scf_1_2416564_2416759	61	P			51	P
GaLu96scf_1_2457825_2458004	57	P			57	P
GaLu96scf_1_2496686_2496820	64	M/P/FB			48	P
GaLu96scf_1_2575581_2575618	71	M/P			59	P
GaLu96scf_1_2753942_2754067	62	P			58	P
GaLu96scf_1_2937155_2937516	57	P			83	P
GaLu96scf_1_2942213_2942306	69	P			51	P
GaLu96scf_1_3125046_3125111	50	P			58	P
GaLu96scf_1_3142092_3142252	330	M/P			59	P
GaLu96scf_1_3332784_3332953	453	M/P/FB			71	P
GaLu96scf_1_3517326_3517373	108	P/FB			58	P
GaLu96scf_1_3551960_3552082	53	M/P			51	P
GaLu96scf_1_3622004_3622097	62	M/P/FB			61	P
GaLu96scf_1_3766876_3766951	47	P			61	P
GaLu96scf_1_3850733_3850917	54	P			73	P
GaLu96scf_1_3922878_3922975	59	P			50	P
GaLu96scf_1_4020862_4020917	186	P/FB			81	P
GaLu96scf_1_4109049_4109181	58	P			53	P
GaLu96scf_1_4219277_4219338	70	P			71	P
GaLu96scf_1_4323938_4324121	356	M/P/FB			63	P
GaLu96scf_1_4342184_4342641	77	P			141	P
GaLu96scf_1_440858_441035	90	M/P/FB			108	P
GaLu96scf_1_441144_441245	108	M/P/FB			70	P
GaLu96scf_1_4437569_4437658	147	P/FB			86	P
GaLu96scf_1_564557_564657	316	P/FB			50	P
GaLu96scf_1_687514_687794	131	P			51	P
GaLu96scf_1_822816_822959	117	M/P/FB			197	P
GaLu96scf_1_89101_89255	271	M/P/FB			182	P
GaLu96scf_1_988574_988966	63	P			50	P
GaLu96scf_10_1309498_1309689	54	M/P/FB			57	P
GaLu96scf_10_239610_239683	399	P/FB			36	P
GaLu96scf_10_552706_553173	108	M/P			123	P
GaLu96scf_10_57748_57823	70	M/P/FB			54	P
GaLu96scf_10_633036_633102	318	M/P/FB			70	P

GaLu96scf_10_656443_656633	49	P			55	P
GaLu96scf_10_721197_721208	167	M/P/FB			91	P
GaLu96scf_10_955144_955287	55	M/P			12	P
GaLu96scf_11_100041_100207	196	M/P/FB			112	P
GaLu96scf_11_1153012_1153146	280	M/P/FB			50	P
GaLu96scf_11_1234416_1234525	57	P			193	P
GaLu96scf_11_1309786_1310023	90	P/FB			58	P
GaLu96scf_11_183914_184074	134	P			84	P
GaLu96scf_11_289556_289672	56	P			63	P
GaLu96scf_11_343167_343535	119	P			51	P
GaLu96scf_11_379444_379671	66	P/FB			62	P
GaLu96scf_11_518070_518239	70	P			70	P
GaLu96scf_11_611428_611602	51	P			84	P
GaLu96scf_11_611687_611804	84	P			79	P
GaLu96scf_11_612651_612711	85	M/P			81	P
GaLu96scf_11_714664_714850	448	M/P/FB			66	P
GaLu96scf_11_748237_748429	52	P			50	P
GaLu96scf_11_965780_966388	144	M/P			74	P
GaLu96scf_12_387721_387910	98	M/P/FB			63	P
GaLu96scf_12_451474_451751	219	M/P/FB			162	P
GaLu96scf_12_671396_671553	69	P			56	P
GaLu96scf_12_672275_672382	39	P			53	P
GaLu96scf_12_711922_711994	327	P/FB			64	P
GaLu96scf_12_809983_810118	58	P			56	P
GaLu96scf_12_883406_884450	61	P			61	P
GaLu96scf_12_925185_925315	55	P			74	P
GaLu96scf_13_1002066_1002148	49	M/P			40	P
GaLu96scf_13_1035832_1035986	406	P			604	P
GaLu96scf_13_299485_299556	191	P/FB			81	P
GaLu96scf_13_299638_299729	81	P			64	P
GaLu96scf_13_404666_404848	72	P			105	P
GaLu96scf_13_436487_436558	140	M/P/FB			82	P
GaLu96scf_13_661306_661342	171	P			56	P
GaLu96scf_13_711667_711839	62	P/FB			63	P
GaLu96scf_13_716319_716393	80	M/P			78	P
GaLu96scf_13_719131_719214	69	P			55	P
GaLu96scf_14_1000040_1000166	59	P			151	P
GaLu96scf_14_294888_295105	195	P			79	P
GaLu96scf_14_340073_340528	157	P			55	P
GaLu96scf_14_353431_353777	548	P			67	P
GaLu96scf_14_380775_380941	118	P/FB			80	P
GaLu96scf_14_521364_521454	56	P			65	P
GaLu96scf_14_731709_731926	119	M/P/FB			50	P
GaLu96scf_14_836602_837096	58	P			59	P

GaLu96scf_14_8634_8904	58	P			54	P
GaLu96scf_14_868258_868384	59	P			51	P
GaLu96scf_14_895159_895431	52	P			69	P
GaLu96scf_14_912475_912675	62	P			65	P
GaLu96scf_14_989976_990067	61	M/P/FB			66	P
GaLu96scf_14_997917_998095	111	M/P			75	P
GaLu96scf_15_577641_577715	101	M/P			84	P
GaLu96scf_15_577800_577873	84	M/P/FB			81	P
GaLu96scf_15_719431_719502	91	P			72	P
GaLu96scf_15_763952_764189	85	P			55	P
GaLu96scf_15_845785_845910	594	P			63	P
GaLu96scf_15_888746_889026	275	P			97	P
GaLu96scf_15_889124_889547	97	M/P			44	P
GaLu96scf_15_894656_894912	184	M/P/FB			58	P
GaLu96scf_15_966920_967044	992	M/P/FB			112	P
GaLu96scf_16_275739_275762	61	P			81	P
GaLu96scf_16_604576_604684	934	M/P/FB			113	P
GaLu96scf_16_622307_622791	316	P/FB			25	P
GaLu96scf_16_623035_623162	138	M/P/FB			213	P
GaLu96scf_16_675161_675288	51	M/P/FB			61	P
GaLu96scf_16_851595_851669	63	P			101	P
GaLu96scf_16_855760_855891	239	M/P/FB			184	P
GaLu96scf_16_856076_856128	184	P			117	P
GaLu96scf_16_856246_856320	117	P/FB			63	P
GaLu96scf_16_962229_962596	151	P			32	P
GaLu96scf_16_972866_972965	63	P			73	P
GaLu96scf_16_975346_975432	56	P/FB			79	P
GaLu96scf_17_302614_302761	48	M/P			46	P
GaLu96scf_17_309529_309719	193	M/P/FB			88	P
GaLu96scf_17_548716_549133	78	P			79	P
GaLu96scf_17_783119_783312	110	P			65	P
GaLu96scf_17_937387_937524	104	P/FB			81	P
GaLu96scf_18_226622_226820	91	M/P			54	P
GaLu96scf_18_250631_250865	79	P			74	P
GaLu96scf_18_382504_382575	564	M/P			101	P
GaLu96scf_18_447013_447496	89	M/P			69	P
GaLu96scf_18_567176_567332	83	M/P/FB			109	P
GaLu96scf_18_617342_617453	83	P/FB			25	P
GaLu96scf_18_621102_621236	89	M/P			83	P
GaLu96scf_18_621638_621667	236	M/P/FB			94	P
GaLu96scf_19_256680_256838	51	P			50	P
GaLu96scf_19_649165_649246	57	P/FB			83	P
GaLu96scf_19_696641_696956	61	P			44	P
GaLu96scf_19_737976_738132	191	P			138	P

GaLu96scf_19_800399_800513	95	P			64	P
GaLu96scf_19_819546_819631	187	M/P			100	P
GaLu96scf_2_1056816_1056943	61	M/P/FB			117	P
GaLu96scf_2_1096807_1097025	76	P			57	P
GaLu96scf_2_1186191_1186414	88	P			81	P
GaLu96scf_2_1238164_1238209	71	P			75	P
GaLu96scf_2_1297860_1298087	65	M/P/FB			52	P
GaLu96scf_2_1337037_1337195	59	P			56	P
GaLu96scf_2_1544548_1544760	234	M/P			223	P
GaLu96scf_2_176558_176637	57	P/FB			102	P
GaLu96scf_2_176740_176878	102	M/P/FB			64	P
GaLu96scf_2_1770102_1770284	56	P			138	P
GaLu96scf_2_1792380_1792590	61	P			63	P
GaLu96scf_2_2068219_2068354	54	M/P			57	P
GaLu96scf_2_2244381_2244456	54	P			51	P
GaLu96scf_2_286720_286882	90	P/FB			70	P
GaLu96scf_2_313829_313960	117	M/P			58	P
GaLu96scf_2_519187_519277	84	P			97	P
GaLu96scf_2_718009_718195	82	M/P/FB			64	P
GaLu96scf_2_733930_734001	58	P/FB			96	P
GaLu96scf_2_923644_923821	106	M/P/FB			55	P
GaLu96scf_2_979223_979340	199	P/FB			707	P
GaLu96scf_20_567409_567543	135	P			56	P
GaLu96scf_20_824149_824236	54	P			55	P
GaLu96scf_20_846169_846281	83	P			68	P
GaLu96scf_21_181527_181650	86	M/P/FB			63	P
GaLu96scf_21_294285_294404	746	P			52	P
GaLu96scf_21_35045_35648	322	M/P/FB			120	P
GaLu96scf_21_411672_411790	73	P			51	P
GaLu96scf_21_536672_536792	70	P			128	P
GaLu96scf_21_641342_641633	59	M/P			62	P
GaLu96scf_22_118127_118253	114	M/P/FB			71	P
GaLu96scf_22_184039_184144	113	P			43	P
GaLu96scf_22_318923_319037	49	P			58	P
GaLu96scf_22_779372_779568	69	P			81	P
GaLu96scf_22_780137_780230	72	M/P/FB			61	P
GaLu96scf_23_114407_114695	103	M/P/FB			53	P
GaLu96scf_23_197130_197327	51	P			58	P
GaLu96scf_23_280609_280689	106	P			96	P
GaLu96scf_23_307567_307705	127	P/FB			55	P
GaLu96scf_23_531474_531645	110	P/FB			205	P
GaLu96scf_23_615904_616085	162	M/P/FB			81	P
GaLu96scf_23_73825_73982	71	P			66	P
GaLu96scf_24_274858_275086	64	P/FB			70	P

GaLu96scf_24_43838_43938	221	P			95	P
GaLu96scf_24_87252_87525	63	P			62	P
GaLu96scf_25_426165_426404	116	M/P/FB			66	P
GaLu96scf_26_132391_132492	942	P			64	P
GaLu96scf_26_140017_140178	110	P/FB			243	P
GaLu96scf_26_148280_148389	78	M/P			91	P
GaLu96scf_26_483750_483995	74	P			56	P
GaLu96scf_26_547883_548062	56	P/FB			55	P
GaLu96scf_27_128048_128101	196	M/P/FB			69	P
GaLu96scf_27_264824_264934	115	P			50	P
GaLu96scf_27_564612_564678	120	P			67	P
GaLu96scf_27_564880_565132	82	P			58	P
GaLu96scf_28_279749_279870	88	P			64	P
GaLu96scf_28_363019_363157	62	P			57	P
GaLu96scf_28_39612_39738	65	P			230	P
GaLu96scf_29_121333_121455	66	P			53	P
GaLu96scf_29_36074_36198	205	P			128	P
GaLu96scf_29_94299_94585	53	P			66	P
GaLu96scf_3_1179570_1179652	98	M/P			94	P
GaLu96scf_3_1314243_1314421	53	P			64	P
GaLu96scf_3_1332003_1332095	120	M/P			54	P
GaLu96scf_3_1341812_1341929	58	P			54	P
GaLu96scf_3_1674497_1674580	84	M/P			74	P
GaLu96scf_3_217542_217804	65	P			68	P
GaLu96scf_3_220458_220723	84	P			52	P
GaLu96scf_3_223378_223567	61	P			65	P
GaLu96scf_3_495382_495427	58	P			50	P
GaLu96scf_3_495478_495561	50	M/P/FB			66	P
GaLu96scf_3_575832_575925	342	M/P/FB			48	P
GaLu96scf_3_637440_637683	77	P			80	P
GaLu96scf_3_711836_711957	149	M/P/FB			59	P
GaLu96scf_30_254564_254941	94	M/P			56	P
GaLu96scf_31_120722_120993	229	M/P/FB			243	P
GaLu96scf_31_201444_201975	178	M/P/FB			52	P
GaLu96scf_31_376798_376939	626	FB			52	P
GaLu96scf_31_435742_436432	447	P			73	P
GaLu96scf_31_88837_88918	58	P			100	P
GaLu96scf_32_29345_29463	148	M/P			89	P
GaLu96scf_32_331602_331804	55	P			58	P
GaLu96scf_33_107500_107539	47	M/P			415	P
GaLu96scf_33_192894_192993	111	P			133	P
GaLu96scf_33_91707_91997	75	M/P			64	P
GaLu96scf_34_188909_189098	165	P			54	P
GaLu96scf_34_336501_336644	139	M/P/FB			62	P

GaLu96scf_34_337457_337501	476	M/P/FB			46	P
GaLu96scf_34_340054_340182	59	P/FB			60	P
GaLu96scf_34_45294_45391	99	M/P/FB			80	P
GaLu96scf_35_279057_279177	98	P			119	P
GaLu96scf_35_350223_350354	59	M/P			52	P
GaLu96scf_35_67311_67438	79	P			53	P
GaLu96scf_37_118613_118683	90	P			56	P
GaLu96scf_37_224634_224689	523	P/FB			47	P
GaLu96scf_37_231727_231882	141	M/P/FB			57	P
GaLu96scf_4_1124355_1124494	216	P			183	P
GaLu96scf_4_1127979_1128023	162	M/P/FB			122	P
GaLu96scf_4_1128146_1128225	122	M/P/FB			93	P
GaLu96scf_4_138240_138389	107	M/P/FB			50	P
GaLu96scf_4_1459868_1460054	64	M/P/FB			73	P
GaLu96scf_4_1470480_1470545	57	M/P			63	P
GaLu96scf_4_1560573_1560669	64	P			62	P
GaLu96scf_4_168492_168552	354	M/P/FB			89	P
GaLu96scf_4_1879635_1879891	53	P			56	P
GaLu96scf_4_1884292_1884400	78	P			81	P
GaLu96scf_4_226425_226518	192	P			54	P
GaLu96scf_4_228229_228368	56	P			54	P
GaLu96scf_4_283196_283290	48	P			50	P
GaLu96scf_4_381502_381600	51	P			52	P
GaLu96scf_4_422602_422648	52	P			48	P
GaLu96scf_4_435074_435195	49	P			55	P
GaLu96scf_4_532307_532384	82	M/P/FB			62	P
GaLu96scf_4_56967_57162	47	P			57	P
GaLu96scf_4_692211_692300	57	P			51	P
GaLu96scf_4_832796_832984	87	P			90	P
GaLu96scf_4_858934_859059	175	M/P/FB			76	P
GaLu96scf_4_863734_863767	262	M/P			60	P
GaLu96scf_4_942105_942205	580	P/FB			71	P
GaLu96scf_40_3122_3208	55	P			60	P
GaLu96scf_41_110255_110290	242	P/FB			54	P
GaLu96scf_41_156524_156630	123	M/P/FB			383	P
GaLu96scf_42_65944_66179	66	P			59	P
GaLu96scf_45_158125_158408	35	P			65	P
GaLu96scf_46_57339_57436	176	M/P/FB			66	P
GaLu96scf_46_58490_58634	209	P			193	P
GaLu96scf_47_64056_64509	78	P/FB			57	P
GaLu96scf_47_89640_89756	139	M/P			85	P
GaLu96scf_48_75884_76107	73	P/FB			55	P
GaLu96scf_5_1045707_1045798	52	M/P			55	P
GaLu96scf_5_1064257_1064381	60	P			59	P

GaLu96scf_5_1144051_1144138	51	P			256	P
GaLu96scf_5_1382118_1382217	69	M/P/FB			73	P
GaLu96scf_5_1583766_1583884	49	P			35	P
GaLu96scf_5_1626995_1627023	61	P			59	P
GaLu96scf_5_174453_174788	69	P			137	P
GaLu96scf_5_288195_288260	69	M/P			105	P
GaLu96scf_5_36393_36580	129	M/P/FB			51	P
GaLu96scf_5_417693_417759	77	M/P/FB			86	P
GaLu96scf_5_419746_419828	107	P/FB			59	P
GaLu96scf_5_471914_472021	178	P			161	P
GaLu96scf_5_581823_582045	61	P			54	P
GaLu96scf_5_634361_634588	132	P			44	P
GaLu96scf_5_681237_681301	108	P/FB			168	P
GaLu96scf_5_683753_683915	130	M/P/FB			80	P
GaLu96scf_5_892014_892226	64	P			4	P
GaLu96scf_50_69505_69671	69	P			72	P
GaLu96scf_50_72806_72870	88	M/P			47	P
GaLu96scf_51_54110_54250	77	P			67	P
GaLu96scf_6_1077950_1078102	50	P			51	P
GaLu96scf_6_1268432_1268520	145	M/P			117	P
GaLu96scf_6_1277664_1277893	89	P			163	P
GaLu96scf_6_1278057_1278187	163	M/P/FB			96	P
GaLu96scf_6_306628_307022	63	P/FB			39	P
GaLu96scf_6_33894_33962	82	M/P/FB			58	P
GaLu96scf_6_392502_392638	51	P			54	P
GaLu96scf_6_55244_55351	58	P/FB			31	P
GaLu96scf_6_579734_579987	103	M/P/FB			136	P
GaLu96scf_6_597741_597848	41	P/FB			80	P
GaLu96scf_6_671717_671887	81	P			76	P
GaLu96scf_6_687443_687617	57	P			8	P
GaLu96scf_6_714661_714751	49	P			54	P
GaLu96scf_6_963910_964117	80	M/P/FB			54	P
GaLu96scf_7_1165988_1166233	71	P			53	P
GaLu96scf_7_1167567_1167656	83	P			121	P
GaLu96scf_7_206066_206203	306	M/P			145	P
GaLu96scf_7_210293_210385	98	M/P/FB			182	P
GaLu96scf_7_395510_395624	105	M/P			61	P
GaLu96scf_7_443074_443164	349	M/P/FB			58	P
GaLu96scf_7_596888_597021	57	P			10	P
GaLu96scf_7_644305_644561	68	P			60	P
GaLu96scf_7_724417_724526	46	P			47	P
GaLu96scf_7_776327_776575	63	P			58	P
GaLu96scf_7_847373_847473	123	M/P/FB			62	P
GaLu96scf_7_851851_851890	100	M/P			61	P

GaLu96scf_7_926139_926307	96	M/P/FB			61	P
GaLu96scf_8_1022209_1022412	1162	P			111	P
GaLu96scf_8_1080012_1080455	219	M/P			75	P
GaLu96scf_8_1159399_1159454	88	M/P/FB			66	P
GaLu96scf_8_1226682_1226928	108	P/FB			58	P
GaLu96scf_8_1269426_1269571	9	P			51	P
GaLu96scf_8_131438_131635	53	P			54	P
GaLu96scf_8_131690_131856	54	P			52	P
GaLu96scf_8_1530703_1531345	242	P			58	P
GaLu96scf_8_488801_488884	222	P/FB			66	P
GaLu96scf_8_731051_731101	411	M/P/FB			200	P
GaLu96scf_8_779487_779671	95	M/P			57	P
GaLu96scf_8_824225_824320	48	P			51	P
GaLu96scf_8_991639_991758	818	P			66	P
GaLu96scf_9_1048703_1048813	98	M/P/FB			70	P
GaLu96scf_9_1080098_1080159	107	P			126	P
GaLu96scf_9_1237193_1237373	292	P			52	P
GaLu96scf_9_1280373_1280512	59	M/P			57	P
GaLu96scf_9_1293365_1293520	79	P			87	P
GaLu96scf_9_1411797_1411916	52	P			52	P
GaLu96scf_9_1454807_1454987	54	P			63	P
GaLu96scf_9_150313_150446	88	M/P/FB			51	P
GaLu96scf_9_220011_220194	63	P			59	P
GaLu96scf_9_564822_564938	54	M/P/FB			54	P
GaLu96scf_9_672509_672830	112	M/P			64	P
GaLu96scf_9_729107_729376	480	P/FB			89	P
GaLu96scf_9_765949_766035	101	P			75	P
GaLu96scf_9_820471_820549	215	M/P/FB			52	P
GaLu96scf_9_841950_842063	57	P			58	P
GaLu96scf_9_864649_864764	283	M/P/FB			62	P
GaLu96scf_9_866454_866536	214	M/P/FB			52	P
GaLu96scf_9_981635_981779	360	M/P/FB			65	P

Table S4b Length of repetitive elements in the introns associated with exonics across the three developmental stages of *G. lucidum*.

Exonic ID	Length of repeat (bps) found in the introns (percentage of the intron length)		
	left intron	right intron	control intron
GaLu96scf_1_807086_807416	50 (34.97%)	32 (44.44%)	
GaLu96scf_11_101360_101610	37 (28.24%)	106 (37.32%)	
GaLu96scf_13_62664_63412	38 (40.43%)	33 (27.97%)	
GaLu96scf_2_1541899_1542100	109 (6.01%)	61 (3.98%)	
GaLu96scf_24_643346_643626	29 (9.27%)	31 (37.80%)	
GaLu96scf_26_559095_559469	472 (87.41%)	86 (9.11%)	
GaLu96scf_11_183914_184074	29 (21.64%)		39 (26.43%)
GaLu96scf_1_407026_407234		37 (23.12%)	37 (23.12%)
GaLu96scf_28_501725_502010		49 (47.57%)	49 (27.57%)
GaLu96scf_1_1319541_1319959	27 (34.62%)		
GaLu96scf_1_441144_441245	40 (37.04%)		
GaLu96scf_1_4641233_4641493	61 (6.11%)		
GaLu96scf_1_55000_55251	27 (27.84%)		
GaLu96scf_1_89438_89611	40 (21.98%)		
GaLu96scf_1_94170_94407	40 (10.93%)		
GaLu96scf_10_339814_340017	32 (47.06%)		
GaLu96scf_10_633173_633434	31 (44.29%)		
GaLu96scf_11_100041_100207	40 (20.41%)		
GaLu96scf_11_612651_612711	36 (42.35%)		
GaLu96scf_11_725031_725128	40 (8.40%)		
GaLu96scf_12_387721_387910	38 (38.78%)		
GaLu96scf_12_956568_957270	43 (31.85%)		
GaLu96scf_13_276282_276476	33 (34.02%)		
GaLu96scf_13_438876_439167	167 (69.58%)		
GaLu96scf_13_572959_573298	19 (24.05%)		
GaLu96scf_13_63531_64110	33 (27.97%)		
GaLu96scf_15_561186_561534	37 (26.24%)		
GaLu96scf_15_573883_573993	36 (40.00%)		
GaLu96scf_15_578462_578706	70 (45.45%)		
GaLu96scf_15_763952_764189	37 (43.53%)		
GaLu96scf_16_855760_855891	38 (15.90%)		
GaLu96scf_17_309529_309719	48 (24.87%)		
GaLu96scf_18_447013_447496	35 (39.33%)		
GaLu96scf_19_737976_738132	39 (20.42%)		

GaLu96scf_19_99310_99744	71 (47.02%)		
GaLu96scf_2_1463452_1463671	49 (29.52%)		
GaLu96scf_2_1543633_1544054	61 (3.98%)		
GaLu96scf_2_1770102_1770284	44 (78.57%)		
GaLu96scf_2_663567_663734	39 (9.47%)		
GaLu96scf_2_813532_814126	32 (22.70%)		
GaLu96scf_2_951265_951385	49 (43.75%)		
GaLu96scf_2_979223_979340	35 (17.59%)		
GaLu96scf_2_993116_993709	136 (41.98%)		
GaLu96scf_20_277215_277725	33 (28.70%)		
GaLu96scf_20_73587_73708	39 (19.31%)		
GaLu96scf_20_845487_845780	37 (19.68%)		
GaLu96scf_22_118127_118253	26 (22.81%)		
GaLu96scf_22_187637_187860	49 (29.70%)		
GaLu96scf_25_426165_426404	48 (41.38%)		
GaLu96scf_25_427065_427245	40 (20.51%)		
GaLu96scf_26_136975_137250	52 (57.53%)		
GaLu96scf_27_128806_128850	34 (12.78%)		
GaLu96scf_28_502114_502375	49 (47.57%)		
GaLu96scf_28_526348_526653	94 (60.26%)		
GaLu96scf_29_50957_51189	28 (11.81%)		
GaLu96scf_3_1236789_1237003	49 (11.78%)		
GaLu96scf_3_1674340_1674412	31 (12.97%)		
GaLu96scf_3_711836_711957	64 (42.95%)		
GaLu96scf_30_188966_189297	28 (30.11%)		
GaLu96scf_31_234899_235338	33 (22.15%)		
GaLu96scf_31_320750_320941	39 (39.39%)		
GaLu96scf_31_430223_430310	20 (9.62%)		
GaLu96scf_34_337457_337501	35 (7.35%)		
GaLu96scf_34_58949_59214	31 (28.70%)		
GaLu96scf_35_180898_181075	84 (46.93%)		
GaLu96scf_36_97718_98054	37 (33.64%)		
GaLu96scf_4_1124355_1124494	26 (12.04%)		
GaLu96scf_4_984554_984792	52 (52.53%)		
GaLu96scf_40_120652_120959	34 (47.22%)		
GaLu96scf_45_69519_69787	67 (63.21%)		
GaLu96scf_46_57339_57436	25 (14.20%)		
GaLu96scf_48_90717_90821	33 (62.26%)		
GaLu96scf_7_1167778_1168011	62 (51.24%)		
GaLu96scf_7_901352_901475	44 (16.30%)		
GaLu96scf_1_1293235_1293336		36 (7.27%)	
GaLu96scf_1_224262_224453		19 (6.17%)	
GaLu96scf_1_3850991_3851106		41 (13.53%)	
GaLu96scf_1_4023593_4023916		46 (29.30%)	

GaLu96scf_10_941606_941818		36 (37.50%)	
GaLu96scf_11_186062_186176		44 (36.67%)	
GaLu96scf_11_377445_377622		46 (20.09%)	
GaLu96scf_11_612424_612565		36 (42.35%)	
GaLu96scf_11_86974_87347		116 (89.92%)	
GaLu96scf_12_387357_387622		38 (38.78%)	
GaLu96scf_13_1077937_1078212		42 (12.03%)	
GaLu96scf_13_436641_436788		35 (9.09%)	
GaLu96scf_13_62415_62569		38 (40.43%)	
GaLu96scf_13_70956_71305		46 (31.94%)	
GaLu96scf_13_786277_786430		167 (93.82%)	
GaLu96scf_13_946765_947266		74 (74.00%)	
GaLu96scf_14_381022_381156		35 (57.38%)	
GaLu96scf_15_495959_496094		29 (28.43%)	
GaLu96scf_15_578105_578307		70 (45.45%)	
GaLu96scf_15_821433_822351		27 (4.05%)	
GaLu96scf_18_884641_884741		38 (6.87%)	
GaLu96scf_19_175956_176092		39 (26.90%)	
GaLu96scf_2_1177722_1177901		41 (18.98%)	
GaLu96scf_2_1545914_1545992		35 (23.65%)	
GaLu96scf_2_2292444_2292702		44 (28.95%)	
GaLu96scf_2_764152_764305		48 (9.18%)	
GaLu96scf_2_778974_779108		112 (9.37%)	
GaLu96scf_2_79752_80226		27 (25.71%)	
GaLu96scf_2_977772_977848		35 (23.81%)	
GaLu96scf_21_26613_26895		130 (50.00%)	
GaLu96scf_21_737969_738131		35 (36.08%)	
GaLu96scf_23_616167_616279		46 (49.46%)	
GaLu96scf_24_642832_643032		29 (9.27%)	
GaLu96scf_25_426753_426869		40 (20.51%)	
GaLu96scf_27_128469_128539		34 (12.78%)	
GaLu96scf_27_193935_194803		23 (27.38%)	
GaLu96scf_27_225254_225551		60 (27.03%)	
GaLu96scf_28_525829_526191		94 (60.26%)	
GaLu96scf_3_1117772_1118098		22 (11.17%)	
GaLu96scf_3_1675827_1676107		40 (11.90%)	
GaLu96scf_3_1899354_1899568		42 (18.67%)	
GaLu96scf_31_202028_202379		50 (30.30%)	
GaLu96scf_31_234356_234749		33 (22.15%)	
GaLu96scf_33_92540_92921		34 (17.62%)	
GaLu96scf_34_30502_30896		30 (31.58%)	
GaLu96scf_34_336802_336980		35 (7.35%)	
GaLu96scf_34_45472_45620		68 (51.91%)	
GaLu96scf_34_46040_46255		52 (10.22%)	

GaLu96scf_34_82313_82461		65 (45.45%)	
GaLu96scf_34_95773_96416		33 (42.31%)	
GaLu96scf_35_279297_279437		26 (19.55%)	
GaLu96scf_4_1128319_1128467		30 (17.54%)	
GaLu96scf_4_1884748_1884883		48 (33.10%)	
GaLu96scf_40_145087_145321		59 (43.38%)	
GaLu96scf_42_145252_145529		43 (61.43%)	
GaLu96scf_44_32765_33242		35 (19.34%)	
GaLu96scf_45_26854_27149		28 (24.56%)	
GaLu96scf_46_79582_80089		47 (20.52%)	
GaLu96scf_6_307062_307187		40 (47.62%)	
GaLu96scf_1_440858_441035			40 (37.04%)
GaLu96scf_1_440858_441430			40 (37.04%)
GaLu96scf_1_89101_89255			40 (21.98%)
GaLu96scf_1_89101_89611			40 (21.98%)
GaLu96scf_10_633036_633102			31 (24.29%)
GaLu96scf_10_633036_633434			31 (24.29%)
GaLu96scf_11_550698_551236			34 (17.53%)
GaLu96scf_11_612424_612944			36 (32.35%)
GaLu96scf_11_725031_725261			42 (17.53%)
GaLu96scf_13_438564_439167			167 (19.58%)
GaLu96scf_22_184039_184144			36 (33.72%)
GaLu96scf_23_535599_536009			24 (21.05%)
GaLu96scf_29_50957_51214			28 (11.81%)
GaLu96scf_34_45294_45391			34 (22.50%)
GaLu96scf_34_45294_45620			34 (32.50%)
GaLu96scf_7_1167567_1167656			62 (21.24%)

Table S4c Sequence details of repetitive elements in the introns associated with exonics across the three developmental stages of *G. lucidum*.

Exonic ID	Repeat elements ID	Start	End	Repeat sequence	Repeat class/family	Developmental Stages	Intron type
GaLu96scf_1_807086_807416	1	71	120	(CCTTTC)n	Simple_repeat	FB	left
GaLu96scf_11_101360_101610	1	86	122	(CACCCC)n	Simple_repeat	M/FB	left
GaLu96scf_13_62664_63412	1	39	76	(CCCCCA)n	Simple_repeat	M	left
GaLu96scf_2_1541899_1542100	1	39	71	(CAT)n	Simple_repeat	M/P/FB	left
GaLu96scf_2_1541899_1542100	2	1437	1457	(CTCG)n	Simple_repeat	M/P/FB	left
GaLu96scf_2_1541899_1542100	3	1696	1750	(CCATGCC)n	Simple_repeat	M/P/FB	left
GaLu96scf_24_643346_643626	1	59	87	(GT)n	Simple_repeat	M/P/FB	left
GaLu96scf_26_559095_559469	1	42	147	rnd-4_family-1990	Unknown	M/P/FB	left
GaLu96scf_26_559095_559469	2	142	465	rnd-4_family-322	Unknown	M/P/FB	left
GaLu96scf_26_559095_559469	3	470	517	rnd-4_family-1990	Unknown	M/P/FB	left
GaLu96scf_11_183914_184074	1	80	108	(TGGGAT)n	Simple_repeat	P	left
GaLu96scf_1_1319541_1319959	1	49	75	(TCTCC)n	Simple_repeat	FB	left
GaLu96scf_1_441144_441245	1	60	99	(AGGATG)n	Simple_repeat	M/P/FB	left
GaLu96scf_1_4641233_4641493	3	796	856	rnd-4_family-860	Unknown	M	left
GaLu96scf_1_55000_55251	1	6	32	(TCCCCC)n	Simple_repeat	M	left
GaLu96scf_1_89438_89611	1	137	176	(AGCGGAG)n	Simple_repeat	P/FB	left
GaLu96scf_1_94170_94407	1	321	360	(CCCCGAC)n	Simple_repeat	P/FB	left

GaLu96scf_10_339814_340017	1	4	35	(CGTCCC)n	Simple_repeat	P	left
GaLu96scf_10_633173_633434	1	6	36	(TC)n	Simple_repeat	M	left
GaLu96scf_11_100041_100207	1	17	56	(CCATTT)n	Simple_repeat	M/P/FB	left
GaLu96scf_11_612651_612711	1	46	81	G-rich	Low_complexity	M/P	left
GaLu96scf_11_725031_725128	1	108	147	rnd-4_family-1990	Unknown	M/FB	left
GaLu96scf_12_387721_387910	1	40	77	(GGAGGAT)n	Simple_repeat	M/P/FB	left
GaLu96scf_12_956568_957270	1	16	58	(GC)n	Simple_repeat	P	left
GaLu96scf_13_276282_276476	1	30	62	(CTCCC)n	Simple_repeat	M	left
GaLu96scf_13_438876_439167	1	8	174	(CTCCCTCT)n	Simple_repeat	M/P/FB	left
GaLu96scf_13_572959_573298	1	52	70	(AG)n	Simple_repeat	M	left
GaLu96scf_13_63531_64110	1	23	55	(CATC)n	Simple_repeat	M	left
GaLu96scf_15_561186_561534	1	97	133	(CCCTCCC)n	Simple_repeat	M	left
GaLu96scf_15_573883_573993	1	51	86	GA-rich	Low_complexity	M	left
GaLu96scf_15_578462_578706	1	12	81	(CCCTTGC)n	Simple_repeat	M	left
GaLu96scf_15_763952_764189	1	42	78	(TGCGTGA)n	Simple_repeat	P	left
GaLu96scf_16_855760_855891	1	195	232	GA-rich	Low_complexity	M/P/FB	left
GaLu96scf_17_309529_309719	1	123	170	GA-rich	Low_complexity	M/P/FB	left
GaLu96scf_18_447013_447496	1	24	58	(CAACCA)n	Simple_repeat	M/P	left
GaLu96scf_19_737976_738132	1	133	171	(CGCACC)n	Simple_repeat	P	left
GaLu96scf_19_99310_99744	1	51	121	G-rich	Low_complexity	M/P	left
GaLu96scf_2_1463452_1463671	1	29	77	(TCAGCCC)n	Simple_repeat	M/P/FB	left
GaLu96scf_2_1543633_1544054	1	35	61	(TTCGGG)n	Simple_repeat	M	left
GaLu96scf_2_1543633_1544054	2	66	99	(TCTGTG)n	Simple_repeat	M	left
GaLu96scf_2_1770102_1770284	1	2	45	(TGATGAA)n	Simple_repeat	P	left
GaLu96scf_2_663567_663734	1	362	400	(ACAAGCG)n	Simple_repeat	M/P/FB	left

GaLu96scf_2_813532_814126	1	44	75	(GTGGTT)n	Simple_repeat	P	left
GaLu96scf_2_951265_951385	1	56	104	GA-rich	Low_complexity	M	left
GaLu96scf_2_979223_979340	1	20	54	(GTGCGT)n	Simple_repeat	P/FB	left
GaLu96scf_2_993116_993709	1	18	76	(GCGCATC)n	Simple_repeat	M/P/FB	left
GaLu96scf_2_993116_993709	2	188	222	(GAACGGA)n	Simple_repeat	M/P/FB	left
GaLu96scf_2_993116_993709	3	267	308	(AACGCAG)n	Simple_repeat	M/P/FB	left
GaLu96scf_20_277215_277725	1	49	81	(TCCC)n	Simple_repeat	M	left
GaLu96scf_20_73587_73708	1	161	199	(TGTCT)n	Simple_repeat	M	left
GaLu96scf_20_845487_845780	1	141	177	A-rich	Low_complexity	P/FB	left
GaLu96scf_22_118127_118253	1	30	55	(ACCCG)n	Simple_repeat	M/P/FB	left
GaLu96scf_22_187637_187860	1	112	160	G-rich	Low_complexity	M/P/FB	left
GaLu96scf_25_426165_426404	1	32	79	G-rich	Low_complexity	M/P/FB	left
GaLu96scf_25_427065_427245	1	94	133	(TCTTTC)n	Simple_repeat	M	left
GaLu96scf_26_136975_137250	1	42	93	(CT)n	Simple_repeat	M/P/FB	left
GaLu96scf_27_128806_128850	1	70	103	(TGGGT)n	Simple_repeat	M/P/FB	left
GaLu96scf_28_502114_502375	1	15	63	(T)n	Simple_repeat	M	left
GaLu96scf_28_526348_526653	1	11	40	(CCCTT)n	Simple_repeat	M/P	left
GaLu96scf_28_526348_526653	2	86	118	(CTGATT)n	Simple_repeat	M/P	left
GaLu96scf_28_526348_526653	3	126	156	(ACGAAC)n	Simple_repeat	M/P	left
GaLu96scf_29_50957_51189	1	6	33	(CGTCG)n	Simple_repeat	M/P/FB	left
GaLu96scf_3_1236789_1237003	1	224	272	(CCTAAGC)n	Simple_repeat	FB	left
GaLu96scf_3_1674340_1674412	1	153	183	(GCCTA)n	Simple_repeat	M	left
GaLu96scf_3_711836_711957	1	66	129	GA-rich	Low_complexity	M/P/FB	left
GaLu96scf_30_188966_189297	1	29	56	(CA)n	Simple_repeat	M	left
GaLu96scf_31_234899_235338	1	6	38	(CCCT)n	Simple_repeat	M/P	left

GaLu96scf_31_320750_320941	1	12	50	(TCTGCT)n	Simple_repeat	M	left
GaLu96scf_31_430223_430310	1	124	143	(GTT)n	Simple_repeat	M/FB	left
GaLu96scf_34_337457_337501	1	75	109	G-rich	Low_complexity	M/P/FB	left
GaLu96scf_34_58949_59214	1	6	36	(TCCC)n	Simple_repeat	FB	left
GaLu96scf_35_180898_181075	1	45	109	GA-rich	Low_complexity	FB	left
GaLu96scf_35_180898_181075	2	89	128	(GAGGAC)n	Simple_repeat	FB	left
GaLu96scf_36_97718_98054	1	6	42	(CCCATTCC)n	Simple_repeat	M	left
GaLu96scf_4_1124355_1124494	1	185	210	(AAAG)n	Simple_repeat	P	left
GaLu96scf_4_984554_984792	1	7	58	(CCTCC)n	Simple_repeat	P	left
GaLu96scf_40_120652_120959	1	8	41	(TCCCA)n	Simple_repeat	P/FB	left
GaLu96scf_45_69519_69787	1	9	44	(CTCTTC)n	Simple_repeat	M	left
GaLu96scf_45_69519_69787	2	76	106	(ACTCGC)n	Simple_repeat	M	left
GaLu96scf_46_57339_57436	1	52	76	(CT)n	Simple_repeat	M/P/FB	left
GaLu96scf_48_90717_90821	1	16	48	G-rich	Low_complexity	M	left
GaLu96scf_7_1167778_1168011	1	47	108	(TCTCGC)n	Simple_repeat	M/P/FB	left
GaLu96scf_7_901352_901475	1	190	233	(GGGA)n	Simple_repeat	FB	left
GaLu96scf_1_807086_807416	1	6	37	(TGGTCA)n	Simple_repeat	FB	right
GaLu96scf_11_101360_101610	1	108	213	(CTTTCGT)n	Simple_repeat	M/FB	right
GaLu96scf_13_62664_63412	1	23	55	(CATC)n	Simple_repeat	M	right
GaLu96scf_2_1541899_1542100	1	35	61	(TTCGGG)n	Simple_repeat	M/P/FB	right
GaLu96scf_2_1541899_1542100	2	66	99	(TCTGTG)n	Simple_repeat	M/P/FB	right
GaLu96scf_24_643346_643626	1	46	76	(GGGAGG)n	Simple_repeat	M/P/FB	right
GaLu96scf_26_559095_559469	1	10	95	rnd-4_family-322	Unknown	M/P/FB	right
GaLu96scf_1_407026_407234	1	68	104	(AGGGGC)n	Simple_repeat	M/FB	right
GaLu96scf_28_501725_502010	1	15	63	(T)n	Simple_repeat	M	right

GaLu96scf_1_1293235_1293336	1	420	455	GA-rich	Low_complexity	M/P	right
GaLu96scf_1_224262_224453	1	143	161	(GGC)n	Simple_repeat	M/P/FB	right
GaLu96scf_1_3850991_3851106	1	42	82	(GATGGC)n	Simple_repeat	P	right
GaLu96scf_1_4023593_4023916	1	61	106	(GTGTC)n	Simple_repeat	M/FB	right
GaLu96scf_10_941606_941818	1	56	91	(AAGGG)n	Simple_repeat	M	right
GaLu96scf_11_186062_186176	1	27	70	(CTCTT)n	Simple_repeat	M/P/FB	right
GaLu96scf_11_377445_377622	1	22	67	(ATCCC)n	Simple_repeat	M	right
GaLu96scf_11_612424_612565	1	46	81	G-rich	Low_complexity	P	right
GaLu96scf_11_86974_87347	1	6	47	(TCCC)n	Simple_repeat	M	right
GaLu96scf_11_86974_87347	2	48	74	(TCT)n	Simple_repeat	M	right
GaLu96scf_11_86974_87347	3	75	121	(TCCC)n	Simple_repeat	M	right
GaLu96scf_12_387357_387622	1	40	77	(GGAGGAT)n	Simple_repeat	M	right
GaLu96scf_13_1077937_1078212	1	180	221	G-rich	Low_complexity	M	right
GaLu96scf_13_436641_436788	1	12	46	(TTGCGTC)n	Simple_repeat	M/P/FB	right
GaLu96scf_13_62415_62569	1	39	76	(CCCCCA)n	Simple_repeat	M	right
GaLu96scf_13_70956_71305	1	38	83	(TTTCCGT)n	Simple_repeat	M	right
GaLu96scf_13_786277_786430	1	4	170	GA-rich	Low_complexity	M	right
GaLu96scf_13_946765_947266	1	20	30	G-rich	Low_complexity	M/P	right
GaLu96scf_13_946765_947266	2	31	93	GA-rich	Low_complexity	M/P	right
GaLu96scf_14_381022_381156	1	12	46	(TCCT)n	Simple_repeat	P/FB	right
GaLu96scf_15_495959_496094	1	23	51	(CACCA)n	Simple_repeat	FB	right
GaLu96scf_15_578105_578307	1	12	81	(CCCTTGC)n	Simple_repeat	M/P/FB	right
GaLu96scf_15_821433_822351	1	609	635	(AACGC)n	Simple_repeat	M/P/FB	right
GaLu96scf_18_884641_884741	1	490	527	(CGCAGA)n	Simple_repeat	M	right
GaLu96scf_19_175956_176092	1	78	116	(CACTCTT)n	Simple_repeat	M/FB	right

GaLu96scf_2_1177722_1177901	1	46	86	G-rich	Low_complexity	M	right
GaLu96scf_2_1545914_1545992	1	5	39	(GCTTC)n	Simple_repeat	M/P	right
GaLu96scf_2_2292444_2292702	1	17	60	(CCTTT)n	Simple_repeat	M	right
GaLu96scf_2_764152_764305	1	450	497	(CGGAAGA)n	Simple_repeat	M	right
GaLu96scf_2_778974_779108	1	158	201	(GCGATCT)n	Simple_repeat	M/P/FB	right
GaLu96scf_2_778974_779108	2	300	367	(CCGTTCT)n	Simple_repeat	M/P/FB	right
GaLu96scf_2_79752_80226	1	71	97	(C)n	Simple_repeat	M	right
GaLu96scf_2_977772_977848	1	35	69	(TTCC)n	Simple_repeat	FB	right
GaLu96scf_21_26613_26895	1	41	170	rnd-4_family-1710	Unknown	M	right
GaLu96scf_21_737969_738131	1	6	40	(TGTTCC)n	Simple_repeat	M/FB	right
GaLu96scf_23_616167_616279	1	3	48	(GAGTCC)n	Simple_repeat	M/P/FB	right
GaLu96scf_24_642832_643032	1	59	87	(GT)n	Simple_repeat	M	right
GaLu96scf_25_426753_426869	1	94	133	(TCTTTC)n	Simple_repeat	M/P	right
GaLu96scf_27_128469_128539	1	70	103	(TGGGT)n	Simple_repeat	M/P/FB	right
GaLu96scf_27_193935_194803	1	35	57	(G)n	Simple_repeat	M	right
GaLu96scf_27_225254_225551	1	40	99	rnd-4_family-1704	Unknown	M	right
GaLu96scf_28_525829_526191	1	11	40	(CCCTT)n	Simple_repeat	M/FB	right
GaLu96scf_28_525829_526191	2	86	118	(CTGATT)n	Simple_repeat	M/FB	right
GaLu96scf_28_525829_526191	3	126	156	(ACGAAC)n	Simple_repeat	M/FB	right
GaLu96scf_3_1117772_1118098	1	7	28	(CTTG)n	Simple_repeat	P/FB	right
GaLu96scf_3_1675827_1676107	1	104	143	(CCCTC)n	Simple_repeat	M/P/FB	right
GaLu96scf_3_1899354_1899568	1	135	176	(CTCGCG)n	Simple_repeat	M/FB	right
GaLu96scf_31_202028_202379	1	65	114	(AATG)n	Simple_repeat	M/P/FB	right
GaLu96scf_31_234356_234749	1	6	38	(CCCT)n	Simple_repeat	M	right
GaLu96scf_33_92540_92921	1	32	65	GA-rich	Low_complexity	M/P	right

GaLu96scf_34_30502_30896	1	9	38	(GCCA)n	Simple_repeat	M	right
GaLu96scf_34_336802_336980	1	75	109	G-rich	Low_complexity	M/P/FB	right
GaLu96scf_34_45472_45620	1	2	27	(TGCA)n	Simple_repeat	M/P/FB	right
GaLu96scf_34_45472_45620	2	41	82	(CAAGT)n	Simple_repeat	M/P/FB	right
GaLu96scf_34_46040_46255	1	35	86	G-rich	Low_complexity	P	right
GaLu96scf_34_82313_82461	1	10	50	(CTCCCC)n	Simple_repeat	M	right
GaLu96scf_34_82313_82461	2	111	134	(C)n	Simple_repeat	M	right
GaLu96scf_34_95773_96416	1	7	39	(GCTCTC)n	Simple_repeat	M/P	right
GaLu96scf_35_279297_279437	1	104	129	(AGGG)n	Simple_repeat	P	right
GaLu96scf_4_1128319_1128467	1	136	165	(ACTCGG)n	Simple_repeat	M/P/FB	right
GaLu96scf_4_1884748_1884883	1	59	106	(TGTGGCT)n	Simple_repeat	P	right
GaLu96scf_40_145087_145321	1	6	64	G-rich	Low_complexity	M/FB	right
GaLu96scf_42_145252_145529	1	19	61	(TAGGG)n	Simple_repeat	M/P/FB	right
GaLu96scf_44_32765_33242	1	124	158	A-rich	Low_complexity	FB	right
GaLu96scf_45_26854_27149	1	41	68	(GGTTGT)n	Simple_repeat	P	right
GaLu96scf_46_79582_80089	1	6	52	(TTTCGTC)n	Simple_repeat	M/FB	right
GaLu96scf_6_307062_307187	1	6	45	(CT)n	Simple_repeat	P/FB	right
GaLu96scf_1_440858_441035	1	60	99	(AGGATG)n	Simple_repeat	M/P/FB	control
GaLu96scf_1_440858_441430	1	60	99	(AGGATG)n	Simple_repeat	M/P/FB	control
GaLu96scf_1_89101_89255	1	137	176	(AGCGGAG)n	Simple_repeat	M/P/FB	control
GaLu96scf_1_89101_89611	1	137	176	(AGCGGAG)n	Simple_repeat	M/P/FB	control
GaLu96scf_10_633036_633102	1	6	36	(TC)n	Simple_repeat	M/P/FB	control
GaLu96scf_10_633036_633434	1	6	36	(TC)n	Simple_repeat	M/P/FB	control
GaLu96scf_11_550698_551236	1	33	66	(CGCTCT)n	Simple_repeat	M	control
GaLu96scf_11_612424_612944	1	46	81	G-rich	Low_complexity	M/FB	control

GaLu96scf_11_725031_725261	1	8	49	(CGA)n	Simple_repeat	M/FB	control
GaLu96scf_13_438564_439167	1	8	174	(CTCCCTCT)n	Simple_repeat	M/FB	control
GaLu96scf_22_184039_184144	1	7	42	(AGCGACA)n	Simple_repeat	P	control
GaLu96scf_23_535599_536009	1	44	67	(GAAG)n	Simple_repeat	M/FB	control
GaLu96scf_29_50957_51214	1	6	33	(CGTCG)n	Simple_repeat	M	control
GaLu96scf_34_45294_45391	1	43	76	G-rich	Low_complexity	M/P/FB	control
GaLu96scf_34_45294_45620	1	43	76	G-rich	Low_complexity	M/P/FB	control
GaLu96scf_7_1167567_1167656	1	47	108	(TCTCGC)n	Simple_repeat	P	control
GaLu96scf_11_183914_184074	1	1	39	(GTGC)n	Simple_repeat	P	control
GaLu96scf_1_407026_407234	1	68	104	(AGGGGC)n	Simple_repeat	P	control
GaLu96scf_28_501725_502010	1	15	63	(T)n	Simple_repeat	P	control

Table S5 Short inverted repeats sequences in the introns flanking the exonics in *G. lucidum*.

Exonic ID	Score	Matches	Gaps	Short inverted repeat sequences	Developmental stages
GaLu96scf26_559095_559469	55	33/44 (75%)	0	430 gaagcgactcagaatcgttaactttgactacccctattatatat 473<br>||| || |||| ||| | | ||| |||| || |||||| || || 615 cttagcggagttttaacgaatgagactggtgaggataaaattta 572	Mycelia<br>Primordia<br>Fruiting Bodies
GaLu96scf25_426165_426869	58	22/24 (91%)	0	44 ggggggggagggagagggaggaga 67<br>|||||||||||||| ||| ||||| 323 cccccccctccctcccccgcctct 300	Primordia

Table S6 Primers used for the validation of exonic circRNAs (Table S6.xlsx).

Table S7 Enrichment analysis for the parental genes of exonics based on GO terms. Here total number of genes is 16127. Total number of parent genes is 561.

GO ID	GO Annotation	Number of Genes Mapped to This Category	Number of Parent Gene Mapped to this Category	P value	Q value (FDR)	Significance level
GO:0055114	oxidation reduction	618	50	0.0000	0.0000	q<0.01
GO:0016020	membrane	312	32	0.0000	0.0000	q<0.01
GO:0003824	catalytic activity	791	58	0.0000	0.0000	q<0.01
GO:0005622	intracellular	381	36	0.0000	0.0000	q<0.01
GO:0005525	GTP binding	97	16	0.0000	0.0000	q<0.01
GO:0006412	translation	149	20	0.0000	0.0000	q<0.01
GO:0003735	structural constituent of ribosome	107	16	0.0000	0.0000	q<0.01
GO:0055085	transmembrane transport	340	30	0.0000	0.0001	q<0.01
GO:0008483	transaminase activity	8	5	0.0000	0.0001	q<0.01
GO:0005840	ribosome	105	15	0.0000	0.0001	q<0.01
GO:0015031	protein transport	57	10	0.0000	0.0006	q<0.01
GO:0000166	nucleotide binding	214	20	0.0000	0.0011	q<0.01
GO:0016491	oxidoreductase activity	482	34	0.0000	0.0011	q<0.01
GO:0003924	GTPase activity	53	9	0.0001	0.0016	q<0.01
GO:0006810	transport	174	17	0.0001	0.0019	q<0.01

GO:0030170	pyridoxal phosphate binding	56	9	0.0001	0.0022	q<0.01
GO:0006457	protein folding	57	9	0.0001	0.0023	q<0.01
GO:0016021	integral to membrane	251	21	0.0001	0.0023	q<0.01
GO:0019001	guanyl nucleotide binding	16	5	0.0001	0.0026	q<0.01
GO:0020037	heme binding	257	21	0.0002	0.0028	q<0.01
GO:0005506	iron ion binding	260	21	0.0002	0.0031	q<0.01
GO:0006350	transcription	76	10	0.0002	0.0035	q<0.01
GO:0005737	cytoplasm	138	14	0.0002	0.0034	q<0.01
GO:0008152	metabolic process	683	41	0.0002	0.0033	q<0.01
GO:0009055	electron carrier activity	250	20	0.0003	0.0041	q<0.01
GO:0005215	transporter activity	65	9	0.0003	0.0041	q<0.01
GO:0005488	binding	476	31	0.0003	0.0041	q<0.01
GO:0004871	signal transducer activity	29	6	0.0004	0.0044	q<0.01
GO:0007165	signal transduction	67	9	0.0004	0.0046	q<0.01
GO:0016616	oxidoreductase activity, acting on the CH-OH group of donors, NAD or NADP as acceptor	20	5	0.0005	0.0051	q<0.01
GO:0007264	small GTPase mediated signal transduction	42	7	0.0005	0.0051	q<0.01
GO:0015986	ATP synthesis coupled proton transport	22	5	0.0007	0.0076	q<0.01
GO:0005509	calcium ion binding	34	6	0.0009	0.0088	q<0.01
GO:0007186	G-protein coupled receptor protein signaling pathway	23	5	0.0009	0.0088	q<0.01
GO:0051082	unfolded protein binding	35	6	0.0010	0.0097	q<0.01
GO:0009987	cellular process	14	4	0.0010	0.0095	q<0.01
GO:0004497	monooxygenase activity	258	19	0.0010	0.0095	q<0.01
GO:0004470	malic enzyme activity	2	2	0.0012	0.0106	0.01<q<0.05

GO:0004768	stearoyl-CoA 9-desaturase activity	2	2	0.0012	0.0106	0.01<q<0.05
GO:0046872	metal ion binding	37	6	0.0014	0.0113	0.01<q<0.05
GO:0016192	vesicle-mediated transport	38	6	0.0016	0.0127	0.01<q<0.05
GO:0005524	ATP binding	644	36	0.0016	0.0125	0.01<q<0.05
GO:0004674	protein serine/threonine kinase activity	173	14	0.0019	0.0146	0.01<q<0.05
GO:0030145	manganese ion binding	8	3	0.0020	0.0150	0.01<q<0.05
GO:0003677	DNA binding	319	21	0.0022	0.0161	0.01<q<0.05
GO:0051287	NAD or NADH binding	28	5	0.0022	0.0160	0.01<q<0.05
GO:0005975	carbohydrate metabolic process	220	16	0.0026	0.0187	0.01<q<0.05
GO:0043169	cation binding	91	9	0.0031	0.0218	0.01<q<0.05
GO:0006355	regulation of transcription, DNA-dependent	127	11	0.0032	0.0219	0.01<q<0.05
GO:0006108	malate metabolic process	3	2	0.0035	0.0234	0.01<q<0.05
GO:0015078	hydrogen ion transmembrane transporter activity	10	3	0.0039	0.0258	0.01<q<0.05
GO:0015935	small ribosomal subunit	10	3	0.0039	0.0258	0.01<q<0.05
GO:0019787	small conjugating protein ligase activity	20	4	0.0040	0.0253	0.01<q<0.05
GO:0043687	post-translational protein modification	20	4	0.0040	0.0253	0.01<q<0.05
GO:0051246	regulation of protein metabolic process	20	4	0.0040	0.0253	0.01<q<0.05
GO:0005634	nucleus	345	21	0.0048	0.0285	0.01<q<0.05
GO:0006633	fatty acid biosynthetic process	11	3	0.0052	0.0306	0.01<q<0.05
GO:0006334	nucleosome assembly	22	4	0.0056	0.0325	0.01<q<0.05
GO:0022891	substrate-specific transmembrane transporter activity	22	4	0.0056	0.0325	0.01<q<0.05
GO:0004672	protein kinase activity	202	14	0.0064	0.0360	0.01<q<0.05
GO:0005743	mitochondrial inner membrane	23	4	0.0066	0.0361	0.01<q<0.05
GO:0000276	mitochondrial proton-transporting ATP synthase	4	2	0.0068	0.0365	0.01<q<0.05

	complex, coupling factor F(o)					
GO:0016787	hydrolase activity	123	10	0.0070	0.0371	0.01<q<0.05
GO:0006913	nucleocytoplasmic transport	13	3	0.0084	0.0441	0.01<q<0.05
GO:0050660	FAD binding	111	9	0.0100	0.0516	no
GO:0004519	endonuclease activity	14	3	0.0103	0.0525	no
GO:0006164	purine nucleotide biosynthetic process	5	2	0.0109	0.0543	no
GO:0005783	endoplasmic reticulum	15	3	0.0125	0.0615	no
GO:0003779	actin binding	16	3	0.0148	0.0720	no
GO:0006904	vesicle docking during exocytosis	6	2	0.0157	0.0753	no
GO:0006468	protein amino acid phosphorylation	240	14	0.0205	0.0967	no
GO:0016881	acid-amino acid ligase activity	7	2	0.0213	0.0989	no
GO:0016773	phosphotransferase activity, alcohol group as acceptor	19	3	0.0231	0.1060	no
GO:0000786	nucleosome	20	3	0.0262	0.1187	no
GO:0016769	transferase activity, transferring nitrogenous groups	20	3	0.0262	0.1187	no
GO:0005216	ion channel activity	8	2	0.0274	0.1206	no
GO:0030163	protein catabolic process	8	2	0.0274	0.1206	no
GO:0004185	serine-type carboxypeptidase activity	21	3	0.0295	0.1269	no
GO:0006508	proteolysis	232	13	0.0298	0.1264	no
GO:0010181	FMN binding	37	4	0.0300	0.1255	no
GO:0005515	protein binding	363	18	0.0332	0.1374	no
GO:0009058	biosynthetic process	57	5	0.0337	0.1377	no
GO:0046961	proton-transporting ATPase activity, rotational mechanism	9	2	0.0340	0.1371	no
GO:0003937	IMP cyclohydrolase activity	1	1	0.0348	0.1387	no

GO:0004019	adenylosuccinate synthase activity	1	1	0.0348	0.1387	no
GO:0004043	L-aminoadipate-semialdehyde dehydrogenase activity	1	1	0.0348	0.1387	no
GO:0004076	biotin synthase activity	1	1	0.0348	0.1387	no
GO:0004357	glutamate-cysteine ligase activity	1	1	0.0348	0.1387	no
GO:0004402	histone acetyltransferase activity	1	1	0.0348	0.1387	no
GO:0004420	hydroxymethylglutaryl-CoA reductase (NADPH) activity	1	1	0.0348	0.1387	no
GO:0004450	isocitrate dehydrogenase (NADP+) activity	1	1	0.0348	0.1387	no
GO:0004474	malate synthase activity	1	1	0.0348	0.1387	no
GO:0004602	glutathione peroxidase activity	1	1	0.0348	0.1387	no
GO:0004611	phosphoenolpyruvate carboxykinase activity	1	1	0.0348	0.1387	no
GO:0004612	phosphoenolpyruvate carboxykinase (ATP) activity	1	1	0.0348	0.1387	no
GO:0004619	phosphoglycerate mutase activity	1	1	0.0348	0.1387	no
GO:0004643	phosphoribosylaminoimidazolecarboxamide formyltransferase activity	1	1	0.0348	0.1387	no
GO:0004651	polynucleotide 5'-phosphatase activity	1	1	0.0348	0.1387	no
GO:0004801	transaldolase activity	1	1	0.0348	0.1387	no
GO:0004829	threonine-tRNA ligase activity	1	1	0.0348	0.1387	no
GO:0004832	valine-tRNA ligase activity	1	1	0.0348	0.1387	no
GO:0004853	uroporphyrinogen decarboxylase activity	1	1	0.0348	0.1387	no
GO:0005094	Rho GDP-dissociation inhibitor activity	1	1	0.0348	0.1387	no
GO:0005665	DNA-directed RNA polymerase II, core complex	1	1	0.0348	0.1387	no
GO:0006007	glucose catabolic process	1	1	0.0348	0.1387	no
GO:0006097	glyoxylate cycle	1	1	0.0348	0.1387	no

GO:0006102	isocitrate metabolic process	1	1	0.0348	0.1387	no
GO:0006188	IMP biosynthetic process	1	1	0.0348	0.1387	no
GO:0006435	threonyl-tRNA aminoacylation	1	1	0.0348	0.1387	no
GO:0006438	valyl-tRNA aminoacylation	1	1	0.0348	0.1387	no
GO:0006452	translational frameshifting	1	1	0.0348	0.1387	no
GO:0006621	protein retention in ER lumen	1	1	0.0348	0.1387	no
GO:0007585	respiratory gaseous exchange	1	1	0.0348	0.1387	no
GO:0008250	Oligo-saccharyl transferase complex	1	1	0.0348	0.1387	no
GO:0008410	CoA-transferase activity	1	1	0.0348	0.1387	no
GO:0009102	biotin biosynthetic process	1	1	0.0348	0.1387	no
GO:0015936	coenzyme A metabolic process	1	1	0.0348	0.1387	no
GO:0016246	RNA interference	1	1	0.0348	0.1387	no
GO:0016442	RNA-induced silencing complex	1	1	0.0348	0.1387	no
GO:0016847	1-aminocyclopropane-1-carboxylate synthase activity	1	1	0.0348	0.1387	no
GO:0017076	purine nucleotide binding	1	1	0.0348	0.1387	no
GO:0017136	NAD-dependent histone deacetylase activity	1	1	0.0348	0.1387	no
GO:0019478	D-amino acid catabolic process	1	1	0.0348	0.1387	no
GO:0030272	5-formyltetrahydrofolate cyclo-ligase activity	1	1	0.0348	0.1387	no
GO:0031533	mRNA cap methyltransferase complex	1	1	0.0348	0.1387	no
GO:0042578	phosphoric ester hydrolase activity	1	1	0.0348	0.1387	no
GO:0045901	positive regulation of translational elongation	1	1	0.0348	0.1387	no
GO:0045905	positive regulation of translational termination	1	1	0.0348	0.1387	no
GO:0046923	ER retention sequence binding	1	1	0.0348	0.1387	no
GO:0048193	Golgi vesicle transport	1	1	0.0348	0.1387	no

GO:0051186	cofactor metabolic process	1	1	0.0348	0.1387	no
GO:0016810	hydrolase activity, acting on carbon-nitrogen (but not peptide) bonds	24	3	0.0404	0.1026	no
GO:0003700	transcription factor activity	105	7	0.0435	0.1095	no
GO:0006066	alcohol metabolic process	25	3	0.0444	0.1109	no
GO:0003684	damaged DNA binding	11	2	0.0484	0.1200	no
GO:0003899	DNA-directed RNA polymerase activity	26	3	0.0484	0.1192	no
GO:0016614	oxidoreductase activity, acting on CH-OH group of donors	45	4	0.0510	0.1247	no
GO:0006281	DNA repair	46	4	0.0539	0.1309	no
GO:0008270	zinc ion binding	598	25	0.0541	0.1303	no
GO:0006886	intracellular protein transport	67	5	0.0547	0.1308	no
GO:0046873	metal ion transmembrane transporter activity	12	2	0.0560	0.1331	no
GO:0051539	4 iron, 4 sulfur cluster binding	12	2	0.0560	0.1331	no
GO:0031072	heat shock protein binding	29	3	0.0612	0.1434	no
GO:0004175	endopeptidase activity	13	2	0.0639	0.1487	no
GO:0004177	aminopeptidase activity	13	2	0.0639	0.1487	no
GO:0004518	nuclease activity	13	2	0.0639	0.1487	no
GO:0004222	metalloendopeptidase activity	30	3	0.0656	0.1496	no
GO:0003951	NAD+ kinase activity	2	1	0.0672	0.1520	no
GO:0004066	asparagine synthase (glutamine-hydrolyzing) activity	2	1	0.0672	0.1520	no
GO:0004084	branched-chain-amino-acid transaminase activity	2	1	0.0672	0.1520	no
GO:0004348	glucosylceramidase activity	2	1	0.0672	0.1520	no
GO:0004615	phosphomannomutase activity	2	1	0.0672	0.1520	no

GO:0004816	asparagine-tRNA ligase activity	2	1	0.0672	0.1520	no
GO:0005764	lysosome	2	1	0.0672	0.1520	no
GO:0006421	asparaginyl-tRNA aminoacylation	2	1	0.0672	0.1520	no
GO:0006525	arginine metabolic process	2	1	0.0672	0.1520	no
GO:0006529	asparagine biosynthetic process	2	1	0.0672	0.1520	no
GO:0006535	cysteine biosynthetic process from serine	2	1	0.0672	0.1520	no
GO:0006750	glutathione biosynthetic process	2	1	0.0672	0.1520	no
GO:0007040	lysosome organization	2	1	0.0672	0.1520	no
GO:0008612	peptidyl-lysine modification to hypusine	2	1	0.0672	0.1520	no
GO:0009081	branched chain family amino acid metabolic process	2	1	0.0672	0.1520	no
GO:0009085	lysine biosynthetic process	2	1	0.0672	0.1520	no
GO:0016841	ammonia-lyase activity	2	1	0.0672	0.1520	no
GO:0018279	protein amino acid N-linked glycosylation via asparagine	2	1	0.0672	0.1520	no
GO:0019307	mannose biosynthetic process	2	1	0.0672	0.1520	no
GO:0030288	outer membrane-bounded periplasmic space	2	1	0.0672	0.1520	no
GO:0045263	proton-transporting ATP synthase complex, coupling factor F(o)	2	1	0.0672	0.1520	no
GO:0006629	lipid metabolic process	51	4	0.0692	0.1372	no
GO:0006418	tRNA aminoacylation for protein translation	31	3	0.0702	0.1382	no
GO:0003676	nucleic acid binding	461	19	0.0718	0.1406	no
GO:0003755	peptidyl-prolyl cis-trans isomerase activity	14	2	0.0720	0.1402	no
GO:0004298	threonine-type endopeptidase activity	14	2	0.0720	0.1402	no
GO:0005839	proteasome core complex	14	2	0.0720	0.1402	no

GO:0006807	nitrogen compound metabolic process	14	2	0.0720	0.1402	no
GO:0051603	proteolysis involved in cellular protein catabolic process	14	2	0.0720	0.1402	no
GO:0004812	aminoacyl-tRNA ligase activity	32	3	0.0747	0.1414	no
GO:0030001	metal ion transport	15	2	0.0802	0.1509	no
GO:0051536	iron-sulfur cluster binding	16	2	0.0884	0.1655	no
GO:0004386	helicase activity	84	5	0.0959	0.1786	no
GO:0006464	protein modification process	17	2	0.0968	0.1791	no
GO:0016884	carbon-nitrogen ligase activity, with glutamine as amido-N-donor	17	2	0.0968	0.1791	no
GO:0000030	mannosyltransferase activity	3	1	0.0972	0.1780	no
GO:0003918	DNA topoisomerase (ATP-hydrolyzing) activity	3	1	0.0972	0.1780	no
GO:0004356	glutamate-ammonia ligase activity	3	1	0.0972	0.1780	no
GO:0004407	histone deacetylase activity	3	1	0.0972	0.1780	no
GO:0004579	dolichyl-diphosphooligosaccharide-protein glycotransferase activity	3	1	0.0972	0.1780	no
GO:0005247	voltage-gated chloride channel activity	3	1	0.0972	0.1780	no
GO:0006366	transcription from RNA polymerase II promoter	3	1	0.0972	0.1780	no
GO:0006370	mRNA capping	3	1	0.0972	0.1780	no
GO:0006465	signal peptide processing	3	1	0.0972	0.1780	no
GO:0006493	protein amino acid O-linked glycosylation	3	1	0.0972	0.1780	no
GO:0006559	L-phenylalanine catabolic process	3	1	0.0972	0.1780	no
GO:0006665	sphingolipid metabolic process	3	1	0.0972	0.1780	no
GO:0006813	potassium ion transport	3	1	0.0972	0.1780	no
GO:0006915	apoptosis	3	1	0.0972	0.1780	no

GO:0015450	P-P-bond-hydrolysis-driven protein transmembrane transporter activity	3	1	0.0972	0.1780	no
GO:0016568	chromatin modification	3	1	0.0972	0.1780	no
GO:0016575	histone deacetylation	3	1	0.0972	0.1780	no
GO:0016705	oxidoreductase activity, acting on paired donors, with incorporation or reduction of molecular oxygen	3	1	0.0972	0.1780	no
GO:0016717	oxidoreductase activity, acting on paired donors, with oxidation of a pair of donors resulting in the reduction of molecular oxygen to two molecules of water	3	1	0.0972	0.1780	no
GO:0016813	hydrolase activity, acting on carbon-nitrogen (but not peptide) bonds, in linear amidines	3	1	0.0972	0.1780	no
GO:0031177	phosphopantetheine binding	3	1	0.0972	0.1780	no
GO:0033179	proton-transporting V-type ATPase, V0 domain	3	1	0.0972	0.1780	no
GO:0043022	ribosome binding	3	1	0.0972	0.1780	no
GO:0008026	ATP-dependent helicase activity	64	4	0.1113	0.1809	no
GO:0006979	response to oxidative stress	19	2	0.1134	0.1835	no
GO:0004553	hydrolase activity, hydrolyzing O-glycosyl compounds	151	7	0.1165	0.1876	no
GO:0000287	magnesium ion binding	41	3	0.1169	0.1874	no
GO:0006520	cellular amino acid metabolic process	20	2	0.1216	0.1940	no
GO:0045454	cell redox homeostasis	20	2	0.1216	0.1940	no
GO:0050661	NADP or NADPH binding	20	2	0.1216	0.1940	no
GO:0017111	nucleoside-triphosphatase activity	96	5	0.1244	0.1956	no
GO:0003849	3-deoxy-7-phosphoheptulonate synthase activity	4	1	0.1251	0.1959	no
GO:0004089	carbonate dehydratase activity	4	1	0.1251	0.1959	no

GO:0004784	superoxide dismutase activity	4	1	0.1251	0.1959	no
GO:0005740	mitochondrial envelope	4	1	0.1251	0.1959	no
GO:0006094	gluconeogenesis	4	1	0.1251	0.1959	no
GO:0006098	pentose-phosphate shunt	4	1	0.1251	0.1959	no
GO:0006821	chloride transport	4	1	0.1251	0.1959	no
GO:0008121	ubiquinol-cytochrome-c reductase activity	4	1	0.1251	0.1959	no
GO:0015976	carbon utilization	4	1	0.1251	0.1959	no
GO:0016624	oxidoreductase activity, acting on the aldehyde or oxo group of donors, disulfide as acceptor	4	1	0.1251	0.1959	no
GO:0019318	hexose metabolic process	4	1	0.1251	0.1959	no
GO:0030833	regulation of actin filament polymerization	4	1	0.1251	0.1959	no
GO:0031105	septin complex	4	1	0.1251	0.1959	no
GO:0046907	intracellular transport	4	1	0.1251	0.1959	no
GO:0004190	aspartic-type endopeptidase activity	69	4	0.1269	0.1864	no
GO:0003723	RNA binding	104	5	0.1411	0.2064	no
GO:0006865	amino acid transport	23	2	0.1456	0.2121	no
GO:0015171	amino acid transmembrane transporter activity	23	2	0.1456	0.2121	no
GO:0016788	hydrolase activity, acting on ester bonds	23	2	0.1456	0.2121	no
GO:0016791	phosphatase activity	23	2	0.1456	0.2121	no
GO:0004721	phosphoprotein phosphatase activity	5	1	0.1510	0.2162	no
GO:0004815	aspartate-tRNA ligase activity	5	1	0.1510	0.2162	no
GO:0006422	aspartyl-tRNA aminoacylation	5	1	0.1510	0.2162	no
GO:0006801	superoxide metabolic process	5	1	0.1510	0.2162	no
GO:0006811	ion transport	5	1	0.1510	0.2162	no
GO:0008137	NADH dehydrogenase (ubiquinone) activity	5	1	0.1510	0.2162	no

GO:0008202	steroid metabolic process	5	1	0.1510	0.2162	no
GO:0008235	metalloexopeptidase activity	5	1	0.1510	0.2162	no
GO:0008825	cyclopropane-fatty-acyl-phospholipid synthase activity	5	1	0.1510	0.2162	no
GO:0016876	ligase activity, forming aminoacyl-tRNA and related compounds	5	1	0.1510	0.2162	no
GO:0031461	cullin-RING ubiquitin ligase complex	5	1	0.1510	0.2162	no
GO:0031625	ubiquitin protein ligase binding	5	1	0.1510	0.2162	no
GO:0033177	proton-transporting two-sector ATPase complex, proton-transporting domain	5	1	0.1510	0.2162	no
GO:0043039	tRNA aminoacylation	5	1	0.1510	0.2162	no
GO:0045261	proton-transporting ATP synthase complex, catalytic core F(1)	5	1	0.1510	0.2162	no
GO:0048038	quinone binding	5	1	0.1510	0.2162	no
GO:0008168	methyltransferase activity	49	3	0.1523	0.2041	no
GO:0030246	carbohydrate binding	25	2	0.1609	0.2147	no
GO:0005507	copper ion binding	26	2	0.1682	0.2237	no
GO:0000155	two-component sensor activity	6	1	0.1749	0.2316	no
GO:0004609	phosphatidylserine decarboxylase activity	6	1	0.1749	0.2316	no
GO:0004673	protein histidine kinase activity	6	1	0.1749	0.2316	no
GO:0005083	small GTPase regulator activity	6	1	0.1749	0.2316	no
GO:0006265	DNA topological change	6	1	0.1749	0.2316	no
GO:0006284	base-excision repair	6	1	0.1749	0.2316	no
GO:0006779	porphyrin biosynthetic process	6	1	0.1749	0.2316	no
GO:0008610	lipid biosynthetic process	6	1	0.1749	0.2316	no

GO:0016272	prefoldin complex	6	1	0.1749	0.2316	no
GO:0018106	peptidyl-histidine phosphorylation	6	1	0.1749	0.2316	no
GO:0046983	protein dimerization activity	27	2	0.1754	0.2234	no
GO:0005856	cytoskeleton	7	1	0.1970	0.2499	no
GO:0006306	DNA methylation	7	1	0.1970	0.2499	no
GO:0009073	aromatic amino acid family biosynthetic process	7	1	0.1970	0.2499	no
GO:0009396	folic acid and derivative biosynthetic process	7	1	0.1970	0.2499	no
GO:0016651	oxidoreductase activity, acting on NADH or NADPH	7	1	0.1970	0.2499	no
GO:0019752	carboxylic acid metabolic process	7	1	0.1970	0.2499	no
GO:0019843	rRNA binding	7	1	0.1970	0.2499	no
GO:0030131	clathrin adaptor complex	7	1	0.1970	0.2499	no
GO:0033178	proton-transporting two-sector ATPase complex, catalytic domain	7	1	0.1970	0.2499	no
GO:0046912	transferase activity, transferring acyl groups, acyl groups converted into alkyl on transfer	7	1	0.1970	0.2499	no
GO:0050662	coenzyme binding	32	2	0.2077	0.2539	no
GO:0006396	RNA processing	33	2	0.2134	0.2600	no
GO:0000156	two-component response regulator activity	8	1	0.2173	0.2637	no
GO:0003746	translation elongation factor activity	8	1	0.2173	0.2637	no
GO:0004129	cytochrome-c oxidase activity	8	1	0.2173	0.2637	no
GO:0015035	protein disulfide oxidoreductase activity	8	1	0.2173	0.2637	no
GO:0016455	RNA polymerase II transcription mediator activity	8	1	0.2173	0.2637	no
GO:0016592	mediator complex	8	1	0.2173	0.2637	no
GO:0046933	hydrogen ion transporting ATP synthase activity, rotational mechanism	8	1	0.2173	0.2637	no

GO:0045449	regulation of transcription	67	1	0.2251	0.2664	no
GO:0000160	two-component signal transduction system (phosphorelay)	9	1	0.2360	0.2783	no
GO:0006289	nucleotide-excision repair	9	1	0.2360	0.2783	no
GO:0006357	regulation of transcription from RNA polymerase II promoter	9	1	0.2360	0.2783	no
GO:0008299	isoprenoid biosynthetic process	9	1	0.2360	0.2783	no
GO:0016853	isomerase activity	9	1	0.2360	0.2783	no
GO:0044267	cellular protein metabolic process	9	1	0.2360	0.2783	no
GO:0003993	acid phosphatase activity	10	1	0.2531	0.2923	no
GO:0004499	flavin-containing monooxygenase activity	10	1	0.2531	0.2923	no
GO:0004571	mannosyl-oligosaccharide 1,2-alpha-mannosidase activity	10	1	0.2531	0.2923	no
GO:0006259	DNA metabolic process	10	1	0.2531	0.2923	no
GO:0008236	serine-type peptidase activity	45	2	0.2618	0.2983	no
GO:0016887	ATPase activity	46	2	0.2642	0.3000	no
GO:0004568	chitinase activity	11	1	0.2687	0.3041	no
GO:0006032	chitin catabolic process	11	1	0.2687	0.3041	no
GO:0006342	chromatin silencing	11	1	0.2687	0.3041	no
GO:0006476	protein amino acid deacetylation	11	1	0.2687	0.3041	no
GO:0016747	transferase activity, transferring acyl groups other than amino-acyl groups	11	1	0.2687	0.3041	no
GO:0070403	NAD binding	11	1	0.2687	0.3041	no
GO:0043565	sequence-specific DNA binding	52	2	0.2737	0.3036	no
GO:0003887	DNA-directed DNA polymerase activity	12	1	0.2829	0.3128	no

GO:0005789	endoplasmic reticulum membrane	12	1	0.2829	0.3128	no
GO:0007049	cell cycle	12	1	0.2829	0.3128	no
GO:0008138	protein tyrosine/serine/threonine phosphatase activity	12	1	0.2829	0.3128	no
GO:0016310	phosphorylation	12	1	0.2829	0.3128	no
GO:0016772	transferase activity, transferring phosphorus-containing groups	12	1	0.2829	0.3128	no
GO:0000036	acyl carrier activity	13	1	0.2959	0.3208	no
GO:0004197	cysteine-type endopeptidase activity	13	1	0.2959	0.3208	no
GO:0006099	tricarboxylic acid cycle	13	1	0.2959	0.3208	no
GO:0008654	phospholipid biosynthetic process	13	1	0.2959	0.3208	no
GO:0005694	chromosome	14	1	0.3076	0.3292	no
GO:0006397	mRNA processing	14	1	0.3076	0.3292	no
GO:0008233	peptidase activity	14	1	0.3076	0.3292	no
GO:0016811	hydrolase activity, acting on carbon-nitrogen (but not peptide) bonds, in linear amides	14	1	0.3076	0.3292	no
GO:0005198	structural molecule activity	15	1	0.3181	0.3361	no
GO:0004252	serine-type endopeptidase activity	46	1	0.3254	0.3428	no
GO:0004601	peroxidase activity	16	1	0.3275	0.3439	no
GO:0005089	Rho guanyl-nucleotide exchange factor activity	17	1	0.3359	0.3516	no
GO:0016311	dephosphorylation	17	1	0.3359	0.3516	no
GO:0016874	ligase activity	17	1	0.3359	0.3516	no
GO:0035023	regulation of Rho protein signal transduction	17	1	0.3359	0.3516	no
GO:0048037	cofactor binding	17	1	0.3359	0.3516	no
GO:0016829	lyase activity	18	1	0.3432	0.3538	no

GO:0005576	extracellular region	41	1	0.3463	0.3559	no
GO:0006413	translational initiation	19	1	0.3497	0.3583	no
GO:0003743	translation initiation factor activity	20	1	0.3553	0.3629	no
GO:0006511	ubiquitin-dependent protein catabolic process	35	1	0.3656	0.3723	no
GO:0006470	protein amino acid dephosphorylation	23	1	0.3675	0.3730	no
GO:0008565	protein transporter activity	24	1	0.3701	0.3746	no
GO:0042626	ATPase activity, coupled to transmembrane movement of substances	24	1	0.3701	0.3746	no
GO:0006310	DNA recombination	27	1	0.3744	0.3767	no
GO:0009277	fungal-type cell wall	27	1	0.3744	0.3767	no
GO:0005199	structural constituent of cell wall	29	1	0.3747	0.3747	no

Table S8 Enrichment analysis for the parental genes of exonics based on KEGG pathways. Here the total number of gene is 16127 and the total numbers of parent genes is 561.

KEGG ID	KEGG Annotation	Number of Genes	Number of Parent Gene	Pvalue	Qvalue(FDR)	Significance level
ko01100	Metabolic pathways	675	90	0.0000	0.0000	q<0.01
ko01212	Fatty acid metabolism	21	12	0.0000	0.0000	q<0.01
ko01220	Degradation of aromatic compounds	31	10	0.0000	0.0000	q<0.01
ko01120	Microbial metabolism in diverse environments	187	21	0.0000	0.0000	q<0.01
ko01130	Biosynthesis of antibiotics	193	22	0.0000	0.0000	q<0.01
ko05034	Alcoholism	19	6	0.0000	0.0000	q<0.01
ko05016	Huntington's disease	73	12	0.0000	0.0001	q<0.01
ko01200	Carbon metabolism	91	13	0.0000	0.0000	q<0.01
ko03010	Ribosome	69	12	0.0000	0.0001	q<0.01
ko05010	Alzheimer's disease	54	10	0.0000	0.0001	q<0.01
ko00190	Oxidative phosphorylation	64	11	0.0000	0.0002	q<0.01
ko04152	AMPK signaling pathway	20	6	0.0000	0.0003	q<0.01
ko00710	Carbon fixation in photosynthetic organisms	13	5	0.0000	0.0003	q<0.01
ko05012	Parkinson's disease	46	9	0.0000	0.0003	q<0.01
ko04392	Hippo signaling pathway -multiple species	5	3	0.0000	0.0006	q<0.01
ko00040	Pentose and glucuronate interconversions	8	4	0.0000	0.0006	q<0.01
ko04390	Hippo signaling pathway	11	4	0.0000	0.0006	q<0.01

ko00250	Alanine, aspartate and glutamate metabolism	24	6	0.0001	0.0006	q<0.01
ko04141	Protein processing in endoplasmic reticulum	81	10	0.0001	0.0007	q<0.01
ko03015	mRNA surveillance pathway	43	8	0.0001	0.0008	q<0.01
ko05168	Herpes simplex infection	26	6	0.0002	0.0016	q<0.01
ko04120	Ubiquitin mediated proteolysis	53	8	0.0002	0.0018	q<0.01
ko04921	Oxytocin signaling pathway	13	4	0.0002	0.0022	q<0.01
ko04624	Toll and Imd signaling pathway	7	3	0.0004	0.0034	q<0.01
ko04011	MAPK signaling pathway - yeast	58	7	0.0004	0.0034	q<0.01
ko00330	Arginine and proline metabolism	34	6	0.0004	0.0035	q<0.01
ko00020	Citrate cycle	26	5	0.0007	0.0056	q<0.01
ko04723	Retrograde endocannabinoid signaling	8	3	0.0008	0.0056	q<0.01
ko05169	Epstein-Barr virus infection	67	8	0.0008	0.0055	q<0.01
ko04212	Longevity regulating pathway - worm	20	4	0.0010	0.0071	q<0.01
ko01210	2-Oxocarboxylic acid metabolism	33	5	0.0011	0.0073	q<0.01
ko04975	Fat digestion and absorption	2	2	0.0012	0.0079	q<0.01
ko04013	MAPK signaling pathway - fly	14	3	0.0013	0.0080	q<0.01
ko04114	Oocyte meiosis	32	5	0.0013	0.0081	q<0.01
ko04111	Cell cycle - yeast	71	8	0.0015	0.0092	q<0.01
ko04261	Adrenergic signaling in cardiomyocytes	13	3	0.0020	0.0114	0.01<q<0.05
ko04728	Dopaminergic synapse	8	3	0.0020	0.0114	0.01<q<0.05
ko00340	Histidine metabolism	19	4	0.0022	0.0120	0.01<q<0.05
ko00480	Glutathione metabolism	48	4	0.0022	0.0120	0.01<q<0.05
ko00510	N-Glycan biosynthesis	23	4	0.0022	0.0120	0.01<q<0.05
ko00620	Pyruvate metabolism	69	7	0.0025	0.0126	0.01<q<0.05
ko05110	Vibrio cholerae infection	18	4	0.0027	0.0134	0.01<q<0.05

ko04310	Wnt signaling pathway	12	3	0.0028	0.0138	0.01<q<0.05
ko00270	Cysteine and methionine metabolism	38	5	0.0034	0.0161	0.01<q<0.05
ko00590	Arachidonic acid metabolism	3	2	0.0035	0.0162	0.01<q<0.05
ko04710	Circadian rhythm	4	2	0.0035	0.0162	0.01<q<0.05
ko05031	Amphetamine addiction	3	2	0.0035	0.0162	0.01<q<0.05
ko05133	Pertussis	5	2	0.0035	0.0162	0.01<q<0.05
ko04530	Tight junction	11	3	0.0039	0.0167	0.01<q<0.05
ko04922	Glucagon signaling pathway	10	3	0.0039	0.0167	0.01<q<0.05
ko04144	Endocytosis	51	6	0.0049	0.0198	0.01<q<0.05
ko00513	Various types of N-glycan biosynthesis	20	3	0.0052	0.0209	0.01<q<0.05
ko00650	Butanoate metabolism	14	3	0.0052	0.0209	0.01<q<0.05
ko00625	Chloroalkane and chloroalkene degradation	5	2	0.0068	0.0260	0.01<q<0.05
ko04270	Vascular smooth muscle contraction	6	2	0.0068	0.0260	0.01<q<0.05
ko04340	Hedgehog signaling pathway	5	2	0.0068	0.0260	0.01<q<0.05
ko04350	TGF-beta signaling pathway	6	2	0.0068	0.0260	0.01<q<0.05
ko04720	Long-term potentiation	6	2	0.0068	0.0260	0.01<q<0.05
ko04750	Inflammatory mediator regulation of TRP channels	4	2	0.0068	0.0260	0.01<q<0.05
ko04932	Non-alcoholic fatty liver disease	41	5	0.0079	0.0273	0.01<q<0.05
ko00310	Lysine degradation	15	3	0.0084	0.0287	0.01<q<0.05
ko00220	Arginine biosynthesis	16	3	0.0103	0.0347	0.01<q<0.05
ko00360	Phenylalanine metabolism	17	3	0.0103	0.0347	0.01<q<0.05
ko00950	Isoquinoline alkaloid biosynthesis	8	2	0.0109	0.0353	0.01<q<0.05
ko00960	Tropane, piperidine and pyridine alkaloid biosynthesis	8	2	0.0109	0.0353	0.01<q<0.05

ko04022	cGMP-PKG signaling pathway	10	2	0.0109	0.0353	0.01<q<0.05
ko04341	Hedgehog signaling pathway - fly	6	2	0.0109	0.0353	0.01<q<0.05
ko04391	Hippo signaling pathway - fly	8	2	0.0109	0.0353	0.01<q<0.05
ko00520	Amino sugar and nucleotide sugar metabolism	52	4	0.0113	0.0341	0.01<q<0.05
ko03013	RNA transport	100	8	0.0138	0.0409	0.01<q<0.05
ko05202	Transcriptional misregulation in cancer	32	3	0.0148	0.0435	0.01<q<0.05
ko00980	Metabolism of xenobiotics by cytochrome P450	36	2	0.0157	0.0455	0.01<q<0.05
ko01040	Biosynthesis of unsaturated fatty acids	14	2	0.0157	0.0455	0.01<q<0.05
ko03040	Spliceosome	87	7	0.0181	0.0509	no
ko00970	Aminoacyl-tRNA biosynthesis	42	4	0.0195	0.0540	no
ko00680	Methane metabolism	22	3	0.0201	0.0551	no
ko00053	Ascorbate and aldarate metabolism	9	2	0.0213	0.0574	no
ko00982	Drug metabolism - cytochrome P450	37	2	0.0213	0.0574	no
ko05204	Chemical carcinogenesis	37	2	0.0213	0.0574	no
ko05152	Tuberculosis	29	3	0.0231	0.0600	no
ko03050	Proteasome	36	4	0.0234	0.0601	no
ko00626	Naphthalene degradation	26	3	0.0262	0.0665	no
ko04721	Synaptic vesicle cycle	21	3	0.0262	0.0665	no
ko04024	cAMP signaling pathway	17	2	0.0274	0.0678	no
ko04072	Phospholipase D signaling pathway	10	2	0.0274	0.0678	no
ko05162	Measles	10	2	0.0274	0.0678	no
ko04722	Neurotrophin signaling pathway	15	2	0.0340	0.0812	no
ko00903	Limonene and pinene degradation	2	1	0.0348	0.0822	no
ko04744	Phototransduction	1	1	0.0348	0.0822	no

ko04745	Phototransduction - fly	1	1	0.0348	0.0822	no
ko04924	Renin secretion	1	1	0.0348	0.0822	no
ko04925	Aldosterone synthesis and secretion	1	1	0.0348	0.0822	no
ko04142	Lysosome	63	3	0.0367	0.0820	no
ko00380	Tryptophan metabolism	31	3	0.0404	0.0895	no
ko00300	Lysine biosynthesis	13	2	0.0410	0.0897	no
ko04064	NF-kappa B signaling pathway	12	2	0.0410	0.0897	no
ko04211	Longevity regulating pathway	17	2	0.0410	0.0897	no
ko04260	Cardiac muscle contraction	13	2	0.0410	0.0897	no
ko04966	Collecting duct acid secretion	10	2	0.0410	0.0897	no
ko05323	Rheumatoid arthritis	11	2	0.0484	0.1006	no
ko03020	RNA polymerase	29	3	0.0526	0.1083	no
ko04145	Phagosome	28	3	0.0526	0.1083	no
ko00670	One carbon pool by folate	12	2	0.0560	0.1131	no
ko00071	Fatty acid degradation	19	2	0.0639	0.1278	no
ko00561	Glycerolipid metabolism	17	2	0.0639	0.1278	no
ko04014	Ras signaling pathway	15	2	0.0639	0.1278	no
ko04068	FoxO signaling pathway	22	2	0.0639	0.1278	no
ko04071	Sphingolipid signaling pathway	25	2	0.0639	0.1278	no
ko04140	Regulation of autophagy	14	2	0.0639	0.1278	no
ko04623	Cytosolic DNA-sensing pathway	13	2	0.0639	0.1278	no
ko00830	Retinol metabolism	2	1	0.0672	0.1258	no
ko04016	MAPK signaling pathway - plant	4	1	0.0672	0.1258	no
ko04740	Olfactory transduction	2	1	0.0672	0.1258	no
ko04964	Proximal tubule bicarbonate reclamation	6	1	0.0672	0.1258	no

ko04970	Salivary secretion	5	1	0.0672	0.1258	no
ko04971	Gastric acid secretion	5	1	0.0672	0.1258	no
ko04972	Pancreatic secretion	5	1	0.0672	0.1258	no
ko04146	Peroxisome	43	3	0.0702	0.1237	no
ko00051	Fructose and mannose metabolism	15	2	0.0720	0.1258	no
ko05131	Shigellosis	17	2	0.0720	0.1258	no
ko04910	Insulin signaling pathway	40	3	0.0747	0.1285	no
ko00230	Purine metabolism	79	5	0.0761	0.1298	no
ko00640	Propanoate metabolism	20	2	0.0802	0.1356	no
ko05120	Epithelial cell signaling in Helicobacter pylori infection	15	2	0.0802	0.1356	no
ko05205	Proteoglycans in cancer	18	2	0.0802	0.1356	no
ko00410	beta-Alanine metabolism	19	2	0.0968	0.1597	no
ko04810	Regulation of actin cytoskeleton	20	2	0.0968	0.1597	no
ko00072	Synthesis and degradation of ketone bodies	4	1	0.0972	0.1580	no
ko04614	Renin-angiotensin system	5	1	0.0972	0.1580	no
ko04712	Circadian rhythm - plant	4	1	0.0972	0.1580	no
ko04713	Circadian entrainment	5	1	0.0972	0.1580	no
ko04730	Long-term depression	5	1	0.0972	0.1580	no
ko04913	Ovarian steroidogenesis	3	1	0.0972	0.1580	no
ko04916	Melanogenesis	5	1	0.0972	0.1580	no
ko04920	Adipocytokine signaling pathway	4	1	0.0972	0.1580	no
ko04923	Regulation of lipolysis in adipocytes	3	1	0.0972	0.1580	no
ko05220	Chronic myeloid leukemia	6	1	0.0972	0.1580	no
ko00621	Dioxin degradation	24	2	0.1051	0.1584	no

ko00624	Polycyclic aromatic hydrocarbon degradation	24	2	0.1051	0.1584	no
ko05206	MicroRNAs in cancer	22	2	0.1051	0.1584	no
ko01524	Platinum drug resistance	78	3	0.1076	0.1587	no
ko00630	Glyoxylate and dicarboxylate metabolism	25	2	0.1134	0.1660	no
ko03060	Protein export	20	2	0.1216	0.1768	no
ko04130	SNARE interactions in vesicular transport	22	2	0.1216	0.1768	no
ko00362	Benzoate degradation	5	1	0.1251	0.1795	no
ko04020	Calcium signaling pathway	4	1	0.1251	0.1795	no
ko04330	Notch signaling pathway	5	1	0.1251	0.1795	no
ko04380	Osteoclast differentiation	7	1	0.1251	0.1795	no
ko04626	Plant-pathogen interaction	5	1	0.1251	0.1795	no
ko04918	Thyroid hormone synthesis	7	1	0.1251	0.1795	no
ko05140	Leishmaniasis	6	1	0.1251	0.1795	no
ko05142	Chagas disease	6	1	0.1251	0.1795	no
ko05214	Glioma	6	1	0.1251	0.1795	no
ko03410	Base excision repair	22	2	0.1297	0.1752	no
ko04113	Meiosis - yeast	50	3	0.1351	0.1813	no
ko00500	Starch and sucrose metabolism	40	2	0.1377	0.1836	no
ko00627	Aminobenzoate degradation	25	2	0.1456	0.1929	no
ko00514	Other types of O-glycan biosynthesis	6	1	0.1510	0.1988	no
ko00643	Styrene degradation	5	1	0.1510	0.1988	no
ko04668	TNF signaling pathway	7	1	0.1510	0.1988	no
ko04933	AGE-RAGE signaling pathway in diabetic complications	8	1	0.1510	0.1988	no
ko04976	Bile secretion	8	1	0.1510	0.1988	no

ko05222	Small cell lung cancer	5	1	0.1510	0.1988	no
ko04110	Cell cycle	59	3	0.1523	0.1932	no
ko05200	Pathways in cancer	28	2	0.1533	0.1933	no
ko04919	Thyroid hormone signaling pathway	39	2	0.1609	0.2016	no
ko04360	Axon guidance	8	1	0.1749	0.2178	no
ko04520	Adherens junction	9	1	0.1749	0.2178	no
ko04611	Platelet activation	8	1	0.1749	0.2178	no
ko04915	Estrogen signaling pathway	10	1	0.1749	0.2178	no
ko04931	Insulin resistance	7	1	0.1749	0.2178	no
ko00240	Pyrimidine metabolism	63	3	0.1918	0.2320	no
ko00780	Biotin metabolism	8	1	0.1970	0.2368	no
ko04015	Rap1 signaling pathway	9	1	0.1970	0.2368	no
ko04510	Focal adhesion	10	1	0.1970	0.2368	no
ko05322	Systemic lupus erythematosus	13	1	0.1970	0.2368	no
ko03008	Ribosome biogenesis in eukaryotes	71	3	0.2117	0.2488	no
ko05166	HTLV-I infection	38	2	0.2134	0.2494	no
ko01523	Antifolate resistance	8	1	0.2173	0.2525	no
ko02024	Quorum sensing	9	1	0.2173	0.2525	no
ko03320	PPAR signaling pathway	15	1	0.2173	0.2525	no
ko04122	Sulfur relay system	8	1	0.2173	0.2525	no
ko04370	VEGF signaling pathway	10	1	0.2173	0.2525	no
ko04961	Endocrine and other factor-regulated calcium reabsorption	11	1	0.2173	0.2525	no
ko03420	Nucleotide excision repair	38	2	0.2337	0.2628	no
ko00720	Carbon fixation pathways in prokaryotes	14	1	0.2360	0.2639	no

ko04070	Phosphatidylinositol signaling system	11	1	0.2360	0.2639	no
ko00910	Nitrogen metabolism	13	1	0.2531	0.2800	no
ko05100	Bacterial invasion of epithelial cells	10	1	0.2531	0.2800	no
ko05160	Hepatitis C	13	1	0.2531	0.2800	no
ko00600	Sphingolipid metabolism	15	1	0.2687	0.2926	no
ko02020	Two-component system	14	1	0.2687	0.2926	no
ko04962	Vasopressin-regulated water reabsorption	11	1	0.2687	0.2926	no
ko03018	RNA degradation	51	2	0.2699	0.2894	no
ko05230	Central carbon metabolism in cancer	15	1	0.2829	0.3018	no
ko00920	Sulfur metabolism	14	1	0.2959	0.3140	no
ko04912	GnRH signaling pathway	18	1	0.3076	0.3247	no
ko00052	Galactose metabolism	23	1	0.3275	0.3440	no
ko00860	Porphyrin and chlorophyll metabolism	17	1	0.3275	0.3440	no
ko00900	Terpenoid backbone biosynthesis	17	1	0.3275	0.3440	no
ko04151	PI3K-Akt signaling pathway	26	1	0.3359	0.3475	no
ko04150	mTOR signaling pathway	24	1	0.3497	0.3601	no
ko05164	Influenza A	23	1	0.3497	0.3601	no
ko03430	Mismatch repair	43	1	0.3500	0.3569	no
ko00564	Glycerophospholipid metabolism	27	1	0.3601	0.3654	no
ko03460	Fanconi anemia pathway	23	1	0.3642	0.3677	no
ko00100	Steroid biosynthesis	37	1	0.3747	0.3765	no
ko05203	Viral carcinogenesis	36	1	0.3748	0.3748	no

Table S9 Differential enrichment analysis of parental genes for GO terms among the three developmental stages. Here the total number of genes and the parent genes of cricRNAs are 16127 and 514 respectively.

ID	GO Annotation	No. of Genes Mapped to This Category	Number of Parent Genes Mapped to this Category	P value	Q value (FDR)	Significance level	Stages
GO:0055114	oxidation reduction	618	47	0.0000	0.0000	q<0.01	M
GO:0005622	intracellular	381	34	0.0000	0.0000	q<0.01	M
GO:0003824	catalytic activity	791	53	0.0000	0.0000	q<0.01	M
GO:0016020	membrane	312	29	0.0000	0.0000	q<0.01	M
GO:0006412	translation	149	18	0.0000	0.0001	q<0.01	M
GO:0003735	structural constituent of ribosome	107	15	0.0000	0.0001	q<0.01	M
GO:0008483	transaminase activity	8	5	0.0000	0.0001	q<0.01	M
GO:0005525	GTP binding	97	14	0.0000	0.0001	q<0.01	M
GO:0005840	ribosome	105	14	0.0000	0.0002	q<0.01	M
GO:0055085	transmembrane transport	340	27	0.0000	0.0003	q<0.01	M
GO:0006810	transport	174	17	0.0000	0.0009	q<0.01	M
GO:0000166	nucleotide binding	214	19	0.0000	0.0011	q<0.01	M
GO:0030170	pyridoxal phosphate binding	56	9	0.0001	0.0013	q<0.01	M
GO:0006457	protein folding	57	9	0.0001	0.0014	q<0.01	M
GO:0015031	protein transport	57	9	0.0001	0.0014	q<0.01	M
GO:0005737	cytoplasm	138	14	0.0001	0.0020	q<0.01	M

GO:0016491	oxidoreductase activity	482	31	0.0001	0.0019	q<0.01	M
GO:0019001	guanyl nucleotide binding	16	5	0.0001	0.0018	q<0.01	M
GO:0016021	integral to membrane	251	20	0.0001	0.0018	q<0.01	M
GO:0020037	heme binding	257	20	0.0001	0.0023	q<0.01	M
GO:0005506	iron ion binding	260	20	0.0002	0.0026	q<0.01	M
GO:0005215	transporter activity	65	9	0.0002	0.0025	q<0.01	M
GO:0003924	GTPase activity	53	8	0.0002	0.0030	q<0.01	M
GO:0009055	electron carrier activity	250	19	0.0003	0.0037	q<0.01	M
GO:0008152	metabolic process	683	38	0.0003	0.0039	q<0.01	M
GO:0016616	oxidoreductase activity, acting on the CH-OH group of donors, NAD or NADP as acceptor	20	5	0.0003	0.0038	q<0.01	M
GO:0005488	binding	476	29	0.0004	0.0044	q<0.01	M
GO:0015986	ATP synthesis coupled proton transport	22	5	0.0005	0.0057	q<0.01	M
GO:0006350	transcription	76	9	0.0005	0.0059	q<0.01	M
GO:0005509	calcium ion binding	34	6	0.0006	0.0060	q<0.01	M
GO:0007186	G-protein coupled receptor protein signaling pathway	23	5	0.0006	0.0063	q<0.01	M
GO:0051082	unfolded protein binding	35	6	0.0007	0.0066	q<0.01	M
GO:0009987	cellular process	14	4	0.0007	0.0072	q<0.01	M
GO:0046872	metal ion binding	37	6	0.0009	0.0083	q<0.01	M
GO:0004497	monooxygenase activity	258	18	0.0010	0.0090	q<0.01	M
GO:0007165	signal transduction	67	8	0.0010	0.0089	q<0.01	M
GO:0016192	vesicle-mediated transport	38	6	0.0010	0.0088	q<0.01	M
GO:0004470	malic enzyme activity	2	2	0.0010	0.0086	q<0.01	M

GO:0004768	stearoyl-CoA 9-desaturase activity	2	2	0.0010	0.0086	q<0.01	M
GO:0051287	NAD or NADH binding	28	5	0.0015	0.0122	0.01<q<0.05	M
GO:0030145	manganese ion binding	8	3	0.0015	0.0121	0.01<q<0.05	M
GO:0007264	small GTPase mediated signal transduction	42	6	0.0017	0.0129	0.01<q<0.05	M
GO:0004871	signal transducer activity	29	5	0.0018	0.0133	0.01<q<0.05	M
GO:0003677	DNA binding	319	20	0.0018	0.0131	0.01<q<0.05	M
GO:0005634	nucleus	345	21	0.0020	0.0141	0.01<q<0.05	M
GO:0006108	malate metabolic process	3	2	0.0029	0.0206	0.01<q<0.05	M
GO:0015078	hydrogen ion transmembrane transporter activity	10	3	0.0031	0.0211	0.01<q<0.05	M
GO:0015935	small ribosomal subunit	10	3	0.0031	0.0211	0.01<q<0.05	M
GO:0016787	hydrolase activity	123	10	0.0041	0.0267	0.01<q<0.05	M
GO:0006633	fatty acid biosynthetic process	11	3	0.0041	0.0264	0.01<q<0.05	M
GO:0006334	nucleosome assembly	22	4	0.0042	0.0264	0.01<q<0.05	M
GO:0022891	substrate-specific transmembrane transporter activity	22	4	0.0042	0.0264	0.01<q<0.05	M
GO:0005743	mitochondrial inner membrane	23	4	0.0049	0.0298	0.01<q<0.05	M
GO:0000276	mitochondrial proton-transporting ATP synthase complex, coupling factor F(o)	4	2	0.0057	0.0340	0.01<q<0.05	M
GO:0004674	protein serine/threonine kinase activity	173	12	0.0059	0.0344	0.01<q<0.05	M
GO:0005524	ATP binding	644	31	0.0061	0.0352	0.01<q<0.05	M
GO:0050660	FAD binding	111	9	0.0062	0.0348	0.01<q<0.05	M
GO:0005975	carbohydrate metabolic process	220	14	0.0064	0.0354	0.01<q<0.05	M
GO:0006913	nucleocytoplasmic transport	13	3	0.0067	0.0364	0.01<q<0.05	M
GO:0004519	endonuclease activity	14	3	0.0082	0.0441	0.01<q<0.05	M

GO:0006164	purine nucleotide biosynthetic process	5	2	0.0092	0.0486	0.01<q<0.05	M
GO:0005783	endoplasmic reticulum	15	3	0.0100	0.0517	no	M
GO:0003779	actin binding	16	3	0.0119	0.0606	no	M
GO:0006355	regulation of transcription, DNA-dependent	127	9	0.0129	0.0651	no	M
GO:0006904	vesicle docking during exocytosis	6	2	0.0134	0.0662	no	M
GO:0004672	protein kinase activity	202	12	0.0158	0.0771	no	M
GO:0043169	cation binding	91	7	0.0176	0.0847	no	M
GO:0016881	acid-amino acid ligase activity	7	2	0.0181	0.0858	no	M
GO:0016773	phosphotransferase activity, alcohol group as acceptor	19	3	0.0186	0.0869	no	M
GO:0000786	nucleosome	20	3	0.0212	0.0976	no	M
GO:0016769	transferase activity, transferring nitrogenous groups	20	3	0.0212	0.0976	no	M
GO:0005216	ion channel activity	8	2	0.0234	0.1046	no	M
GO:0030163	protein catabolic process	8	2	0.0234	0.1046	no	M
GO:0004185	serine-type carboxypeptidase activity	21	3	0.0240	0.1043	no	M
GO:0009058	biosynthetic process	57	5	0.0254	0.1091	no	M
GO:0046961	proton-transporting ATPase activity, rotational mechanism	9	2	0.0291	0.1234	no	M
GO:0005515	protein binding	363	17	0.0307	0.1283	no	M
GO:0003937	IMP cyclohydrolase activity	1	1	0.0319	0.1316	no	M
GO:0004019	adenylosuccinate synthase activity	1	1	0.0319	0.1316	no	M
GO:0004043	L-aminoadipate-semialdehyde dehydrogenase activity	1	1	0.0319	0.1316	no	M
GO:0004076	biotin synthase activity	1	1	0.0319	0.1316	no	M

GO:0004357	glutamate-cysteine ligase activity	1	1	0.0319	0.1316	no	M
GO:0004402	histone acetyltransferase activity	1	1	0.0319	0.1316	no	M
GO:0004420	hydroxymethylglutaryl-CoA reductase (NADPH) activity	1	1	0.0319	0.1316	no	M
GO:0004450	isocitrate dehydrogenase (NADP+) activity	1	1	0.0319	0.1316	no	M
GO:0004474	malate synthase activity	1	1	0.0319	0.1316	no	M
GO:0004602	glutathione peroxidase activity	1	1	0.0319	0.1316	no	M
GO:0004611	phosphoenolpyruvate carboxykinase activity	1	1	0.0319	0.1316	no	M
GO:0004612	phosphoenolpyruvate carboxykinase (ATP) activity	1	1	0.0319	0.1316	no	M
GO:0004619	phosphoglycerate mutase activity	1	1	0.0319	0.1316	no	M
GO:0004643	phosphoribosylaminoimidazolecarboxamide formyltransferase activity	1	1	0.0319	0.1316	no	M
GO:0004651	polynucleotide 5'-phosphatase activity	1	1	0.0319	0.1316	no	M
GO:0004801	transaldolase activity	1	1	0.0319	0.1316	no	M
GO:0004829	threonine-tRNA ligase activity	1	1	0.0319	0.1316	no	M
GO:0004832	valine-tRNA ligase activity	1	1	0.0319	0.1316	no	M
GO:0004853	uroporphyrinogen decarboxylase activity	1	1	0.0319	0.1316	no	M
GO:0005094	Rho GDP-dissociation inhibitor activity	1	1	0.0319	0.1316	no	M
GO:0005665	DNA-directed RNA polymerase II, core complex	1	1	0.0319	0.1316	no	M
GO:0006007	glucose catabolic process	1	1	0.0319	0.1316	no	M
GO:0006097	glyoxylate cycle	1	1	0.0319	0.1316	no	M
GO:0006102	isocitrate metabolic process	1	1	0.0319	0.1316	no	M

GO:0006188	IMP biosynthetic process	1	1	0.0319	0.1316	no	M
GO:0006435	threonyl-tRNA aminoacylation	1	1	0.0319	0.1316	no	M
GO:0006438	valyl-tRNA aminoacylation	1	1	0.0319	0.1316	no	M
GO:0006452	translational frameshifting	1	1	0.0319	0.1316	no	M
GO:0007585	respiratory gaseous exchange	1	1	0.0319	0.1316	no	M
GO:0008250	oligosaccharyltransferase complex	1	1	0.0319	0.1316	no	M
GO:0008410	CoA-transferase activity	1	1	0.0319	0.1316	no	M
GO:0009102	biotin biosynthetic process	1	1	0.0319	0.1316	no	M
GO:0015936	coenzyme A metabolic process	1	1	0.0319	0.1316	no	M
GO:0016246	RNA interference	1	1	0.0319	0.1316	no	M
GO:0016442	RNA-induced silencing complex	1	1	0.0319	0.1316	no	M
GO:0016847	1-aminocyclopropane-1-carboxylate synthase activity	1	1	0.0319	0.1316	no	M
GO:0017076	purine nucleotide binding	1	1	0.0319	0.1316	no	M
GO:0017136	NAD-dependent histone deacetylase activity	1	1	0.0319	0.1316	no	M
GO:0019478	D-amino acid catabolic process	1	1	0.0319	0.1316	no	M
GO:0031533	mRNA cap methyltransferase complex	1	1	0.0319	0.1316	no	M
GO:0042578	phosphoric ester hydrolase activity	1	1	0.0319	0.1316	no	M
GO:0045901	positive regulation of translational elongation	1	1	0.0319	0.1316	no	M
GO:0045905	positive regulation of translational termination	1	1	0.0319	0.1316	no	M
GO:0048193	Golgi vesicle transport	1	1	0.0319	0.1316	no	M
GO:0051186	cofactor metabolic process	1	1	0.0319	0.1316	no	M

GO:0016810	hydrolase activity, acting on carbon-nitrogen (but not peptide) bonds	24	3	0.0331	0.0867	no	M
GO:0006066	alcohol metabolic process	25	3	0.0364	0.0946	no	M
GO:0006468	protein amino acid phosphorylation	240	12	0.0390	0.1005	no	M
GO:0016614	oxidoreductase activity, acting on CH-OH group of donors	45	4	0.0406	0.1038	no	M
GO:0003684	damaged DNA binding	11	2	0.0417	0.1057	no	M
GO:0006281	DNA repair	46	4	0.0431	0.1084	no	M
GO:0008270	zinc ion binding	598	24	0.0448	0.1119	no	M
GO:0051539	4 iron, 4 sulfur cluster binding	12	2	0.0485	0.1200	no	M
GO:0031072	heat shock protein binding	29	3	0.0509	0.1251	no	M
GO:0004222	metalloendopeptidase activity	30	3	0.0547	0.1335	no	M
GO:0006508	proteolysis	232	11	0.0552	0.1337	no	M
GO:0004175	endopeptidase activity	13	2	0.0554	0.1332	no	M
GO:0004177	aminopeptidase activity	13	2	0.0554	0.1332	no	M
GO:0004518	nuclease activity	13	2	0.0554	0.1332	no	M
GO:0006629	lipid metabolic process	51	4	0.0562	0.1320	no	M
GO:0006418	tRNA aminoacylation for protein translation	31	3	0.0587	0.1369	no	M
GO:0003951	NAD+ kinase activity	2	1	0.0617	0.1430	no	M
GO:0004066	asparagine synthase (glutamine-hydrolyzing) activity	2	1	0.0617	0.1430	no	M
GO:0004084	branched-chain-amino-acid transaminase activity	2	1	0.0617	0.1430	no	M
GO:0004348	glucosylceramidase activity	2	1	0.0617	0.1430	no	M
GO:0004615	phosphomannomutase activity	2	1	0.0617	0.1430	no	M

GO:0004816	asparagine-tRNA ligase activity	2	1	0.0617	0.1430	no	M
GO:0005764	lysosome	2	1	0.0617	0.1430	no	M
GO:0006421	asparaginyl-tRNA aminoacylation	2	1	0.0617	0.1430	no	M
GO:0006525	arginine metabolic process	2	1	0.0617	0.1430	no	M
GO:0006529	asparagine biosynthetic process	2	1	0.0617	0.1430	no	M
GO:0006535	cysteine biosynthetic process from serine	2	1	0.0617	0.1430	no	M
GO:0006750	glutathione biosynthetic process	2	1	0.0617	0.1430	no	M
GO:0007040	lysosome organization	2	1	0.0617	0.1430	no	M
GO:0008612	peptidyl-lysine modification to hypusine	2	1	0.0617	0.1430	no	M
GO:0009081	branched chain family amino acid metabolic process	2	1	0.0617	0.1430	no	M
GO:0009085	lysine biosynthetic process	2	1	0.0617	0.1430	no	M
GO:0016841	ammonia-lyase activity	2	1	0.0617	0.1430	no	M
GO:0018279	protein amino acid N-linked glycosylation via asparagine	2	1	0.0617	0.1430	no	M
GO:0019307	mannose biosynthetic process	2	1	0.0617	0.1430	no	M
GO:0030288	outer membrane-bounded periplasmic space	2	1	0.0617	0.1430	no	M
GO:0045263	proton-transporting ATP synthase complex, coupling factor F(o)	2	1	0.0617	0.1430	no	M
GO:0003755	peptidyl-prolyl cis-trans isomerase activity	14	2	0.0626	0.1261	no	M
GO:0004298	threonine-type endopeptidase activity	14	2	0.0626	0.1261	no	M
GO:0005839	proteasome core complex	14	2	0.0626	0.1261	no	M
GO:0006807	nitrogen compound metabolic process	14	2	0.0626	0.1261	no	M
GO:0051603	proteolysis involved in cellular protein	14	2	0.0626	0.1261	no	M

	catabolic process						
GO:0004812	aminoacyl-tRNA ligase activity	32	3	0.0627	0.1224	no	M
GO:0003700	transcription factor activity	105	6	0.0682	0.1323	no	M
GO:0051536	iron-sulfur cluster binding	16	2	0.0774	0.1493	no	M
GO:0003676	nucleic acid binding	461	17	0.0830	0.1591	no	M
GO:0010181	FMN binding	37	3	0.0836	0.1593	no	M
GO:0006464	protein modification process	17	2	0.0850	0.1609	no	M
GO:0016884	carbon-nitrogen ligase activity, with glutamine as amido-N-donor	17	2	0.0850	0.1609	no	M
GO:0000030	mannosyltransferase activity	3	1	0.0896	0.1678	no	M
GO:0003918	DNA topoisomerase (ATP-hydrolyzing) activity	3	1	0.0896	0.1678	no	M
GO:0004356	glutamate-ammonia ligase activity	3	1	0.0896	0.1678	no	M
GO:0004407	histone deacetylase activity	3	1	0.0896	0.1678	no	M
GO:0004579	dolichyl-diphosphooligosaccharide-protein glycotransferase activity	3	1	0.0896	0.1678	no	M
GO:0005247	voltage-gated chloride channel activity	3	1	0.0896	0.1678	no	M
GO:0006366	transcription from RNA polymerase II promoter	3	1	0.0896	0.1678	no	M
GO:0006370	mRNA capping	3	1	0.0896	0.1678	no	M
GO:0006465	signal peptide processing	3	1	0.0896	0.1678	no	M
GO:0006493	protein amino acid O-linked glycosylation	3	1	0.0896	0.1678	no	M
GO:0006559	L-phenylalanine catabolic process	3	1	0.0896	0.1678	no	M
GO:0006665	sphingolipid metabolic process	3	1	0.0896	0.1678	no	M
GO:0006915	apoptosis	3	1	0.0896	0.1678	no	M

GO:0015450	P-P-bond-hydrolysis-driven protein transmembrane transporter activity	3	1	0.0896	0.1678	no	M
GO:0016568	chromatin modification	3	1	0.0896	0.1678	no	M
GO:0016575	histone deacetylation	3	1	0.0896	0.1678	no	M
GO:0016705	oxidoreductase activity, acting on paired donors, with incorporation or reduction of molecular oxygen	3	1	0.0896	0.1678	no	M
GO:0016717	oxidoreductase activity, acting on paired donors, with oxidation of a pair of donors resulting in the reduction of molecular oxygen to two molecules of water	3	1	0.0896	0.1678	no	M
GO:0016813	hydrolase activity, acting on carbon-nitrogen (but not peptide) bonds, in linear amidines	3	1	0.0896	0.1678	no	M
GO:0031177	phosphopantetheine binding	3	1	0.0896	0.1678	no	M
GO:0033179	proton-transporting V-type ATPase, V0 domain	3	1	0.0896	0.1678	no	M
GO:0043022	ribosome binding	3	1	0.0896	0.1678	no	M
GO:0006979	response to oxidative stress	19	2	0.1001	0.1662	no	M
GO:0000287	magnesium ion binding	41	3	0.1008	0.1664	no	M
GO:0006886	intracellular protein transport	67	4	0.1028	0.1689	no	M
GO:0017111	nucleoside-triphosphatase activity	96	5	0.1056	0.1726	no	M
GO:0006520	cellular amino acid metabolic process	20	2	0.1077	0.1752	no	M
GO:0019787	small conjugating protein ligase activity	20	2	0.1077	0.1752	no	M
GO:0043687	post-translational protein modification	20	2	0.1077	0.1752	no	M
GO:0045454	cell redox homeostasis	20	2	0.1077	0.1752	no	M
GO:0050661	NADP or NADPH binding	20	2	0.1077	0.1752	no	M

GO:0051246	regulation of protein metabolic process	20	2	0.1077	0.1752	no	M
GO:0003849	3-deoxy-7-phosphoheptulonate synthase activity	4	1	0.1157	0.1826	no	M
GO:0004089	carbonate dehydratase activity	4	1	0.1157	0.1826	no	M
GO:0004784	superoxide dismutase activity	4	1	0.1157	0.1826	no	M
GO:0005740	mitochondrial envelope	4	1	0.1157	0.1826	no	M
GO:0006094	gluconeogenesis	4	1	0.1157	0.1826	no	M
GO:0006098	pentose-phosphate shunt	4	1	0.1157	0.1826	no	M
GO:0006821	chloride transport	4	1	0.1157	0.1826	no	M
GO:0008121	ubiquinol-cytochrome-c reductase activity	4	1	0.1157	0.1826	no	M
GO:0015976	carbon utilization	4	1	0.1157	0.1826	no	M
GO:0016624	oxidoreductase activity, acting on the aldehyde or oxo group of donors, disulfide as acceptor	4	1	0.1157	0.1826	no	M
GO:0019318	hexose metabolic process	4	1	0.1157	0.1826	no	M
GO:0030833	regulation of actin filament polymerization	4	1	0.1157	0.1826	no	M
GO:0031105	septin complex	4	1	0.1157	0.1826	no	M
GO:0046907	intracellular transport	4	1	0.1157	0.1826	no	M
GO:0003723	RNA binding	104	5	0.1227	0.1812	no	M
GO:0016788	hydrolase activity, acting on ester bonds	23	2	0.1302	0.1915	no	M
GO:0016791	phosphatase activity	23	2	0.1302	0.1915	no	M
GO:0008168	methyltransferase activity	49	3	0.1345	0.1960	no	M
GO:0004721	phosphoprotein phosphatase activity	5	1	0.1400	0.2031	no	M
GO:0004815	aspartate-tRNA ligase activity	5	1	0.1400	0.2031	no	M
GO:0006422	aspartyl-tRNA aminoacylation	5	1	0.1400	0.2031	no	M

GO:0006801	superoxide metabolic process	5	1	0.1400	0.2031	no	M
GO:0006811	ion transport	5	1	0.1400	0.2031	no	M
GO:0008137	NADH dehydrogenase (ubiquinone) activity	5	1	0.1400	0.2031	no	M
GO:0008202	steroid metabolic process	5	1	0.1400	0.2031	no	M
GO:0008235	metalloexopeptidase activity	5	1	0.1400	0.2031	no	M
GO:0008825	cyclopropane-fatty-acyl-phospholipid synthase activity	5	1	0.1400	0.2031	no	M
GO:0016876	ligase activity, forming aminoacyl-tRNA and related compounds	5	1	0.1400	0.2031	no	M
GO:0031461	cullin-RING ubiquitin ligase complex	5	1	0.1400	0.2031	no	M
GO:0031625	ubiquitin protein ligase binding	5	1	0.1400	0.2031	no	M
GO:0033177	proton-transporting two-sector ATPase complex, proton-transporting domain	5	1	0.1400	0.2031	no	M
GO:0043039	tRNA aminoacylation	5	1	0.1400	0.2031	no	M
GO:0045261	proton-transporting ATP synthase complex, catalytic core F(1)	5	1	0.1400	0.2031	no	M
GO:0048038	quinone binding	5	1	0.1400	0.2031	no	M
GO:0004553	hydrolase activity, hydrolyzing O-glycosyl compounds	151	6	0.1430	0.1935	no	M
GO:0030246	carbohydrate binding	25	2	0.1447	0.1950	no	M
GO:0004386	helicase activity	84	4	0.1495	0.2006	no	M
GO:0003899	DNA-directed RNA polymerase activity	26	2	0.1518	0.2028	no	M
GO:0005507	copper ion binding	26	2	0.1518	0.2028	no	M
GO:0004609	phosphatidylserine decarboxylase activity	6	1	0.1627	0.2156	no	M
GO:0005083	small GTPase regulator activity	6	1	0.1627	0.2156	no	M

GO:0006265	DNA topological change	6	1	0.1627	0.2156	no	M
GO:0006284	base-excision repair	6	1	0.1627	0.2156	no	M
GO:0006779	porphyrin biosynthetic process	6	1	0.1627	0.2156	no	M
GO:0008610	lipid biosynthetic process	6	1	0.1627	0.2156	no	M
GO:0016272	prefoldin complex	6	1	0.1627	0.2156	no	M
GO:0005856	cytoskeleton	7	1	0.1838	0.2367	no	M
GO:0006306	DNA methylation	7	1	0.1838	0.2367	no	M
GO:0009073	aromatic amino acid family biosynthetic process	7	1	0.1838	0.2367	no	M
GO:0016651	oxidoreductase activity, acting on NADH or NADPH	7	1	0.1838	0.2367	no	M
GO:0019752	carboxylic acid metabolic process	7	1	0.1838	0.2367	no	M
GO:0019843	rRNA binding	7	1	0.1838	0.2367	no	M
GO:0030131	clathrin adaptor complex	7	1	0.1838	0.2367	no	M
GO:0033178	proton-transporting two-sector ATPase complex, catalytic domain	7	1	0.1838	0.2367	no	M
GO:0046912	transferase activity, transferring acyl groups, acyl groups converted into alkyl on transfer	7	1	0.1838	0.2367	no	M
GO:0008026	ATP-dependent helicase activity	64	3	0.1874	0.2330	no	M
GO:0050662	coenzyme binding	32	2	0.1909	0.2364	no	M
GO:0006396	RNA processing	33	2	0.1967	0.2427	no	M
GO:0003746	translation elongation factor activity	8	1	0.2033	0.2499	no	M
GO:0004129	cytochrome-c oxidase activity	8	1	0.2033	0.2499	no	M
GO:0015035	protein disulfide oxidoreductase activity	8	1	0.2033	0.2499	no	M

GO:0016455	RNA polymerase II transcription mediator activity	8	1	0.2033	0.2499	no	M
GO:0016592	mediator complex	8	1	0.2033	0.2499	no	M
GO:0046933	hydrogen ion transporting ATP synthase activity, rotational mechanism	8	1	0.2033	0.2499	no	M
GO:0006289	nucleotide-excision repair	9	1	0.2215	0.2661	no	M
GO:0006357	regulation of transcription from RNA polymerase II promoter	9	1	0.2215	0.2661	no	M
GO:0008299	isoprenoid biosynthetic process	9	1	0.2215	0.2661	no	M
GO:0016853	isomerase activity	9	1	0.2215	0.2661	no	M
GO:0044267	cellular protein metabolic process	9	1	0.2215	0.2661	no	M
GO:0003993	acid phosphatase activity	10	1	0.2382	0.2810	no	M
GO:0004499	flavin-containing monooxygenase activity	10	1	0.2382	0.2810	no	M
GO:0004571	mannosyl-oligosaccharide 1,2-alpha-mannosidase activity	10	1	0.2382	0.2810	no	M
GO:0006259	DNA metabolic process	10	1	0.2382	0.2810	no	M
GO:0008236	serine-type peptidase activity	45	2	0.2502	0.2908	no	M
GO:0045449	regulation of transcription	67	1	0.2517	0.2916	no	M
GO:0016887	ATPase activity	46	2	0.2532	0.2923	no	M
GO:0004568	chitinase activity	11	1	0.2537	0.2918	no	M
GO:0006032	chitin catabolic process	11	1	0.2537	0.2918	no	M
GO:0006342	chromatin silencing	11	1	0.2537	0.2918	no	M
GO:0006476	protein amino acid deacetylation	11	1	0.2537	0.2918	no	M
GO:0016747	transferase activity, transferring acyl groups other than amino-acyl groups	11	1	0.2537	0.2918	no	M

GO:0070403	NAD binding	11	1	0.2537	0.2918	no	M
GO:0003887	DNA-directed DNA polymerase activity	12	1	0.2680	0.3017	no	M
GO:0005789	endoplasmic reticulum membrane	12	1	0.2680	0.3017	no	M
GO:0007049	cell cycle	12	1	0.2680	0.3017	no	M
GO:0008138	protein tyrosine/serine/threonine phosphatase activity	12	1	0.2680	0.3017	no	M
GO:0046873	metal ion transmembrane transporter activity	12	1	0.2680	0.3017	no	M
GO:0004190	aspartic-type endopeptidase activity	69	2	0.2726	0.3016	no	M
GO:0000036	acyl carrier activity	13	1	0.2811	0.3099	no	M
GO:0004197	cysteine-type endopeptidase activity	13	1	0.2811	0.3099	no	M
GO:0006099	tricarboxylic acid cycle	13	1	0.2811	0.3099	no	M
GO:0008654	phospholipid biosynthetic process	13	1	0.2811	0.3099	no	M
GO:0005694	chromosome	14	1	0.2931	0.3188	no	M
GO:0006397	mRNA processing	14	1	0.2931	0.3188	no	M
GO:0008233	peptidase activity	14	1	0.2931	0.3188	no	M
GO:0016811	hydrolase activity, acting on carbon-nitrogen (but not peptide) bonds, in linear amides	14	1	0.2931	0.3188	no	M
GO:0005198	structural molecule activity	15	1	0.3040	0.3263	no	M
GO:0030001	metal ion transport	15	1	0.3040	0.3263	no	M
GO:0004601	peroxidase activity	16	1	0.3139	0.3347	no	M
GO:0043565	sequence-specific DNA binding	52	1	0.3179	0.3378	no	M
GO:0005089	Rho guanyl-nucleotide exchange factor activity	17	1	0.3229	0.3421	no	M
GO:0016311	dephosphorylation	17	1	0.3229	0.3421	no	M

GO:0016874	ligase activity	17	1	0.3229	0.3421	no	M
GO:0035023	regulation of Rho protein signal transduction	17	1	0.3229	0.3421	no	M
GO:0048037	cofactor binding	17	1	0.3229	0.3421	no	M
GO:0016829	lyase activity	18	1	0.3310	0.3450	no	M
GO:0006413	translational initiation	19	1	0.3383	0.3514	no	M
GO:0004252	serine-type endopeptidase activity	46	1	0.3416	0.3536	no	M
GO:0003743	translation initiation factor activity	20	1	0.3448	0.3558	no	M
GO:0005576	extracellular region	41	1	0.3580	0.3683	no	M
GO:0006470	protein amino acid dephosphorylation	23	1	0.3598	0.3690	no	M
GO:0006865	amino acid transport	23	1	0.3598	0.3690	no	M
GO:0015171	amino acid transmembrane transporter activity	23	1	0.3598	0.3690	no	M
GO:0008565	protein transporter activity	24	1	0.3635	0.3692	no	M
GO:0042626	ATPase activity, coupled to transmembrane movement of substances	24	1	0.3635	0.3692	no	M
GO:0006310	DNA recombination	27	1	0.3711	0.3745	no	M
GO:0009277	fungal-type cell wall	27	1	0.3711	0.3745	no	M
GO:0006511	ubiquitin-dependent protein catabolic process	35	1	0.3712	0.3724	no	M
GO:0005199	structural constituent of cell wall	29	1	0.3735	0.3735	no	M
GO:0005622	intracellular	381	24	0.0000	0.0000	q<0.01	P
GO:0015031	protein transport	57	9	0.0000	0.0000	q<0.01	P
GO:0005525	GTP binding	97	11	0.0000	0.0000	q<0.01	P
GO:0007264	small GTPase mediated signal transduction	42	7	0.0000	0.0002	q<0.01	P
GO:0006412	translation	149	12	0.0000	0.0002	q<0.01	P

GO:0005840	ribosome	105	10	0.0000	0.0003	q<0.01	P
GO:0003735	structural constituent of ribosome	107	10	0.0000	0.0003	q<0.01	P
GO:0007165	signal transduction	67	7	0.0001	0.0027	q<0.01	P
GO:0016020	membrane	312	15	0.0001	0.0036	q<0.01	P
GO:0003824	catalytic activity	791	27	0.0002	0.0034	q<0.01	P
GO:0005509	calcium ion binding	34	5	0.0002	0.0040	q<0.01	P
GO:0008483	transaminase activity	8	3	0.0002	0.0041	q<0.01	P
GO:0005743	mitochondrial inner membrane	23	4	0.0005	0.0078	q<0.01	P
GO:0006913	nucleocytoplasmic transport	13	3	0.0011	0.0166	0.01<q<0.05	P
GO:0004871	signal transducer activity	29	4	0.0011	0.0164	0.01<q<0.05	P
GO:0003924	GTPase activity	53	5	0.0015	0.0207	0.01<q<0.05	P
GO:0030170	pyridoxal phosphate binding	56	5	0.0019	0.0246	0.01<q<0.05	P
GO:0019001	guanyl nucleotide binding	16	3	0.0020	0.0240	0.01<q<0.05	P
GO:0006164	purine nucleotide biosynthetic process	5	2	0.0026	0.0294	0.01<q<0.05	P
GO:0055085	transmembrane transport	340	13	0.0027	0.0292	0.01<q<0.05	P
GO:0043169	cation binding	91	6	0.0031	0.0324	0.01<q<0.05	P
GO:0005215	transporter activity	65	5	0.0036	0.0355	0.01<q<0.05	P
GO:0016616	oxidoreductase activity, acting on the CH-OH group of donors, NAD or NADP as acceptor	20	3	0.0038	0.0359	0.01<q<0.05	P
GO:0019787	small conjugating protein ligase activity	20	3	0.0038	0.0359	0.01<q<0.05	P
GO:0043687	post-translational protein modification	20	3	0.0038	0.0359	0.01<q<0.05	P
GO:0051246	regulation of protein metabolic process	20	3	0.0038	0.0359	0.01<q<0.05	P
GO:0006355	regulation of transcription, DNA-dependent	127	7	0.0038	0.0309	0.01<q<0.05	P
GO:0007186	G-protein coupled receptor protein signaling	23	3	0.0056	0.0436	0.01<q<0.05	P

	pathway						
GO:0006810	transport	174	8	0.0058	0.0436	0.01<q<0.05	P
GO:0000166	nucleotide binding	214	9	0.0061	0.0446	0.01<q<0.05	P
GO:0006350	transcription	76	5	0.0067	0.0472	0.01<q<0.05	P
GO:0030145	manganese ion binding	8	2	0.0068	0.0465	0.01<q<0.05	P
GO:0055114	oxidation reduction	618	18	0.0071	0.0467	0.01<q<0.05	P
GO:0008152	metabolic process	683	19	0.0088	0.0561	no	P
GO:0051287	NAD or NADH binding	28	3	0.0095	0.0594	no	P
GO:0015078	hydrogen ion transmembrane transporter activity	10	2	0.0106	0.0643	no	P
GO:0016787	hydrolase activity	123	6	0.0118	0.0696	no	P
GO:0016021	integral to membrane	251	9	0.0145	0.0832	no	P
GO:0051539	4 iron, 4 sulfur cluster binding	12	2	0.0151	0.0842	no	P
GO:0003937	IMP cyclohydrolase activity	1	1	0.0164	0.0896	no	P
GO:0004019	adenylosuccinate synthase activity	1	1	0.0164	0.0896	no	P
GO:0004076	biotin synthase activity	1	1	0.0164	0.0896	no	P
GO:0004402	histone acetyltransferase activity	1	1	0.0164	0.0896	no	P
GO:0004420	hydroxymethylglutaryl-CoA reductase (NADPH) activity	1	1	0.0164	0.0896	no	P
GO:0004450	isocitrate dehydrogenase (NADP+) activity	1	1	0.0164	0.0896	no	P
GO:0004611	phosphoenolpyruvate carboxykinase activity	1	1	0.0164	0.0896	no	P
GO:0004612	phosphoenolpyruvate carboxykinase (ATP) activity	1	1	0.0164	0.0896	no	P
GO:0004643	phosphoribosylaminoimidazolecarboxamide	1	1	0.0164	0.0896	no	P

	formyltransferase activity						
GO:0004801	transaldolase activity	1	1	0.0164	0.0896	no	P
GO:0004829	threonine-tRNA ligase activity	1	1	0.0164	0.0896	no	P
GO:0005665	DNA-directed RNA polymerase II, core complex	1	1	0.0164	0.0896	no	P
GO:0006102	isocitrate metabolic process	1	1	0.0164	0.0896	no	P
GO:0006188	IMP biosynthetic process	1	1	0.0164	0.0896	no	P
GO:0006435	threonyl-tRNA aminoacylation	1	1	0.0164	0.0896	no	P
GO:0006452	translational frameshifting	1	1	0.0164	0.0896	no	P
GO:0006621	protein retention in ER lumen	1	1	0.0164	0.0896	no	P
GO:0007585	respiratory gaseous exchange	1	1	0.0164	0.0896	no	P
GO:0009102	biotin biosynthetic process	1	1	0.0164	0.0896	no	P
GO:0015936	coenzyme A metabolic process	1	1	0.0164	0.0896	no	P
GO:0017076	purine nucleotide binding	1	1	0.0164	0.0896	no	P
GO:0017136	NAD-dependent histone deacetylase activity	1	1	0.0164	0.0896	no	P
GO:0019478	D-amino acid catabolic process	1	1	0.0164	0.0896	no	P
GO:0030272	5-formyltetrahydrofolate cyclo-ligase activity	1	1	0.0164	0.0896	no	P
GO:0045901	positive regulation of translational elongation	1	1	0.0164	0.0896	no	P
GO:0045905	positive regulation of translational termination	1	1	0.0164	0.0896	no	P
GO:0046923	ER retention sequence binding	1	1	0.0164	0.0896	no	P
GO:0051186	cofactor metabolic process	1	1	0.0164	0.0896	no	P
GO:0004518	nuclease activity	13	2	0.0175	0.0561	no	P

GO:0005975	carbohydrate metabolic process	220	8	0.0185	0.0586	no	P
GO:0006886	intracellular protein transport	67	4	0.0195	0.0607	no	P
GO:0046872	metal ion binding	37	3	0.0195	0.0599	no	P
GO:0004519	endonuclease activity	14	2	0.0201	0.0608	no	P
GO:0004190	aspartic-type endopeptidase activity	69	4	0.0213	0.0636	no	P
GO:0016884	carbon-nitrogen ligase activity, with glutamine as amido-N-donor	17	2	0.0286	0.0842	no	P
GO:0004066	asparagine synthase (glutamine-hydrolyzing) activity	2	1	0.0323	0.0940	no	P
GO:0004348	glucosylceramidase activity	2	1	0.0323	0.0940	no	P
GO:0005764	lysosome	2	1	0.0323	0.0940	no	P
GO:0006529	asparagine biosynthetic process	2	1	0.0323	0.0940	no	P
GO:0007040	lysosome organization	2	1	0.0323	0.0940	no	P
GO:0008612	peptidyl-lysine modification to hypusine	2	1	0.0323	0.0940	no	P
GO:0030288	outer membrane-bounded periplasmic space	2	1	0.0323	0.0940	no	P
GO:0045263	proton-transporting ATP synthase complex, coupling factor F(o)	2	1	0.0323	0.0940	no	P
GO:0006281	DNA repair	46	3	0.0329	0.0864	no	P
GO:0020037	heme binding	257	8	0.0358	0.0930	no	P
GO:0004497	monooxygenase activity	258	8	0.0364	0.0933	no	P
GO:0006520	cellular amino acid metabolic process	20	2	0.0380	0.0964	no	P
GO:0016769	transferase activity, transferring nitrogenous groups	20	2	0.0380	0.0964	no	P
GO:0050661	NADP or NADPH binding	20	2	0.0380	0.0964	no	P

GO:0003677	DNA binding	319	9	0.0427	0.1047	no	P
GO:0015986	ATP synthesis coupled proton transport	22	2	0.0447	0.1083	no	P
GO:0022891	substrate-specific transmembrane transporter activity	22	2	0.0447	0.1083	no	P
GO:0000030	mannosyltransferase activity	3	1	0.0477	0.1130	no	P
GO:0004407	histone deacetylase activity	3	1	0.0477	0.1130	no	P
GO:0006366	transcription from RNA polymerase II promoter	3	1	0.0477	0.1130	no	P
GO:0006493	protein amino acid O-linked glycosylation	3	1	0.0477	0.1130	no	P
GO:0006665	sphingolipid metabolic process	3	1	0.0477	0.1130	no	P
GO:0006813	potassium ion transport	3	1	0.0477	0.1130	no	P
GO:0006915	apoptosis	3	1	0.0477	0.1130	no	P
GO:0015450	P-P-bond-hydrolysis-driven protein transmembrane transporter activity	3	1	0.0477	0.1130	no	P
GO:0016568	chromatin modification	3	1	0.0477	0.1130	no	P
GO:0016575	histone deacetylation	3	1	0.0477	0.1130	no	P
GO:0016813	hydrolase activity, acting on carbon-nitrogen (but not peptide) bonds, in linear amidines	3	1	0.0477	0.1130	no	P
GO:0033179	proton-transporting V-type ATPase, V0 domain	3	1	0.0477	0.1130	no	P
GO:0043022	ribosome binding	3	1	0.0477	0.1130	no	P
GO:0009058	biosynthetic process	57	3	0.0529	0.1099	no	P
GO:0000276	mitochondrial proton-transporting ATP synthase complex, coupling factor F(o)	4	1	0.0626	0.1286	no	P
GO:0003849	3-deoxy-7-phosphoheptulonate synthase activity	4	1	0.0626	0.1286	no	P

GO:0004784	superoxide dismutase activity	4	1	0.0626	0.1286	no	P
GO:0005740	mitochondrial envelope	4	1	0.0626	0.1286	no	P
GO:0006094	gluconeogenesis	4	1	0.0626	0.1286	no	P
GO:0006098	pentose-phosphate shunt	4	1	0.0626	0.1286	no	P
GO:0046907	intracellular transport	4	1	0.0626	0.1286	no	P
GO:0009055	electron carrier activity	250	7	0.0640	0.1235	no	P
GO:0005524	ATP binding	644	14	0.0646	0.1235	no	P
GO:0003700	transcription factor activity	105	4	0.0652	0.1236	no	P
GO:0008026	ATP-dependent helicase activity	64	3	0.0672	0.1262	no	P
GO:0005506	iron ion binding	260	7	0.0717	0.1337	no	P
GO:0050660	FAD binding	111	4	0.0740	0.1368	no	P
GO:0004721	phosphoprotein phosphatase activity	5	1	0.0769	0.1409	no	P
GO:0006801	superoxide metabolic process	5	1	0.0769	0.1409	no	P
GO:0006811	ion transport	5	1	0.0769	0.1409	no	P
GO:0008137	NADH dehydrogenase (ubiquinone) activity	5	1	0.0769	0.1409	no	P
GO:0008202	steroid metabolic process	5	1	0.0769	0.1409	no	P
GO:0016876	ligase activity, forming aminoacyl-tRNA and related compounds	5	1	0.0769	0.1409	no	P
GO:0033177	proton-transporting two-sector ATPase complex, proton-transporting domain	5	1	0.0769	0.1409	no	P
GO:0043039	tRNA aminoacylation	5	1	0.0769	0.1409	no	P
GO:0048038	quinone binding	5	1	0.0769	0.1409	no	P
GO:0050662	coenzyme binding	32	2	0.0814	0.1387	no	P
GO:0006396	RNA processing	33	2	0.0853	0.1441	no	P
GO:0004674	protein serine/threonine kinase activity	173	5	0.0903	0.1514	no	P

GO:0000155	two-component sensor activity	6	1	0.0908	0.1511	no	P
GO:0004673	protein histidine kinase activity	6	1	0.0908	0.1511	no	P
GO:0006284	base-excision repair	6	1	0.0908	0.1511	no	P
GO:0018106	peptidyl-histidine phosphorylation	6	1	0.0908	0.1511	no	P
GO:0005634	nucleus	345	8	0.0921	0.1487	no	P
GO:0006508	proteolysis	232	6	0.0946	0.1517	no	P
GO:0005488	binding	476	10	0.0962	0.1530	no	P
GO:0016491	oxidoreductase activity	482	10	0.0989	0.1562	no	P
GO:0005515	protein binding	363	8	0.1033	0.1620	no	P
GO:0009073	aromatic amino acid family biosynthetic process	7	1	0.1042	0.1622	no	P
GO:0009396	folic acid and derivative biosynthetic process	7	1	0.1042	0.1622	no	P
GO:0016651	oxidoreductase activity, acting on NADH or NADPH	7	1	0.1042	0.1622	no	P
GO:0016881	acid-amino acid ligase activity	7	1	0.1042	0.1622	no	P
GO:0019752	carboxylic acid metabolic process	7	1	0.1042	0.1622	no	P
GO:0046912	transferase activity, transferring acyl groups, acyl groups converted into alkyl on transfer	7	1	0.1042	0.1622	no	P
GO:0016192	vesicle-mediated transport	38	2	0.1046	0.1561	no	P
GO:0004386	helicase activity	84	3	0.1105	0.1639	no	P
GO:0005737	cytoplasm	138	4	0.1147	0.1690	no	P
GO:0000287	magnesium ion binding	41	2	0.1161	0.1698	no	P
GO:0000156	two-component response regulator activity	8	1	0.1171	0.1702	no	P

GO:0003746	translation elongation factor activity	8	1	0.1171	0.1702	no	P
GO:0005216	ion channel activity	8	1	0.1171	0.1702	no	P
GO:0015035	protein disulfide oxidoreductase activity	8	1	0.1171	0.1702	no	P
GO:0016455	RNA polymerase II transcription mediator activity	8	1	0.1171	0.1702	no	P
GO:0016592	mediator complex	8	1	0.1171	0.1702	no	P
GO:0008270	zinc ion binding	598	8	0.1179	0.1648	no	P
GO:0004672	protein kinase activity	202	5	0.1226	0.1703	no	P
GO:0000160	two-component signal transduction system (phosphorelay)	9	1	0.1296	0.1788	no	P
GO:0006289	nucleotide-excision repair	9	1	0.1296	0.1788	no	P
GO:0006357	regulation of transcription from RNA polymerase II promoter	9	1	0.1296	0.1788	no	P
GO:0008299	isoprenoid biosynthetic process	9	1	0.1296	0.1788	no	P
GO:0016614	oxidoreductase activity, acting on CH-OH group of donors	45	2	0.1312	0.1765	no	P
GO:0004553	hydrolase activity, hydrolyzing O-glycosyl compounds	151	4	0.1332	0.1782	no	P
GO:0004499	flavin-containing monooxygenase activity	10	1	0.1416	0.1883	no	P
GO:0004571	mannosyl-oligosaccharide 1,2-alpha-mannosidase activity	10	1	0.1416	0.1883	no	P
GO:0015935	small ribosomal subunit	10	1	0.1416	0.1883	no	P
GO:0008168	methyltransferase activity	49	2	0.1459	0.1904	no	P
GO:0003676	nucleic acid binding	461	7	0.1486	0.1929	no	P
GO:0003684	damaged DNA binding	11	1	0.1532	0.1977	no	P

GO:0004568	chitinase activity	11	1	0.1532	0.1977	no	P
GO:0006032	chitin catabolic process	11	1	0.1532	0.1977	no	P
GO:0006342	chromatin silencing	11	1	0.1532	0.1977	no	P
GO:0006476	protein amino acid deacetylation	11	1	0.1532	0.1977	no	P
GO:0016747	transferase activity, transferring acyl groups other than amino-acyl groups	11	1	0.1532	0.1977	no	P
GO:0070403	NAD binding	11	1	0.1532	0.1977	no	P
GO:0006468	protein amino acid phosphorylation	240	5	0.1564	0.1938	no	P
GO:0003887	DNA-directed DNA polymerase activity	12	1	0.1644	0.2025	no	P
GO:0016310	phosphorylation	12	1	0.1644	0.2025	no	P
GO:0016772	transferase activity, transferring phosphorus-containing groups	12	1	0.1644	0.2025	no	P
GO:0046873	metal ion transmembrane transporter activity	12	1	0.1644	0.2025	no	P
GO:0004175	endopeptidase activity	13	1	0.1752	0.2110	no	P
GO:0004177	aminopeptidase activity	13	1	0.1752	0.2110	no	P
GO:0004197	cysteine-type endopeptidase activity	13	1	0.1752	0.2110	no	P
GO:0003755	peptidyl-prolyl cis-trans isomerase activity	14	1	0.1856	0.2199	no	P
GO:0004298	threonine-type endopeptidase activity	14	1	0.1856	0.2199	no	P
GO:0005839	proteasome core complex	14	1	0.1856	0.2199	no	P
GO:0006397	mRNA processing	14	1	0.1856	0.2199	no	P
GO:0009987	cellular process	14	1	0.1856	0.2199	no	P
GO:0016811	hydrolase activity, acting on carbon-nitrogen (but not peptide) bonds, in linear amides	14	1	0.1856	0.2199	no	P
GO:0051603	proteolysis involved in cellular protein	14	1	0.1856	0.2199	no	P

	catabolic process						
GO:0005198	structural molecule activity	15	1	0.1956	0.2233	no	P
GO:0030001	metal ion transport	15	1	0.1956	0.2233	no	P
GO:0003779	actin binding	16	1	0.2052	0.2318	no	P
GO:0051536	iron-sulfur cluster binding	16	1	0.2052	0.2318	no	P
GO:0006464	protein modification process	17	1	0.2145	0.2398	no	P
GO:0016773	phosphotransferase activity, alcohol group as acceptor	19	1	0.2319	0.2580	no	P
GO:0045454	cell redox homeostasis	20	1	0.2401	0.2657	no	P
GO:0004185	serine-type carboxypeptidase activity	21	1	0.2480	0.2731	no	P
GO:0006334	nucleosome assembly	22	1	0.2556	0.2800	no	P
GO:0017111	nucleoside-triphosphatase activity	96	2	0.2603	0.2837	no	P
GO:0006865	amino acid transport	23	1	0.2628	0.2850	no	P
GO:0015171	amino acid transmembrane transporter activity	23	1	0.2628	0.2850	no	P
GO:0016788	hydrolase activity, acting on ester bonds	23	1	0.2628	0.2850	no	P
GO:0016791	phosphatase activity	23	1	0.2628	0.2850	no	P
GO:0003723	RNA binding	104	2	0.2678	0.2848	no	P
GO:0008565	protein transporter activity	24	1	0.2697	0.2854	no	P
GO:0016810	hydrolase activity, acting on carbon-nitrogen (but not peptide) bonds	24	1	0.2697	0.2854	no	P
GO:0006066	alcohol metabolic process	25	1	0.2763	0.2896	no	P
GO:0003899	DNA-directed RNA polymerase activity	26	1	0.2827	0.2949	no	P
GO:0031072	heat shock protein binding	29	1	0.3001	0.3115	no	P
GO:0004222	metalloendopeptidase activity	30	1	0.3053	0.3154	no	P

GO:0006418	tRNA aminoacylation for protein translation	31	1	0.3103	0.3191	no	P
GO:0004812	aminoacyl-tRNA ligase activity	32	1	0.3151	0.3225	no	P
GO:0010181	FMN binding	37	1	0.3354	0.3416	no	P
GO:0016887	ATPase activity	46	1	0.3593	0.3643	no	P
GO:0043565	sequence-specific DNA binding	52	1	0.3677	0.3711	no	P
GO:0045449	regulation of transcription	67	1	0.3695	0.3712	no	P
GO:0006457	protein folding	57	1	0.3710	0.3710	no	P
GO:0005622	intracellular	381	20	0.0000	0.0029	q<0.01	FB
GO:0016020	membrane	312	17	0.0000	0.0040	q<0.01	FB
GO:0015031	protein transport	57	7	0.0001	0.0045	q<0.01	FB
GO:0055114	oxidation reduction	618	25	0.0001	0.0045	q<0.01	FB
GO:0006412	translation	149	10	0.0003	0.0126	0.01<q<0.05	FB
GO:0006457	protein folding	57	6	0.0005	0.0175	0.01<q<0.05	FB
GO:0005840	ribosome	105	8	0.0005	0.0157	0.01<q<0.05	FB
GO:0003824	catalytic activity	791	27	0.0005	0.0153	0.01<q<0.05	FB
GO:0003735	structural constituent of ribosome	107	8	0.0005	0.0138	0.01<q<0.05	FB
GO:0007264	small GTPase mediated signal transduction	42	5	0.0008	0.0179	0.01<q<0.05	FB
GO:0055085	transmembrane transport	340	15	0.0008	0.0168	0.01<q<0.05	FB
GO:0006913	nucleocytoplasmic transport	13	3	0.0013	0.0260	0.01<q<0.05	FB
GO:0020037	heme binding	257	12	0.0016	0.0279	0.01<q<0.05	FB
GO:0004497	monooxygenase activity	258	12	0.0016	0.0267	0.01<q<0.05	FB
GO:0006350	transcription	76	6	0.0020	0.0301	0.01<q<0.05	FB
GO:0005509	calcium ion binding	34	4	0.0027	0.0391	0.01<q<0.05	FB
GO:0004674	protein serine/threonine kinase activity	173	9	0.0028	0.0386	0.01<q<0.05	FB
GO:0006810	transport	174	9	0.0029	0.0378	0.01<q<0.05	FB

GO:0051082	unfolded protein binding	35	4	0.0030	0.0365	0.01<q<0.05	FB
GO:0005524	ATP binding	644	21	0.0031	0.0358	0.01<q<0.05	FB
GO:0000166	nucleotide binding	214	10	0.0036	0.0391	0.01<q<0.05	FB
GO:0009055	electron carrier activity	250	11	0.0036	0.0376	0.01<q<0.05	FB
GO:0016192	vesicle-mediated transport	38	4	0.0040	0.0403	0.01<q<0.05	FB
GO:0005506	iron ion binding	260	11	0.0047	0.0449	0.01<q<0.05	FB
GO:0016616	oxidoreductase activity, acting on the CH-OH group of donors, NAD or NADP as acceptor	20	3	0.0047	0.0438	0.01<q<0.05	FB
GO:0005215	transporter activity	65	5	0.0050	0.0444	0.01<q<0.05	FB
GO:0006355	regulation of transcription, DNA-dependent	127	7	0.0058	0.0495	0.01<q<0.05	FB
GO:0005525	GTP binding	97	6	0.0061	0.0502	no	FB
GO:0016881	acid-amino acid ligase activity	7	2	0.0061	0.0486	no	FB
GO:0005488	binding	476	16	0.0064	0.0494	no	FB
GO:0005743	mitochondrial inner membrane	23	3	0.0070	0.0521	no	FB
GO:0004672	protein kinase activity	202	9	0.0071	0.0511	no	FB
GO:0003677	DNA binding	319	12	0.0074	0.0521	no	FB
GO:0005515	protein binding	363	13	0.0080	0.0541	no	FB
GO:0008483	transaminase activity	8	2	0.0080	0.0528	no	FB
GO:0016021	integral to membrane	251	10	0.0094	0.0605	no	FB
GO:0008152	metabolic process	683	20	0.0099	0.0617	no	FB
GO:0051287	NAD or NADH binding	28	3	0.0118	0.0719	no	FB
GO:0003924	GTPase activity	53	4	0.0122	0.0722	no	FB
GO:0015078	hydrogen ion transmembrane transporter activity	10	2	0.0124	0.0716	no	FB

GO:0031072	heat shock protein binding	29	3	0.0130	0.0730	no	FB
GO:0016491	oxidoreductase activity	482	15	0.0137	0.0751	no	FB
GO:0016787	hydrolase activity	123	6	0.0165	0.0885	no	FB
GO:0046873	metal ion transmembrane transporter activity	12	2	0.0175	0.0921	no	FB
GO:0051539	4 iron, 4 sulfur cluster binding	12	2	0.0175	0.0921	no	FB
GO:0006468	protein amino acid phosphorylation	240	9	0.0176	0.0882	no	FB
GO:0043169	cation binding	91	5	0.0177	0.0871	no	FB
GO:0003937	IMP cyclohydrolase activity	1	1	0.0179	0.0859	no	FB
GO:0004076	biotin synthase activity	1	1	0.0179	0.0859	no	FB
GO:0004357	glutamate-cysteine ligase activity	1	1	0.0179	0.0859	no	FB
GO:0004402	histone acetyltransferase activity	1	1	0.0179	0.0859	no	FB
GO:0004420	hydroxymethylglutaryl-CoA reductase (NADPH) activity	1	1	0.0179	0.0859	no	FB
GO:0004450	isocitrate dehydrogenase (NADP+) activity	1	1	0.0179	0.0859	no	FB
GO:0004611	phosphoenolpyruvate carboxykinase activity	1	1	0.0179	0.0859	no	FB
GO:0004612	phosphoenolpyruvate carboxykinase (ATP) activity	1	1	0.0179	0.0859	no	FB
GO:0004643	phosphoribosylaminoimidazolecarboxamide formyltransferase activity	1	1	0.0179	0.0859	no	FB
GO:0004651	polynucleotide 5'-phosphatase activity	1	1	0.0179	0.0859	no	FB
GO:0005094	Rho GDP-dissociation inhibitor activity	1	1	0.0179	0.0859	no	FB
GO:0005665	DNA-directed RNA polymerase II, core complex	1	1	0.0179	0.0859	no	FB

GO:0006102	isocitrate metabolic process	1	1	0.0179	0.0859	no	FB
GO:0006188	IMP biosynthetic process	1	1	0.0179	0.0859	no	FB
GO:0006452	translational frameshifting	1	1	0.0179	0.0859	no	FB
GO:0006621	protein retention in ER lumen	1	1	0.0179	0.0859	no	FB
GO:0007585	respiratory gaseous exchange	1	1	0.0179	0.0859	no	FB
GO:0009102	biotin biosynthetic process	1	1	0.0179	0.0859	no	FB
GO:0015936	coenzyme A metabolic process	1	1	0.0179	0.0859	no	FB
GO:0017076	purine nucleotide binding	1	1	0.0179	0.0859	no	FB
GO:0017136	NAD-dependent histone deacetylase activity	1	1	0.0179	0.0859	no	FB
GO:0019478	D-amino acid catabolic process	1	1	0.0179	0.0859	no	FB
GO:0030272	5-formyltetrahydrofolate cyclo-ligase activity	1	1	0.0179	0.0859	no	FB
GO:0031533	mRNA cap methyltransferase complex	1	1	0.0179	0.0859	no	FB
GO:0045901	positive regulation of translational elongation	1	1	0.0179	0.0859	no	FB
GO:0045905	positive regulation of translational termination	1	1	0.0179	0.0859	no	FB
GO:0046923	ER retention sequence binding	1	1	0.0179	0.0859	no	FB
GO:0048193	Golgi vesicle transport	1	1	0.0179	0.0859	no	FB
GO:0051186	cofactor metabolic process	1	1	0.0179	0.0859	no	FB
GO:0004177	aminopeptidase activity	13	2	0.0204	0.0611	no	FB
GO:0004518	nuclease activity	13	2	0.0204	0.0611	no	FB
GO:0004519	endonuclease activity	14	2	0.0233	0.0682	no	FB
GO:0009987	cellular process	14	2	0.0233	0.0682	no	FB
GO:0006886	intracellular protein transport	67	4	0.0249	0.0709	no	FB

GO:0007165	signal transduction	67	4	0.0249	0.0709	no	FB
GO:0005737	cytoplasm	138	6	0.0255	0.0710	no	FB
GO:0030001	metal ion transport	15	2	0.0264	0.0727	no	FB
GO:0003700	transcription factor activity	105	5	0.0287	0.0779	no	FB
GO:0003779	actin binding	16	2	0.0297	0.0797	no	FB
GO:0006464	protein modification process	17	2	0.0330	0.0877	no	FB
GO:0016884	carbon-nitrogen ligase activity, with glutamine as amido-N-donor	17	2	0.0330	0.0877	no	FB
GO:0004066	asparagine synthase (glutamine-hydrolyzing) activity	2	1	0.0351	0.0911	no	FB
GO:0004348	glucosylceramidase activity	2	1	0.0351	0.0911	no	FB
GO:0004816	asparagine-tRNA ligase activity	2	1	0.0351	0.0911	no	FB
GO:0005764	lysosome	2	1	0.0351	0.0911	no	FB
GO:0006421	asparaginyl-tRNA aminoacylation	2	1	0.0351	0.0911	no	FB
GO:0006529	asparagine biosynthetic process	2	1	0.0351	0.0911	no	FB
GO:0006750	glutathione biosynthetic process	2	1	0.0351	0.0911	no	FB
GO:0007040	lysosome organization	2	1	0.0351	0.0911	no	FB
GO:0008612	peptidyl-lysine modification to hypusine	2	1	0.0351	0.0911	no	FB
GO:0030288	outer membrane-bounded periplasmic space	2	1	0.0351	0.0911	no	FB
GO:0045263	proton-transporting ATP synthase complex, coupling factor F(o)	2	1	0.0351	0.0911	no	FB
GO:0006281	DNA repair	46	3	0.0397	0.0917	no	FB
GO:0019787	small conjugating protein ligase activity	20	2	0.0437	0.1001	no	FB
GO:0043687	post-translational protein modification	20	2	0.0437	0.1001	no	FB

GO:0050661	NADP or NADPH binding	20	2	0.0437	0.1001	no	FB
GO:0051246	regulation of protein metabolic process	20	2	0.0437	0.1001	no	FB
GO:0006334	nucleosome assembly	22	2	0.0513	0.1129	no	FB
GO:0015986	ATP synthesis coupled proton transport	22	2	0.0513	0.1129	no	FB
GO:0022891	substrate-specific transmembrane transporter activity	22	2	0.0513	0.1129	no	FB
GO:0000030	mannosyltransferase activity	3	1	0.0517	0.1105	no	FB
GO:0003918	DNA topoisomerase (ATP-hydrolyzing) activity	3	1	0.0517	0.1105	no	FB
GO:0004356	glutamate-ammonia ligase activity	3	1	0.0517	0.1105	no	FB
GO:0004407	histone deacetylase activity	3	1	0.0517	0.1105	no	FB
GO:0006366	transcription from RNA polymerase II promoter	3	1	0.0517	0.1105	no	FB
GO:0006370	mRNA capping	3	1	0.0517	0.1105	no	FB
GO:0006465	signal peptide processing	3	1	0.0517	0.1105	no	FB
GO:0006493	protein amino acid O-linked glycosylation	3	1	0.0517	0.1105	no	FB
GO:0006665	sphingolipid metabolic process	3	1	0.0517	0.1105	no	FB
GO:0006813	potassium ion transport	3	1	0.0517	0.1105	no	FB
GO:0015450	P-P-bond-hydrolysis-driven protein transmembrane transporter activity	3	1	0.0517	0.1105	no	FB
GO:0016568	chromatin modification	3	1	0.0517	0.1105	no	FB
GO:0016575	histone deacetylation	3	1	0.0517	0.1105	no	FB
GO:0016813	hydrolase activity, acting on carbon-nitrogen (but not peptide) bonds, in linear amidines	3	1	0.0517	0.1105	no	FB
GO:0033179	proton-transporting V-type ATPase, V0	3	1	0.0517	0.1105	no	FB

	domain						
GO:0043022	ribosome binding	3	1	0.0517	0.1105	no	FB
GO:0016791	phosphatase activity	23	2	0.0552	0.1028	no	FB
GO:0003899	DNA-directed RNA polymerase activity	26	2	0.0672	0.1242	no	FB
GO:0000276	mitochondrial proton-transporting ATP synthase complex, coupling factor F(o)	4	1	0.0677	0.1241	no	FB
GO:0003849	3-deoxy-7-phosphoheptulonate synthase activity	4	1	0.0677	0.1241	no	FB
GO:0004784	superoxide dismutase activity	4	1	0.0677	0.1241	no	FB
GO:0005740	mitochondrial envelope	4	1	0.0677	0.1241	no	FB
GO:0006094	gluconeogenesis	4	1	0.0677	0.1241	no	FB
GO:0008121	ubiquinol-cytochrome-c reductase activity	4	1	0.0677	0.1241	no	FB
GO:0046983	protein dimerization activity	27	2	0.0713	0.1248	no	FB
GO:0005634	nucleus	345	9	0.0746	0.1295	no	FB
GO:0004815	aspartate-tRNA ligase activity	5	1	0.0831	0.1433	no	FB
GO:0006164	purine nucleotide biosynthetic process	5	1	0.0831	0.1433	no	FB
GO:0006422	aspartyl-tRNA aminoacylation	5	1	0.0831	0.1433	no	FB
GO:0006801	superoxide metabolic process	5	1	0.0831	0.1433	no	FB
GO:0008137	NADH dehydrogenase (ubiquinone) activity	5	1	0.0831	0.1433	no	FB
GO:0008202	steroid metabolic process	5	1	0.0831	0.1433	no	FB
GO:0008235	metalloexopeptidase activity	5	1	0.0831	0.1433	no	FB
GO:0033177	proton-transporting two-sector ATPase complex, proton-transporting domain	5	1	0.0831	0.1433	no	FB
GO:0048038	quinone binding	5	1	0.0831	0.1433	no	FB
GO:0050660	FAD binding	111	4	0.0886	0.1430	no	FB

GO:0050662	coenzyme binding	32	2	0.0921	0.1478	no	FB
GO:0008270	zinc ion binding	598	8	0.0967	0.1541	no	FB
GO:0005083	small GTPase regulator activity	6	1	0.0979	0.1550	no	FB
GO:0006265	DNA topological change	6	1	0.0979	0.1550	no	FB
GO:0006284	base-excision repair	6	1	0.0979	0.1550	no	FB
GO:0006306	DNA methylation	7	1	0.1122	0.1740	no	FB
GO:0009073	aromatic amino acid family biosynthetic process	7	1	0.1122	0.1740	no	FB
GO:0009396	folic acid and derivative biosynthetic process	7	1	0.1122	0.1740	no	FB
GO:0016651	oxidoreductase activity, acting on NADH or NADPH	7	1	0.1122	0.1740	no	FB
GO:0019752	carboxylic acid metabolic process	7	1	0.1122	0.1740	no	FB
GO:0030131	clathrin adaptor complex	7	1	0.1122	0.1740	no	FB
GO:0046912	transferase activity, transferring acyl groups, acyl groups converted into alkyl on transfer	7	1	0.1122	0.1740	no	FB
GO:0006508	proteolysis	232	6	0.1126	0.1667	no	FB
GO:0010181	FMN binding	37	2	0.1131	0.1664	no	FB
GO:0046872	metal ion binding	37	2	0.1131	0.1664	no	FB
GO:0003746	translation elongation factor activity	8	1	0.1260	0.1830	no	FB
GO:0015035	protein disulfide oxidoreductase activity	8	1	0.1260	0.1830	no	FB
GO:0030145	manganese ion binding	8	1	0.1260	0.1830	no	FB
GO:0030163	protein catabolic process	8	1	0.1260	0.1830	no	FB
GO:0006289	nucleotide-excision repair	9	1	0.1392	0.1973	no	FB

GO:0008299	isoprenoid biosynthetic process	9	1	0.1392	0.1973	no	FB
GO:0044267	cellular protein metabolic process	9	1	0.1392	0.1973	no	FB
GO:0003676	nucleic acid binding	461	8	0.1423	0.1980	no	FB
GO:0016614	oxidoreductase activity, acting on CH-OH group of donors	45	2	0.1456	0.2014	no	FB
GO:0003993	acid phosphatase activity	10	1	0.1519	0.2089	no	FB
GO:0004499	flavin-containing monooxygenase activity	10	1	0.1519	0.2089	no	FB
GO:0004571	mannosyl-oligosaccharide 1,2-alpha-mannosidase activity	10	1	0.1519	0.2089	no	FB
GO:0006259	DNA metabolic process	10	1	0.1519	0.2089	no	FB
GO:0017111	nucleoside-triphosphatase activity	96	3	0.1526	0.2050	no	FB
GO:0005975	carbohydrate metabolic process	220	5	0.1558	0.2080	no	FB
GO:0003684	damaged DNA binding	11	1	0.1641	0.2179	no	FB
GO:0004568	chitinase activity	11	1	0.1641	0.2179	no	FB
GO:0006032	chitin catabolic process	11	1	0.1641	0.2179	no	FB
GO:0006342	chromatin silencing	11	1	0.1641	0.2179	no	FB
GO:0006476	protein amino acid deacetylation	11	1	0.1641	0.2179	no	FB
GO:0070403	NAD binding	11	1	0.1641	0.2179	no	FB
GO:0043565	sequence-specific DNA binding	52	2	0.1720	0.2207	no	FB
GO:0003887	DNA-directed DNA polymerase activity	12	1	0.1759	0.2245	no	FB
GO:0008138	protein tyrosine/serine/threonine phosphatase activity	12	1	0.1759	0.2245	no	FB
GO:0030170	pyridoxal phosphate binding	56	2	0.1859	0.2347	no	FB
GO:0004175	endopeptidase activity	13	1	0.1871	0.2349	no	FB
GO:0004197	cysteine-type endopeptidase activity	13	1	0.1871	0.2349	no	FB

GO:0009058	biosynthetic process	57	2	0.1893	0.2350	no	FB
GO:0003755	peptidyl-prolyl cis-trans isomerase activity	14	1	0.1979	0.2445	no	FB
GO:0004298	threonine-type endopeptidase activity	14	1	0.1979	0.2445	no	FB
GO:0005694	chromosome	14	1	0.1979	0.2445	no	FB
GO:0005839	proteasome core complex	14	1	0.1979	0.2445	no	FB
GO:0006397	mRNA processing	14	1	0.1979	0.2445	no	FB
GO:0006807	nitrogen compound metabolic process	14	1	0.1979	0.2445	no	FB
GO:0008233	peptidase activity	14	1	0.1979	0.2445	no	FB
GO:0016811	hydrolase activity, acting on carbon-nitrogen (but not peptide) bonds, in linear amides	14	1	0.1979	0.2445	no	FB
GO:0051603	proteolysis involved in cellular protein catabolic process	14	1	0.1979	0.2445	no	FB
GO:0008026	ATP-dependent helicase activity	64	2	0.2108	0.2485	no	FB
GO:0004601	peroxidase activity	16	1	0.2182	0.2559	no	FB
GO:0019001	guanyl nucleotide binding	16	1	0.2182	0.2559	no	FB
GO:0051536	iron-sulfur cluster binding	16	1	0.2182	0.2559	no	FB
GO:0004190	aspartic-type endopeptidase activity	69	2	0.2242	0.2590	no	FB
GO:0005089	Rho guanyl-nucleotide exchange factor activity	17	1	0.2277	0.2617	no	FB
GO:0016311	dephosphorylation	17	1	0.2277	0.2617	no	FB
GO:0035023	regulation of Rho protein signal transduction	17	1	0.2277	0.2617	no	FB
GO:0006979	response to oxidative stress	19	1	0.2455	0.2780	no	FB
GO:0004553	hydrolase activity, hydrolyzing O-glycosyl compounds	151	2	0.2471	0.2784	no	FB
GO:0000786	nucleosome	20	1	0.2539	0.2847	no	FB

GO:0006520	cellular amino acid metabolic process	20	1	0.2539	0.2847	no	FB
GO:0016769	transferase activity, transferring nitrogenous groups	20	1	0.2539	0.2847	no	FB
GO:0045454	cell redox homeostasis	20	1	0.2539	0.2847	no	FB
GO:0004386	helicase activity	84	2	0.2544	0.2799	no	FB
GO:0004185	serine-type carboxypeptidase activity	21	1	0.2618	0.2866	no	FB
GO:0006470	protein amino acid dephosphorylation	23	1	0.2766	0.3014	no	FB
GO:0006865	amino acid transport	23	1	0.2766	0.3014	no	FB
GO:0007186	G-protein coupled receptor protein signaling pathway	23	1	0.2766	0.3014	no	FB
GO:0015171	amino acid transmembrane transporter activity	23	1	0.2766	0.3014	no	FB
GO:0016788	hydrolase activity, acting on ester bonds	23	1	0.2766	0.3014	no	FB
GO:0008565	protein transporter activity	24	1	0.2835	0.3018	no	FB
GO:0016810	hydrolase activity, acting on carbon-nitrogen (but not peptide) bonds	24	1	0.2835	0.3018	no	FB
GO:0006066	alcohol metabolic process	25	1	0.2900	0.3059	no	FB
GO:0003723	RNA binding	104	1	0.2904	0.3049	no	FB
GO:0009277	fungal-type cell wall	27	1	0.3022	0.3159	no	FB
GO:0004871	signal transducer activity	29	1	0.3131	0.3258	no	FB
GO:0005199	structural constituent of cell wall	29	1	0.3131	0.3258	no	FB
GO:0006418	tRNA aminoacylation for protein translation	31	1	0.3229	0.3330	no	FB
GO:0004812	aminoacyl-tRNA ligase activity	32	1	0.3273	0.3361	no	FB
GO:0006396	RNA processing	33	1	0.3315	0.3389	no	FB
GO:0000287	magnesium ion binding	41	1	0.3567	0.3630	no	FB

GO:0008236	serine-type peptidase activity	45	1	0.3643	0.3691	no	FB
GO:0045449	regulation of transcription	67	1	0.3649	0.3681	no	FB
GO:0016887	ATPase activity	46	1	0.3657	0.3673	no	FB
GO:0008168	methyltransferase activity	49	1	0.3691	0.3691	no	FB

Table S10 Differential enrichment analysis of parental genes for KEGG pathways among the three developmental stages

ID	KEGG Annotation	No. of Genes	No. of Genes in this Category	Number of Parent Genes	No of Parent Genes in this category	P value	Q value (FDR)	Significance level	Stages
ko01100	Metabolic pathways	16127	675	514	59	0.0000	0.0000	q<0.01	M
ko01120	Microbial metabolism in diverse environments	16127	187	514	21	0.0000	0.0000	q<0.01	M
ko01110	Biosynthesis of secondary metabolites	16127	255	514	24	0.0000	0.0000	q<0.01	M
ko01130	Biosynthesis of antibiotics	16127	193	514	21	0.0000	0.0000	q<0.01	M
ko00040	Pentose and glucuronate interconversions	16127	8	514	5	0.0000	0.0000	q<0.01	M
ko05016	Huntington's disease	16127	73	514	12	0.0000	0.0000	q<0.01	M
ko01200	Carbon metabolism	16127	91	514	13	0.0000	0.0000	q<0.01	M
ko00190	Oxidative phosphorylation	16127	64	514	11	0.0000	0.0001	q<0.01	M
ko03010	Ribosome	16127	69	514	11	0.0000	0.0002	q<0.01	M
ko00620	Pyruvate metabolism	16127	69	514	10	0.0000	0.0002	q<0.01	M
ko05012	Parkinson's disease	16127	46	514	9	0.0000	0.0002	q<0.01	M
ko00710	Carbon fixation in photosynthetic organisms	16127	13	514	5	0.0000	0.0002	q<0.01	M
ko05010	Alzheimer's disease	16127	54	514	9	0.0000	0.0002	q<0.01	M
ko04392	Hippo signaling pathway	16127	5	514	3	0.0000	0.0004	q<0.01	M

	-multiple species								
ko00250	Alanine, aspartate and glutamate metabolism	16127	24	514	6	0.0000	0.0004	q<0.01	M
ko05168	Herpes simplex infection	16127	26	514	6	0.0001	0.0012	q<0.01	M
ko04152	AMPK signaling pathway	16127	20	514	5	0.0001	0.0015	q<0.01	M
ko05034	Alcoholism	16127	19	514	4	0.0002	0.0019	q<0.01	M
ko00330	Arginine and proline metabolism	16127	34	514	6	0.0003	0.0028	q<0.01	M
ko00010	Glycolysis / Gluconeogenesis	16127	40	514	6	0.0005	0.0045	q<0.01	M
ko04212	Longevity regulating pathway - worm	16127	20	514	4	0.0007	0.0067	q<0.01	M
ko01210	2-Oxocarboxylic acid metabolism	16127	33	514	5	0.0007	0.0064	q<0.01	M
ko04919	Thyroid hormone signaling pathway	16127	39	514	5	0.0009	0.0075	q<0.01	M
ko01230	Biosynthesis of amino acids	16127	95	514	9	0.0009	0.0072	q<0.01	M
ko04390	Hippo signaling pathway	16127	11	514	3	0.0010	0.0075	q<0.01	M
ko04975	Fat digestion and absorption	16127	2	514	2	0.0010	0.0074	q<0.01	M
ko04011	MAPK signaling pathway - yeast	16127	58	514	6	0.0015	0.0105	0.01<q<0.05	M
ko05169	Epstein-Barr virus infection	16127	67	514	7	0.0021	0.0141	0.01<q<0.05	M
ko04310	Wnt signaling pathway	16127	12	514	3	0.0022	0.0146	0.01<q<0.05	M
ko00270	Cysteine and methionine metabolism	16127	38	514	5	0.0024	0.0151	0.01<q<0.05	M
ko04710	Circadian rhythm	16127	4	514	2	0.0029	0.0181	0.01<q<0.05	M
ko04921	Oxytocin signaling pathway	16127	13	514	3	0.0031	0.0183	0.01<q<0.05	M

ko04120	Ubiquitin mediated proteolysis	16127	53	514	6	0.0033	0.0187	0.01<q<0.05	M
ko04141	Protein processing in endoplasmic reticulum	16127	81	514	7	0.0035	0.0195	0.01<q<0.05	M
ko04111	Cell cycle - yeast	16127	71	514	7	0.0038	0.0205	0.01<q<0.05	M
ko00650	Butanoate metabolism	16127	14	514	3	0.0041	0.0217	0.01<q<0.05	M
ko04932	Non-alcoholic fatty liver disease	16127	41	514	5	0.0056	0.0288	0.01<q<0.05	M
ko01220	Degradation of aromatic compounds	16127	31	514	4	0.0057	0.0285	0.01<q<0.05	M
ko00625	Chloroalkane and chloroalkene degradation	16127	5	514	2	0.0057	0.0278	0.01<q<0.05	M
ko04340	Hedgehog signaling pathway	16127	5	514	2	0.0057	0.0278	0.01<q<0.05	M
ko01524	Platinum drug resistance	16127	78	514	5	0.0062	0.0289	0.01<q<0.05	M
ko03430	Mismatch repair	16127	43	514	5	0.0069	0.0312	0.01<q<0.05	M
ko00220	Arginine biosynthesis	16127	16	514	3	0.0082	0.0363	0.01<q<0.05	M
ko00360	Phenylalanine metabolism	16127	17	514	3	0.0082	0.0363	0.01<q<0.05	M
ko03015	mRNA surveillance pathway	16127	43	514	5	0.0084	0.0353	0.01<q<0.05	M
ko00520	Amino sugar and nucleotide sugar metabolism	16127	52	514	4	0.0085	0.0353	0.01<q<0.05	M
ko04145	Phagosome	16127	28	514	4	0.0085	0.0353	0.01<q<0.05	M
ko00950	Isoquinoline alkaloid biosynthesis	16127	8	514	2	0.0092	0.0364	0.01<q<0.05	M
ko00960	Tropane, piperidine and pyridine alkaloid biosynthesis	16127	8	514	2	0.0092	0.0364	0.01<q<0.05	M
ko04341	Hedgehog signaling pathway	16127	6	514	2	0.0092	0.0364	0.01<q<0.05	M

	- fly								
ko04624	Toll and Imd signaling pathway	16127	7	514	2	0.0092	0.0364	0.01<q<0.05	M
ko00350	Tyrosine metabolism	16127	19	514	3	0.0100	0.0364	0.01<q<0.05	M
ko05202	Transcriptional misregulation in cancer	16127	32	514	3	0.0119	0.0425	0.01<q<0.05	M
ko00980	Metabolism of xenobiotics by cytochrome P450	16127	36	514	2	0.0134	0.0470	0.01<q<0.05	M
ko01040	Biosynthesis of unsaturated fatty acids	16127	14	514	2	0.0134	0.0470	0.01<q<0.05	M
ko04144	Endocytosis	16127	51	514	5	0.0139	0.0471	0.01<q<0.05	M
ko00480	Glutathione metabolism	16127	48	514	3	0.0139	0.0465	0.01<q<0.05	M
ko00510	N-Glycan biosynthesis	16127	23	514	3	0.0139	0.0465	0.01<q<0.05	M
ko00970	Aminoacyl-tRNA biosynthesis	16127	42	514	4	0.0149	0.0480	0.01<q<0.05	M
ko00680	Methane metabolism	16127	22	514	3	0.0162	0.0513	no	M
ko05110	Vibrio cholerae infection	16127	18	514	3	0.0162	0.0513	no	M
ko03050	Proteasome	16127	36	514	4	0.0180	0.0552	no	M
ko00053	Ascorbate and aldarate metabolism	16127	9	514	2	0.0181	0.0546	no	M
ko00982	Drug metabolism - cytochrome P450	16127	37	514	2	0.0181	0.0546	no	M
ko04013	MAPK signaling pathway - fly	16127	14	514	2	0.0181	0.0546	no	M
ko05204	Chemical carcinogenesis	16127	37	514	2	0.0181	0.0546	no	M
ko00626	Naphthalene degradation	16127	26	514	3	0.0212	0.0602	no	M
ko04721	Synaptic vesicle cycle	16127	21	514	3	0.0212	0.0602	no	M

ko05162	Measles	16127	10	514	2	0.0234	0.0644	no	M
ko03013	RNA transport	16127	100	514	7	0.0240	0.0653	no	M
ko00020	Citrate cycle	16127	26	514	3	0.0269	0.0719	no	M
ko04142	Lysosome	16127	63	514	3	0.0299	0.0790	no	M
ko00903	Limonene and pinene degradation	16127	2	514	1	0.0319	0.0830	no	M
ko00380	Tryptophan metabolism	16127	31	514	3	0.0331	0.0850	no	M
ko03040	Spliceosome	16127	87	514	6	0.0339	0.0858	no	M
ko04064	NF-kappa B signaling pathway	16127	12	514	2	0.0352	0.0881	no	M
ko04211	Longevity regulating pathway	16127	17	514	2	0.0352	0.0881	no	M
ko04260	Cardiac muscle contraction	16127	13	514	2	0.0352	0.0881	no	M
ko04966	Collecting duct acid secretion	16127	10	514	2	0.0352	0.0881	no	M
ko04114	Oocyte meiosis	16127	32	514	3	0.0364	0.0865	no	M
ko00513	Various types of N-glycan biosynthesis	16127	20	514	2	0.0417	0.0978	no	M
ko05323	Rheumatoid arthritis	16127	11	514	2	0.0417	0.0978	no	M
ko01212	Fatty acid metabolism	16127	21	514	2	0.0485	0.1109	no	M
ko00100	Steroid biosynthesis	16127	37	514	3	0.0509	0.1151	no	M
ko00071	Fatty acid degradation	16127	19	514	2	0.0554	0.1239	no	M
ko00310	Lysine degradation	16127	15	514	2	0.0554	0.1239	no	M
ko00561	Glycerolipid metabolism	16127	17	514	2	0.0554	0.1239	no	M
ko04146	Peroxisome	16127	43	514	3	0.0587	0.1267	no	M
ko00830	Retinol metabolism	16127	2	514	1	0.0617	0.1318	no	M
ko04964	Proximal tubule bicarbonate	16127	6	514	1	0.0617	0.1318	no	M

	reclamation								
ko04972	Pancreatic secretion	16127	5	514	1	0.0617	0.1318	no	M
ko00051	Fructose and mannose metabolism	16127	15	514	2	0.0626	0.1294	no	M
ko05131	Shigellosis	16127	17	514	2	0.0626	0.1294	no	M
ko04910	Insulin signaling pathway	16127	40	514	3	0.0627	0.1267	no	M
ko00030	Pentose phosphate pathway	16127	16	514	2	0.0700	0.1400	no	M
ko00640	Propanoate metabolism	16127	20	514	2	0.0700	0.1400	no	M
ko05120	Epithelial cell signaling in Helicobacter pylori infection	16127	15	514	2	0.0700	0.1400	no	M
ko05205	Proteoglycans in cancer	16127	18	514	2	0.0700	0.1400	no	M
ko00410	beta-Alanine metabolism	16127	19	514	2	0.0850	0.1631	no	M
ko04810	Regulation of actin cytoskeleton	16127	20	514	2	0.0850	0.1631	no	M
ko00072	Synthesis and degradation of ketone bodies	16127	4	514	1	0.0896	0.1686	no	M
ko00590	Arachidonic acid metabolism	16127	3	514	1	0.0896	0.1686	no	M
ko04614	Renin-angiotensin system	16127	5	514	1	0.0896	0.1686	no	M
ko04712	Circadian rhythm - plant	16127	4	514	1	0.0896	0.1686	no	M
ko04913	Ovarian steroidogenesis	16127	3	514	1	0.0896	0.1686	no	M
ko04920	Adipocytokine signaling pathway	16127	4	514	1	0.0896	0.1686	no	M
ko04923	Regulation of lipolysis in adipocytes	16127	3	514	1	0.0896	0.1686	no	M
ko05031	Amphetamine addiction	16127	3	514	1	0.0896	0.1686	no	M

ko05133	Pertussis	16127	5	514	1	0.0896	0.1686	no	M
ko05220	Chronic myeloid leukemia	16127	6	514	1	0.0896	0.1686	no	M
ko00621	Dioxin degradation	16127	24	514	2	0.0925	0.1584	no	M
ko00624	Polycyclic aromatic hydrocarbon degradation	16127	24	514	2	0.0925	0.1584	no	M
ko05206	MicroRNAs in cancer	16127	22	514	2	0.0925	0.1584	no	M
ko00630	Glyoxylate and dicarboxylate metabolism	16127	25	514	2	0.1001	0.1669	no	M
ko05152	Tuberculosis	16127	29	514	2	0.1001	0.1669	no	M
ko03060	Protein export	16127	20	514	2	0.1077	0.1765	no	M
ko04130	SNARE interactions in vesicular transport	16127	22	514	2	0.1077	0.1765	no	M
ko03410	Base excision repair	16127	22	514	2	0.1153	0.1856	no	M
ko00362	Benzoate degradation	16127	5	514	1	0.1157	0.1847	no	M
ko04270	Vascular smooth muscle contraction	16127	6	514	1	0.1157	0.1847	no	M
ko04330	Notch signaling pathway	16127	5	514	1	0.1157	0.1847	no	M
ko04350	TGF-beta signaling pathway	16127	6	514	1	0.1157	0.1847	no	M
ko04380	Osteoclast differentiation	16127	7	514	1	0.1157	0.1847	no	M
ko04720	Long-term potentiation	16127	6	514	1	0.1157	0.1847	no	M
ko04750	Inflammatory mediator regulation of TRP channels	16127	4	514	1	0.1157	0.1847	no	M
ko05140	Leishmaniasis	16127	6	514	1	0.1157	0.1847	no	M
ko00230	Purine metabolism	16127	79	514	4	0.1287	0.1926	no	M
ko00627	Aminobenzoate degradation	16127	25	514	2	0.1302	0.1933	no	M

ko04110	Cell cycle	16127	59	514	3	0.1345	0.1982	no	M
ko05200	Pathways in cancer	16127	28	514	2	0.1375	0.2010	no	M
ko00514	Other types of O-glycan biosynthesis	16127	6	514	1	0.1400	0.2031	no	M
ko00643	Styrene degradation	16127	5	514	1	0.1400	0.2031	no	M
ko04022	cGMP-PKG signaling pathway	16127	10	514	1	0.1400	0.2031	no	M
ko04391	Hippo signaling pathway - fly	16127	8	514	1	0.1400	0.2031	no	M
ko04668	TNF signaling pathway	16127	7	514	1	0.1400	0.2031	no	M
ko04670	Leukocyte transendothelial migration	16127	6	514	1	0.1400	0.2031	no	M
ko04933	AGE-RAGE signaling pathway in diabetic complications	16127	8	514	1	0.1400	0.2031	no	M
ko04976	Bile secretion	16127	8	514	1	0.1400	0.2031	no	M
ko05222	Small cell lung cancer	16127	5	514	1	0.1400	0.2031	no	M
ko03020	RNA polymerase	16127	29	514	2	0.1587	0.2154	no	M
ko04360	Axon guidance	16127	8	514	1	0.1627	0.2192	no	M
ko04520	Adherens junction	16127	9	514	1	0.1627	0.2192	no	M
ko04611	Platelet activation	16127	8	514	1	0.1627	0.2192	no	M
ko04723	Retrograde endocannabinoid signaling	16127	8	514	1	0.1627	0.2192	no	M
ko04931	Insulin resistance	16127	7	514	1	0.1627	0.2192	no	M
ko04510	Focal adhesion	16127	10	514	1	0.1838	0.2391	no	M
ko05322	Systemic lupus erythematosus	16127	13	514	1	0.1838	0.2391	no	M

ko05166	HTLV-I infection	16127	38	514	2	0.1967	0.2525	no	M
ko03008	Ribosome biogenesis in eukaryotes	16127	71	514	3	0.1981	0.2526	no	M
ko01523	Antifolate resistance	16127	8	514	1	0.2033	0.2575	no	M
ko02024	Quorum sensing	16127	9	514	1	0.2033	0.2575	no	M
ko03320	PPAR signaling pathway	16127	15	514	1	0.2033	0.2575	no	M
ko04024	cAMP signaling pathway	16127	17	514	1	0.2033	0.2575	no	M
ko04072	Phospholipase D signaling pathway	16127	10	514	1	0.2033	0.2575	no	M
ko04122	Sulfur relay system	16127	8	514	1	0.2033	0.2575	no	M
ko04261	Adrenergic signaling in cardiomyocytes	16127	13	514	1	0.2033	0.2575	no	M
ko04370	VEGF signaling pathway	16127	10	514	1	0.2033	0.2575	no	M
ko04728	Dopaminergic synapse	16127	8	514	1	0.2033	0.2575	no	M
ko04961	Endocrine and other factor-regulated calcium reabsorption	16127	11	514	1	0.2033	0.2575	no	M
ko03420	Nucleotide excision repair	16127	38	514	2	0.2180	0.2589	no	M
ko00720	Carbon fixation pathways in prokaryotes	16127	14	514	1	0.2215	0.2614	no	M
ko04722	Neurotrophin signaling pathway	16127	15	514	1	0.2215	0.2614	no	M
ko00300	Lysine biosynthesis	16127	13	514	1	0.2382	0.2777	no	M
ko00910	Nitrogen metabolism	16127	13	514	1	0.2382	0.2777	no	M
ko04530	Tight junction	16127	11	514	1	0.2382	0.2777	no	M
ko04922	Glucagon signaling pathway	16127	10	514	1	0.2382	0.2777	no	M

ko05100	Bacterial invasion of epithelial cells	16127	10	514	1	0.2382	0.2777	no	M
ko04113	Meiosis - yeast	16127	50	514	2	0.2502	0.2829	no	M
ko00600	Sphingolipid metabolism	16127	15	514	1	0.2537	0.2853	no	M
ko02020	Two-component system	16127	14	514	1	0.2537	0.2853	no	M
ko04962	Vasopressin-regulated water reabsorption	16127	11	514	1	0.2537	0.2853	no	M
ko03018	RNA degradation	16127	51	514	2	0.2611	0.2884	no	M
ko00670	One carbon pool by folate	16127	12	514	1	0.2680	0.2943	no	M
ko05230	Central carbon metabolism in cancer	16127	15	514	1	0.2680	0.2943	no	M
ko00240	Pyrimidine metabolism	16127	63	514	2	0.2753	0.2988	no	M
ko00920	Sulfur metabolism	16127	14	514	1	0.2811	0.3034	no	M
ko04066	HIF-1 signaling pathway	16127	20	514	1	0.2811	0.3034	no	M
ko04068	FoxO signaling pathway	16127	22	514	1	0.2811	0.3034	no	M
ko04071	Sphingolipid signaling pathway	16127	25	514	1	0.2811	0.3034	no	M
ko04623	Cytosolic DNA-sensing pathway	16127	13	514	1	0.2811	0.3034	no	M
ko04912	GnRH signaling pathway	16127	18	514	1	0.2931	0.3076	no	M
ko00052	Galactose metabolism	16127	23	514	1	0.3139	0.3277	no	M
ko00860	Porphyrin and chlorophyll metabolism	16127	17	514	1	0.3139	0.3277	no	M
ko00900	Terpenoid backbone biosynthesis	16127	17	514	1	0.3139	0.3277	no	M
ko00340	Histidine metabolism	16127	19	514	1	0.3229	0.3317	no	M

ko04150	mTOR signaling pathway	16127	24	514	1	0.3383	0.3456	no	M
ko00564	Glycerophospholipid metabolism	16127	27	514	1	0.3505	0.3561	no	M
ko00500	Starch and sucrose metabolism	16127	40	514	1	0.3555	0.3592	no	M
ko03460	Fanconi anemia pathway	16127	23	514	1	0.3555	0.3592	no	M
ko05203	Viral carcinogenesis	16127	36	514	1	0.3725	0.3725	no	M
ko01100	Metabolic pathways	16127	675	265	29	0.0000	0.0000	q<0.01	P
ko00330	Arginine and proline metabolism	16127	34	265	6	0.0000	0.0005	q<0.01	P
ko00250	Alanine, aspartate and glutamate metabolism	16127	24	265	5	0.0000	0.0009	q<0.01	P
ko01130	Biosynthesis of antibiotics	16127	193	265	12	0.0000	0.0009	q<0.01	P
ko04624	Toll and Imd signaling pathway	16127	7	265	3	0.0000	0.0012	q<0.01	P
ko01120	Microbial metabolism in diverse environments	16127	187	265	11	0.0000	0.0012	q<0.01	P
ko03010	Ribosome	16127	69	265	7	0.0001	0.0022	q<0.01	P
ko00040	Pentose and glucuronate interconversions	16127	8	265	3	0.0001	0.0026	q<0.01	P
ko04390	Hippo signaling pathway	16127	11	265	3	0.0001	0.0026	q<0.01	P
ko01110	Biosynthesis of secondary metabolites	16127	255	265	12	0.0002	0.0032	q<0.01	P
ko04975	Fat digestion and absorption	16127	2	265	2	0.0003	0.0035	q<0.01	P
ko01210	2-Oxocarboxylic acid metabolism	16127	33	265	4	0.0005	0.0065	q<0.01	P

ko05016	Huntington's disease	16127	73	265	6	0.0006	0.0065	q<0.01	P
ko00710	Carbon fixation in photosynthetic organisms	16127	13	265	3	0.0006	0.0065	q<0.01	P
ko04919	Thyroid hormone signaling pathway	16127	39	265	4	0.0006	0.0061	q<0.01	P
ko04392	Hippo signaling pathway -multiple species	16127	5	265	2	0.0008	0.0071	q<0.01	P
ko04120	Ubiquitin mediated proteolysis	16127	53	265	5	0.0010	0.0083	q<0.01	P
ko00360	Phenylalanine metabolism	16127	17	265	3	0.0013	0.0106	0.01<q<0.05	P
ko01200	Carbon metabolism	16127	91	265	6	0.0014	0.0105	0.01<q<0.05	P
ko01230	Biosynthesis of amino acids	16127	95	265	6	0.0019	0.0136	0.01<q<0.05	P
ko00510	N-Glycan biosynthesis	16127	23	265	3	0.0024	0.0162	0.01<q<0.05	P
ko00950	Isoquinoline alkaloid biosynthesis	16127	8	265	2	0.0026	0.0166	0.01<q<0.05	P
ko00960	Tropane, piperidine and pyridine alkaloid biosynthesis	16127	8	265	2	0.0026	0.0166	0.01<q<0.05	P
ko05110	Vibrio cholerae infection	16127	18	265	3	0.0028	0.0167	0.01<q<0.05	P
ko00190	Oxidative phosphorylation	16127	64	265	5	0.0034	0.0193	0.01<q<0.05	P
ko04011	MAPK signaling pathway - yeast	16127	58	265	4	0.0039	0.0217	0.01<q<0.05	P
ko03015	mRNA surveillance pathway	16127	43	265	4	0.0043	0.0227	0.01<q<0.05	P
ko00053	Ascorbate and aldarate metabolism	16127	9	265	2	0.0052	0.0266	0.01<q<0.05	P
ko04013	MAPK signaling pathway - fly	16127	14	265	2	0.0052	0.0266	0.01<q<0.05	P
ko04144	Endocytosis	16127	51	265	4	0.0068	0.0322	0.01<q<0.05	P

ko05010	Alzheimer's disease	16127	54	265	4	0.0068	0.0322	0.01<q<0.05	P
ko04072	Phospholipase D signaling pathway	16127	10	265	2	0.0068	0.0305	0.01<q<0.05	P
ko00520	Amino sugar and nucleotide sugar metabolism	16127	52	265	3	0.0087	0.0375	0.01<q<0.05	P
ko05034	Alcoholism	16127	19	265	2	0.0106	0.0446	0.01<q<0.05	P
ko00620	Pyruvate metabolism	16127	69	265	4	0.0112	0.0457	0.01<q<0.05	P
ko04146	Peroxisome	16127	43	265	3	0.0125	0.0495	0.01<q<0.05	P
ko00513	Various types of N-glycan biosynthesis	16127	20	265	2	0.0128	0.0493	0.01<q<0.05	P
ko05169	Epstein-Barr virus infection	16127	67	265	4	0.0132	0.0497	0.01<q<0.05	P
ko04910	Insulin signaling pathway	16127	40	265	3	0.0135	0.0496	0.01<q<0.05	P
ko00670	One carbon pool by folate	16127	12	265	2	0.0151	0.0538	no	P
ko00903	Limonene and pinene degradation	16127	2	265	1	0.0164	0.0573	no	P
ko00561	Glycerolipid metabolism	16127	17	265	2	0.0175	0.0596	no	P
ko04111	Cell cycle - yeast	16127	71	265	4	0.0186	0.0620	no	P
ko00220	Arginine biosynthesis	16127	16	265	2	0.0201	0.0653	no	P
ko00350	Tyrosine metabolism	16127	19	265	2	0.0228	0.0725	no	P
ko05205	Proteoglycans in cancer	16127	18	265	2	0.0228	0.0725	no	P
ko03430	Mismatch repair	16127	43	265	3	0.0236	0.0719	no	P
ko05202	Transcriptional misregulation in cancer	16127	32	265	2	0.0256	0.0764	no	P
ko04152	AMPK signaling pathway	16127	20	265	2	0.0286	0.0834	no	P
ko04810	Regulation of actin	16127	20	265	2	0.0286	0.0834	no	P

	cytoskeleton								
ko04972	Pancreatic secretion	16127	5	265	1	0.0323	0.0906	no	P
ko05012	Parkinson's disease	16127	46	265	3	0.0329	0.0905	no	P
ko04721	Synaptic vesicle cycle	16127	21	265	2	0.0380	0.1026	no	P
ko03410	Base excision repair	16127	22	265	2	0.0413	0.1094	no	P
ko00020	Citrate cycle	16127	26	265	2	0.0447	0.1163	no	P
ko00500	Starch and sucrose metabolism	16127	40	265	2	0.0447	0.1163	no	P
ko04710	Circadian rhythm	16127	4	265	1	0.0477	0.1197	no	P
ko05031	Amphetamine addiction	16127	3	265	1	0.0477	0.1197	no	P
ko05133	Pertussis	16127	5	265	1	0.0477	0.1197	no	P
ko05220	Chronic myeloid leukemia	16127	6	265	1	0.0477	0.1197	no	P
ko00627	Aminobenzoate degradation	16127	25	265	2	0.0482	0.1129	no	P
ko04142	Lysosome	16127	63	265	2	0.0482	0.1129	no	P
ko00380	Tryptophan metabolism	16127	31	265	2	0.0517	0.1173	no	P
ko01220	Degradation of aromatic compounds	16127	31	265	2	0.0517	0.1173	no	P
ko04114	Oocyte meiosis	16127	32	265	2	0.0553	0.1216	no	P
ko05168	Herpes simplex infection	16127	26	265	2	0.0553	0.1216	no	P
ko00362	Benzoate degradation	16127	5	265	1	0.0626	0.1335	no	P
ko00625	Chloroalkane and chloroalkene degradation	16127	5	265	1	0.0626	0.1335	no	P
ko04270	Vascular smooth muscle contraction	16127	6	265	1	0.0626	0.1335	no	P
ko04330	Notch signaling pathway	16127	5	265	1	0.0626	0.1335	no	P

ko04340	Hedgehog signaling pathway	16127	5	265	1	0.0626	0.1335	no	P
ko04720	Long-term potentiation	16127	6	265	1	0.0626	0.1335	no	P
ko04750	Inflammatory mediator regulation of TRP channels	16127	4	265	1	0.0626	0.1335	no	P
ko04145	Phagosome	16127	28	265	2	0.0626	0.1209	no	P
ko04141	Protein processing in endoplasmic reticulum	16127	81	265	3	0.0693	0.1321	no	P
ko00100	Steroid biosynthesis	16127	37	265	2	0.0700	0.1318	no	P
ko00514	Other types of O-glycan biosynthesis	16127	6	265	1	0.0769	0.1428	no	P
ko00643	Styrene degradation	16127	5	265	1	0.0769	0.1428	no	P
ko04022	cGMP-PKG signaling pathway	16127	10	265	1	0.0769	0.1428	no	P
ko04341	Hedgehog signaling pathway - fly	16127	6	265	1	0.0769	0.1428	no	P
ko04391	Hippo signaling pathway - fly	16127	8	265	1	0.0769	0.1428	no	P
ko04976	Bile secretion	16127	8	265	1	0.0769	0.1428	no	P
ko00270	Cysteine and methionine metabolism	16127	38	265	2	0.0776	0.1337	no	P
ko00010	Glycolysis / Gluconeogenesis	16127	40	265	2	0.0853	0.1452	no	P
ko05166	HTLV-I infection	16127	38	265	2	0.0853	0.1452	no	P
ko03050	Proteasome	16127	36	265	2	0.0891	0.1482	no	P
ko04360	Axon guidance	16127	8	265	1	0.0908	0.1492	no	P
ko04611	Platelet activation	16127	8	265	1	0.0908	0.1492	no	P
ko04931	Insulin resistance	16127	7	265	1	0.0908	0.1492	no	P

ko00230	Purine metabolism	16127	79	265	3	0.0930	0.1478	no	P
ko04510	Focal adhesion	16127	10	265	1	0.1042	0.1637	no	P
ko04932	Non-alcoholic fatty liver disease	16127	41	265	2	0.1046	0.1625	no	P
ko01523	Antifolate resistance	16127	8	265	1	0.1171	0.1801	no	P
ko02024	Quorum sensing	16127	9	265	1	0.1171	0.1801	no	P
ko04024	cAMP signaling pathway	16127	17	265	1	0.1171	0.1801	no	P
ko04122	Sulfur relay system	16127	8	265	1	0.1171	0.1801	no	P
ko04261	Adrenergic signaling in cardiomyocytes	16127	13	265	1	0.1171	0.1801	no	P
ko04728	Dopaminergic synapse	16127	8	265	1	0.1171	0.1801	no	P
ko04961	Endocrine and other factor-regulated calcium reabsorption	16127	11	265	1	0.1171	0.1801	no	P
ko05162	Measles	16127	10	265	1	0.1171	0.1801	no	P
ko00720	Carbon fixation pathways in prokaryotes	16127	14	265	1	0.1296	0.1835	no	P
ko04310	Wnt signaling pathway	16127	12	265	1	0.1296	0.1835	no	P
ko03013	RNA transport	16127	100	265	3	0.1401	0.1945	no	P
ko00300	Lysine biosynthesis	16127	13	265	1	0.1416	0.1947	no	P
ko04211	Longevity regulating pathway	16127	17	265	1	0.1416	0.1947	no	P
ko04921	Oxytocin signaling pathway	16127	13	265	1	0.1416	0.1947	no	P
ko04966	Collecting duct acid secretion	16127	10	265	1	0.1416	0.1947	no	P
ko05100	Bacterial invasion of epithelial cells	16127	10	265	1	0.1416	0.1947	no	P

ko04110	Cell cycle	16127	59	265	2	0.1459	0.1914	no	P
ko00650	Butanoate metabolism	16127	14	265	1	0.1532	0.1992	no	P
ko05323	Rheumatoid arthritis	16127	11	265	1	0.1532	0.1992	no	P
ko00071	Fatty acid degradation	16127	19	265	1	0.1752	0.2237	no	P
ko00310	Lysine degradation	16127	15	265	1	0.1752	0.2237	no	P
ko04014	Ras signaling pathway	16127	15	265	1	0.1752	0.2237	no	P
ko04068	FoxO signaling pathway	16127	22	265	1	0.1752	0.2237	no	P
ko04212	Longevity regulating pathway - worm	16127	20	265	1	0.1856	0.2288	no	P
ko05131	Shigellosis	16127	17	265	1	0.1856	0.2288	no	P
ko00030	Pentose phosphate pathway	16127	16	265	1	0.1956	0.2370	no	P
ko05120	Epithelial cell signaling in Helicobacter pylori infection	16127	15	265	1	0.1956	0.2370	no	P
ko00900	Terpenoid backbone biosynthesis	16127	17	265	1	0.2052	0.2446	no	P
ko03008	Ribosome biogenesis in eukaryotes	16127	71	265	2	0.2065	0.2441	no	P
ko00340	Histidine metabolism	16127	19	265	1	0.2145	0.2514	no	P
ko00410	beta-Alanine metabolism	16127	19	265	1	0.2145	0.2514	no	P
ko00480	Glutathione metabolism	16127	48	265	1	0.2145	0.2514	no	P
ko00621	Dioxin degradation	16127	24	265	1	0.2234	0.2555	no	P
ko00624	Polycyclic aromatic hydrocarbon degradation	16127	24	265	1	0.2234	0.2555	no	P
ko00680	Methane metabolism	16127	22	265	1	0.2234	0.2555	no	P
ko00630	Glyoxylate and dicarboxylate	16127	25	265	1	0.2319	0.2591	no	P

	metabolism								
ko05152	Tuberculosis	16127	29	265	1	0.2319	0.2591	no	P
ko05164	Influenza A	16127	23	265	1	0.2319	0.2591	no	P
ko00626	Naphthalene degradation	16127	26	265	1	0.2401	0.2621	no	P
ko03060	Protein export	16127	20	265	1	0.2401	0.2621	no	P
ko04130	SNARE interactions in vesicular transport	16127	22	265	1	0.2401	0.2621	no	P
ko03040	Spliceosome	16127	87	265	2	0.2427	0.2589	no	P
ko05200	Pathways in cancer	16127	28	265	1	0.2697	0.2857	no	P
ko03020	RNA polymerase	16127	29	265	1	0.2888	0.3036	no	P
ko05203	Viral carcinogenesis	16127	36	265	1	0.2945	0.3074	no	P
ko00970	Aminoacyl-tRNA biosynthesis	16127	42	265	1	0.3151	0.3265	no	P
ko03420	Nucleotide excision repair	16127	38	265	1	0.3354	0.3450	no	P
ko01524	Platinum drug resistance	16127	78	265	1	0.3420	0.3493	no	P
ko04113	Meiosis - yeast	16127	50	265	1	0.3573	0.3624	no	P
ko03018	RNA degradation	16127	51	265	1	0.3642	0.3667	no	P
ko00240	Pyrimidine metabolism	16127	63	265	1	0.3716	0.3716	no	P
ko01100	Metabolic pathways	16127	675	288	29	0.0000	0.0001	q<0.01	FB
ko05169	Epstein-Barr virus infection	16127	67	288	7	0.0001	0.0057	q<0.01	FB
ko05016	Huntington's disease	16127	73	288	7	0.0001	0.0069	q<0.01	FB
ko00330	Arginine and proline metabolism	16127	34	288	5	0.0002	0.0062	q<0.01	FB
ko00040	Pentose and glucuronate interconversions	16127	8	288	3	0.0002	0.0057	q<0.01	FB
ko04390	Hippo signaling pathway	16127	11	288	3	0.0002	0.0057	q<0.01	FB

ko05034	Alcoholism	16127	19	288	3	0.0006	0.0131	0.01<q<0.05	FB
ko03015	mRNA surveillance pathway	16127	43	288	5	0.0008	0.0149	0.01<q<0.05	FB
ko01130	Biosynthesis of antibiotics	16127	193	288	10	0.0009	0.0148	0.01<q<0.05	FB
ko04919	Thyroid hormone signaling pathway	16127	39	288	4	0.0009	0.0134	0.01<q<0.05	FB
ko05168	Herpes simplex infection	16127	26	288	4	0.0009	0.0134	0.01<q<0.05	FB
ko04392	Hippo signaling pathway -multiple species	16127	5	288	2	0.0009	0.0120	0.01<q<0.05	FB
ko03010	Ribosome	16127	69	288	6	0.0011	0.0133	0.01<q<0.05	FB
ko04120	Ubiquitin mediated proteolysis	16127	53	288	5	0.0014	0.0154	0.01<q<0.05	FB
ko04212	Longevity regulating pathway - worm	16127	20	288	3	0.0017	0.0173	0.01<q<0.05	FB
ko05202	Transcriptional misregulation in cancer	16127	32	288	3	0.0025	0.0241	0.01<q<0.05	FB
ko00510	N-Glycan biosynthesis	16127	23	288	3	0.0030	0.0270	0.01<q<0.05	FB
ko04624	Toll and Imd signaling pathway	16127	7	288	2	0.0030	0.0258	0.01<q<0.05	FB
ko05110	Vibrio cholerae infection	16127	18	288	3	0.0035	0.0285	0.01<q<0.05	FB
ko01110	Biosynthesis of secondary metabolites	16127	255	288	10	0.0044	0.0336	0.01<q<0.05	FB
ko00190	Oxidative phosphorylation	16127	64	288	5	0.0047	0.0345	0.01<q<0.05	FB
ko04011	MAPK signaling pathway - yeast	16127	58	288	4	0.0052	0.0366	0.01<q<0.05	FB
ko01120	Microbial metabolism in diverse environments	16127	187	288	8	0.0054	0.0361	0.01<q<0.05	FB

ko00053	Ascorbate and aldarate metabolism	16127	9	288	2	0.0061	0.0392	0.01<q<0.05	FB
ko04013	MAPK signaling pathway - fly	16127	14	288	2	0.0061	0.0392	0.01<q<0.05	FB
ko04142	Lysosome	16127	63	288	3	0.0070	0.0414	0.01<q<0.05	FB
ko01210	2-Oxocarboxylic acid metabolism	16127	33	288	3	0.0078	0.0447	0.01<q<0.05	FB
ko04310	Wnt signaling pathway	16127	12	288	2	0.0101	0.0555	no	FB
ko00520	Amino sugar and nucleotide sugar metabolism	16127	52	288	3	0.0107	0.0571	no	FB
ko04064	NF-kappa B signaling pathway	16127	12	288	2	0.0124	0.0636	no	FB
ko04921	Oxytocin signaling pathway	16127	13	288	2	0.0124	0.0636	no	FB
ko00513	Various types of N-glycan biosynthesis	16127	20	288	2	0.0149	0.0716	no	FB
ko00710	Carbon fixation in photosynthetic organisms	16127	13	288	2	0.0149	0.0716	no	FB
ko00270	Cysteine and methionine metabolism	16127	38	288	3	0.0154	0.0696	no	FB
ko00670	One carbon pool by folate	16127	12	288	2	0.0175	0.0772	no	FB
ko00903	Limonene and pinene degradation	16127	2	288	1	0.0179	0.0764	no	FB
ko03050	Proteasome	16127	36	288	3	0.0194	0.0807	no	FB
ko00310	Lysine degradation	16127	15	288	2	0.0204	0.0825	no	FB
ko04141	Protein processing in endoplasmic reticulum	16127	81	288	4	0.0228	0.0899	no	FB
ko00360	Phenylalanine metabolism	16127	17	288	2	0.0233	0.0898	no	FB

ko05205	Proteoglycans in cancer	16127	18	288	2	0.0264	0.0993	no	FB
ko01524	Platinum drug resistance	16127	78	288	3	0.0271	0.0993	no	FB
ko03430	Mismatch repair	16127	43	288	3	0.0288	0.1030	no	FB
ko00480	Glutathione metabolism	16127	48	288	2	0.0330	0.1157	no	FB
ko04152	AMPK signaling pathway	16127	20	288	2	0.0330	0.1157	no	FB
ko04810	Regulation of actin cytoskeleton	16127	20	288	2	0.0330	0.1157	no	FB
ko04972	Pancreatic secretion	16127	5	288	1	0.0351	0.1149	no	FB
ko05206	MicroRNAs in cancer	16127	22	288	2	0.0365	0.1172	no	FB
ko05012	Parkinson's disease	16127	46	288	3	0.0397	0.1248	no	FB
ko05152	Tuberculosis	16127	29	288	2	0.0401	0.1235	no	FB
ko01230	Biosynthesis of amino acids	16127	95	288	4	0.0434	0.1311	no	FB
ko05010	Alzheimer's disease	16127	54	288	3	0.0437	0.1293	no	FB
ko03060	Protein export	16127	20	288	2	0.0437	0.1271	no	FB
ko04130	SNARE interactions in vesicular transport	16127	22	288	2	0.0437	0.1271	no	FB
ko00250	Alanine, aspartate and glutamate metabolism	16127	24	288	2	0.0475	0.1330	no	FB
ko03410	Base excision repair	16127	22	288	2	0.0475	0.1330	no	FB
ko00020	Citrate cycle	16127	26	288	2	0.0513	0.1387	no	FB
ko00590	Arachidonic acid metabolism	16127	3	288	1	0.0517	0.1372	no	FB
ko04614	Renin-angiotensin system	16127	5	288	1	0.0517	0.1372	no	FB
ko04710	Circadian rhythm	16127	4	288	1	0.0517	0.1372	no	FB
ko04712	Circadian rhythm - plant	16127	4	288	1	0.0517	0.1372	no	FB
ko04913	Ovarian steroidogenesis	16127	3	288	1	0.0517	0.1372	no	FB

ko04923	Regulation of lipolysis in adipocytes	16127	3	288	1	0.0517	0.1372	no	FB
ko05031	Amphetamine addiction	16127	3	288	1	0.0517	0.1372	no	FB
ko05133	Pertussis	16127	5	288	1	0.0517	0.1372	no	FB
ko05220	Chronic myeloid leukemia	16127	6	288	1	0.0517	0.1372	no	FB
ko00627	Aminobenzoate degradation	16127	25	288	2	0.0552	0.1269	no	FB
ko00380	Tryptophan metabolism	16127	31	288	2	0.0592	0.1340	no	FB
ko01220	Degradation of aromatic compounds	16127	31	288	2	0.0592	0.1340	no	FB
ko05200	Pathways in cancer	16127	28	288	2	0.0592	0.1340	no	FB
ko00620	Pyruvate metabolism	16127	69	288	3	0.0606	0.1315	no	FB
ko04114	Oocyte meiosis	16127	32	288	2	0.0632	0.1351	no	FB
ko03013	RNA transport	16127	100	288	4	0.0674	0.1422	no	FB
ko00362	Benzoate degradation	16127	5	288	1	0.0677	0.1409	no	FB
ko00625	Chloroalkane and chloroalkene degradation	16127	5	288	1	0.0677	0.1409	no	FB
ko04270	Vascular smooth muscle contraction	16127	6	288	1	0.0677	0.1409	no	FB
ko04330	Notch signaling pathway	16127	5	288	1	0.0677	0.1409	no	FB
ko04340	Hedgehog signaling pathway	16127	5	288	1	0.0677	0.1409	no	FB
ko04720	Long-term potentiation	16127	6	288	1	0.0677	0.1409	no	FB
ko04750	Inflammatory mediator regulation of TRP channels	16127	4	288	1	0.0677	0.1409	no	FB
ko05140	Leishmaniasis	16127	6	288	1	0.0677	0.1409	no	FB
ko03020	RNA polymerase	16127	29	288	2	0.0713	0.1339	no	FB

ko04145	Phagosome	16127	28	288	2	0.0713	0.1339	no	FB
ko00100	Steroid biosynthesis	16127	37	288	2	0.0796	0.1459	no	FB
ko00514	Other types of O-glycan biosynthesis	16127	6	288	1	0.0831	0.1506	no	FB
ko00643	Styrene degradation	16127	5	288	1	0.0831	0.1506	no	FB
ko00950	Isoquinoline alkaloid biosynthesis	16127	8	288	1	0.0831	0.1506	no	FB
ko00960	Tropane, piperidine and pyridine alkaloid biosynthesis	16127	8	288	1	0.0831	0.1506	no	FB
ko04022	cGMP-PKG signaling pathway	16127	10	288	1	0.0831	0.1506	no	FB
ko04341	Hedgehog signaling pathway - fly	16127	6	288	1	0.0831	0.1506	no	FB
ko04391	Hippo signaling pathway - fly	16127	8	288	1	0.0831	0.1506	no	FB
ko04668	TNF signaling pathway	16127	7	288	1	0.0831	0.1506	no	FB
ko04976	Bile secretion	16127	8	288	1	0.0831	0.1506	no	FB
ko05222	Small cell lung cancer	16127	5	288	1	0.0831	0.1506	no	FB
ko04111	Cell cycle - yeast	16127	71	288	3	0.0837	0.1357	no	FB
ko04146	Peroxisome	16127	43	288	2	0.0879	0.1410	no	FB
ko04910	Insulin signaling pathway	16127	40	288	2	0.0921	0.1462	no	FB
ko00010	Glycolysis / Gluconeogenesis	16127	40	288	2	0.0963	0.1513	no	FB
ko04360	Axon guidance	16127	8	288	1	0.0979	0.1524	no	FB
ko04520	Adherens junction	16127	9	288	1	0.0979	0.1524	no	FB
ko04611	Platelet activation	16127	8	288	1	0.0979	0.1524	no	FB
ko04723	Retrograde endocannabinoid	16127	8	288	1	0.0979	0.1524	no	FB

	signaling								
ko04931	Insulin resistance	16127	7	288	1	0.0979	0.1524	no	FB
ko00230	Purine metabolism	16127	79	288	3	0.1075	0.1592	no	FB
ko01200	Carbon metabolism	16127	91	288	3	0.1099	0.1611	no	FB
ko04510	Focal adhesion	16127	10	288	1	0.1122	0.1631	no	FB
ko05204	Chemical carcinogenesis	16127	37	288	1	0.1122	0.1631	no	FB
ko05322	Systemic lupus erythematosus	16127	13	288	1	0.1122	0.1631	no	FB
ko04932	Non-alcoholic fatty liver disease	16127	41	288	2	0.1172	0.1656	no	FB
ko01523	Antifolate resistance	16127	8	288	1	0.1260	0.1764	no	FB
ko02024	Quorum sensing	16127	9	288	1	0.1260	0.1764	no	FB
ko04024	cAMP signaling pathway	16127	17	288	1	0.1260	0.1764	no	FB
ko04122	Sulfur relay system	16127	8	288	1	0.1260	0.1764	no	FB
ko04261	Adrenergic signaling in cardiomyocytes	16127	13	288	1	0.1260	0.1764	no	FB
ko04370	VEGF signaling pathway	16127	10	288	1	0.1260	0.1764	no	FB
ko04728	Dopaminergic synapse	16127	8	288	1	0.1260	0.1764	no	FB
ko05162	Measles	16127	10	288	1	0.1260	0.1764	no	FB
ko03040	Spliceosome	16127	87	288	3	0.1262	0.1647	no	FB
ko00720	Carbon fixation pathways in prokaryotes	16127	14	288	1	0.1392	0.1802	no	FB
ko04722	Neurotrophin signaling pathway	16127	15	288	1	0.1392	0.1802	no	FB
ko04113	Meiosis - yeast	16127	50	288	2	0.1456	0.1853	no	FB

ko00300	Lysine biosynthesis	16127	13	288	1	0.1519	0.1918	no	FB
ko04211	Longevity regulating pathway	16127	17	288	1	0.1519	0.1918	no	FB
ko04260	Cardiac muscle contraction	16127	13	288	1	0.1519	0.1918	no	FB
ko04530	Tight junction	16127	11	288	1	0.1519	0.1918	no	FB
ko04966	Collecting duct acid secretion	16127	10	288	1	0.1519	0.1918	no	FB
ko04144	Endocytosis	16127	51	288	2	0.1572	0.1906	no	FB
ko00650	Butanoate metabolism	16127	14	288	1	0.1641	0.1975	no	FB
ko04962	Vasopressin-regulated water reabsorption	16127	11	288	1	0.1641	0.1975	no	FB
ko05323	Rheumatoid arthritis	16127	11	288	1	0.1641	0.1975	no	FB
ko00071	Fatty acid degradation	16127	19	288	1	0.1871	0.2200	no	FB
ko00561	Glycerolipid metabolism	16127	17	288	1	0.1871	0.2200	no	FB
ko04068	FoxO signaling pathway	16127	22	288	1	0.1871	0.2200	no	FB
ko04623	Cytosolic DNA-sensing pathway	16127	13	288	1	0.1871	0.2200	no	FB
ko00220	Arginine biosynthesis	16127	16	288	1	0.1979	0.2258	no	FB
ko04912	GnRH signaling pathway	16127	18	288	1	0.1979	0.2258	no	FB
ko05131	Shigellosis	16127	17	288	1	0.1979	0.2258	no	FB
ko00240	Pyrimidine metabolism	16127	63	288	2	0.1989	0.2219	no	FB
ko00350	Tyrosine metabolism	16127	19	288	1	0.2083	0.2308	no	FB
ko05120	Epithelial cell signaling in Helicobacter pylori infection	16127	15	288	1	0.2083	0.2308	no	FB
ko00900	Terpenoid backbone biosynthesis	16127	17	288	1	0.2182	0.2384	no	FB
ko03008	Ribosome biogenesis in	16127	71	288	2	0.2217	0.2404	no	FB

	eukaryotes								
ko00340	Histidine metabolism	16127	19	288	1	0.2277	0.2453	no	FB
ko00410	beta-Alanine metabolism	16127	19	288	1	0.2277	0.2453	no	FB
ko00621	Dioxin degradation	16127	24	288	1	0.2368	0.2515	no	FB
ko00624	Polycyclic aromatic hydrocarbon degradation	16127	24	288	1	0.2368	0.2515	no	FB
ko05164	Influenza A	16127	23	288	1	0.2455	0.2572	no	FB
ko00626	Naphthalene degradation	16127	26	288	1	0.2539	0.2642	no	FB
ko04721	Synaptic vesicle cycle	16127	21	288	1	0.2539	0.2642	no	FB
ko05203	Viral carcinogenesis	16127	36	288	1	0.3078	0.3160	no	FB
ko00970	Aminoacyl-tRNA biosynthesis	16127	42	288	1	0.3273	0.3338	no	FB
ko05166	HTLV-I infection	16127	38	288	1	0.3315	0.3359	no	FB
ko03420	Nucleotide excision repair	16127	38	288	1	0.3459	0.3482	no	FB
ko03018	RNA degradation	16127	51	288	1	0.3691	0.3691	no	FB
ko04110	Cell cycle	16127	59	288	1	0.3691	0.3691	no	FB

Table S11 List of parental genes of exonics annotated as transcription factors.

Gene_Name	Best hits in Database	TF Family Name	E-value
GL29224	AraC_Af10007	Helix-turn-helix, AraC type	5.00E-11
GL15096	AraC_Afl0012	Helix-turn-helix, AraC type	7.00E-46
GL15327	AraC_Afl0012	Helix-turn-helix, AraC type	6.00E-17
GL24715	AraC_Cg0002	Helix-turn-helix, AraC type	8.00E-59
GL25003	AraC_Cgu0002	Helix-turn-helix, AraC type	1.00E-15
GL15342	AraC_Fg0013	Helix-turn-helix, AraC type	2.00E-06
GL30656	AraC_Fg0013	Helix-turn-helix, AraC type	5.00E-08
GL23411	AraC_Fv0010	Helix-turn-helix, AraC type	1.00E-30
GL24047	AraC_Ha0001	Helix-turn-helix, AraC type	1.00E-14
GL18792	AraC_Nc0009	Helix-turn-helix, AraC type	6.00E-12
GL25355	AraC_Nc0009	Helix-turn-helix, AraC type	9.00E-19
GL21580	AraC_Nc0012	Helix-turn-helix, AraC type	9.00E-26
GL26572	AraC_Pst0001	Helix-turn-helix, AraC type	9.00E-34
GL30422	AraC_Sca0003	Helix-turn-helix, AraC type	2.00E-09
GL30069	AraC_Sk0001	Helix-turn-helix, AraC type	7.00E-11
GL17625	AraC_Sr0003	Helix-turn-helix, AraC type	4.00E-08
GL21844	AraC_Ss0003	Helix-turn-helix, AraC type	3.00E-10
GL25417	AraC_Ss0004	Helix-turn-helix, AraC type	1.00E-90
GL18364	ARID_Cg0002	AT-rich interaction region	8.00E-06
GL28931	ARID_Pc0005	AT-rich interaction region	4.00E-18
GL24214	bHLH_Pg0003	bHLH	8.00E-10
GL22518	bHLH_Pm0001	bHLH	5.00E-06
GL26688	bZIP_Ss0010	bZIP	8.00E-06
GL23794	C2H2_AnA0065	C2H2 zinc finger	2.00E-08
GL24170	C2H2_Caw0016	C2H2 zinc finger	1.00E-10
GL20883	C2H2_Ct0080	C2H2 zinc finger	8.00E-06
GL18632	C2H2_Pb0120	C2H2 zinc finger	2.00E-06
GL29852	C2H2_Pg0062	C2H2 zinc finger	6.00E-09
GL22680	C2H2_Pi0072	C2H2 zinc finger	1.00E-15
GL30586	C2H2_Sj0039	C2H2 zinc finger	2.00E-08
GL22608	C2H2_So0020	C2H2 zinc finger	1.00E-40
GL29411	C2H2_Sr0010	C2H2 zinc finger	2.00E-09
GL21669	C2H2_Ta0088	C2H2 zinc finger	2.00E-17
GL30222	C2H2_Vd0056	C2H2 zinc finger	2.00E-27
GL16370	CCAAT_Caw0006	Heteromeric CCAAT factors	8.00E-06
GL26791	CCAAT_Pg0005	Heteromeric CCAAT factors	2.00E-06

GL30704	CCHC_Ab0003	Zinc finger, CCHC-type	5.00E-06
GL17366	CCHC_Ag0006	Zinc finger, CCHC-type	4.00E-06
GL18762	CCHC_Al0002	Zinc finger, CCHC-type	7.00E-19
GL25382	CCHC_Cgo0053	Zinc finger, CCHC-type	4.00E-06
GL26477	CCHC_Fg0017	Zinc finger, CCHC-type	3.00E-06
GL29863	CCHC_Pbl0001	Zinc finger, CCHC-type	7.00E-11
GL23036	CCHC_PCR0017	Zinc finger, CCHC-type	2.00E-11
GL29687	CCHC_Pi0105	Zinc finger, CCHC-type	3.00E-07
GL26588	CCHC_Pr0372	Zinc finger, CCHC-type	6.00E-07
GL18413	CCHC_Pr0379	Zinc finger, CCHC-type	1.00E-15
GL20592	CCHC_Pr0379	Zinc finger, CCHC-type	2.00E-11
GL20847	CCHC_Ps0467	Zinc finger, CCHC-type	9.00E-07
GL16715	DHHC_Vdm0002	Zinc finger, DHHC-type	5.00E-16
GL24139	DHHC_Vdm0004	Zinc finger, DHHC-type	2.00E-08
GL27298	GATA_Pc0015	GATA type zinc finger	7.00E-06
GL21693	GATA_Ss0006	GATA type zinc finger	4.00E-18
GL23073	GntR_Dh0001	Bacterial regulatory protein GntR, HTH	3.00E-06
GL28477	GRF_Pi0001	Zinc finger, GRF-type	4.00E-11
GL30182	GRF_Pr0002	Zinc finger, GRF-type	2.00E-08
GL15931	HMG_Afl0021	HMG	4.00E-06
GL20814	HMG_Afl0038	HMG	9.00E-06
GL25780	HMG_Ate0023	HMG	9.00E-06
GL25489	HMG_Fg0067	HMG	2.00E-06
GL29593	HMG_Fs0059	HMG	5.00E-74
GL20761	HMG_Fv0105	HMG	1.00E-17
GL20816	HMG_Kp0007	HMG	1.00E-05
GL31471	HMG_MP0006	HMG	5.00E-06
GL22778	HMG_Pb0041	HMG	3.00E-10
GL27852	HMG_Pr0036	HMG	4.00E-09
GL25671	HMG_Ps0038	HMG	2.00E-06
GL29690	HMG_Ptr0023	HMG	7.00E-06
GL22569	HMG_Sj0012	HMG	5.00E-11
GL22950	HMG_Ta0016	HMG	3.00E-09
GL18105	HMG_Um50019	HMG	6.00E-06
GL31774	HOMEO_Acl0019	Homeobox	4.00E-07
GL22551	HOMEO_Ha0006	Homeobox	4.00E-12
GL22656	HOMEO_Ha0006	Homeobox	1.00E-06
GL22592	HOMEO_Ha0012	Homeobox	7.00E-28
GL24198	HOMEO_Ha0012	Homeobox	3.00E-07
GL31640	HOMEO_Ha0012	Homeobox	5.00E-11
GL21747	HOMEO_Lb0008	Homeobox	3.00E-06
GL28295	HOMEO_Lb0008	Homeobox	2.00E-08

GL29585	HOMEO_Mg70015	Homeobox	7.00E-06
GL21717	HOMEO_Sr0001	Homeobox	2.00E-17
GL26471	HOMEO_Sr0001	Homeobox	3.00E-06
GL25850	HOMEO_Sr0003	Homeobox	3.00E-09
GL25231	HOMEO_Sr0004	Homeobox	9.00E-08
GL24092	HOMEO_Ta0001	Homeobox	1.00E-16
GL22666	HOMEOLIKE_AnA0011	Homeodomain-like	7.00E-74
GL21684	HOMEOLIKE_Kp0009	Homeodomain-like	9.00E-06
GL29258	Lambda_Ag0003	Lambda repressor-like, DNA-binding	2.00E-19
GL22678	Lambda_Mg70005	Lambda repressor-like, DNA-binding	6.00E-29
GL20745	LSD1_Pr0002	Zinc finger, LSD1-type	4.00E-10
GL28656	LSD1_Pst0001	Zinc finger, LSD1-type	5.00E-08
GL28663	LSD1_Yl0001	Zinc finger, LSD1-type	7.00E-29
GL17361	MIZ_Mgr0001	Zinc finger, MIZ-type	9.00E-06
GL18743	MIZ_PCR0002	Zinc finger, MIZ-type	9.00E-06
GL22662	Myb_Fv0113	Myb	1.00E-40
GL22353	Myb_Ha0005	Myb	9.00E-11
GL29333	Myb_Nc0076	Myb	7.00E-06
GL30057	Myb_Pi0023	Myb	2.00E-06
GL22396	Myb_Um50009	Myb	7.00E-19
GL22708	OBFOLD_Afl0082	Nucleic acid-binding, OB-fold	1.00E-40
GL26735	OBFOLD_Caw0021	Nucleic acid-binding, OB-fold	8.00E-07
GL21362	OBFOLD_Ct0057	Nucleic acid-binding, OB-fold	3.00E-07
GL30301	OBFOLD_Fg0106	Nucleic acid-binding, OB-fold	6.00E-10
GL22968	OBFOLD_Fv0103	Nucleic acid-binding, OB-fold	5.08E+01
GL25820	OBFOLD_Ha0002	Nucleic acid-binding, OB-fold	7.00E-18
GL25300	OBFOLD_Kp0023	Nucleic acid-binding, OB-fold	3.00E-08
GL25146	OBFOLD_Le0010	Nucleic acid-binding, OB-fold	3.00E-08
GL30097	OBFOLD_Pbl0031	Nucleic acid-binding, OB-fold	2.00E-12
GL30361	OBFOLD_Sb0022	Nucleic acid-binding, OB-fold	7.00E-83
GL21387	OBFOLD_Sca0030	Nucleic acid-binding, OB-fold	5.00E-08
GL24337	OBFOLD_Sca0030	Nucleic acid-binding, OB-fold	1.00E-12
GL25480	PARP_Pp0002	Zinc finger, PARP-type	3.00E-06
GL24286	WING_Afl0050	Winged helix repressor DNA-binding	4.00E-09
GL23507	WING_Ate0013	Winged helix repressor DNA-binding	6.00E-07
GL25987	WING_Cih0009	Winged helix repressor DNA-binding	3.00E-17
GL25822	WING_Hc0060	Winged helix repressor DNA-binding	1.00E-05

GL30712	WING_Pb0003	Winged helix repressor DNA-binding	4.00E-18
GL23585	WING_Pi0024	Winged helix repressor DNA-binding	6.00E-06
GL25610	WING_Pi0028	Winged helix repressor DNA-binding	2.00E-07
GL29251	WING_Pm0001	Winged helix repressor DNA-binding	2.00E-30
GL25902	WING_Sb0008	Winged helix repressor DNA-binding	6.00E-31
GL15663	YL1_Cgo0001	YL1 nuclear protein	9.00E-06
GL23798	YL1_Fv0003	YL1 nuclear protein	7.00E-07
GL20790	ZnCys_Acl0329	Zn2Cys6	2.00E-07
GL21206	ZnCys_Afl0361	Zn2Cys6	6.00E-99
GL31764	ZnCys_Afl0374	Zn2Cys6	2.00E-07
GL15004	ZnCys_Afl0452	Zn2Cys6	3.00E-27
GL17382	ZnCys_Afl0496	Zn2Cys6	2.00E-14
GL22327	ZnCys_Afl0496	Zn2Cys6	1.00E-06
GL27278	ZnCys_Afl0496	Zn2Cys6	5.00E-12
GL21586	ZnCys_Afl0553	Zn2Cys6	1.00E-58
GL20786	ZnCys_AnA0275	Zn2Cys6	2.00E-13
GL26008	ZnCys_AnA0275	Zn2Cys6	3.00E-06
GL18179	ZnCys_Cgo0114	Zn2Cys6	2.00E-09
GL22587	ZnCys_Cgo0114	Zn2Cys6	1.00E-12
GL22203	ZnCys_CnD0160	Zn2Cys6	4.00E-06
GL26114	ZnCys_Dh0089	Zn2Cys6	3.00E-06
GL30770	ZnCys_Fs0701	Zn2Cys6	1.00E-20
GL28308	ZnCys_Ha0063	Zn2Cys6	1.00E-20
GL20764	ZnCys_Hc0237	Zn2Cys6	1.00E-08
GL27036	ZnCys_Mgr0119	Zn2Cys6	1.00E-25
GL23647	ZnCys_MP0008	Zn2Cys6	9.00E-72
GL18500	ZnCys_Pa0249	Zn2Cys6	4.00E-23
GL30386	ZnCys_Pbl0028	Zn2Cys6	7.00E-06
GL21056	ZnCys_PCR0285	Zn2Cys6	4.00E-09
GL24072	ZnCys_PCR0285	Zn2Cys6	9.00E-13
GL30875	ZnCys_Pm0052	Zn2Cys6	4.00E-07
GL21093	ZnCys_Pm0161	Zn2Cys6	1.00E-09
GL15391	ZnCys_Pp0008	Zn2Cys6	2.00E-12
GL23491	ZnCys_Pp0008	Zn2Cys6	6.00E-06
GL26775	ZnCys_Pp0008	Zn2Cys6	8.00E-06
GL21694	ZnCys_Sr0007	Zn2Cys6	6.00E-06
GL21135	ZnCys_Ss0094	Zn2Cys6	4.00E-16
GL20660	ZnCys_Tr0383	Zn2Cys6	1.00E-16

GL28294	ZnCys_Tr0383	Zn2Cys6	1.00E-07
GL16020	ZnCys_Tvg0292	Zn2Cys6	7.00E-06
GL28634	ZnCys_Tvg0292	Zn2Cys6	1.00E-12
GL24012	ZnCys_Tvg0337	Zn2Cys6	6.00E-08

Table S12 Differentially expressed exonics among the three development stages in *G. lucidum* (Table S12.xlsx).

Table S13 Correlation for expression profiles between exonic exonics and their parent genes in *G. lucidum*.

Exonic ID	Log2(Average Condition RPM)			Gene ID	Log2(RPKM)			Pearson correlation coefficient	Q-Value	Significance level
	M	P	FB		M	P	FB			
GaLu96scf_47_92737_93159	9.60	0.00	0.00	GL25107	10.78	7.31	7.33	1.00	0.0000	q<0.01
GaLu96scf_24_374067_374477	8.51	0.00	0.00	GL23146	6.17	1.06	1.13	1.00	0.0000	q<0.01
GaLu96scf_9_958374_958775	8.02	0.00	0.00	GL18179	5.22	7.07	7.05	-1.00	0.0000	q<0.01
GaLu96scf_8_586612_586805	10.23	0.00	5.75	GL26114	4.34	3.42	3.95	1.00	0.0000	q<0.01
GaLu96scf_26_126835_127042	7.48	0.00	0.00	GL23299	4.30	5.97	5.94	-1.00	0.0000	q<0.01
GaLu96scf_4_169285_169975	0.00	6.14	5.75	GL28477	5.82	4.76	4.85	-1.00	0.0000	q<0.01
GaLu96scf_13_276282_276873	7.54	0.00	0.00	GL15391	3.56	4.97	5.00	-1.00	0.0000	q<0.01
GaLu96scf_1_2114443_2114867	7.48	0.00	0.00	GL29950	4.18	3.85	3.85	1.00	0.0000	q<0.01
GaLu96scf_4_692800_693247	7.96	0.00	0.00	GL24584	7.42	4.94	5.00	1.00	0.0000	q<0.01
GaLu96scf_4_692800_693127	7.77	0.00	0.00	GL24584	7.42	4.94	5.00	1.00	0.0000	q<0.01
GaLu96scf_33_12161_12892	8.38	0.00	0.00	GL24222	5.10	0.95	0.85	1.00	0.0000	q<0.01
GaLu96scf_4_1127979_1128467	7.80	8.52	8.30	GL24752	1.33	3.69	2.92	1.00	0.0000	q<0.01
GaLu96scf_8_1384261_1384494	7.48	0.00	0.00	GL25850	4.46	2.94	2.91	1.00	0.0000	q<0.01
GaLu96scf_7_647053_647313	9.21	8.35	8.53	GL25610	8.50	4.51	5.43	1.00	0.0000	q<0.01
GaLu96scf_8_359260_361090	7.80	0.00	0.00	GL25880	4.74	1.65	1.57	1.00	0.0000	q<0.01
GaLu96scf_37_224634_225370	0.00	6.72	7.03	GL24384	2.04	3.78	3.91	1.00	0.0000	q<0.01
GaLu96scf_22_181588_181808	10.67	0.00	0.00	GL22913	10.75	1.63	1.88	1.00	0.0000	q<0.01
GaLu96scf_2_889910_890290	11.19	0.00	0.00	GL16169	9.21	5.27	5.16	1.00	0.0000	q<0.01

GaLu96scf_26_531078_531338	0.00	0.00	7.63	GL23342	2.20	2.12	4.90	1.00	0.0000	q<0.01
GaLu96scf_15_50637_50979	0.00	7.36	7.61	GL21556	4.55	5.36	5.41	1.00	0.0000	q<0.01
GaLu96scf_38_176312_176825	9.08	0.00	0.00	GL24391	5.69	7.56	7.62	-1.00	0.0000	q<0.01
GaLu96scf_19_175500_175732	10.17	0.00	0.00	GL22184	5.73	2.79	2.69	1.00	0.0000	q<0.01
GaLu96scf_2_821935_822137	9.77	0.00	0.00	GL22666	7.53	2.74	2.89	1.00	0.0000	q<0.01
GaLu96scf_2_820827_822137	10.56	0.00	0.00	GL22666	7.53	2.74	2.89	1.00	0.0000	q<0.01
GaLu96scf_2_820827_821844	9.12	0.00	0.00	GL22666	7.53	2.74	2.89	1.00	0.0000	q<0.01
GaLu96scf_27_284010_284295	10.10	0.00	0.00	GL23411	7.95	4.83	4.73	1.00	0.0000	q<0.01
GaLu96scf_5_811662_812027	9.08	0.00	0.00	GL25253	9.86	3.29	3.06	1.00	0.0000	q<0.01
GaLu96scf_5_811092_811588	7.80	0.00	0.00	GL25253	9.86	3.29	3.06	1.00	0.0000	q<0.01
GaLu96scf_6_217665_218029	11.86	0.00	0.00	GL31658	6.32	2.41	2.28	1.00	0.0000	q<0.01
GaLu96scf_6_217787_218029	7.80	0.00	0.00	GL31658	6.32	2.41	2.28	1.00	0.0000	q<0.01
GaLu96scf_11_1283922_1284272	10.25	0.00	0.00	GL20814	5.58	4.12	4.17	1.00	0.0000	q<0.01
GaLu96scf_1_822816_823531	7.22	8.40	8.76	GL29633	1.43	1.91	2.08	1.00	0.0000	q<0.01
GaLu96scf_27_464123_464435	8.38	0.00	0.00	GL23453	5.55	2.77	2.66	1.00	0.0000	q<0.01
GaLu96scf_4_988325_988869	8.80	0.00	0.00	GL24619	6.77	5.95	5.92	1.00	0.0000	q<0.01
GaLu96scf_5_11814_12136	7.54	0.00	0.00	GL25146	5.63	2.94	3.04	1.00	0.0000	q<0.01
GaLu96scf_5_13170_13484	7.54	0.00	0.00	GL25146	5.63	2.94	3.04	1.00	0.0000	q<0.01
GaLu96scf_3_51339_51710	8.21	0.00	0.00	GL16814	7.82	10.17	10.08	-1.00	0.0000	q<0.01
GaLu96scf_28_108207_108560	7.80	0.00	0.00	GL23496	4.87	6.12	6.07	-1.00	0.0000	q<0.01
GaLu96scf_9_1049221_1049550	9.20	0.00	0.00	GL26471	4.91	3.85	3.80	1.00	0.0000	q<0.01
GaLu96scf_4_941240_941524	7.74	6.14	6.81	GL17366	3.10	2.54	2.79	1.00	0.0000	q<0.01
GaLu96scf_45_47325_47584	13.97	0.00	0.00	GL28656	5.90	4.54	4.47	1.00	0.0000	q<0.01
GaLu96scf_45_47325_47496	7.54	0.00	0.00	GL28656	5.90	4.54	4.47	1.00	0.0000	q<0.01
GaLu96scf_36_97718_98460	7.54	0.00	0.00	GL29258	2.77	5.70	5.84	-1.00	0.0000	q<0.01

GaLu96scf_9_208429_208736	7.96	0.00	0.00	GL18143	3.32	2.25	2.20	1.00	0.0000	q<0.01
GaLu96scf_9_497310_497494	10.05	6.36	0.00	GL26637	4.42	4.06	3.53	1.00	0.0000	q<0.01
GaLu96scf_5_595052_595416	8.21	0.00	6.73	GL25234	6.86	7.37	6.98	-1.00	0.0000	q<0.01
GaLu96scf_11_1234719_1234903	7.48	0.00	0.00	GL20816	3.06	2.87	2.88	1.00	0.0000	q<0.01
GaLu96scf_3_1675629_1676107	11.38	0.00	7.05	GL23899	3.26	2.65	3.00	1.00	0.0000	q<0.01
GaLu96scf_10_57748_58352	7.78	0.00	0.00	GL15004	3.62	1.04	0.89	1.00	0.0000	q<0.01
GaLu96scf_31_249500_249822	7.74	0.00	0.00	GL24117	3.87	3.52	3.50	1.00	0.0000	q<0.01
GaLu96scf_8_1226373_1226573	7.54	0.00	0.00	GL25958	2.55	4.82	4.69	-1.00	0.0000	q<0.01
GaLu96scf_1_1408495_1408813	10.71	0.00	0.00	GL30901	6.52	5.24	5.17	1.00	0.0000	q<0.01
GaLu96scf_35_328571_328844	17.75	9.72	10.58	GL24337	10.13	5.73	5.95	1.00	0.0000	q<0.01
GaLu96scf_8_1303781_1304246	7.54	0.00	0.00	GL25853	4.70	6.23	6.14	-1.00	0.0000	q<0.01
GaLu96scf_25_479247_479504	0.00	10.02	10.32	GL23247	2.61	11.20	10.93	1.00	0.0000	q<0.01
GaLu96scf_19_585745_586100	7.54	0.00	0.00	GL18873	7.14	8.24	8.31	-1.00	0.0000	q<0.01
GaLu96scf_9_917450_917719	8.02	0.00	0.00	GL26657	3.89	5.81	5.69	-1.00	0.0000	q<0.01
GaLu96scf_11_612424_612944	10.60	0.00	6.73	GL20868	3.51	2.70	3.18	1.00	0.0000	q<0.01
GaLu96scf_11_132103_132409	8.21	0.00	0.00	GL20790	3.92	3.48	3.50	1.00	0.0000	q<0.01
GaLu96scf_17_265349_265533	7.54	0.00	0.00	GL18743	5.40	4.81	4.77	1.00	0.0000	q<0.01
GaLu96scf_27_128048_128539	16.38	15.04	14.69	GL23383	5.06	4.28	4.14	1.00	0.0000	q<0.01
GaLu96scf_12_922030_922271	12.49	9.74	10.26	GL20960	5.20	7.88	7.54	-1.00	0.0000	q<0.01
GaLu96scf_1_343489_344528	7.48	0.00	0.00	GL30986	2.02	2.84	2.78	-1.00	0.0000	q<0.01
GaLu96scf_2_1497801_1497965	8.97	0.00	0.00	GL22362	7.75	3.27	3.58	1.00	0.0000	q<0.01
GaLu96scf_1_4434778_4435327	0.00	0.00	7.53	GL29561	4.95	4.96	4.78	-1.00	0.0000	q<0.01
GaLu96scf_7_1121284_1121658	8.48	0.00	0.00	GL28863	7.03	4.14	3.92	1.00	0.0000	q<0.01
GaLu96scf_22_188417_188634	7.99	0.00	0.00	GL22966	7.12	9.12	8.98	-1.00	0.0000	q<0.01
GaLu96scf_17_474886_475109	9.18	0.00	0.00	GL21931	9.80	7.96	7.81	1.00	0.0000	q<0.01

GaLu96scf_2_1724880_1725615	9.38	0.00	0.00	GL22587	2.43	3.27	3.34	-1.00	0.0000	q<0.01
GaLu96scf_13_1002778_1003234	7.54	0.00	0.00	GL21206	3.87	5.27	5.16	-1.00	0.0000	q<0.01
GaLu96scf_4_169756_170276	5.25	7.45	7.12	GL28477	5.82	4.76	4.85	-1.00	0.0000	q<0.01
GaLu96scf_4_1306972_1307375	9.07	5.38	6.32	GL24505	4.64	5.41	5.17	-1.00	0.0000	q<0.01
GaLu96scf_4_6819_7159	8.63	7.01	5.90	GL24715	3.01	3.12	3.22	-1.00	0.0000	q<0.01
GaLu96scf_16_675161_675427	10.46	7.25	8.30	GL15686	4.29	5.60	5.26	-1.00	0.0000	q<0.01
GaLu96scf_9_1409566_1409817	8.53	0.00	0.00	GL26506	3.24	3.84	3.79	-1.00	0.0000	q<0.01
GaLu96scf_8_1164705_1165005	7.80	0.00	0.00	GL20241	5.70	2.35	2.63	1.00	0.0000	q<0.01
GaLu96scf_8_1164203_1164592	9.71	0.00	0.00	GL20241	5.70	2.35	2.63	1.00	0.0000	q<0.01
GaLu96scf_9_1106239_1106552	8.49	0.00	0.00	GL26480	7.75	6.72	6.81	1.00	0.0000	q<0.01
GaLu96scf_11_1110999_1111405	7.48	0.00	0.00	GL20673	4.68	6.30	6.16	-1.00	0.0000	q<0.01
GaLu96scf_8_463132_463471	7.74	0.00	0.00	GL25987	6.79	7.47	7.41	-1.00	0.0000	q<0.01
GaLu96scf_5_683996_684442	7.54	0.00	0.00	GL17625	4.46	5.49	5.40	-1.00	0.0000	q<0.01
GaLu96scf_29_427459_427700	11.35	9.98	9.88	GL23621	7.36	4.36	4.46	1.00	0.0000	q<0.01
GaLu96scf_4_168492_169400	0.00	10.65	10.78	GL28477	5.82	4.76	4.85	-1.00	0.0000	q<0.01
GaLu96scf_2_728269_728479	8.33	0.00	0.00	GL27629	7.52	4.87	4.58	1.00	0.0000	q<0.01
GaLu96scf_1_1350356_1350654	7.54	0.00	0.00	GL29865	2.97	4.48	4.65	-1.00	0.0000	q<0.01
GaLu96scf_18_621762_622038	11.34	0.00	0.00	GL22024	6.45	4.91	4.73	1.00	0.0000	q<0.01
GaLu96scf_13_572959_573298	10.39	0.00	0.00	GL21186	7.05	4.07	3.73	1.00	0.0000	q<0.01
GaLu96scf_9_468608_469135	7.80	0.00	0.00	GL26635	3.41	2.22	2.34	1.00	0.0000	q<0.01
GaLu96scf_47_82969_83169	5.25	6.55	6.32	GL25104	2.02	3.23	3.13	1.00	0.0000	q<0.01
GaLu96scf_1_1186490_1186746	7.80	0.00	0.00	GL29333	4.12	2.86	2.99	1.00	0.0000	q<0.01
GaLu96scf_12_837790_838122	10.74	8.33	6.89	GL20950	4.02	3.75	3.64	0.99	0.0000	q<0.01
GaLu96scf_8_68809_69109	7.54	0.00	0.00	GL26027	2.15	4.40	4.16	-0.99	0.0000	q<0.01
GaLu96scf_22_187637_187860	8.33	5.38	5.90	GL22966	7.12	9.12	8.98	-0.99	0.0000	q<0.01

GaLu96scf_16_604576_605067	12.09	4.60	4.77	GL21653	6.18	3.88	4.19	0.99	0.0000	q<0.01
GaLu96scf_16_916727_917143	7.48	0.00	0.00	GL21669	6.31	5.41	5.51	0.99	0.0000	q<0.01
GaLu96scf_17_302614_303070	8.96	8.22	0.00	GL21921	5.66	5.63	6.52	-0.99	0.0000	q<0.01
GaLu96scf_45_107155_107404	8.96	0.00	0.00	GL25061	7.94	2.57	3.19	0.99	0.0000	q<0.01
GaLu96scf_3_507235_507764	7.80	0.00	4.77	GL23732	5.71	6.61	5.97	-0.99	0.0000	q<0.01
GaLu96scf_1_2158293_2158658	8.49	0.00	0.00	GL30942	4.58	4.89	4.86	-0.99	0.0000	q<0.01
GaLu96scf_8_593249_593534	8.53	0.00	6.32	GL25902	3.85	3.27	3.76	0.99	0.0000	q<0.01
GaLu96scf_2_2221011_2221383	9.73	0.00	5.06	GL22403	4.17	5.32	4.61	-0.99	0.0000	q<0.01
GaLu96scf_15_966920_967395	10.46	7.94	8.10	GL21618	6.21	0.64	0.26	0.99	0.0000	q<0.01
GaLu96scf_19_390915_391439	9.71	0.00	0.00	GL22154	4.17	2.47	2.68	0.99	0.0000	q<0.01
GaLu96scf_1_336402_336711	8.20	0.00	0.00	GL30301	7.64	4.65	4.21	0.99	0.0000	q<0.01
GaLu96scf_33_19167_19452	11.55	0.00	0.00	GL24198	6.90	2.80	2.20	0.99	0.0000	q<0.01
GaLu96scf_33_18524_19026	8.62	0.00	0.00	GL24198	6.90	2.80	2.20	0.99	0.0000	q<0.01
GaLu96scf_32_29345_29713	8.00	4.60	0.00	GL28308	4.01	3.90	3.79	0.99	0.0000	q<0.01
GaLu96scf_1_2659518_2659735	0.00	8.10	7.12	GL29411	0.55	2.63	2.65	0.99	0.0000	q<0.01
GaLu96scf_1_2065870_2066251	7.80	0.00	0.00	GL30536	5.32	6.68	6.50	-0.99	0.0000	q<0.01
GaLu96scf_1_223979_224453	0.00	6.36	7.71	GL29930	7.41	5.60	5.52	-0.99	0.0000	q<0.01
GaLu96scf_3_586110_586294	9.21	7.36	6.32	GL23736	6.85	6.55	6.46	0.99	0.0000	q<0.01
GaLu96scf_11_86033_87347	7.54	0.00	0.00	GL20786	2.13	3.12	2.98	-0.99	0.0000	q<0.01
GaLu96scf_11_1234719_1235461	10.37	8.19	7.95	GL20816	3.06	2.87	2.88	0.99	0.0000	q<0.01
GaLu96scf_20_845487_845780	0.00	6.94	9.77	GL22726	0.00	4.18	4.99	0.99	0.0000	q<0.01
GaLu96scf_3_1121840_1122081	9.12	0.00	0.00	GL23646	7.81	6.03	5.74	0.99	0.0000	q<0.01
GaLu96scf_3_1121840_1122206	9.83	0.00	0.00	GL23646	7.81	6.03	5.74	0.99	0.0000	q<0.01
GaLu96scf_2_1331117_1331659	7.80	0.00	0.00	GL22353	4.05	1.64	2.00	0.99	0.0000	q<0.01
GaLu96scf_8_1159399_1159900	10.78	6.14	7.05	GL25837	6.99	4.68	4.80	0.99	0.0000	q<0.01

GaLu96scf_9_672509_673108	7.80	0.00	0.00	GL20443	7.49	8.51	8.36	-0.99	0.0000	q<0.01
GaLu96scf_2_1770335_1770882	6.81	9.47	9.46	GL22592	2.04	3.37	3.15	0.99	0.0000	q<0.01
GaLu96scf_8_89554_89854	10.64	8.30	9.07	GL26030	5.76	7.06	6.47	-0.99	0.0000	q<0.01
GaLu96scf_11_965353_965635	6.81	5.57	7.24	GL20883	5.41	5.17	5.57	0.99	0.0000	q<0.01
GaLu96scf_7_848191_848404	12.38	0.00	0.00	GL25620	10.42	6.98	7.53	0.99	0.0000	q<0.01
GaLu96scf_7_847373_848404	10.78	0.00	0.00	GL25620	10.42	6.98	7.53	0.99	0.0000	q<0.01
GaLu96scf_7_847536_847936	9.70	0.00	0.00	GL25620	10.42	6.98	7.53	0.99	0.0000	q<0.01
GaLu96scf_19_259914_260237	7.54	0.00	0.00	GL15912	7.64	9.44	9.15	-0.99	0.0000	q<0.01
GaLu96scf_19_259702_260237	8.21	0.00	0.00	GL15912	7.64	9.44	9.15	-0.99	0.0000	q<0.01
GaLu96scf_52_7460_8063	7.80	0.00	0.00	GL25501	10.19	9.28	9.10	0.99	0.0000	q<0.01
GaLu96scf_12_162281_162525	8.21	0.00	0.00	GL21029	6.05	4.32	3.98	0.99	0.0000	q<0.01
GaLu96scf_34_95413_95660	9.16	7.19	0.00	GL24286	7.32	6.94	6.28	0.99	0.0000	q<0.01
GaLu96scf_31_201444_202379	8.38	7.36	7.63	GL24088	7.77	4.29	5.70	0.99	0.0000	q<0.01
GaLu96scf_7_499827_500109	9.28	0.00	0.00	GL28931	3.60	4.42	4.59	-0.99	0.0000	q<0.01
GaLu96scf_11_1153012_1153342	12.49	9.63	10.28	GL20745	6.40	5.79	6.02	0.99	0.0000	q<0.01
GaLu96scf_16_544240_544486	11.84	0.00	0.00	GL18694	7.62	3.10	3.88	0.99	0.0000	q<0.01
GaLu96scf_32_167826_168203	8.16	0.00	0.00	GL24162	7.70	3.95	4.60	0.99	0.0000	q<0.01
GaLu96scf_5_516816_517239	13.37	6.36	6.04	GL17615	6.39	4.70	4.25	0.99	0.0000	q<0.01
GaLu96scf_2_778974_780464	13.70	10.74	11.72	GL22662	4.01	5.68	5.39	-0.99	0.0000	q<0.01
GaLu96scf_2_777117_778787	7.80	0.00	0.00	GL22662	4.01	5.68	5.39	-0.99	0.0000	q<0.01
GaLu96scf_28_437148_437556	9.93	0.00	0.00	GL23491	8.49	6.11	5.59	0.99	0.0000	q<0.01
GaLu96scf_28_434983_435391	10.53	0.00	0.00	GL23491	8.49	6.11	5.59	0.99	0.0000	q<0.01
GaLu96scf_6_871714_872157	7.16	0.00	5.75	GL31341	7.50	8.48	7.85	-0.99	0.0000	q<0.01
GaLu96scf_27_428519_428861	8.98	0.00	0.00	GL16711	4.92	1.45	2.08	0.99	0.0000	q<0.01
GaLu96scf_1_1560803_1561066	8.38	4.60	6.43	GL29355	2.89	4.79	4.16	-0.98	0.0000	q<0.01

GaLu96scf_6_306628_307187	0.00	5.38	6.73	GL31411	3.05	3.55	3.56	0.98	0.0000	q<0.01
GaLu96scf_1_3183139_3183352	8.53	0.00	0.00	GL30324	5.32	5.68	5.61	-0.98	0.0000	q<0.01
GaLu96scf_1_613526_613932	8.80	0.00	0.00	GL29617	8.88	6.19	5.58	0.98	0.0000	q<0.01
GaLu96scf_8_413101_413324	8.53	0.00	0.00	GL26101	6.58	7.62	7.42	-0.98	0.0000	q<0.01
GaLu96scf_9_866454_867150	9.08	10.31	9.97	GL26572	3.78	7.00	6.68	0.98	0.0000	q<0.01
GaLu96scf_7_1425181_1425884	8.02	0.00	0.00	GL25711	8.66	7.88	7.69	0.98	0.0000	q<0.01
GaLu96scf_43_25907_26321	7.50	0.00	0.00	GL25003	1.46	0.98	1.08	0.98	0.0000	q<0.01
GaLu96scf_27_366191_366606	8.02	0.00	0.00	GL23395	2.03	4.55	4.07	-0.98	0.0000	q<0.01
GaLu96scf_1_2046624_2046987	13.79	0.00	0.00	GL30934	9.85	6.40	5.57	0.98	0.0000	q<0.01
GaLu96scf_1_2046413_2046987	10.61	0.00	0.00	GL30934	9.85	6.40	5.57	0.98	0.0000	q<0.01
GaLu96scf_1_2046624_2047207	8.38	0.00	0.00	GL30934	9.85	6.40	5.57	0.98	0.0000	q<0.01
GaLu96scf_2_2081953_2082257	8.21	0.00	0.00	GL22608	4.69	3.10	2.72	0.98	0.0000	q<0.01
GaLu96scf_2_2081953_2082786	8.21	0.00	0.00	GL22608	4.69	3.10	2.72	0.98	0.0000	q<0.01
GaLu96scf_31_430223_430702	9.12	0.00	6.32	GL24072	7.23	3.87	5.64	0.98	0.0000	q<0.01
GaLu96scf_22_759446_759774	7.54	0.00	0.00	GL22951	6.31	4.56	4.13	0.98	0.0000	q<0.01
GaLu96scf_11_100041_100404	0.00	8.00	6.32	GL20660	4.62	3.40	3.41	-0.98	0.0000	q<0.01
GaLu96scf_22_744830_745187	9.78	0.00	0.00	GL22950	4.74	3.63	3.86	0.98	0.0000	q<0.01
GaLu96scf_18_621638_621839	0.00	7.59	6.73	GL22024	6.45	4.91	4.73	-0.98	0.0000	q<0.01
GaLu96scf_6_124526_124939	7.54	0.00	0.00	GL31371	11.76	7.47	8.36	0.98	0.0000	q<0.01
GaLu96scf_1_564557_564850	0.00	6.36	7.42	GL30398	4.81	8.00	7.76	0.98	0.0000	q<0.01
GaLu96scf_1_4460049_4460531	7.54	0.00	0.00	GL30130	9.96	4.28	5.46	0.98	0.0000	q<0.01
GaLu96scf_5_516816_518001	8.38	0.00	0.00	GL17615	6.39	4.70	4.25	0.98	0.0000	q<0.01
GaLu96scf_5_517364_518001	8.65	0.00	0.00	GL17615	6.39	4.70	4.25	0.98	0.0000	q<0.01
GaLu96scf_2_179449_179714	10.60	0.00	0.00	GL27501	7.48	7.02	6.90	0.98	0.0000	q<0.01
GaLu96scf_1_4029699_4029994	7.96	0.00	0.00	GL30772	4.87	6.15	5.88	-0.98	0.0000	q<0.01

GaLu96scf_31_83945_84343	8.76	0.00	0.00	GL28282	9.77	6.62	5.75	0.98	0.0000	q<0.01
GaLu96scf_2_778673_779108	7.54	9.73	9.84	GL22662	4.01	5.68	5.39	0.98	0.0000	q<0.01
GaLu96scf_1_3332784_3333104	13.30	11.88	12.68	GL30048	9.06	9.76	9.23	-0.98	0.0000	q<0.01
GaLu96scf_5_419746_420267	0.00	6.55	6.62	GL25223	3.96	6.36	5.85	0.98	0.0000	q<0.01
GaLu96scf_14_977560_978372	11.13	12.22	12.09	GL27220	3.07	4.49	4.03	0.98	0.0000	q<0.01
GaLu96scf_2_1238285_1238581	8.91	0.00	6.73	GL22552	3.11	3.68	3.36	-0.98	0.0000	q<0.01
GaLu96scf_1_1853379_1853571	7.96	0.00	0.00	GL30222	2.79	3.31	3.19	-0.98	0.0000	q<0.01
GaLu96scf_28_525456_526191	7.54	0.00	0.00	GL23538	4.61	4.42	4.46	0.98	0.0000	q<0.01
GaLu96scf_13_985328_985970	8.91	0.00	0.00	GL15443	5.95	3.09	3.74	0.98	0.0000	q<0.01
GaLu96scf_16_825491_825940	7.48	0.00	0.00	GL18716	6.20	6.72	6.88	-0.98	0.0000	q<0.01
GaLu96scf_11_725031_725261	10.02	0.00	7.05	GL20718	5.23	5.59	5.40	-0.98	0.0000	q<0.01
GaLu96scf_7_647515_647836	8.38	0.00	0.00	GL25610	8.50	4.51	5.43	0.98	0.0000	q<0.01
GaLu96scf_13_436487_436788	12.52	7.55	9.16	GL21226	6.66	8.32	8.13	-0.98	0.0000	q<0.01
GaLu96scf_1_89438_89611	0.00	5.38	7.05	GL30875	5.06	5.91	5.91	0.98	0.0000	q<0.01
GaLu96scf_12_358451_359047	9.19	0.00	5.06	GL27036	6.56	1.32	3.17	0.97	0.0000	q<0.01
GaLu96scf_12_716899_717846	10.34	0.00	5.06	GL21074	3.75	1.97	2.48	0.97	0.0000	q<0.01
GaLu96scf_23_84546_84768	8.02	0.00	0.00	GL23073	2.64	1.56	1.82	0.97	0.0000	q<0.01
GaLu96scf_11_1309786_1310357	0.00	7.87	8.94	GL20818	3.65	3.87	3.99	0.97	0.0000	q<0.01
GaLu96scf_37_75073_75655	7.80	0.00	0.00	GL24358	9.11	6.38	7.03	0.97	0.0000	q<0.01
GaLu96scf_11_627413_627989	11.50	0.00	0.00	GL20764	8.38	5.94	6.52	0.97	0.0000	q<0.01
GaLu96scf_27_432715_433523	8.02	0.00	6.04	GL28055	3.52	3.82	3.65	-0.97	0.0000	q<0.01
GaLu96scf_11_550698_551236	7.80	0.00	0.00	GL20860	2.77	3.72	3.49	-0.97	0.0000	q<0.01
GaLu96scf_4_1040438_1040815	8.77	0.00	5.06	GL24479	5.04	3.32	3.96	0.97	0.0000	q<0.01
GaLu96scf_23_432457_432750	7.80	0.00	0.00	GL23057	4.76	6.03	5.72	-0.97	0.0000	q<0.01
GaLu96scf_1_3550729_3551062	8.61	0.00	0.00	GL30069	6.96	7.67	7.50	-0.97	0.0000	q<0.01

GaLu96scf_27_128806_129874	8.67	9.30	9.67	GL23383	5.06	4.28	4.14	-0.97	0.0000	q<0.01
GaLu96scf_19_436708_437006	8.18	0.00	0.00	GL22199	6.50	5.76	5.52	0.97	0.0000	q<0.01
GaLu96scf_1_2047995_2049134	10.85	0.00	0.00	GL30239	8.88	6.50	5.71	0.97	0.0000	q<0.01
GaLu96scf_1_2047995_2048234	8.91	0.00	0.00	GL30239	8.88	6.50	5.71	0.97	0.0000	q<0.01
GaLu96scf_34_95773_96416	10.70	4.60	0.00	GL24286	7.32	6.94	6.28	0.97	0.0000	q<0.01
GaLu96scf_4_620240_620393	6.81	4.60	5.90	GL24851	8.39	7.36	7.75	0.97	0.0000	q<0.01
GaLu96scf_4_740674_740930	9.12	0.00	0.00	GL24591	7.60	5.37	5.93	0.97	0.0000	q<0.01
GaLu96scf_13_937874_938103	7.74	0.00	0.00	GL21156	4.33	3.87	3.71	0.97	0.0000	q<0.01
GaLu96scf_11_567777_568108	13.51	0.00	0.00	GL26980	6.74	4.09	3.19	0.97	0.0000	q<0.01
GaLu96scf_1_2691071_2691374	8.80	0.00	0.00	GL30273	4.88	5.61	5.43	-0.97	0.0000	q<0.01
GaLu96scf_1_3585501_3585800	9.93	0.00	8.38	GL30712	5.59	7.10	6.18	-0.97	0.0000	q<0.01
GaLu96scf_4_581267_581650	7.54	0.00	0.00	GL24567	8.54	5.42	6.23	0.97	0.0000	q<0.01
GaLu96scf_9_1475386_1476375	7.54	0.00	0.00	GL26735	4.58	5.51	5.27	-0.97	0.0000	q<0.01
GaLu96scf_20_336853_337127	8.02	5.38	0.00	GL19024	3.95	3.81	2.22	0.97	0.0000	q<0.01
GaLu96scf_22_119250_119804	8.78	0.00	0.00	GL22968	3.98	5.14	5.56	-0.97	0.0000	q<0.01
GaLu96scf_22_119730_120074	7.80	0.00	0.00	GL22968	3.98	5.14	5.56	-0.97	0.0000	q<0.01
GaLu96scf_9_583208_583481	8.75	0.00	0.00	GL18154	4.30	1.35	2.14	0.97	0.0000	q<0.01
GaLu96scf_21_26613_26895	8.21	0.00	0.00	GL22826	4.39	2.75	3.18	0.97	0.0000	q<0.01
GaLu96scf_3_1917278_1917511	7.96	0.00	0.00	GL23706	5.79	5.38	5.23	0.97	0.0000	q<0.01
GaLu96scf_11_379444_380247	0.00	7.25	5.06	GL20847	4.11	5.35	5.30	0.97	0.0000	q<0.01
GaLu96scf_14_295185_295479	10.82	0.00	0.00	GL18545	4.40	2.83	2.24	0.97	0.0000	q<0.01
GaLu96scf_35_54893_55259	9.02	0.00	0.00	GL17140	9.84	4.75	2.84	0.96	0.0000	q<0.01
GaLu96scf_2_1579790_1580086	8.48	0.00	0.00	GL16044	7.93	4.80	3.62	0.96	0.0000	q<0.01
GaLu96scf_1_4437569_4437931	0.00	6.94	7.31	GL30807	2.24	1.19	1.44	-0.96	0.0000	q<0.01
GaLu96scf_31_120722_121354	8.86	5.38	7.52	GL24079	1.62	2.51	2.18	-0.96	0.0000	q<0.01

GaLu96scf_8_89554_90055	7.54	8.16	7.73	GL26030	5.76	7.06	6.47	0.96	0.0000	q<0.01
GaLu96scf_4_140106_140434	9.30	6.55	6.43	GL17243	6.41	5.08	5.40	0.96	0.0000	q<0.01
GaLu96scf_4_619405_619652	10.92	5.38	6.04	GL24851	8.39	7.36	7.75	0.96	0.0000	q<0.01
GaLu96scf_4_1128146_1128467	8.21	9.76	8.89	GL24752	1.33	3.69	2.92	0.96	0.0000	q<0.01
GaLu96scf_35_259014_259521	10.74	0.00	0.00	GL24331	5.40	7.64	7.02	-0.96	0.0000	q<0.01
GaLu96scf_1_4548752_4549297	8.76	0.00	0.00	GL30386	5.37	1.31	2.45	0.96	0.0000	q<0.01
GaLu96scf_6_33894_34253	6.81	5.38	6.32	GL31133	4.79	5.18	4.82	-0.96	0.0000	q<0.01
GaLu96scf_31_98237_98568	8.48	0.00	0.00	GL24047	2.44	4.08	3.62	-0.96	0.0000	q<0.01
GaLu96scf_7_527164_527536	9.82	5.38	6.53	GL20180	3.76	3.27	3.51	0.96	0.0000	q<0.01
GaLu96scf_12_837790_838612	8.48	0.00	0.00	GL20950	4.02	3.75	3.64	0.96	0.0000	q<0.01
GaLu96scf_15_817686_817952	8.74	0.00	0.00	GL21508	4.19	5.50	5.13	-0.96	0.0000	q<0.01
GaLu96scf_1_2168323_2168738	8.02	0.00	0.00	GL30250	7.35	8.02	7.83	-0.96	0.0000	q<0.01
GaLu96scf_5_1042559_1042864	9.51	6.94	6.96	GL25355	4.84	5.30	5.17	-0.96	0.0000	q<0.01
GaLu96scf_10_941606_941818	9.81	0.00	0.00	GL20528	10.66	8.09	7.06	0.96	0.0000	q<0.01
GaLu96scf_26_559095_559469	6.81	5.38	6.04	GL23344	2.79	3.65	3.04	-0.96	0.0000	q<0.01
GaLu96scf_12_953525_953752	7.96	0.00	0.00	GL20961	6.88	7.19	7.10	-0.96	0.0000	q<0.01
GaLu96scf_1_3045559_3045913	7.54	0.00	0.00	GL30656	4.69	6.95	6.31	-0.96	0.0000	q<0.01
GaLu96scf_31_371585_371932	7.80	0.00	0.00	GL24092	7.69	5.71	6.28	0.96	0.0000	q<0.01
GaLu96scf_3_1675827_1676107	12.16	5.38	7.61	GL23899	3.26	2.65	3.00	0.96	0.0000	q<0.01
GaLu96scf_12_716899_717730	7.74	0.00	0.00	GL21074	3.75	1.97	2.48	0.96	0.0000	q<0.01
GaLu96scf_11_518310_518605	8.02	0.00	0.00	GL20761	5.03	4.75	4.64	0.96	0.0000	q<0.01
GaLu96scf_21_35045_35886	11.12	6.02	4.77	GL22778	7.41	4.80	5.09	0.96	0.0000	q<0.01
GaLu96scf_2_313829_314427	9.78	7.94	0.00	GL22634	4.92	4.42	3.80	0.96	0.0000	q<0.01
GaLu96scf_4_869298_869525	7.54	0.00	0.00	GL24605	6.59	7.63	8.07	-0.96	0.0000	q<0.01
GaLu96scf_6_670719_670947	10.40	7.72	7.63	GL31438	6.36	8.06	7.61	-0.96	0.0000	q<0.01

GaLu96scf_26_547883_548228	0.00	6.94	7.88	GL23326	1.67	7.58	6.45	0.96	0.0000	q<0.01
GaLu96scf_8_541961_542452	7.48	0.00	0.00	GL17944	2.01	3.32	2.93	-0.96	0.0000	q<0.01
GaLu96scf_26_449079_449496	8.38	0.00	0.00	GL23341	6.77	5.77	5.34	0.96	0.0000	q<0.01
GaLu96scf_26_449240_449496	8.21	0.00	0.00	GL23341	6.77	5.77	5.34	0.96	0.0000	q<0.01
GaLu96scf_1_1533440_1533730	7.52	6.14	5.75	GL29359	5.16	4.54	3.95	0.96	0.0000	q<0.01
GaLu96scf_17_924788_925156	8.16	0.00	0.00	GL21972	6.09	4.88	4.36	0.96	0.0000	q<0.01
GaLu96scf_34_337457_337659	9.47	4.60	6.53	GL24263	4.33	3.57	3.66	0.96	0.0000	q<0.01
GaLu96scf_12_716899_717289	11.29	6.80	6.73	GL21074	3.75	1.97	2.48	0.96	0.0000	q<0.01
GaLu96scf_22_171118_171475	9.99	8.57	9.13	GL22965	4.52	5.42	5.31	-0.96	0.0000	q<0.01
GaLu96scf_4_148792_149052	9.94	8.19	8.02	GL24464	6.94	8.43	8.09	-0.96	0.0000	q<0.01
GaLu96scf_1_4204512_4204829	7.54	0.00	0.00	GL30112	6.35	8.29	7.68	-0.95	0.0000	q<0.01
GaLu96scf_1_441754_442359	0.00	6.87	4.77	GL30097	4.89	5.96	5.34	0.95	0.0000	q<0.01
GaLu96scf_9_1280373_1280724	8.21	5.38	0.00	GL26718	4.26	4.16	4.09	0.95	0.0000	q<0.01
GaLu96scf_1_4761471_4761677	10.47	5.38	0.00	GL29593	4.42	4.47	4.69	-0.95	0.0000	q<0.01
GaLu96scf_9_1425968_1426155	8.97	0.00	0.00	GL29184	7.82	7.54	7.41	0.95	0.0000	q<0.01
GaLu96scf_10_721197_721538	7.22	5.38	5.06	GL20553	2.76	1.33	1.61	0.95	0.0000	q<0.01
GaLu96scf_10_738185_738462	8.16	0.00	0.00	GL20637	1.66	2.01	2.17	-0.95	0.0000	q<0.01
GaLu96scf_3_704958_706056	9.12	0.00	0.00	GL23755	7.02	4.71	5.47	0.95	0.0000	q<0.01
GaLu96scf_7_1465011_1465205	0.00	0.00	7.73	GL28887	4.59	5.52	7.42	0.95	0.0000	q<0.01
GaLu96scf_2_1629480_1629720	7.54	0.00	0.00	GL22368	6.89	6.55	6.39	0.95	0.0000	q<0.01
GaLu96scf_15_58153_58592	9.12	0.00	0.00	GL18595	4.71	4.16	4.34	0.95	0.0000	q<0.01
GaLu96scf_2_1297860_1298666	5.25	7.19	7.75	GL16020	5.70	3.56	3.83	-0.95	0.0000	q<0.01
GaLu96scf_8_1086287_1086584	8.53	0.00	0.00	GL25820	5.05	7.05	6.38	-0.94	0.0000	q<0.01
GaLu96scf_8_1085842_1086215	7.54	0.00	0.00	GL25820	5.05	7.05	6.38	-0.94	0.0000	q<0.01
GaLu96scf_3_1674984_1675525	7.54	0.00	6.73	GL23899	3.26	2.65	3.00	0.94	0.0000	q<0.01

GaLu96scf_13_1106880_1107339	7.80	0.00	0.00	GL21215	7.04	5.28	4.39	0.94	0.0000	q<0.01
GaLu96scf_9_819996_820255	7.52	5.38	5.75	GL26562	3.19	4.48	3.87	-0.94	0.0000	q<0.01
GaLu96scf_27_499582_499912	7.54	0.00	0.00	GL23465	6.72	5.67	5.13	0.94	0.0000	q<0.01
GaLu96scf_17_538649_538861	7.76	5.38	5.75	GL15763	2.41	3.03	2.74	-0.94	0.0000	q<0.01
GaLu96scf_11_433670_433872	8.02	0.00	0.00	GL20856	4.68	6.17	5.66	-0.94	0.0000	q<0.01
GaLu96scf_2_1541899_1542100	7.16	5.38	4.77	GL22569	2.84	3.44	3.37	-0.94	0.0000	q<0.01
GaLu96scf_7_847373_848067	13.88	8.90	7.61	GL25620	10.42	6.98	7.53	0.94	0.0000	q<0.01
GaLu96scf_5_985045_985501	8.16	0.00	0.00	GL25266	2.62	3.70	3.33	-0.94	0.0000	q<0.01
GaLu96scf_21_34416_34722	11.68	0.00	5.06	GL22778	7.41	4.80	5.09	0.94	0.0000	q<0.01
GaLu96scf_18_226876_227010	10.62	0.00	0.00	GL21985	8.99	7.72	8.16	0.94	0.0000	q<0.01
GaLu96scf_18_382504_382751	9.50	6.55	0.00	GL21997	5.25	4.93	4.72	0.94	0.0000	q<0.01
GaLu96scf_3_1140214_1140529	9.01	8.71	8.69	GL23798	5.69	6.84	7.60	-0.94	0.0000	q<0.01
GaLu96scf_8_863122_863358	16.57	8.40	10.80	GL25929	7.63	5.54	5.37	0.94	0.0000	q<0.01
GaLu96scf_6_963910_964283	8.38	5.38	4.77	GL31622	6.78	6.00	5.22	0.93	0.0000	q<0.01
GaLu96scf_9_1521817_1522123	8.51	0.00	0.00	GL26620	5.74	5.24	4.96	0.93	0.0000	q<0.01
GaLu96scf_1_1822874_1823210	11.07	0.00	0.00	GL29687	4.12	5.03	5.55	-0.93	0.0000	q<0.01
GaLu96scf_18_567176_568351	0.00	8.00	6.62	GL27401	1.39	2.23	2.46	0.93	0.0000	q<0.01
GaLu96scf_1_4323938_4324354	10.53	9.13	8.69	GL30121	6.60	8.00	7.78	-0.93	0.0000	q<0.01
GaLu96scf_14_474829_475175	7.48	6.14	4.77	GL21351	5.20	5.07	4.37	0.93	0.0000	q<0.01
GaLu96scf_2_1463452_1463671	10.60	6.14	7.58	GL22567	3.63	4.33	3.87	-0.93	0.0000	q<0.01
GaLu96scf_8_531717_531891	10.06	0.00	0.00	GL25996	5.72	5.11	4.75	0.93	0.0000	q<0.01
GaLu96scf_4_1459868_1460308	5.25	6.55	5.75	GL17270	4.99	3.47	3.90	-0.93	0.0000	q<0.01
GaLu96scf_4_1040337_1040815	10.46	0.00	0.00	GL24479	5.04	3.32	3.96	0.93	0.0000	q<0.01
GaLu96scf_31_234356_234749	9.72	0.00	0.00	GL28294	5.82	4.56	5.03	0.93	0.0000	q<0.01
GaLu96scf_3_1675305_1676107	7.50	0.00	7.05	GL23899	3.26	2.65	3.00	0.93	0.0000	q<0.01

GaLu96scf_4_619131_620137	7.54	0.00	0.00	GL24851	8.39	7.36	7.75	0.93	0.0000	q<0.01
GaLu96scf_12_750458_750903	7.80	0.00	0.00	GL15327	3.87	6.38	5.44	-0.93	0.0000	q<0.01
GaLu96scf_19_270134_270627	9.79	6.55	6.89	GL22252	5.21	4.85	4.70	0.93	0.0000	q<0.01
GaLu96scf_50_82315_82708	7.80	0.00	0.00	GL25480	0.00	0.30	0.48	-0.93	0.0000	q<0.01
GaLu96scf_17_309529_310109	9.89	0.00	0.00	GL15753	8.53	7.43	6.76	0.93	0.0000	q<0.01
GaLu96scf_1_4023593_4023916	8.38	0.00	7.61	GL30771	5.15	6.07	5.57	-0.93	0.0000	q<0.01
GaLu96scf_14_125920_126881	7.80	6.36	5.75	GL21387	6.25	3.41	3.70	0.93	0.0000	q<0.01
GaLu96scf_16_961743_962077	7.96	0.00	0.00	GL21717	5.26	6.36	5.93	-0.92	0.0000	q<0.01
GaLu96scf_6_1422199_1422635	8.80	0.00	4.77	GL31393	9.04	5.58	6.23	0.92	0.0000	q<0.01
GaLu96scf_19_250245_250455	12.69	8.81	10.35	GL22149	6.68	8.43	8.42	-0.92	0.0000	q<0.01
GaLu96scf_15_791486_791712	9.65	6.55	8.47	GL21542	3.77	5.30	4.89	-0.92	0.0000	q<0.01
GaLu96scf_1_213969_214288	11.05	0.00	4.77	GL30236	6.84	3.53	6.14	0.92	0.0000	q<0.01
GaLu96scf_27_318775_318952	7.80	0.00	5.06	GL23418	5.23	2.64	3.41	0.92	0.0000	q<0.01
GaLu96scf_5_640216_640543	9.02	0.00	0.00	GL25243	3.03	2.70	2.49	0.92	0.0000	q<0.01
GaLu96scf_1_2460056_2460446	7.80	0.00	0.00	GL29985	9.45	8.74	8.28	0.92	0.0000	q<0.01
GaLu96scf_1_2457103_2457612	8.48	0.00	0.00	GL30586	9.45	8.74	8.28	0.92	0.0000	q<0.01
GaLu96scf_46_78604_78861	5.19	8.16	0.00	GL25091	3.11	2.96	3.17	-0.92	0.0000	q<0.01
GaLu96scf_9_729466_729718	8.49	8.24	8.35	GL26645	4.79	4.24	4.68	0.92	0.0000	q<0.01
GaLu96scf_11_185670_186176	9.66	6.02	4.77	GL20738	4.44	5.76	5.54	-0.92	0.0000	q<0.01
GaLu96scf_19_99310_99744	8.90	6.36	0.00	GL22136	6.09	6.21	5.32	0.92	0.0000	q<0.01
GaLu96scf_1_3217665_3218130	7.48	0.00	6.62	GL30326	6.40	6.98	6.68	-0.92	0.0000	q<0.01
GaLu96scf_40_145087_145321	6.75	0.00	5.75	GL17382	2.05	3.68	2.90	-0.92	0.0000	q<0.01
GaLu96scf_43_149820_150177	9.47	0.00	0.00	GL24996	2.43	1.06	1.61	0.92	0.0000	q<0.01
GaLu96scf_7_148441_148680	10.34	7.19	8.36	GL25633	7.29	9.26	9.35	-0.92	0.0000	q<0.01
GaLu96scf_31_202028_202379	9.19	0.00	0.00	GL24088	7.77	4.29	5.70	0.92	0.0000	q<0.01

GaLu96scf_16_623035_623891	8.53	4.60	5.75	GL21694	3.13	3.02	3.09	0.91	0.0000	q<0.01
GaLu96scf_3_586110_586630	9.15	5.57	0.00	GL23736	6.85	6.55	6.46	0.91	0.0000	q<0.01
GaLu96scf_28_502114_502375	8.33	0.00	0.00	GL23507	1.40	1.22	1.09	0.91	0.0000	q<0.01
GaLu96scf_2_2184361_2184635	9.68	0.00	0.00	GL22396	3.69	2.13	2.77	0.91	0.0000	q<0.01
GaLu96scf_6_356445_356910	9.12	0.00	0.00	GL31415	6.83	7.99	7.51	-0.91	0.0000	q<0.01
GaLu96scf_34_45294_45620	7.75	7.76	5.06	GL24242	3.81	4.02	3.52	0.91	0.0000	q<0.01
GaLu96scf_1_4599953_4601526	7.54	0.00	0.00	GL29585	5.90	2.69	4.02	0.91	0.0000	q<0.01
GaLu96scf_11_611428_611980	0.00	8.13	0.00	GL20868	3.51	2.70	3.18	-0.91	0.0000	q<0.01
GaLu96scf_4_863734_864566	9.79	6.55	0.00	GL17361	6.59	7.63	8.07	-0.91	0.0000	q<0.01
GaLu96scf_1_3483992_3484226	7.54	0.00	0.00	GL30057	9.76	8.42	7.46	0.91	0.0000	q<0.01
GaLu96scf_1_2047995_2048512	9.66	6.14	0.00	GL30239	8.88	6.50	5.71	0.91	0.0000	q<0.01
GaLu96scf_2_724504_724771	11.56	4.60	7.03	GL22518	7.52	4.87	4.58	0.90	0.0000	q<0.01
GaLu96scf_37_231727_232145	11.12	8.16	8.66	GL17179	6.68	7.93	7.22	-0.90	0.0000	q<0.01
GaLu96scf_10_1309498_1309814	9.16	8.90	8.11	GL20477	4.41	3.94	3.68	0.90	0.0000	q<0.01
GaLu96scf_13_363373_363611	9.24	0.00	0.00	GL21117	6.13	8.19	7.29	-0.90	0.0000	q<0.01
GaLu96scf_13_362795_363611	7.54	0.00	0.00	GL21117	6.13	8.19	7.29	-0.90	0.0000	q<0.01
GaLu96scf_7_851851_852235	9.72	5.57	0.00	GL25671	10.47	10.46	11.04	-0.90	0.0000	q<0.01
GaLu96scf_15_561186_562040	9.02	0.00	0.00	GL18632	5.28	3.00	4.00	0.90	0.0000	q<0.01
GaLu96scf_15_561186_561534	7.54	0.00	0.00	GL18632	5.28	3.00	4.00	0.90	0.0000	q<0.01
GaLu96scf_17_470660_470904	8.61	0.00	0.00	GL21829	5.56	3.31	4.30	0.90	0.0000	q<0.01
GaLu96scf_41_156524_157254	10.46	9.11	7.37	GL28597	3.64	3.64	3.12	0.90	0.0000	q<0.01
GaLu96scf_13_661306_661700	0.00	8.35	0.00	GL21135	6.08	6.67	6.34	0.90	0.0000	q<0.01
GaLu96scf_4_138240_138550	10.54	6.94	9.27	GL24734	6.41	5.08	5.40	0.90	0.0000	q<0.01
GaLu96scf_18_617342_617818	0.00	7.63	6.32	GL27410	2.67	4.36	3.37	0.90	0.0000	q<0.01
GaLu96scf_4_1470917_1471133	7.76	0.00	0.00	GL24779	6.52	5.55	4.77	0.90	0.0000	q<0.01

GaLu96scf_1_1891179_1891452	9.91	7.50	7.52	GL29690	3.69	3.26	2.91	0.90	0.0000	q<0.01
GaLu96scf_19_48561_48872	7.74	6.36	4.77	GL22232	5.69	4.40	4.23	0.90	0.0000	q<0.01
GaLu96scf_45_69519_69787	9.24	0.00	0.00	GL25070	4.44	2.04	3.12	0.89	0.0000	q<0.01
GaLu96scf_6_1278057_1278485	9.18	13.01	12.22	GL31640	4.28	4.86	5.08	0.89	0.0000	q<0.01
GaLu96scf_25_314155_314449	8.21	0.00	0.00	GL16604	0.71	2.26	1.57	-0.89	0.0000	q<0.01
GaLu96scf_19_175500_176092	11.21	0.00	4.77	GL22184	5.73	2.79	2.69	0.89	0.0000	q<0.01
GaLu96scf_1_2406810_2407749	0.00	6.87	0.00	GL29974	3.33	4.46	3.84	0.89	0.0000	q<0.01
GaLu96scf_7_847373_847794	14.36	4.60	0.00	GL25620	10.42	6.98	7.53	0.89	0.0000	q<0.01
GaLu96scf_20_474304_475276	8.48	0.00	0.00	GL22753	1.94	3.44	2.76	-0.89	0.0000	q<0.01
GaLu96scf_16_964984_965284	9.38	0.00	6.73	GL15708	5.09	4.89	4.95	0.89	0.0000	q<0.01
GaLu96scf_1_1832393_1833145	8.02	0.00	0.00	GL30917	4.60	5.09	5.50	-0.89	0.0000	q<0.01
GaLu96scf_5_683753_684258	8.77	7.01	5.90	GL17625	4.46	5.49	5.40	-0.89	0.0000	q<0.01
GaLu96scf_10_991858_992304	9.11	0.00	5.06	GL20560	6.73	8.19	8.00	-0.89	0.0000	q<0.01
GaLu96scf_15_312810_313365	7.54	0.00	0.00	GL21534	7.87	2.79	5.11	0.89	0.0000	q<0.01
GaLu96scf_1_3517326_3517680	0.00	9.03	7.05	GL29481	3.17	6.93	8.31	0.89	0.0000	q<0.01
GaLu96scf_3_1654376_1654824	10.09	0.00	0.00	GL23811	4.28	2.59	3.37	0.89	0.0000	q<0.01
GaLu96scf_42_145252_145529	8.80	5.57	6.53	GL17424	5.29	6.57	6.88	-0.89	0.0000	q<0.01
GaLu96scf_26_136975_137250	10.93	8.30	9.52	GL23327	3.24	3.72	3.72	-0.89	0.0000	q<0.01
GaLu96scf_6_284761_285099	8.38	6.80	7.67	GL31157	1.25	2.14	2.05	-0.89	0.0000	q<0.01
GaLu96scf_1_3450815_3451106	8.78	0.00	0.00	GL30704	8.03	7.13	6.34	0.88	0.0000	q<0.01
GaLu96scf_16_369107_369503	7.48	0.00	5.90	GL21747	3.57	5.07	4.53	-0.88	0.0000	q<0.01
GaLu96scf_13_1104139_1104490	8.49	6.02	7.18	GL21214	1.36	4.19	4.20	-0.88	0.0000	q<0.01
GaLu96scf_29_50957_51214	9.35	6.14	7.88	GL23547	5.01	6.53	6.39	-0.88	0.0000	q<0.01
GaLu96scf_9_820471_820700	7.48	0.00	0.00	GL26562	3.19	4.48	3.87	-0.88	0.0000	q<0.01
GaLu96scf_8_1022209_1023370	0.00	8.52	0.00	GL25822	4.07	3.58	4.53	-0.88	0.0000	q<0.01

GaLu96scf_1_1832017_1833145	5.25	5.38	6.89	GL30917	4.60	5.09	5.50	0.88	0.0000	q<0.01
GaLu96scf_7_847373_847936	13.04	4.60	0.00	GL25620	10.42	6.98	7.53	0.88	0.0000	q<0.01
GaLu96scf_18_884414_884741	10.83	0.00	0.00	GL22034	5.27	2.03	3.60	0.87	0.0000	q<0.01
GaLu96scf_13_438876_439167	13.14	7.30	10.65	GL21226	6.66	8.32	8.13	-0.87	0.0000	q<0.01
GaLu96scf_1_823157_823531	7.19	9.40	8.82	GL29633	1.43	1.91	2.08	0.87	0.0000	q<0.01
GaLu96scf_2_993116_993709	8.18	7.68	7.53	GL22678	2.33	2.17	1.84	0.87	0.0000	q<0.01
GaLu96scf_19_649330_649603	11.52	6.72	8.55	GL22217	8.43	9.61	9.80	-0.87	0.0000	q<0.01
GaLu96scf_6_1278284_1278485	6.81	5.38	5.75	GL31640	4.28	4.86	5.08	-0.87	0.0000	q<0.01
GaLu96scf_6_1347523_1348145	0.00	7.25	4.77	GL31148	7.81	5.61	5.10	-0.87	0.0000	q<0.01
GaLu96scf_29_326308_326624	8.16	0.00	0.00	GL23585	5.71	6.17	5.94	-0.87	0.0000	q<0.01
GaLu96scf_31_235519_235921	10.42	6.72	6.04	GL28294	5.82	4.56	5.03	0.86	0.0000	q<0.01
GaLu96scf_47_37444_38068	7.22	0.00	5.06	GL28679	7.03	7.26	6.95	-0.86	0.0000	q<0.01
GaLu96scf_31_376798_377183	8.61	5.57	5.75	GL24119	5.54	3.78	4.75	0.86	0.0000	q<0.01
GaLu96scf_1_407026_407234	9.58	0.00	5.06	GL30095	6.60	7.31	6.57	-0.86	0.0000	q<0.01
GaLu96scf_21_181854_182048	10.21	7.01	6.96	GL22829	7.01	7.45	7.23	-0.86	0.0000	q<0.01
GaLu96scf_46_40959_41378	9.53	0.00	0.00	GL28673	6.26	3.67	5.00	0.86	0.0000	q<0.01
GaLu96scf_23_615904_616279	11.65	8.22	7.61	GL16483	1.57	0.49	0.92	0.86	0.0000	q<0.01
GaLu96scf_5_36393_36732	12.72	7.76	7.35	GL25151	6.53	6.25	5.87	0.86	0.0000	q<0.01
GaLu96scf_1_441144_441919	6.81	4.60	4.77	GL30097	4.89	5.96	5.34	-0.85	0.0000	q<0.01
GaLu96scf_2_1541899_1545769	6.81	6.94	7.05	GL22569	2.84	3.44	3.37	0.85	0.0000	q<0.01
GaLu96scf_16_257810_258051	7.96	4.60	0.00	GL21632	6.37	7.58	7.67	-0.85	0.0000	q<0.01
GaLu96scf_29_246147_246434	9.84	0.00	6.73	GL23564	5.56	5.86	5.80	-0.85	0.0000	q<0.01
GaLu96scf_18_621638_622038	12.70	7.68	0.00	GL22024	6.45	4.91	4.73	0.85	0.0000	q<0.01
GaLu96scf_1_4020862_4021597	0.00	7.72	7.53	GL30770	5.15	6.07	5.57	0.85	0.0000	q<0.01
GaLu96scf_4_119002_119433	7.74	0.00	0.00	GL24469	5.16	4.86	4.52	0.85	0.0000	q<0.01

GaLu96scf_22_124704_125002	9.94	6.14	0.00	GL22915	5.00	3.18	2.95	0.85	0.0000	q<0.01
GaLu96scf_9_782358_782812	7.80	0.00	0.00	GL26651	6.85	7.67	7.23	-0.85	0.0000	q<0.01
GaLu96scf_28_525829_526191	8.76	0.00	6.32	GL23538	4.61	4.42	4.46	0.85	0.0000	q<0.01
GaLu96scf_26_186682_187294	7.80	0.00	0.00	GL19316	4.73	5.59	5.13	-0.85	0.0000	q<0.01
GaLu96scf_5_356471_356897	8.16	0.00	0.00	GL25417	5.35	4.46	3.41	0.84	0.0000	q<0.01
GaLu96scf_21_737969_738131	7.22	0.00	5.06	GL19109	0.00	7.68	6.21	-0.84	0.0000	q<0.01
GaLu96scf_1_3622004_3622288	7.54	4.60	7.05	GL30076	6.59	7.85	7.44	-0.84	0.0000	q<0.01
GaLu96scf_17_538649_539229	6.81	0.00	6.89	GL15763	2.41	3.03	2.74	-0.84	0.0000	q<0.01
GaLu96scf_1_559682_559969	7.96	0.00	0.00	GL30394	0.00	2.74	6.01	-0.84	0.0000	q<0.01
GaLu96scf_16_216972_217600	7.54	0.00	0.00	GL21685	6.16	5.42	4.52	0.84	0.0000	q<0.01
GaLu96scf_20_272630_272957	7.18	0.00	6.73	GL22708	2.92	5.04	4.18	-0.84	0.0000	q<0.01
GaLu96scf_16_607479_608085	11.77	11.70	11.27	GL21693	4.04	3.81	3.69	0.84	0.0000	q<0.01
GaLu96scf_21_181527_182048	11.89	9.76	9.58	GL22829	7.01	7.45	7.23	-0.83	0.0000	q<0.01
GaLu96scf_34_336501_336980	10.54	8.97	7.58	GL24263	4.33	3.57	3.66	0.83	0.0000	q<0.01
GaLu96scf_6_569531_569725	7.22	5.38	0.00	GL31314	5.51	5.50	5.54	-0.83	0.0000	q<0.01
GaLu96scf_45_24180_24572	7.99	0.00	6.04	GL28663	2.85	4.69	4.29	-0.82	0.0000	q<0.01
GaLu96scf_5_681470_681784	11.31	0.00	7.31	GL17624	6.54	5.20	5.33	0.82	0.0000	q<0.01
GaLu96scf_2_1262267_1262709	0.00	0.00	8.90	GL22343	3.79	5.90	7.49	0.82	0.0000	q<0.01
GaLu96scf_13_62664_63412	7.80	0.00	0.00	GL27080	7.00	4.19	5.80	0.82	0.0000	q<0.01
GaLu96scf_13_70956_71305	7.80	0.00	0.00	GL21093	7.00	4.19	5.80	0.82	0.0000	q<0.01
GaLu96scf_13_62206_62569	8.74	0.00	0.00	GL27080	7.00	4.19	5.80	0.82	0.0000	q<0.01
GaLu96scf_13_66924_67136	8.48	0.00	0.00	GL21093	7.00	4.19	5.80	0.82	0.0000	q<0.01
GaLu96scf_27_564880_565267	0.00	7.68	0.00	GL23463	7.30	7.61	6.88	0.82	0.0000	q<0.01
GaLu96scf_15_894656_895046	7.22	8.35	7.61	GL21512	5.95	3.83	4.04	-0.82	0.0000	q<0.01
GaLu96scf_3_1674340_1675076	9.30	0.00	0.00	GL23899	3.26	2.65	3.00	0.82	0.0000	q<0.01

GaLu96scf_3_1674340_1674803	8.66	0.00	0.00	GL23899	3.26	2.65	3.00	0.82	0.0000	q<0.01
GaLu96scf_5_683753_684778	9.58	8.13	6.62	GL17625	4.46	5.49	5.40	-0.82	0.0000	q<0.01
GaLu96scf_1_1292918_1293336	10.53	6.02	0.00	GL29863	6.41	5.06	5.07	0.82	0.0000	q<0.01
GaLu96scf_3_711836_712165	15.57	7.13	8.60	GL23962	6.15	5.01	4.11	0.82	0.0000	q<0.01
GaLu96scf_4_139725_139975	7.22	0.00	5.75	GL17243	6.41	5.08	5.40	0.81	0.0000	q<0.01
GaLu96scf_8_1393722_1393919	7.54	0.00	0.00	GL25852	5.36	5.98	5.62	-0.81	0.0000	q<0.01
GaLu96scf_1_89101_89611	10.78	9.37	7.35	GL30875	5.06	5.91	5.91	-0.81	0.0000	q<0.01
GaLu96scf_1_84704_84952	8.02	0.00	0.00	GL30422	5.32	2.13	4.00	0.81	0.0000	q<0.01
GaLu96scf_1_85022_85476	7.80	0.00	0.00	GL30422	5.32	2.13	4.00	0.81	0.0000	q<0.01
GaLu96scf_1_83631_83927	8.16	0.00	0.00	GL30422	5.32	2.13	4.00	0.81	0.0000	q<0.01
GaLu96scf_1_4115967_4116481	8.67	0.00	0.00	GL29535	4.48	1.43	3.23	0.81	0.0000	q<0.01
GaLu96scf_2_1056816_1057245	7.78	6.36	5.75	GL22329	8.99	8.97	8.87	0.81	0.0000	q<0.01
GaLu96scf_12_667804_668296	9.38	0.00	5.06	GL21067	3.82	4.33	4.37	-0.81	0.0000	q<0.01
GaLu96scf_19_664017_664332	10.92	6.94	0.00	GL22212	6.74	7.89	7.95	-0.80	0.0000	q<0.01
GaLu96scf_11_714664_715040	8.51	0.00	6.04	GL20771	2.63	4.39	4.16	-0.80	0.0000	q<0.01
GaLu96scf_15_573883_574306	8.48	0.00	0.00	GL21586	3.72	3.58	3.38	0.80	0.0000	q<0.01
GaLu96scf_6_1450153_1450393	7.54	0.00	0.00	GL31536	6.66	9.58	7.81	-0.80	0.0000	q<0.01
GaLu96scf_12_810175_810526	8.99	0.00	7.77	GL20955	5.56	6.23	6.05	-0.80	0.0000	q<0.01
GaLu96scf_4_1411238_1411498	7.80	0.00	0.00	GL24783	3.56	3.88	4.38	-0.80	0.0000	q<0.01
GaLu96scf_5_10565_10871	10.51	0.00	6.73	GL25146	5.63	2.94	3.04	0.80	0.0000	q<0.01
GaLu96scf_1_344308_344528	7.16	0.00	4.77	GL30986	2.02	2.84	2.78	-0.79	0.0000	q<0.01
GaLu96scf_23_535599_536301	7.80	0.00	5.06	GL27854	2.99	4.25	4.20	-0.79	0.0000	q<0.01
GaLu96scf_7_847536_847794	11.00	5.38	0.00	GL25620	10.42	6.98	7.53	0.79	0.0000	q<0.01
GaLu96scf_1_2346905_2347327	8.02	6.36	7.18	GL30566	4.49	4.92	4.42	-0.79	0.0000	q<0.01
GaLu96scf_9_820471_820877	10.50	8.71	8.33	GL26562	3.19	4.48	3.87	-0.79	0.0000	q<0.01

GaLu96scf_46_79582_80089	7.74	0.00	4.77	GL25091	3.11	2.96	3.17	0.79	0.0000	q<0.01
GaLu96scf_31_39627_39859	13.88	10.05	10.42	GL24100	9.14	9.62	10.20	-0.79	0.0000	q<0.01
GaLu96scf_1_441144_441430	7.96	6.02	5.90	GL30097	4.89	5.96	5.34	-0.78	0.0000	q<0.01
GaLu96scf_13_438564_439167	9.08	0.00	6.62	GL21226	6.66	8.32	8.13	-0.78	0.0000	q<0.01
GaLu96scf_7_1416763_1417203	7.96	6.02	7.05	GL25706	4.52	6.15	6.40	-0.78	0.0000	q<0.01
GaLu96scf_32_269704_269946	11.69	0.00	6.04	GL24170	9.25	5.18	4.46	0.78	0.0000	q<0.01
GaLu96scf_21_814520_814865	10.14	0.00	6.04	GL16370	2.21	1.38	1.33	0.78	0.0000	q<0.01
GaLu96scf_15_528104_528366	9.33	0.00	4.77	GL21580	8.25	5.55	5.05	0.78	0.0000	q<0.01
GaLu96scf_4_1279082_1279396	13.78	8.71	9.63	GL24762	4.14	3.80	3.46	0.77	0.0000	q<0.01
GaLu96scf_13_1002066_1002338	9.21	5.38	0.00	GL21206	3.87	5.27	5.16	-0.77	0.0000	q<0.01
GaLu96scf_1_3968929_3969173	11.60	0.00	5.75	GL29512	7.09	3.76	3.02	0.77	0.0000	q<0.01
GaLu96scf_6_871714_871951	7.74	0.00	0.00	GL31341	7.50	8.48	7.85	-0.77	0.0000	q<0.01
GaLu96scf_24_18964_19388	9.21	0.00	0.00	GL23161	5.35	4.17	1.90	0.76	0.0000	q<0.01
GaLu96scf_9_150313_150641	8.16	5.38	8.11	GL26588	3.54	4.73	4.34	-0.76	0.0000	q<0.01
GaLu96scf_11_101360_101610	10.55	0.00	7.05	GL20660	4.62	3.40	3.41	0.76	0.0000	q<0.01
GaLu96scf_3_1178883_1179123	8.38	0.00	4.77	GL23655	3.46	2.14	1.97	0.76	0.0000	q<0.01
GaLu96scf_8_498907_499140	0.00	7.94	0.00	GL25895	3.43	3.90	3.74	0.76	0.0000	q<0.01
GaLu96scf_1_835280_835527	8.33	6.36	4.77	GL30851	5.08	4.30	4.50	0.76	0.0000	q<0.01
GaLu96scf_4_169756_169975	8.96	5.38	0.00	GL28477	5.82	4.76	4.85	0.76	0.0000	q<0.01
GaLu96scf_4_942652_942888	6.23	0.00	7.29	GL17366	3.10	2.54	2.79	0.75	0.0000	q<0.01
GaLu96scf_8_1061304_1061968	0.00	0.00	7.05	GL25824	2.26	2.57	2.11	-0.75	0.0000	q<0.01
GaLu96scf_8_1061304_1061639	0.00	0.00	7.42	GL25824	2.26	2.57	2.11	-0.75	0.0000	q<0.01
GaLu96scf_9_1106364_1106552	7.74	0.00	5.75	GL26480	7.75	6.72	6.81	0.75	0.0000	q<0.01
GaLu96scf_31_140867_141452	7.80	0.00	4.77	GL17001	6.06	8.39	8.59	-0.75	0.0000	q<0.01
GaLu96scf_2_1544548_1545992	9.12	5.38	0.00	GL22569	2.84	3.44	3.37	-0.75	0.0000	q<0.01

GaLu96scf_31_264930_265127	10.85	0.00	0.00	GL24114	4.22	3.84	3.03	0.75	0.0000	q<0.01
GaLu96scf_9_1418381_1418731	8.48	0.00	0.00	GL26510	4.79	4.97	5.33	-0.75	0.0000	q<0.01
GaLu96scf_4_1782074_1782367	9.32	5.57	0.00	GL24804	6.66	6.08	7.92	-0.75	0.0000	q<0.01
GaLu96scf_31_140867_141632	8.17	0.00	5.06	GL17001	6.06	8.39	8.59	-0.75	0.0000	q<0.01
GaLu96scf_18_567176_567508	6.81	6.02	0.00	GL27401	1.39	2.23	2.46	-0.74	0.0000	q<0.01
GaLu96scf_17_309529_309938	12.53	7.87	9.52	GL15753	8.53	7.43	6.76	0.74	0.0000	q<0.01
GaLu96scf_23_535599_536009	9.30	0.00	6.73	GL27854	2.99	4.25	4.20	-0.74	0.0000	q<0.01
GaLu96scf_20_142419_143191	9.89	6.87	8.07	GL22680	4.58	6.52	4.34	-0.74	0.0000	q<0.01
GaLu96scf_9_841950_842346	0.00	6.87	0.00	GL26791	4.22	4.11	4.47	-0.74	0.0000	q<0.01
GaLu96scf_7_1167778_1168333	8.91	9.56	8.92	GL25544	1.17	4.67	3.63	0.73	0.0000	q<0.01
GaLu96scf_5_683753_684096	9.83	0.00	7.61	GL17625	4.46	5.49	5.40	-0.73	0.0000	q<0.01
GaLu96scf_4_988435_988869	9.99	0.00	6.73	GL24619	6.77	5.95	5.92	0.73	0.0000	q<0.01
GaLu96scf_1_2485437_2485732	7.80	0.00	0.00	GL29981	3.87	4.66	4.11	-0.73	0.0000	q<0.01
GaLu96scf_14_989202_990323	9.02	0.00	0.00	GL15529	6.20	5.91	6.11	0.73	0.0000	q<0.01
GaLu96scf_3_507235_507497	7.54	0.00	0.00	GL23732	5.71	6.61	5.97	-0.73	0.0000	q<0.01
GaLu96scf_7_455907_456236	9.73	5.57	0.00	GL25740	3.58	2.54	2.71	0.73	0.0000	q<0.01
GaLu96scf_15_889124_889844	8.02	4.60	0.00	GL21511	6.16	1.79	2.52	0.72	0.0000	q<0.01
GaLu96scf_8_779487_779954	7.16	5.38	0.00	GL26008	6.87	1.57	1.32	0.72	0.0000	q<0.01
GaLu96scf_42_145252_145819	7.22	6.46	0.00	GL17424	5.29	6.57	6.88	-0.72	0.0000	q<0.01
GaLu96scf_11_798658_799212	0.00	0.00	7.53	GL20726	5.95	6.31	5.80	-0.72	0.0000	q<0.01
GaLu96scf_4_638508_638817	8.00	0.00	0.00	GL24575	5.03	4.81	4.25	0.72	0.0000	q<0.01
GaLu96scf_18_226622_227010	13.00	5.38	0.00	GL21985	8.99	7.72	8.16	0.72	0.0000	q<0.01
GaLu96scf_9_729107_729718	0.00	7.25	6.62	GL26645	4.79	4.24	4.68	-0.71	0.0000	q<0.01
GaLu96scf_1_224262_224453	8.36	0.00	5.75	GL29930	7.41	5.60	5.52	0.71	0.0000	q<0.01
GaLu96scf_17_425284_425663	8.33	0.00	0.00	GL18762	3.10	2.63	2.97	0.71	0.0000	q<0.01

GaLu96scf_13_946765_947266	8.02	4.60	0.00	GL21157	4.10	3.22	3.38	0.71	0.0000	q<0.01
GaLu96scf_11_596697_596895	8.67	0.00	8.03	GL20763	6.78	7.27	7.17	-0.71	0.0000	q<0.01
GaLu96scf_47_89640_90331	12.03	8.67	0.00	GL25106	10.78	7.31	7.33	0.71	0.0000	q<0.01
GaLu96scf_4_331341_331861	8.53	0.00	0.00	GL17331	5.82	6.78	6.08	-0.71	0.0000	q<0.01
GaLu96scf_31_36002_36247	8.16	0.00	0.00	GL24099	3.09	4.87	3.57	-0.71	0.0000	q<0.01
GaLu96scf_11_730171_730635	10.89	7.55	0.00	GL20772	6.66	8.29	8.22	-0.71	0.0000	q<0.01
GaLu96scf_1_1744520_1744982	8.48	0.00	0.00	GL30217	7.90	9.59	8.36	-0.71	0.0000	q<0.01
GaLu96scf_18_509337_509757	7.96	0.00	5.75	GL15847	6.70	2.65	2.61	0.71	0.0000	q<0.01
GaLu96scf_19_528439_528683	0.00	0.00	7.05	GL22203	6.11	5.62	6.30	0.71	0.0000	q<0.01
GaLu96scf_2_923644_924029	10.18	8.38	6.32	GL22451	5.19	5.58	5.49	-0.71	0.0000	q<0.01
GaLu96scf_3_1135050_1135563	10.39	0.00	0.00	GL23647	3.74	3.49	2.79	0.71	0.0000	q<0.01
GaLu96scf_14_125920_126149	9.75	6.36	0.00	GL21387	6.25	3.41	3.70	0.70	0.0000	q<0.01
GaLu96scf_4_168642_169400	7.48	0.00	6.04	GL28477	5.82	4.76	4.85	0.70	0.0000	q<0.01
GaLu96scf_16_358893_359309	8.21	4.60	0.00	GL27298	3.04	2.51	2.62	0.70	0.0000	q<0.01
GaLu96scf_3_1179570_1180275	7.54	6.36	0.00	GL23655	3.46	2.14	1.97	0.70	0.0000	q<0.01
GaLu96scf_10_1363675_1363940	9.25	0.00	0.00	GL20592	3.54	3.46	3.23	0.70	0.0000	q<0.01
GaLu96scf_15_527991_528366	9.34	0.00	5.75	GL21580	8.25	5.55	5.05	0.70	0.0000	q<0.01
GaLu96scf_33_107955_108150	12.43	10.01	11.84	GL24214	3.36	3.01	3.01	0.69	0.0000	q<0.01
GaLu96scf_5_521739_521956	8.02	6.02	8.14	GL25231	2.35	4.41	3.80	-0.69	0.0000	q<0.01
GaLu96scf_9_864649_865380	7.16	0.00	6.04	GL26572	3.78	7.00	6.68	-0.69	0.0000	q<0.01
GaLu96scf_2_202079_202358	9.82	4.60	8.57	GL16088	4.40	4.61	4.61	-0.69	0.0000	q<0.01
GaLu96scf_16_604798_605067	10.69	6.87	0.00	GL21653	6.18	3.88	4.19	0.69	0.0000	q<0.01
GaLu96scf_15_821086_821358	7.54	0.00	0.00	GL21515	5.93	5.27	5.77	0.69	0.0000	q<0.01
GaLu96scf_15_824691_824989	8.02	0.00	0.00	GL21605	5.93	5.27	5.77	0.69	0.0000	q<0.01
GaLu96scf_3_1102021_1102243	9.21	0.00	7.88	GL23794	5.65	6.20	6.14	-0.69	0.0000	q<0.01

GaLu96scf_7_1167778_1168011	9.43	9.86	10.30	GL25544	1.17	4.67	3.63	0.68	0.0000	q<0.01
GaLu96scf_8_460253_460763	7.77	5.38	0.00	GL26103	6.79	7.47	7.41	-0.68	0.0000	q<0.01
GaLu96scf_3_1329424_1329647	7.80	0.00	4.77	GL19461	7.33	6.06	5.78	0.68	0.0000	q<0.01
GaLu96scf_7_644305_645029	0.00	6.87	0.00	GL25664	8.50	4.51	5.43	-0.68	0.0000	q<0.01
GaLu96scf_9_981635_982113	11.88	10.36	10.04	GL26582	4.04	4.00	3.41	0.68	0.0000	q<0.01
GaLu96scf_11_713891_714215	7.54	0.00	6.73	GL20771	2.63	4.39	4.16	-0.68	0.0000	q<0.01
GaLu96scf_17_71814_72193	8.21	0.00	6.04	GL21904	6.42	5.01	4.94	0.67	0.0000	q<0.01
GaLu96scf_7_1425350_1425884	7.80	0.00	4.77	GL25711	8.66	7.88	7.69	0.67	0.0000	q<0.01
GaLu96scf_5_596965_597421	7.54	0.00	0.00	GL17626	6.86	7.37	6.98	-0.67	0.0000	q<0.01
GaLu96scf_12_280250_280639	8.67	0.00	0.00	GL15296	5.14	1.04	4.24	0.67	0.0000	q<0.01
GaLu96scf_5_288195_288558	9.79	6.36	0.00	GL25300	7.34	9.52	9.17	-0.66	0.0000	q<0.01
GaLu96scf_28_526348_526653	10.72	6.36	0.00	GL23538	4.61	4.42	4.46	0.66	0.0000	q<0.01
GaLu96scf_47_129180_129519	9.38	0.00	7.18	GL25114	6.17	8.27	8.35	-0.66	0.0000	q<0.01
GaLu96scf_1_1046885_1047489	8.61	0.00	0.00	GL30887	6.56	6.46	6.05	0.66	0.0000	q<0.01
GaLu96scf_1_441534_441919	11.47	10.68	10.42	GL30097	4.89	5.96	5.34	-0.66	0.0000	q<0.01
GaLu96scf_7_932083_932292	7.16	0.00	5.06	GL25780	2.02	2.78	2.88	-0.65	0.0000	q<0.01
GaLu96scf_50_72806_73658	7.22	5.38	0.00	GL28773	0.00	1.10	1.02	-0.65	0.0000	q<0.01
GaLu96scf_1_1143289_1143591	9.06	5.38	0.00	GL29852	2.65	2.78	2.75	-0.65	0.0000	q<0.01
GaLu96scf_9_1059347_1059821	5.25	0.00	7.03	GL18105	3.56	5.56	4.73	-0.65	0.0000	q<0.01
GaLu96scf_30_183359_183598	8.33	4.60	0.00	GL28255	4.85	2.77	3.37	0.65	0.0000	q<0.01
GaLu96scf_12_1110164_1110488	9.46	8.77	7.67	GL18413	3.51	4.27	4.10	-0.64	0.0000	q<0.01
GaLu96scf_1_3923133_3923420	6.79	7.41	6.89	GL30361	2.95	4.56	4.52	0.64	0.0000	q<0.01
GaLu96scf_9_1035105_1035555	8.21	6.80	5.06	GL26477	3.19	5.60	4.88	-0.64	0.0000	q<0.01
GaLu96scf_9_636082_636429	7.22	0.00	5.06	GL26775	4.08	3.34	3.22	0.64	0.0000	q<0.01
GaLu96scf_10_956005_956405	9.71	0.00	0.00	GL20648	5.44	5.11	3.59	0.64	0.0000	q<0.01

GaLu96scf_4_1470480_1471133	5.19	7.41	0.00	GL24779	6.52	5.55	4.77	0.63	0.0000	q<0.01
GaLu96scf_45_66710_67086	8.38	0.00	4.77	GL17472	4.37	3.60	3.29	0.63	0.0000	q<0.01
GaLu96scf_42_187462_188053	7.54	0.00	5.75	GL28618	4.10	5.01	5.11	-0.62	0.0000	q<0.01
GaLu96scf_8_1080012_1080573	9.40	6.14	0.00	GL26045	5.31	7.34	6.89	-0.61	0.0000	q<0.01
GaLu96scf_19_616057_616293	8.87	5.57	8.72	GL22214	10.43	7.31	7.63	0.61	0.0000	q<0.01
GaLu96scf_15_583443_583687	7.22	0.00	5.90	GL31774	2.82	2.62	2.61	0.61	0.0000	q<0.01
GaLu96scf_30_120197_120467	8.48	0.00	0.00	GL24012	3.94	3.37	3.86	0.61	0.0000	q<0.01
GaLu96scf_21_298566_298876	7.96	5.38	0.00	GL19097	3.09	2.55	3.50	-0.60	0.0000	q<0.01
GaLu96scf_1_1832393_1832925	8.21	8.27	8.10	GL30917	4.60	5.09	5.50	-0.60	0.0000	q<0.01
GaLu96scf_24_643346_643626	15.82	7.41	9.31	GL23172	5.63	5.26	5.67	0.60	0.0000	q<0.01
GaLu96scf_6_55244_55589	0.00	5.57	6.62	GL31471	4.72	4.31	4.60	-0.60	0.0000	q<0.01
GaLu96scf_12_982987_983486	8.90	0.00	5.90	GL15342	5.64	4.75	4.49	0.60	0.0000	q<0.01
GaLu96scf_45_134109_134540	0.00	0.00	9.48	GL25065	3.95	5.33	5.54	0.60	0.0000	q<0.01
GaLu96scf_25_71903_72224	10.52	0.00	0.00	GL23234	5.54	4.99	5.47	0.60	0.0000	q<0.01
GaLu96scf_2_1056816_1057450	7.54	0.00	0.00	GL22329	8.99	8.97	8.87	0.60	0.0000	q<0.01
GaLu96scf_3_1113081_1113421	8.16	6.55	6.62	GL23795	2.47	3.90	2.59	-0.59	0.0001	q<0.01
GaLu96scf_1_2922218_2922676	13.23	8.90	9.46	GL30011	5.51	5.54	5.63	-0.58	0.0001	q<0.01
GaLu96scf_3_1654565_1654824	10.01	4.60	0.00	GL23811	4.28	2.59	3.37	0.58	0.0001	q<0.01
GaLu96scf_13_786042_786430	7.74	0.00	0.00	GL21149	5.94	5.85	4.96	0.57	0.0001	q<0.01
GaLu96scf_19_870608_870940	7.54	0.00	5.75	GL22225	3.80	3.11	2.97	0.57	0.0001	q<0.01
GaLu96scf_17_937387_937830	0.00	8.81	9.10	GL18792	5.15	6.88	5.35	0.57	0.0001	q<0.01
GaLu96scf_27_339069_339598	10.83	0.00	7.42	GL23420	7.85	6.85	6.53	0.56	0.0001	q<0.01
GaLu96scf_4_82007_82257	7.48	0.00	0.00	GL17240	4.85	5.34	4.89	-0.56	0.0001	q<0.01
GaLu96scf_24_34110_34346	7.48	0.00	4.77	GL23132	0.23	1.21	1.62	-0.56	0.0001	q<0.01
GaLu96scf_2_1224538_1224876	11.77	9.62	10.32	GL22551	5.93	5.30	6.16	0.56	0.0001	q<0.01

GaLu96scf_5_288195_288837	7.20	5.57	0.00	GL25300	7.34	9.52	9.17	-0.56	0.0001	q<0.01
GaLu96scf_2_718009_718422	9.46	6.55	7.24	GL22440	5.12	5.01	4.74	0.55	0.0001	q<0.01
GaLu96scf_10_1396074_1396289	8.21	6.36	0.00	GL20588	7.74	9.66	9.34	-0.55	0.0001	q<0.01
GaLu96scf_16_26866_27197	7.16	4.60	0.00	GL27278	4.11	1.90	2.59	0.54	0.0001	q<0.01
GaLu96scf_13_716319_716608	7.22	5.57	0.00	GL21141	4.48	6.16	5.86	-0.54	0.0001	q<0.01
GaLu96scf_5_417693_418112	7.74	7.45	6.62	GL25223	3.96	6.36	5.85	-0.54	0.0001	q<0.01
GaLu96scf_11_1234416_1234903	0.00	6.87	0.00	GL20816	3.06	2.87	2.88	-0.54	0.0001	q<0.01
GaLu96scf_31_320750_320941	7.98	0.00	0.00	GL17009	2.31	1.46	2.27	0.54	0.0001	q<0.01
GaLu96scf_40_78231_78716	7.80	0.00	4.77	GL24887	2.87	2.04	1.59	0.54	0.0001	q<0.01
GaLu96scf_2_1034675_1034909	8.38	4.60	7.35	GL22327	4.00	5.23	5.61	-0.53	0.0001	q<0.01
GaLu96scf_12_387721_388104	8.51	9.02	8.67	GL21056	2.34	5.24	6.35	0.53	0.0001	q<0.01
GaLu96scf_3_1332003_1332585	7.80	6.94	0.00	GL16846	5.04	5.39	5.37	-0.53	0.0001	q<0.01
GaLu96scf_15_577641_578307	9.28	5.57	0.00	GL21539	6.60	6.08	6.98	-0.53	0.0001	q<0.01
GaLu96scf_1_1137382_1137856	0.00	7.13	6.73	GL30182	6.35	6.36	6.49	0.53	0.0001	q<0.01
GaLu96scf_9_1185508_1185778	7.80	0.00	0.00	GL26704	6.08	5.74	6.06	0.53	0.0001	q<0.01
GaLu96scf_22_187637_188188	6.81	8.69	6.73	GL22966	7.12	9.12	8.98	0.53	0.0001	q<0.01
GaLu96scf_7_207334_207925	0.00	6.87	0.00	GL25646	5.02	4.95	6.82	-0.53	0.0001	q<0.01
GaLu96scf_2_1057061_1057450	13.08	9.13	9.51	GL22329	8.99	8.97	8.87	0.52	0.0001	q<0.01
GaLu96scf_3_1899354_1899568	8.38	0.00	8.36	GL23921	8.76	8.78	8.06	-0.52	0.0001	q<0.01
GaLu96scf_12_449898_450282	10.27	7.94	8.30	GL18437	6.68	7.82	6.50	-0.52	0.0001	q<0.01
GaLu96scf_32_174746_175093	7.54	0.00	0.00	GL24139	3.54	3.18	3.53	0.51	0.0001	q<0.01
GaLu96scf_27_128508_128850	7.96	0.00	6.62	GL23383	5.06	4.28	4.14	0.51	0.0001	q<0.01
GaLu96scf_43_107810_108039	11.75	8.45	8.64	GL28634	3.99	5.94	3.90	-0.51	0.0001	q<0.01
GaLu96scf_5_621587_621966	9.72	0.00	0.00	GL25241	5.72	6.37	5.73	-0.51	0.0001	q<0.01
GaLu96scf_1_2496686_2497062	6.81	6.36	4.77	GL30578	4.46	4.87	4.35	0.50	0.0001	q<0.01

GaLu96scf_1_2575581_2575944	7.80	6.55	0.00	GL30598	4.68	5.07	4.61	0.50	0.0001	q<0.01
GaLu96scf_30_254564_256000	7.16	7.19	0.00	GL24029	1.72	3.26	3.26	-0.50	0.0001	q<0.01
GaLu96scf_22_119250_120074	10.46	0.00	7.71	GL22968	3.98	5.14	5.56	-0.50	0.0001	q<0.01
GaLu96scf_5_1382118_1382794	7.74	6.14	7.71	GL25382	7.77	5.61	5.55	0.50	0.0001	q<0.01
GaLu96scf_31_450431_450722	9.53	6.02	0.00	GL24131	5.28	7.60	6.73	-0.49	0.0001	q<0.01
GaLu96scf_1_3142092_3142483	7.22	5.57	0.00	GL30662	7.76	8.92	8.65	-0.49	0.0001	q<0.01
GaLu96scf_7_851952_852235	8.18	0.00	0.00	GL25671	10.47	10.46	11.04	-0.49	0.0001	q<0.01
GaLu96scf_41_157014_157254	8.38	7.36	8.35	GL28597	3.64	3.64	3.12	-0.49	0.0001	q<0.01
GaLu96scf_6_813635_813907	7.74	0.00	0.00	GL31615	5.49	6.03	5.48	-0.48	0.0001	q<0.01
GaLu96scf_8_1207123_1207684	0.00	8.35	0.00	GL20245	2.73	3.05	3.06	0.48	0.0002	q<0.01
GaLu96scf_25_479612_479927	0.00	0.00	7.37	GL23247	2.61	11.20	10.93	0.48	0.0002	q<0.01
GaLu96scf_17_301396_301681	9.48	0.00	0.00	GL21921	5.66	5.63	6.52	-0.47	0.0002	q<0.01
GaLu96scf_21_562743_563142	8.48	7.63	7.99	GL16341	3.35	5.41	1.65	-0.47	0.0002	q<0.01
GaLu96scf_1_2004521_2004822	11.91	8.19	8.52	GL30538	5.77	6.84	5.61	-0.47	0.0002	q<0.01
GaLu96scf_33_107500_108150	6.75	6.94	0.00	GL24214	3.36	3.01	3.01	0.47	0.0002	q<0.01
GaLu96scf_35_314043_314229	12.32	0.00	7.71	GL24340	8.98	7.29	6.13	0.47	0.0002	q<0.01
GaLu96scf_4_532307_532692	9.86	6.02	4.77	GL28557	3.75	4.74	3.96	-0.47	0.0002	q<0.01
GaLu96scf_9_1031117_1031333	0.00	0.00	7.05	GL26688	5.40	3.09	3.20	-0.46	0.0002	q<0.01
GaLu96scf_13_1074725_1075149	8.52	4.60	0.00	GL21210	4.44	3.89	4.84	-0.46	0.0002	q<0.01
GaLu96scf_9_864649_865042	8.91	7.25	9.20	GL26572	3.78	7.00	6.68	-0.46	0.0002	q<0.01
GaLu96scf_16_855760_856605	9.10	8.38	9.20	GL15699	1.95	2.31	4.20	0.46	0.0002	q<0.01
GaLu96scf_23_114407_115124	5.25	7.68	5.06	GL23036	3.72	2.41	2.44	-0.46	0.0002	q<0.01
GaLu96scf_4_168492_169034	6.81	0.00	8.00	GL28477	5.82	4.76	4.85	0.44	0.0002	q<0.01
GaLu96scf_1_3551960_3552335	7.54	6.14	0.00	GL30069	6.96	7.67	7.50	-0.44	0.0002	q<0.01
GaLu96scf_50_63748_64028	9.21	6.14	6.96	GL28774	5.97	6.30	5.80	-0.44	0.0002	q<0.01

GaLu96scf_1_3294448_3294739	8.98	0.00	5.06	GL31004	4.79	4.51	5.28	0.43	0.0002	q<0.01
GaLu96scf_18_532142_532405	0.00	0.00	7.05	GL22017	1.52	2.02	1.98	0.43	0.0002	q<0.01
GaLu96scf_1_822816_823309	8.37	8.13	8.85	GL29633	1.43	1.91	2.08	0.43	0.0003	q<0.01
GaLu96scf_13_562681_563010	8.53	0.00	7.53	GL18500	5.19	3.85	3.50	0.42	0.0003	q<0.01
GaLu96scf_24_642832_643626	7.50	0.00	0.00	GL23172	5.63	5.26	5.67	0.42	0.0003	q<0.01
GaLu96scf_12_649243_649552	9.55	0.00	7.75	GL21066	11.03	9.13	8.41	0.42	0.0003	q<0.01
GaLu96scf_16_856246_856605	0.00	7.68	5.75	GL15699	1.95	2.31	4.20	0.42	0.0003	q<0.01
GaLu96scf_25_426165_426869	10.43	7.72	0.00	GL31764	3.24	6.36	5.28	-0.42	0.0003	q<0.01
GaLu96scf_17_692008_692283	9.59	0.00	0.00	GL21953	5.04	5.06	4.91	0.41	0.0003	q<0.01
GaLu96scf_20_142419_143504	8.02	0.00	0.00	GL22680	4.58	6.52	4.34	-0.41	0.0003	q<0.01
GaLu96scf_3_1818467_1818647	10.14	7.63	9.05	GL23913	3.98	4.76	5.61	-0.41	0.0003	q<0.01
GaLu96scf_5_1100364_1100544	10.34	6.36	8.87	GL17543	6.08	6.34	5.40	-0.41	0.0003	q<0.01
GaLu96scf_19_819546_819912	6.23	6.14	0.00	GL22176	3.03	3.78	3.11	0.40	0.0003	q<0.01
GaLu96scf_3_495478_495826	5.25	6.14	8.14	GL23731	2.92	4.23	3.70	0.40	0.0003	q<0.01
GaLu96scf_5_686345_686732	8.77	0.00	0.00	GL25319	6.00	4.56	6.21	0.40	0.0004	q<0.01
GaLu96scf_9_517326_517556	7.96	0.00	0.00	GL26763	4.97	6.16	4.80	-0.40	0.0004	q<0.01
GaLu96scf_22_682319_682655	8.02	0.00	6.89	GL19186	4.06	5.57	6.09	-0.39	0.0004	q<0.01
GaLu96scf_2_775722_776014	7.80	0.00	0.00	GL18988	4.30	1.82	4.66	0.39	0.0004	q<0.01
GaLu96scf_14_997917_998788	6.81	8.27	0.00	GL21283	6.20	5.91	6.11	-0.39	0.0004	q<0.01
GaLu96scf_1_1525668_1525994	6.20	6.14	0.00	GL30903	2.62	2.91	2.65	0.39	0.0004	q<0.01
GaLu96scf_10_633173_633434	11.26	0.00	0.00	GL20627	4.94	5.43	4.86	-0.39	0.0004	q<0.01
GaLu96scf_1_2259921_2260257	7.22	5.38	0.00	GL30562	4.50	2.44	3.20	0.39	0.0004	q<0.01
GaLu96scf_51_90105_90342	0.00	5.38	7.31	GL25489	3.76	2.65	3.60	-0.39	0.0004	q<0.01
GaLu96scf_10_633036_633434	11.14	9.86	9.85	GL20627	4.94	5.43	4.86	-0.38	0.0004	q<0.01
GaLu96scf_9_1014924_1015283	8.00	0.00	6.32	GL26475	9.30	9.33	8.88	-0.38	0.0004	q<0.01

GaLu96scf_2_1057061_1057245	14.58	9.23	10.60	GL22329	8.99	8.97	8.87	0.38	0.0004	q<0.01
GaLu96scf_1_613208_613932	7.22	0.00	6.89	GL29617	8.88	6.19	5.58	0.38	0.0004	q<0.01
GaLu96scf_22_118127_118753	12.35	10.66	12.13	GL22968	3.98	5.14	5.56	-0.37	0.0005	q<0.01
GaLu96scf_33_91707_92921	8.53	6.80	0.00	GL24196	1.13	0.88	1.15	-0.37	0.0005	q<0.01
GaLu96scf_11_699350_699504	6.17	0.00	6.62	GL18364	3.64	3.47	2.84	-0.37	0.0005	q<0.01
GaLu96scf_3_1902628_1903523	7.22	0.00	5.90	GL16882	8.76	8.78	8.06	-0.37	0.0005	q<0.01
GaLu96scf_15_838752_839281	0.00	7.76	6.43	GL21608	5.43	5.79	5.27	0.36	0.0005	q<0.01
GaLu96scf_11_965780_967233	9.73	4.60	0.00	GL20883	5.41	5.17	5.57	-0.36	0.0005	q<0.01
GaLu96scf_19_506883_507372	8.52	0.00	6.53	GL15931	7.87	8.33	8.61	-0.35	0.0006	q<0.01
GaLu96scf_15_821086_822351	6.23	6.46	6.62	GL21515	5.93	5.27	5.77	-0.35	0.0006	q<0.01
GaLu96scf_5_1092795_1093112	7.48	0.00	0.00	GL25357	6.56	6.47	6.93	-0.34	0.0007	q<0.01
GaLu96scf_1_3002925_3003256	9.98	5.38	4.77	GL30023	3.84	4.36	3.80	-0.34	0.0007	q<0.01
GaLu96scf_1_407026_407504	8.89	7.94	4.77	GL30095	6.60	7.31	6.57	0.33	0.0007	q<0.01
GaLu96scf_3_637440_638171	0.00	7.68	0.00	GL23952	3.51	2.23	1.90	-0.32	0.0008	q<0.01
GaLu96scf_5_420067_420267	0.00	0.00	7.58	GL25223	3.96	6.36	5.85	0.32	0.0009	q<0.01
GaLu96scf_31_39414_39859	9.16	0.00	5.75	GL24100	9.14	9.62	10.20	-0.32	0.0009	q<0.01
GaLu96scf_3_1242983_1243427	9.12	6.14	0.00	GL23880	5.10	3.91	4.51	0.31	0.0010	q<0.01
GaLu96scf_1_4775922_4776176	8.61	0.00	7.24	GL31086	5.71	5.62	5.57	0.31	0.0010	q<0.01
GaLu96scf_9_564822_565176	6.23	5.38	6.62	GL26766	3.94	3.35	3.41	0.30	0.0011	q<0.01
GaLu96scf_6_796087_796305	9.59	5.38	7.31	GL31330	5.43	5.57	5.94	-0.30	0.0012	q<0.01
GaLu96scf_8_549342_549577	8.49	0.00	5.75	GL25899	4.44	4.49	4.54	-0.30	0.0012	q<0.01
GaLu96scf_36_127166_127531	8.02	0.00	0.00	GL29251	3.83	3.74	4.13	-0.29	0.0013	q<0.01
GaLu96scf_14_989976_990323	7.22	6.55	5.90	GL15529	6.20	5.91	6.11	0.29	0.0014	q<0.01
GaLu96scf_12_451474_452244	10.69	9.34	6.89	GL18437	6.68	7.82	6.50	0.28	0.0015	q<0.01
GaLu96scf_1_2922471_2922676	9.09	5.38	7.03	GL30011	5.51	5.54	5.63	-0.28	0.0015	q<0.01

GaLu96scf_2_663567_664349	14.77	9.86	11.55	GL22656	5.02	4.96	4.52	0.27	0.0017	q<0.01
GaLu96scf_1_3517326_3518004	0.00	8.22	0.00	GL29481	3.17	6.93	8.31	0.26	0.0022	q<0.01
GaLu96scf_31_259187_259684	6.23	7.01	4.77	GL28295	3.68	3.11	3.05	0.26	0.0022	q<0.01
GaLu96scf_2_1682148_1682409	10.16	7.41	6.73	GL22369	2.95	3.26	2.92	-0.26	0.0022	q<0.01
GaLu96scf_5_1045707_1046124	7.80	7.63	0.00	GL25280	4.84	5.30	5.17	-0.25	0.0022	q<0.01
GaLu96scf_6_1277664_1278485	0.00	7.63	0.00	GL31640	4.28	4.86	5.08	0.25	0.0023	q<0.01
GaLu96scf_43_107810_108315	8.21	6.36	5.75	GL28634	3.99	5.94	3.90	-0.24	0.0028	q<0.01
GaLu96scf_15_577800_578307	10.82	8.04	8.52	GL21539	6.60	6.08	6.98	0.24	0.0028	q<0.01
GaLu96scf_16_207724_208080	9.53	4.60	0.00	GL21684	5.33	5.92	5.12	0.23	0.0031	q<0.01
GaLu96scf_6_1268432_1268833	8.85	4.60	0.00	GL31192	5.88	6.29	5.77	0.23	0.0033	q<0.01
GaLu96scf_2_979223_980204	0.00	9.95	8.11	GL27636	4.42	4.71	4.19	0.23	0.0035	q<0.01
GaLu96scf_8_866919_867522	7.75	0.00	5.75	GL25925	6.04	5.61	5.15	0.23	0.0035	q<0.01
GaLu96scf_9_1524047_1524338	6.81	4.60	6.73	GL26740	2.79	3.23	3.44	-0.23	0.0035	q<0.01
GaLu96scf_12_450878_451254	10.80	6.36	0.00	GL18437	6.68	7.82	6.50	0.22	0.0037	q<0.01
GaLu96scf_10_239610_240198	0.00	6.14	6.62	GL20604	3.84	3.77	3.86	-0.20	0.0052	q<0.01
GaLu96scf_22_118538_118753	6.81	0.00	7.31	GL22968	3.98	5.14	5.56	-0.20	0.0057	q<0.01
GaLu96scf_1_440858_441430	9.01	9.30	6.81	GL30097	4.89	5.96	5.34	0.20	0.0062	q<0.01
GaLu96scf_24_18576_19388	8.02	0.00	5.06	GL23161	5.35	4.17	1.90	0.19	0.0064	q<0.01
GaLu96scf_1_2168459_2168738	6.81	7.25	0.00	GL30250	7.35	8.02	7.83	-0.19	0.0069	q<0.01
GaLu96scf_1_1430262_1430474	9.02	4.60	5.75	GL30469	8.21	7.88	8.61	0.19	0.0072	q<0.01
GaLu96scf_11_612651_612944	8.98	6.36	0.00	GL20868	3.51	2.70	3.18	0.19	0.0073	q<0.01
GaLu96scf_26_117579_117907	9.57	0.00	5.06	GL16639	4.47	4.62	3.70	-0.18	0.0081	q<0.01
GaLu96scf_32_29553_29915	9.19	0.00	7.83	GL28308	4.01	3.90	3.79	0.17	0.0103	0.01<q<0.05
GaLu96scf_1_2965726_2965968	0.00	4.60	7.12	GL30637	6.44	7.50	6.44	0.17	0.0108	0.01<q<0.05
GaLu96scf_3_1674497_1674803	8.33	6.14	0.00	GL23899	3.26	2.65	3.00	0.16	0.0113	0.01<q<0.05

GaLu96scf_9_1006778_1007111	10.54	6.46	8.00	GL20397	2.66	2.69	3.78	-0.16	0.0120	0.01<q<0.05
GaLu96scf_16_219107_219575	7.54	7.36	0.00	GL15663	2.10	4.05	3.31	-0.16	0.0124	0.01<q<0.05
GaLu96scf_19_248473_248676	0.00	0.00	7.84	GL18854	3.47	2.02	2.55	-0.15	0.0145	0.01<q<0.05
GaLu96scf_17_630205_630493	7.22	0.00	6.62	GL21838	6.31	8.16	9.59	-0.15	0.0163	0.01<q<0.05
GaLu96scf_1_3294448_3295348	7.96	0.00	0.00	GL31004	4.79	4.51	5.28	-0.15	0.0164	0.01<q<0.05
GaLu96scf_29_261029_261362	7.54	0.00	7.53	GL23582	3.97	4.98	5.58	-0.15	0.0174	0.01<q<0.05
GaLu96scf_20_869159_869376	9.40	0.00	6.04	GL27716	4.73	4.48	3.90	0.14	0.0192	0.01<q<0.05
GaLu96scf_4_858934_859286	9.48	5.38	6.32	GL24604	3.61	3.48	4.19	-0.13	0.0234	0.01<q<0.05
GaLu96scf_11_1160952_1161458	7.96	0.00	0.00	GL20742	4.33	4.91	3.97	-0.13	0.0237	0.01<q<0.05
GaLu96scf_31_234899_235338	6.81	6.94	0.00	GL28294	5.82	4.56	5.03	0.13	0.0278	0.01<q<0.05
GaLu96scf_7_206066_206470	6.81	6.14	0.00	GL28915	4.21	4.92	4.59	-0.12	0.0293	0.01<q<0.05
GaLu96scf_32_314680_314960	6.23	0.00	7.05	GL24158	5.62	5.29	5.16	0.12	0.0299	0.01<q<0.05
GaLu96scf_3_575832_576089	6.75	5.38	7.63	GL23742	3.68	6.38	6.41	-0.12	0.0363	0.01<q<0.05
GaLu96scf_10_547494_547761	7.48	7.72	0.00	GL15096	1.54	2.28	1.99	-0.10	0.0521	no
GaLu96scf_40_120652_120959	0.00	7.01	5.06	GL24914	6.51	6.70	5.47	-0.10	0.0533	no
GaLu96scf_25_389997_390561	0.00	6.87	0.00	GL23279	1.17	2.65	3.70	0.10	0.0562	no
GaLu96scf_14_731709_732043	11.41	8.45	6.73	GL21362	7.65	7.99	7.63	-0.09	0.0655	no
GaLu96scf_27_566829_567492	7.74	0.00	0.00	GL16715	7.30	7.61	6.88	0.09	0.0793	no
GaLu96scf_2_807888_808986	7.54	0.00	0.00	GL22664	4.24	4.72	3.59	0.08	0.0888	no
GaLu96scf_2_176740_177035	10.39	8.54	9.52	GL15974	3.60	3.73	4.59	-0.08	0.0909	no
GaLu96scf_15_577641_578017	8.38	0.00	0.00	GL21539	6.60	6.08	6.98	0.08	0.1044	no
GaLu96scf_15_578105_578307	8.21	0.00	0.00	GL21539	6.60	6.08	6.98	0.08	0.1043	no
GaLu96scf_15_577955_578706	7.80	0.00	0.00	GL21539	6.60	6.08	6.98	0.08	0.1041	no
GaLu96scf_1_4533060_4533570	7.96	8.92	4.77	GL29573	3.27	5.13	4.69	-0.07	0.1166	no
GaLu96scf_15_577955_578307	8.77	6.36	5.90	GL21539	6.60	6.08	6.98	-0.07	0.1221	no

GaLu96scf_23_532213_532829	6.81	0.00	5.75	GL27852	2.63	3.08	3.67	-0.07	0.1399	no
GaLu96scf_17_791214_792475	8.38	7.07	8.03	GL21844	4.53	4.39	4.12	0.06	0.1871	no
GaLu96scf_10_1027170_1027578	7.54	0.00	0.00	GL15031	7.19	7.62	6.84	-0.05	0.2173	no
GaLu96scf_7_210293_210747	8.85	5.38	7.18	GL28919	5.02	4.95	6.82	0.05	0.2438	no
GaLu96scf_10_989288_989517	8.21	0.00	0.00	GL20653	2.54	2.18	2.96	-0.05	0.2458	no
GaLu96scf_8_997325_997534	7.54	0.00	4.77	GL25950	4.72	4.77	4.96	-0.05	0.2488	no
GaLu96scf_4_1283551_1283837	10.03	0.00	0.00	GL24640	10.13	10.43	9.77	0.04	0.3097	no
GaLu96scf_7_527164_527672	8.02	7.87	5.06	GL20180	3.76	3.27	3.51	0.04	0.3254	no
GaLu96scf_36_88635_89008	7.54	0.00	0.00	GL29224	2.99	2.42	3.61	-0.02	0.5749	no
GaLu96scf_36_89918_90195	7.54	0.00	0.00	GL29224	2.99	2.42	3.61	-0.02	0.5749	no
GaLu96scf_8_58909_59252	0.00	0.00	7.05	GL25940	3.01	1.86	2.45	0.02	0.6783	no
GaLu96scf_8_731051_731388	7.54	8.95	8.69	GL25920	5.15	5.29	4.88	-0.01	0.8519	no
GaLu96scf_6_579734_580190	8.38	9.19	8.03	GL31671	7.21	6.78	6.65	-0.01	0.8685	no

Table S14 Correaltion among the expression profiles of genes and their circRNAs and NAT, M: Mycelia; P: Primordia; FB: Fruiting Bodies.

Gene					circRNAs				NAT				Correlation		
**Gene ID**	**Strand**	**M**	**P**	**FB**	**Circ_ID**	**M**	**P**	**FB**	**NAT ID**	**M**	**P**	**FB**	**Gene/ circRNA**	**Gene /NAT**	**circRNA/ NAT**
GL15342	-	4.97	5.44	5.04	GaLu96scf_12_982987_983486	3.27	0.00	2.79	AT4960	5.78	6.55	6.01	-1.00	0.99	-0.99
GL18595	-	5.27	6.80	6.73	GaLu96scf_15_58153_58592	2.60	0.00	0.00	AT6784	4.75	6.53	6.44	-1.00	1.00	-1.00
GL22024	-	6.34	7.41	7.32	GaLu96scf_18_621762_622038	3.62	0.00	0.00	AT8665	2.80	4.87	5.18	-1.00	0.98	-0.99
GL17472	-	3.69	4.73	4.03	GaLu96scf_45_66710_67086	2.51	0.00	1.95	AT19850	0.00	7.53	6.95	-0.99	0.79	-0.72
GL26735	-	6.68	7.56	7.40	GaLu96scf_9_1475386_1476375	2.40	0.00	0.00	AT24838	5.32	6.55	6.52	-0.99	0.99	-1.00
GL21838	-	7.65	9.46	7.32	GaLu96scf_17_630205_630493	2.35	0.00	2.26	AT8108	10.56	11.82	9.84	-0.98	0.97	-0.92
GL31640	-	2.93	6.04	4.61	GaLu96scf_6_1278284_1278485	2.29	2.06	2.13	AT21915	4.49	6.72	5.75	-0.98	1.00	-0.98
GL22329	-	10.80	11.03	10.91	GaLu96scf_2_1057061_1057245	3.96	3.35	3.53	AT9898	10.63	10.62	10.58	-0.97	-0.26	0.51
GL24158	-	4.38	5.49	4.69	GaLu96scf_32_314680_314960	2.20	0.00	2.33	AT16613	4.42	5.49	4.60	-0.95	0.99	-0.98
GL24158	-	4.38	5.49	4.69	GaLu96scf_32_314680_314960	2.20	0.00	2.33	AT1661	3.79	4.19	2.51	-0.95	0.46	-0.72

									4						
GL17424	-	7.70	7.48	7.92	GaLu96scf_42_145252_145819	2.35	2.90	0.00	AT19552	14.32	12.17	13.40	-0.94	0.56	-0.26
GL20588	+	11.37	11.25	11.67	GaLu96scf_10_1396074_1396289	2.49	2.23	0.00	AT3892	8.26	7.86	8.44	-0.93	0.90	-0.67
GL30469	-	7.68	7.73	7.70	GaLu96scf_1_1430262_1430474	2.59	1.92	2.13	AT470	14.51	12.94	13.46	-0.90	-0.91	1.00
GL22329	-	10.80	11.03	10.91	GaLu96scf_2_1057061_1057450	3.80	3.34	3.38	AT9898	10.63	10.62	10.58	-0.89	-0.26	0.68
GL24214	-	2.98	3.80	3.52	GaLu96scf_33_107955_108150	3.75	3.43	3.68	AT16789	4.13	4.65	4.92	-0.87	0.78	-0.38
GL29950	-	5.17	5.26	5.35	GaLu96scf_1_2114443_2114867	2.39	0.00	0.00	AT691	5.24	5.00	5.33	-0.85	0.30	0.24
GL22329	-	10.80	11.03	10.91	GaLu96scf_2_1056816_1057450	2.40	0.00	0.00	AT9898	10.63	10.62	10.58	-0.85	-0.26	0.73
GL21156	-	5.97	6.70	6.27	GaLu96scf_13_937874_938103	2.42	0.00	0.00	AT5638	4.54	4.71	4.86	-0.81	0.43	-0.88
GL22329	-	10.80	11.03	10.91	GaLu96scf_2_1056816_1057245	3.12	2.23	2.13	AT9898	10.63	10.62	10.58	-0.80	-0.26	0.79
GL20637	-	4.08	4.33	4.74	GaLu96scf_10_738185_738462	2.48	0.00	0.00	AT3271	6.22	5.94	8.03	-0.79	0.87	-0.39
GL20637	-	4.08	4.33	4.74	GaLu96scf_10_738185_738462	2.48	0.00	0.00	AT3272	3.19	4.59	4.43	-0.79	0.72	-0.99
GL21632	-	4.55	4.11	5.08	GaLu96scf_16_257810_258051	2.45	1.92	0.00	AT7422	7.95	7.02	7.32	-0.78	0.27	0.40
GL23706	-	6.16	6.85	6.37	GaLu96scf_3_1917278_1917511	2.45	0.00	0.00	AT15373	4.67	2.45	3.71	-0.74	-0.99	0.82
GL23706	-	6.16	6.85	6.37	GaLu96scf_3_1917278_1917511	2.45	0.00	0.00	AT15374	2.97	0.00	0.00	-0.74	-0.74	1.00
GL23706	-	6.16	6.85	6.37	GaLu96scf_3_1917278_1917511	2.45	0.00	0.00	AT15377	4.87	8.00	8.32	-0.74	0.68	-1.00

GL30130	-	4.08	4.87	6.71	GaLu96scf_1_4460049_4460531	2.40	0.00	0.00	AT1428	3.64	4.58	6.63	-0.73	1.00	-0.74
GL22024	-	6.34	7.41	7.32	GaLu96scf_18_621638_622038	3.78	2.42	0.00	AT8665	2.80	4.87	5.18	-0.73	0.98	-0.84
GL28919	+	6.22	6.04	5.53	GaLu96scf_7_210293_210747	2.57	2.06	3.03	AT22955	2.73	3.18	1.82	-0.69	0.83	-0.98
GL16370	-	2.43	2.69	2.79	GaLu96scf_21_814520_814865	3.47	0.00	2.17	AT11745	1.50	2.39	2.12	-0.60	0.85	-0.93
GL30807	-	2.86	2.22	2.77	GaLu96scf_1_4437569_4437931	0.00	2.31	2.37	AT1419	8.05	4.51	6.68	-0.59	0.97	-0.78
GL27401	-	1.33	2.56	2.86	GaLu96scf_18_567176_567508	2.29	2.80	0.00	AT8641	0.70	1.73	3.20	-0.52	0.91	-0.83
GL17382	-	4.34	4.40	3.43	GaLu96scf_40_145087_145321	2.28	0.00	2.13	AT19294	5.26	4.53	4.89	-0.49	-0.05	0.89
GL20718	-	5.06	5.25	4.65	GaLu96scf_11_725031_725261	3.43	0.00	2.33	AT4143	9.69	8.56	8.49	-0.49	0.26	0.71
GL20718	-	5.06	5.25	4.65	GaLu96scf_11_725031_725261	3.43	0.00	2.33	AT4144	5.09	5.30	4.96	-0.49	0.94	-0.76
GL24170	-	6.01	6.38	5.08	GaLu96scf_32_269704_269946	3.64	0.00	2.17	AT16597	7.86	7.73	6.20	-0.38	0.94	-0.04
GL21283	-	1.56	2.17	2.18	GaLu96scf_14_997917_998788	2.29	3.06	0.00	AT6402	4.76	3.83	7.33	-0.30	0.28	-1.00
GL21283	-	1.56	2.17	2.18	GaLu96scf_14_997917_998788	2.29	3.06	0.00	AT6405	3.06	0.71	2.42	-0.30	-0.69	-0.48
GL21066	-	9.83	9.69	9.20	GaLu96scf_12_649243_649552	3.33	0.00	3.09	AT4843	7.84	7.70	7.24	-0.23	1.00	-0.23
GL24214	-	2.98	3.80	3.52	GaLu96scf_33_107500_108150	2.28	2.31	0.00	AT16790	3.57	3.80	4.45	-0.18	0.43	-0.96
GL24119	+	8.12	7.92	8.49	GaLu96scf_31_376798_377183	2.54	2.10	2.13	AT16463	7.30	7.30	8.01	-0.12	0.94	-0.45
GL30398	-	9.12	7.97	9.35	GaLu96scf_1_564557_564850	0.00	2.23	3.07	AT211	6.52	5.90	7.64	-0.10	0.86	0.42
GL30875	-	7.97	9.65	8.73	GaLu96scf_1_89101_89611	3.54	3.37	2.37	AT43	9.19	11.00	10.11	-0.08	1.00	-0.14
GL24469	-	6.31	6.51	6.16	GaLu96scf_4_119002_119433	2.42	0.00	0.00	AT18022	2.58	2.02	2.12	-0.06	-0.23	0.99

GL30586	-	4.27	3.90	4.60	GaLu96scf_1_2457103_2457612	2.52	0.00	0.00	AT800	4.84	4.34	4.86	0.02	0.89	0.48
GL30586	-	4.27	3.90	4.60	GaLu96scf_1_2457103_2457612	2.52	0.00	0.00	AT801	8.89	6.37	7.68	0.02	0.54	0.85
GL21921	-	8.36	7.12	7.50	GaLu96scf_17_302614_303070	2.58	3.19	0.00	AT8004	6.19	5.85	5.33	0.04	0.59	0.83
GL24196	-	1.31	1.77	1.58	GaLu96scf_33_91707_92921	2.53	2.95	0.00	AT16781	1.04	1.80	2.30	0.05	0.67	-0.71
GL25061	+	2.98	3.74	2.01	GaLu96scf_45_107155_107404	2.58	0.00	0.00	AT19926	7.86	8.03	4.84	0.07	0.92	0.46
GL30217	+	8.80	9.39	8.00	GaLu96scf_1_1744520_1744982	2.52	0.00	0.00	AT2161	7.47	8.38	6.58	0.09	1.00	-0.01
GL27629	-	6.63	6.86	6.27	GaLu96scf_2_728269_728479	2.50	0.00	0.00	AT9801	5.94	6.27	5.77	0.13	0.95	-0.17
GL28597	-	4.05	3.70	3.59	GaLu96scf_41_157014_157254	3.12	2.37	3.22	AT19468	4.67	4.16	4.44	0.17	0.67	0.84
GL20790	-	4.14	3.76	4.31	GaLu96scf_11_132103_132409	3.10	0.00	0.00	AT3939	8.90	8.09	8.86	0.22	0.94	0.53
GL24099	-	5.22	5.41	4.32	GaLu96scf_31_36002_36247	2.48	0.00	0.00	AT16175	9.66	8.45	8.25	0.36	0.47	0.99
GL24099	-	5.22	5.41	4.32	GaLu96scf_31_36002_36247	2.48	0.00	0.00	AT16176	9.88	7.90	6.32	0.36	0.73	0.90
GL22592	-	3.65	3.52	4.24	GaLu96scf_2_1770335_1770882	2.29	3.34	3.37	AT10151	5.99	5.21	5.41	0.37	-0.11	-0.96
GL22592	-	3.65	3.52	4.24	GaLu96scf_2_1770335_1770882	2.29	3.34	3.37	AT10154	2.44	2.52	3.21	0.37	0.96	0.61
GL23742	-	6.94	5.40	6.50	GaLu96scf_3_575832_576089	2.28	2.06	3.09	AT15001	5.46	4.77	5.09	0.43	0.96	0.15
GL21608	-	5.42	6.09	5.30	GaLu96scf_15_838752_839281	0.00	3.13	2.88	AT7008	5.96	6.97	6.00	0.44	0.99	0.59
GL28656	-	4.45	1.83	4.67	GaLu96scf_45_47325_47584	3.90	0.00	0.00	AT19845	5.89	1.57	6.15	0.44	1.00	0.46
GL28656	-	4.45	1.83	4.67	GaLu96scf_45_47325_47496	2.40	0.00	0.00	AT1984	5.89	1.57	6.15	0.44	1.00	0.46

									5						
GL19097	-	5.14	4.17	4.35	GaLu96scf_21_298566_298876	2.45	2.06	0.00	AT11587	7.07	6.11	6.13	0.48	0.99	0.61
GL19097	-	5.14	4.17	4.35	GaLu96scf_21_298566_298876	2.45	2.06	0.00	AT11589	2.68	3.55	3.27	0.48	-0.99	-0.35
GL26651	-	9.88	9.88	9.16	GaLu96scf_9_782358_782812	2.43	0.00	0.00	AT24605	8.81	8.62	8.00	0.50	0.98	0.68
GL28308	-	4.40	3.61	3.46	GaLu96scf_32_29553_29915	3.33	0.00	2.44	AT16513	9.97	6.84	7.57	0.59	0.93	0.85
GL20553	-	2.94	1.62	2.53	GaLu96scf_10_721197_721538	2.35	2.06	2.01	AT3261	2.07	2.18	2.40	0.63	-0.11	-0.84
GL23234	-	8.29	7.73	8.16	GaLu96scf_25_71903_72224	3.50	0.00	0.00	AT13187	9.86	8.94	9.74	0.67	1.00	0.60
GL22327	-	6.48	5.61	7.36	GaLu96scf_2_1034675_1034909	2.51	1.92	2.37	AT9891	5.55	4.25	6.57	0.73	1.00	0.78
GL22327	-	6.48	5.61	7.36	GaLu96scf_2_1034675_1034909	2.51	1.92	2.37	AT9892	5.99	5.28	6.93	0.73	1.00	0.67
GL30875	-	7.97	9.65	8.73	GaLu96scf_1_89438_89611	0.00	2.06	2.33	AT43	9.19	11.00	10.11	0.78	1.00	0.81
GL29359	-	5.12	3.87	4.55	GaLu96scf_1_1533440_1533730	3.08	2.19	2.13	AT519	4.84	5.17	5.60	0.81	-0.39	-0.86
GL29359	-	5.12	3.87	4.55	GaLu96scf_1_1533440_1533730	3.08	2.19	2.13	AT520	5.52	3.47	4.78	0.81	0.99	0.74
GL31640	-	2.93	6.04	4.61	GaLu96scf_6_1277664_1278485	0.00	3.11	0.00	AT21915	4.49	6.72	5.75	0.84	1.00	0.83
GL25996	-	8.06	6.05	7.11	GaLu96scf_8_531717_531891	3.41	0.00	0.00	AT23495	7.79	5.66	7.03	0.85	0.99	0.77
GL25996	-	8.06	6.05	7.11	GaLu96scf_8_531717_531891	3.41	0.00	0.00	AT23496	7.06	5.57	6.72	0.85	0.96	0.68
GL25417	-	5.50	5.16	5.34	GaLu96scf_5_356471_356897	2.48	0.00	0.00	AT20286	8.66	8.58	9.32	0.85	0.13	-0.42

GL20847	-	5.25	5.67	5.78	GaLu96scf_11_379444_380247	0.00	3.04	2.01	AT4021	8.93	7.73	7.49	0.86	-1.00	-0.88
GL21387	-	4.72	3.88	3.69	GaLu96scf_14_125920_126149	3.37	2.23	0.00	AT6134	6.37	4.22	4.97	0.86	0.87	0.49
GL30069	-	6.13	5.73	5.34	GaLu96scf_1_3550729_3551062	2.54	0.00	0.00	AT1148	13.36	9.50	10.61	0.87	0.70	0.96
GL26510	-	5.87	5.10	4.36	GaLu96scf_9_1418381_1418731	2.52	0.00	0.00	AT24809	7.71	6.87	6.16	0.87	1.00	0.89
GL26510	-	5.87	5.10	4.36	GaLu96scf_9_1418381_1418731	2.52	0.00	0.00	AT24810	8.36	6.91	6.81	0.87	0.90	1.00
GL28308	-	4.40	3.61	3.46	GaLu96scf_32_29345_29713	3.15	1.92	0.00	AT16515	7.37	3.28	4.24	0.88	0.93	0.64
GL27401	-	1.33	2.56	2.86	GaLu96scf_18_567176_568351	0.00	3.16	2.26	AT8641	0.70	1.73	3.20	0.89	0.91	0.62
GL30069	-	6.13	5.73	5.34	GaLu96scf_1_3551960_3552335	2.40	2.19	0.00	AT1150	4.97	5.14	6.49	0.90	-0.91	-1.00
GL25822	-	4.66	5.85	5.15	GaLu96scf_8_1022209_1023370	0.00	3.25	0.00	AT23662	3.32	3.41	3.44	0.91	0.69	0.32
GL15096	-	5.68	6.33	5.21	GaLu96scf_10_547494_547761	2.39	3.08	0.00	AT3206	3.95	2.05	2.49	0.92	-0.31	0.09
GL31640	-	2.93	6.04	4.61	GaLu96scf_6_1278057_1278485	3.35	3.81	3.73	AT21915	4.49	6.72	5.75	0.95	1.00	0.96
GL21921	-	8.36	7.12	7.50	GaLu96scf_17_301396_301681	3.22	0.00	0.00	AT8004	6.19	5.85	5.33	0.96	0.59	0.80
GL22726	-	3.47	5.29	7.77	GaLu96scf_20_845487_845780	0.00	2.31	3.43	AT11264	2.73	3.52	6.93	0.96	0.97	0.86
GL25940	-	2.34	2.87	4.36	GaLu96scf_8_58909_59252	0.00	0.00	2.33	AT23346	0.00	5.00	8.42	0.97	0.93	0.81
GL31148	-	5.31	5.35	5.34	GaLu96scf_6_1347523_1348145	0.00	3.04	1.95	AT21936	7.48	6.93	6.93	0.97	-0.99	-0.94
GL30422	-	6.85	5.28	4.88	GaLu96scf_1_84704_84952	2.46	0.00	0.00	AT41	8.09	6.12	5.99	0.98	0.99	1.00
GL30422	-	6.85	5.28	4.88	GaLu96scf_1_85022_85476	2.43	0.00	0.00	AT41	8.09	6.12	5.99	0.98	0.99	1.00

GL30422	-	6.85	5.28	4.88	GaLu96scf_1_83631_83927	2.48	0.00	0.00	AT41	8.09	6.12	5.99	0.98	0.99	1.00
GL22024	-	6.34	7.41	7.32	GaLu96scf_18_621638_621839	0.00	3.09	2.28	AT8665	2.80	4.87	5.18	0.98	0.98	0.93
GL18500	-	5.39	5.22	5.36	GaLu96scf_13_562681_563010	2.53	0.00	2.40	AT5532	11.41	9.74	10.59	0.99	0.95	0.89
GL18500	-	5.39	5.22	5.36	GaLu96scf_13_562681_563010	2.53	0.00	2.40	AT5534	4.65	3.77	4.20	0.99	0.95	0.88
GL21387	-	4.72	3.88	3.69	GaLu96scf_14_125920_126881	2.43	2.23	2.13	AT6136	4.90	4.28	3.76	0.99	0.96	0.99
GL17424	-	7.70	7.48	7.92	GaLu96scf_42_145252_145529	2.56	2.10	2.89	AT19552	14.32	12.17	13.40	0.99	0.56	0.65
GL25920	-	3.25	4.85	4.95	GaLu96scf_8_731051_731388	2.40	3.31	3.27	AT23578	3.18	4.30	4.18	1.00	0.99	1.00
GL23547	-	8.53	8.20	8.45	GaLu96scf_29_50957_51214	3.37	2.19	3.15	AT14517	5.12	4.92	5.36	1.00	0.70	0.73
GL28597	-	4.05	3.70	3.59	GaLu96scf_41_156524_157254	3.51	3.14	3.01	AT19468	4.67	4.16	4.44	1.00	0.67	0.67
